# Divergent Strain‐Release Amino‐Functionalization of [1.1.1]Propellane with Electrophilic Nitrogen‐Radicals

**DOI:** 10.1002/anie.202000140

**Published:** 2020-02-26

**Authors:** Ji Hye Kim, Alessandro Ruffoni, Yasair S. S. Al‐Faiyz, Nadeem S. Sheikh, Daniele Leonori

**Affiliations:** ^1^ Department of Chemistry University of Manchester Oxford Road Manchester M13 9PL UK; ^2^ Department of Chemistry College of Science King Faisal University P.O. Box 380 Al-Ahsa 31982 Saudi Arabia

**Keywords:** amino-functionalization, bioisosteres, nitrogen radicals, strain-release

## Abstract

Herein we report the development of a photocatalytic strategy for the divergent preparation of functionalized bicyclo[1.1.1]pentylamines. This approach exploits, for the first time, the ability of nitrogen‐radicals to undergo strain‐release reaction with [1.1.1]propellane. This reactivity is facilitated by the electrophilic nature of these open‐shell intermediates and the presence of strong polar effects in the transition‐state for C−N bond formation/ring‐opening. With the aid of a simple reductive quenching photoredox cycle, we have successfully harnessed this novel radical strain‐release amination as part of a multicomponent cascade compatible with several external trapping agents. Overall, this radical strategy enables the rapid construction of novel amino‐functionalized building blocks with potential application in medicinal chemistry programs as *p*‐substituted aniline bioisosteres.

## Graphical abstract

**Electrophilic nitrogen‐radicals** enable efficient strain‐release amino‐functionalization of [1.1.1]propellane. This radical reactivity can be used for the divergent assembly of 3‐substituted bicyclo[1.1.1]pentylamines, which are powerful aniline bioisosteres.

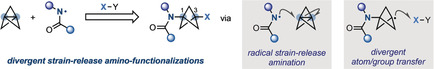

## Introduction

The bicyclo[1.1.1]pentane (BCP)^1^ is a rigid, linear, sp^3^‐rich motif frequently used in medicinal chemistry programs as bioisosteric replacement for aromatic rings, alkynes and *t*‐Bu groups.^2^ Bioactive molecules incorporating this substructural unit usually benefit from improved pharmacokinetic properties like lipophilicity and passive permeability.^3^

**Scheme 1:**
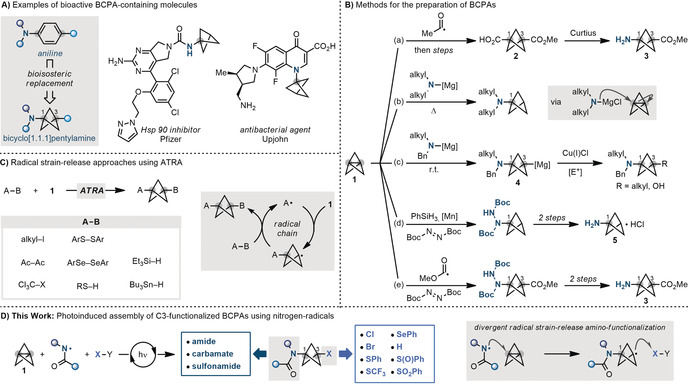
A) Bioactive molecules containing the BCPA motif. B) Previous ionic and radical approaches for the synthesis of BCPA building blocks. C) Formation of 1,3‐disubstituted BCPs using ATRA‐based systems. D) Proposed radical strategy for the divergent preparation of 3‐functionalized BCPAs using nitrogen‐radicals. Ac=acetyl, Bn=benzyl, Boc=*tert*‐butyloxycarbonyl.

In particular, bicyclo[1.1.1]pentylamines (BCPAs) are of considerable interest as aniline bioisosteres for the preparation of novel small‐molecule therapeutics with resistance towards metabolic clearance (Scheme 1 A).^4^ Since the pioneering work of Wiberg^5^ and Szeimies,^6^ few multi‐steps approaches towards these building blocks have been developed, generally relying on the three steps conversion of [1.1.1]propellane **1** into **2** followed by Curtius rearrangement to give **3** and further elaboration (Scheme 1 B, path a).^7^ A breakthrough in the field has come in 2016 when Baran demonstrated that di‐alkyl amines, upon prior deprotonation with *i*‐PrMgCl⋅LiCl, react with **1** by ionic strain‐release thus enabling a straightforward construction of mono‐substituted BCPAs (Scheme 1 B, path b).^8^

In contrast, the preparation of C3‐substituted BCPAs is much more difficult and still go through long multi‐step synthesis.^9^ In an effort to streamline the preparation of these building blocks, Gleason has very recently merged ionic strain‐release amination with Cu^I^‐catalysis using activated electrophiles to assemble C3 alkylated and hydroxylated systems (Scheme 1 B, path c).^10^ This telescoped process requires the accumulation of the magnesiated‐BCPA intermediates **4**, prior addition of the Cu^I^/electrophile, thus restricting the source of N‐partners to benzylic amines.

Radical strain‐release amino‐functionalization would represent an attractive alternative to target the preparation of these building blocks. So far, the only approach for the direct preparation of BCPAs is based on the amination of C3 BCP radicals with N‐based SOMOphiles as developed by Pfizer.^11^ In this case, radical hydro‐hydrazination of **1** using DEAD as the SOMOphile has enabled, upon further elaboration, the scalable preparation of building block **5** in three steps (Scheme 1 B, path d). More recently Uchiyama has extended this approach to the preparation 3‐functionalized BCPAs through a carbo‐hydroazidation strategy using alkyl carbazates/aryl hydrazines as radical precursors and DEAD as radical trap (Scheme 1 B, path e).^12^ Also in this case, the C−N bond is formed after strain‐release reaction with acyl/aryl radicals, and therefore only hydrazine‐type substituents can be introduced. Albeit this multicomponent strategy has not been extended to any other class of radical traps, it has significantly shortened access to common the BCPAs intermediate **3**, which can be further elaborated.

The difficulties in accessing C3‐substituted BCPAs contrast with the ease of preparation of C3‐substituted 1‐alkyl‐BCPs. Indeed, carbon‐, sulfur‐ and, to a lesser extent, silicon‐ and tin‐radicals have been widely used in ATRA‐based (atom‐transfer radical addition) strategies to generate, in a single step, 1,3‐disubstituted BCPs (Scheme 1 C).^5^, ^7^, ^13^ In this case however, the inherent mechanistic requirement for the chain carrying radical to be regenerated from the precursor in the final atom/group transfer step, does not offer option of divergency. Furthermore, despite the reach radical strain‐release chemistry, no example of a nitrogen‐radical able to engage **1** in direct strain‐release amination has so far been reported.

In view of our previous experience on the use of nitrogen‐radicals in olefin amino‐functionalization,^14^ we recently questioned whether it would be possible to use these species in strain‐release settings. Despite there is no example of nitrogen‐radical addition to [1.1.1]propellane **1**, we reasoned that its successful implementation had the potential to streamline access to important BCPA building blocks currently elusive from other approaches. Critical to our design would be the ability to intercept the C3 BCPA radical resulting from strain‐release amination with different SOMOphiles. Divergent radical functionalizations of [1.1.1.]propellane **1** have, to the best of our knowledge, not been reported, but their implementation would provide significant expansion in the structure and scope of the process. Herein, we demonstrate that amidyl radicals undergo efficient strain‐release reaction with **1** and that this reactivity can be harnessed as part of a strategy leading to the multicomponent assembly of 3‐functionalized BCPAs (Scheme 1 D). This approach displays significant synthetic complementarity to ionic strain‐release amination as it enables, for the first time, the direct introduction of amide, carbamate and sulfonamide functionality across the BCP core. The fact that this process does not rely on a radical‐chain propagation mechanism has enabled the diversification of the BCPAs with six different SOMOphiles that are not compatible with any of the previous amination technologies. Furthermore, as amidyl radicals display a distinct electrophilic character, this cascade reactivity represents an umpolung approach to ionic strain‐release amination and, to the best of our knowledge, the first example of a radical process enabling divergent strain‐release‐amino‐functionalization.

## Results and Discussion

### Reaction Design and Analysis

**Scheme 2:**
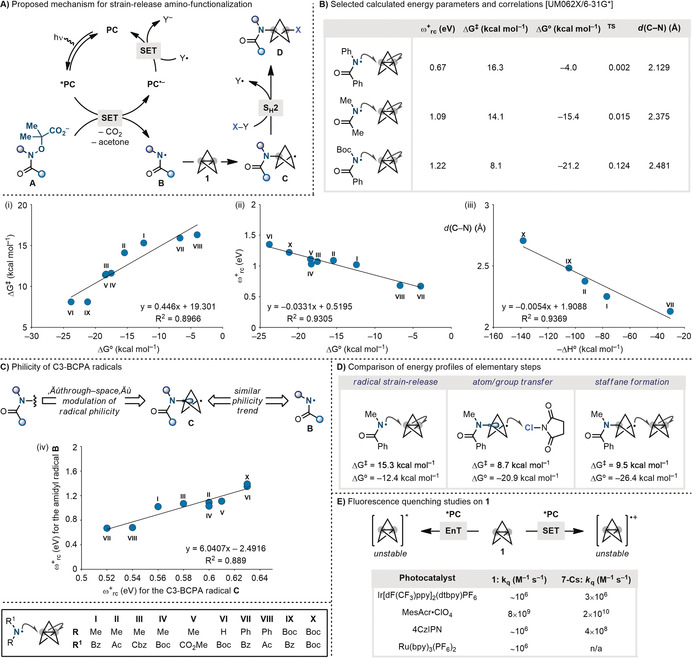
A) Proposed mechanism for strain‐release with nitrogen‐radicals. B) Computational studies on the strain‐release reaction of nitrogen radicals. C) Computational studies on the character of C3 BCPA radicals. D) Comparison of energy profiles for the elementary steps in the radical cascade, compared with the side reactivity leading to staffane. E) Stern–Volmer analyses of **1** with common photocatalysts. Bz=benzoyl, Cbz=benzyloxycarbonyl.

From the outset we recognized that the realization of a divergent strain‐release amino‐functionalization process with nitrogen‐radicals would require developing a three‐component cascade compatible with external SOMOphiles. We therefore postulated a strategy based on a reductive quenching photoredox cycle (Scheme 2 A). Under these settings, a visible‐light excited photocatalyst (*PC)^15^ would oxidize the carboxylate functionality of the radical precursor **A**, triggering two fragmentations (extrusion of CO_2_ and acetone) and forming the amidyl radical **B**.^16^ As this species has a distinct electrophilic character, we were hopeful that it would be able to intercept electron rich **1** by cleaving its central and inverted sp^3^‐sp^3^ C−C bond. This polarized radical strain‐release amination would assemble the key C−N bond and give the C3 BCPA radical **C**.^17^ This species would be then diversified by atom/group‐transfer reaction (S_H_2) with a range of SOMOphiles (X−Y) providing the targeted building blocks **D**. At the end, the electron poor radical Y^.^ would render the process redox‐neutral by closing the photoredox cycle by SET with the reduced photocatalyst (**PC^.−^**). As mentioned before, as amidyl radicals are electrophilic species, this process represents the first example of an umpolung strain‐release amination. This is mechanistically interesting because, while both nucleophiles and nucleophilic radicals can participate in strain‐release chemistry, umpolung reactions can only be conducted via radical approaches owing to the known instability of BCP cations.^18^

As strain‐release reactions of nitrogen‐radicals have not been reported before, we performed computational studies to evaluate the feasibility of the proposed reactivity (Scheme 2 B).^19^ Overall, our investigations demonstrated that these reactions share close similarities to the intermolecular additions of amidyl radicals to olefins. They are increasingly exothermic the more electrophilic is the nitrogen‐radical (determined using their electrophilicity indices, ω^+^_rc_) and the barrier for strain‐release decrease with increasing reaction exothermicity. Furthermore, there is a good correlation between both the exothermicity (Δ*G*°) and the barrier (Δ*G* ^≠^) of the reaction (Scheme 2 B, chart i) and Δ*G*° and the electrophilicity of the incoming nitrogen‐radical (Scheme 2 B, chart ii). Owing to the electron rich nature of **1** (IP=9.7 eV),^20^ there is strong participation of polar effects in the transition state (|δ^TS^|) due to the net charge transfer with the electrophilic amidyl radicals. Finally, there is also a linear correlation between bond formation distance [*d*(C−N)] and the reaction enthalpy, which is in agreement with the Hammond postulate (Scheme 2 B, chart (iii)).^21^ Overall, these computational findings suggest radical strain‐release reactions of amidyl radicals are characterized by early transition states and the enthalpy governs to some extent the transition state geometry.

Despite these encouraging features, it is important to note that the success of the strategy does not hinge solely on the feasibility of the strain‐release reaction of amidyl radical **B** (**1+B→C**) but also on the following atom/group‐transfer reaction (**C**+X‐Y→**D**). In fact, this step is required to be efficient in order to avoid the known and facile oligomerization of BCP‐radicals with other molecules of **1** that leads to common staffane by‐products. Indeed, while exploring the reactivity of S‐radicals, Scaiano demonstrated that despite these species undergo facile strain‐release reaction with **1**, their reactivity could only be unlocked in systems benefitting from very efficient ATRA‐based chain propagations to stop staffane formation.^22^ As our proposed reactivity relies on a multicomponent radical cascade where the amidyl radical generation is dissected by the product formation, there is a fundamental mechanistic requirement for the final atom/group‐transfer step to be kinetically preferred over BCP‐radical oligomerization.^5^

In general, BCP radicals display enhanced reactivity towards SOMOphiles compared to tertiary radicals (e.g. *t*‐Bu^.^) because of their (i) permanent pyramidal arrangement, (ii) reduced steric shielding and (iii) increased SOMO s‐character.^23^ In our case however, we were concerned that due to the very short C1–C3 cross‐cage distance, the electronegative amide functionality could retard the C3 BCPA‐radical reactivity with polarized SOMOphiles by inferring a through‐space negative kinetic polar effect that could instead favor the polarity matched oligomerization.^23^, ^24^ Indeed, our calculations have demonstrated that C3 BCPA radicals **C** are significantly less nucleophilic than 1‐alkyl‐BCP ones and that their electrophilicity closely mirrors the one of the incoming amidyl radicals **B** (Scheme 2 C, chart iv). This supports the presence of strong cross‐cage interaction leading to a remote through‐space modulation of their radical philicity. Nevertheless, Cl‐transfer from *N*‐chlorosuccinimide (NCS) was calculated to have a slightly lower barrier than staffane formation, thus supporting the feasibility of the overall cascade reactivity (Scheme 2 D).

Before starting the optimization of the amino‐functionalization strategy, we decided to evaluate the behavior of **1** in the presence of several photocatalysts. As this species is electron rich and has also demonstrated to undergo energy transfer (EnT) with triplet benzophenone,^25^ we wanted to evaluate the ease of formation of **1^.+^**/1*, that suffer fast decomposition. These Stern–Volmer studies demonstrated **1** undergoes appreciable bimolecular quench only with the strongly oxidant Fukuzumi's acridinium (*E_ox_ >2.1 V vs. SCE) (Scheme 2 E). Importantly, the deprotonated amidyl radical precursors **A** undergo luminescence quenching at significant higher rates with all photocatalyst evaluated,^16^, ^21^ which ensure the overall feasibility of our proposed photoredox manifold.

### Reaction Development and Scope

**Scheme 3:**
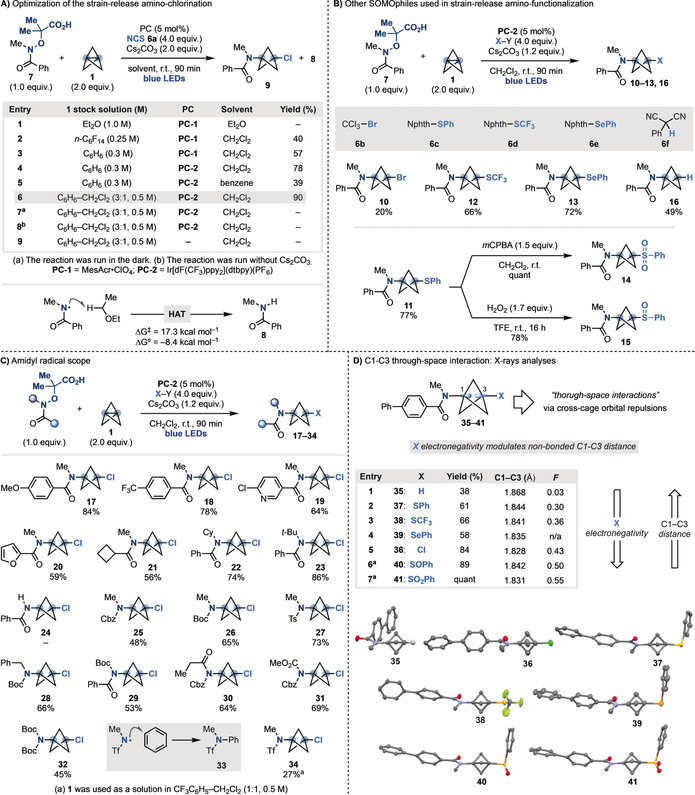
A) Optimization of the strain‐release amino‐functionalization using amidyl radical precursor **5** and NCS. B) SOMOphile compatible to the process. C) Scope and limitation of the process. D) X‐ray analysis on C3‐functionalized BCPA building blocks. Nphth=phtalimide, *m*CPBA=*meta*‐chloroperbenzoic acid, Cy=cyclohexyl, Ts=toluenesulfonyl, Tf=trifluoromethanesulfonyl.

The amino‐functionalization strategy was first evaluated using **7** as the amidyl radical precursor, NCS (**6 a**) as the SOMOphile, Cs_2_CO_3_ as the base and Fukuzumi's acridinium (**PC‐1**) as the photocatalyst (*E_ox_ >2.1 V vs. SCE)^26^ under blue‐light irradiation (*λ*=420 nm) in CH_2_Cl_2_ solvent (Scheme 3 A). The first challenge to address was the preparation of **1**. Baran has demonstrated its large‐scale synthesis as stock solution in Et_2_O,^8^ however, as amidyl radicals are very electrophilic, they undergo efficient H‐atom transfer (HAT)^27^ from this solvent. Indeed, all efforts to optimize the proposed reactivity using **1** as 1.0 m Et_2_O solution resulted in quantitative formation of *N*‐methyl‐benzamide **8** (entry 1). We therefore had to attempt the preparation of **1** in different solvents to exclude the presence of hydridic H‐atom donors. Pleasing, when the reaction was run using **1** as a 0.25 m solution in perfluoro‐*n*‐hexane, 3‐Cl‐BCPA **9** was obtained in 40 % yield albeit in combination with several side products arising from staffane‐formation and inefficient Cl‐transfer (entry 2). Crucially, by co‐distilling **1** in benzene (entry 3) and changing the photocatalyst to the less oxidative Ir[dF(CF_3_)ppy_2_](dtbpy)(PF_6_) (**PC‐2**) (*E_ox_=1.69 V vs. SCE) (entry 4), we progressively improved the yield to 78 %. Interestingly, when the reaction was completely run in benzene, **9** was obtained in lower efficiency, thus pointing to a favorable solvent effect played by CH_2_Cl_2_ (entry 5). Co‐distillation of **1** with CH_2_Cl_2_ was attempted but found not feasible as this solution underwent decomposition over just 1 h at room temperature. We therefore evaluated the preparation of **1** in various benzene:CH_2_Cl_2_ mixtures assessing their stability over time. Eventually we developed a procedure for its reproducible and large‐scale preparation as a 0.5 m solution in a 3:1 benzene:CH_2_Cl_2_ mixture.^21^ This stock solution can be stored for over a month at low temperature (−5 °C under nitrogen in the dark) and displays sufficient stability at room temperature for handling (>1 day). The use of this propellane source was critical for the implementation of the strain‐release cascade and **9** was obtained in 90 % yield (entry 6). Control experiments confirmed the requirement for both photocatalyst, base and continuous blue‐light irradiation (entries 7–9).

Having identified optimal conditions for strain‐release amino‐chlorination, we next explored the possibility to render this strategy divergent using other SOMOphiles (Scheme 3 B). While NBS lead to a complex reaction mixture, Br‐CCl_3_ **6 b** provided the amino‐bromination product **10** in 20 % yield albeit in the presence of staffane by‐products. We rationalized this outcome on the basis of a more challenging Br‐transfer reaction (calculated barrier: Δ*G* ^≠^=11.2 kcal mol^−1^) that can be outcompeted by the oligomerization. Phthalimide‐based reagents **6 c**–**e** were efficient for the introduction of S‐ and Se‐functionalities (**11**–**13**). Building block **11** was further modified by simple oxidation providing access to BCPAs **14** and **15** that contain a C3‐sulfone and sulfoxide, respectively. Finally, several HAT catalysts were evaluated to achieve the formation of amide‐containing BCPA **16** that cannot be accessed by ionic strain‐release. While the commonly used methyl thioglycolate resulted in complex reaction mixtures, 2‐Ph‐malononitrile^28^ **2 f** provided **16** in good yield.

The amidyl radical substitution pattern was evaluated next using NCS **6 a** as the SOMOphile. As shown in Scheme 3 C, *N*‐Me‐benzamides containing both electron donating (**17**) and electron withdrawing (**18**) substituents were tolerated without loss in reaction efficiency. Moreover, the presence of heteroaromatic (**19** and **20**) and alkyl (**21**) substituents did not have a deleterious impact on the reaction performance.

The *N*‐substituent was also modified and we successfully obtained **22** and **23** that contain sterically demanding *N*‐cyclohexyl and *N*‐*t*‐Bu groups. Primary BCPAs (e.g. **24**) currently represent a limitation of the methodology as the reaction with NH‐amidyl radicals gave consistently complex mixtures.

The strategy was then extended to more electrophilic carbamoyl radicals based on commonly used protecting groups like *N*‐Cbz (**25**), *N*‐Boc (**26**) and *N*‐Ts (**27**). BCPA **28** contains a *N*‐Boc,*N*‐Bn substituent and can be orthogonally deprotected and modified. Pleasingly, we also succeeded in engaging nitrogen‐radicals containing two carbonyl substituents (**29**–**32**). Their ability to successfully take part in the strain‐release reactivity is noteworthy as these species are known to undergo facile intramolecular addition to aromatics (e.g. benzene).^29^ This side reactivity became particularly problematic when extending the chemistry to the more electrophilic *N*‐Tf,*N*‐Me radical. In this case, we observed complete formation of the corresponding *N*‐Ph derivative **33** with no trace of the targeted BCPA. Pleasingly, we overcome this hurdle by preparing **1** as a solution in CF_3_C_6_H_5_/CH_2_Cl_2_. This electron poor aromatic co‐solvent suppressed the unwanted *N*‐arylation and enabled the assembly of **34** albeit in moderate yield.^30^

An interesting structural feature of BCP‐based building blocks is that despite their rigid and compact nature, the C1–C3 non‐bonded distance changes depending on the electronic nature of their substituents. In particular, electronegative atoms/groups inductively remove electron density from the bridgehead carbons rear‐lobe orbitals and, by decreasing orbital repulsion, shorten their distance.^31^ As the impact of a nitrogen substituent on a 3‐substituted‐BCP‐core has never been assessed, we prepared BCPAs **35**–**41** containing a *p*‐Ph‐benzamide that enabled growing of crystals suitable for X‐ray analysis (Scheme 3 D). These substrates display progressively shorter C1–C3 cross‐cage distances upon increasing electronegativity of the C3 substituent up to a very short 1.828 Å in the case of **36**. A good correlation was found between these distances and the field/inductive parameters (*F*) for the various C3‐substitutents, which supports the presence of strong through‐space C1–C3 interactions.^21^, ^32^

## Conclusion

In conclusion, we have demonstrated that electrophilic nitrogen‐radicals are powerful intermediates to achieve multicomponent strain‐release amino‐functionalization reactions of [1.1.1]propellane **1** using external SOMOphiles. To the best of our knowledge, the process reported here represents the first example of a divergent radical strain‐release‐functionalization strategy. The ability to engage a range of different SOMOphiles has enabled the assembly of novel building blocks with potential application for the preparation of *p*‐substituted aniline bioisosteres. DFT calculations have given an insight into the interplay of both enthalpic, polar and through‐space effects operating in the strain‐release reaction of nitrogen‐radicals and the following functionalization of the resulting C3‐BCPA radicals. Furthermore, we have demonstrated that inter‐molecular H‐atom transfer, a common side reactivity of electrophilic radicals, can be effectively overcome with the preparation of [1.1.1.]propellane **1** in CH_2_Cl_2_/benzene solution. We hope that these insights might lead to the rational design of related strain‐release functionalizations using other classes of electrophilic radical species.

## Conflict of interest

The authors declare no conflict of interest.

## Supporting information

As a service to our authors and readers, this journal provides supporting information supplied by the authors. Such materials are peer reviewed and may be re‐organized for online delivery, but are not copy‐edited or typeset. Technical support issues arising from supporting information (other than missing files) should be addressed to the authors.

Supplementary
