# Supplementary material for: Divergent Strain‐Release Amino‐Functionalization of [1.1.1]Propellane with Electrophilic Nitrogen‐Radicals
Source: Angew Chem Int Ed Engl. 2020 Feb 26;59(21):8225–31. doi: 10.1002/anie.202000140 (PMC7318212; doi:10.1002/anie.202000140)
Supplement: Supplementary file 1 — Supplementary [file ANIE-59-8225-s001.pdf]

Angewandte Chemie
Eine Zeitschrift der Gesellschaft Deutscher Chemiker

Supporting Information

Divergent Strain-Release Amino-Functionalization of [1.1.1]Propellane with Electrophilic Nitrogen-Radicals

Ji Hye Kim+, Alessandro Ruffoni+, Yasair S. S. Al-Faiyz, Nadeem S. Sheikh, and Daniele Leonori**

anie_202000140_sm_miscellaneous_information.pdf

1 General Experimental Details ... 3

2 Starting Material Synthesis ... 4

3 Propellane Preparation ... 12

4 Emission Quenching Experiments ... 14

5 Pictures of Reaction Set-Up ... 17

6 Reaction Products ... 18

7 X-Ray Crystal Structures ... 29

8 Correlations ... 31

8.1 Radical Strain-Release Step ... 31

8.2 Atom/Group-Transfer Step ... 34

8.3 Side Reactions ... 36

9 Computational Studies ... 37

9.1 Computational Methods ... 37

9.2 Activation Energy ($\Delta G^{\neq}$) and Reaction Energy (ΔG) *39*

9.3 Electronic Properties of Radicals *99*

9.4 Reaction Enthalpy (– ΔH_r), Bond distance [d(C–C)] and Charge Transfer (δ^{TS}) for Radical Addition Reactions *119*

9.5 Electronic Properties of Propellane and Alkenes *141*

10 NMR Spectra 145

11 References: 219

1 General Experimental Details

All required fine chemicals were used directly without purification unless stated otherwise. All air and moisture sensitive reactions were carried out under nitrogen atmosphere using standard Schlenk manifold technique. THF was distilled from sodium/benzophenone, CH_2Cl_2 and was distilled from CaH_2, CH_3CN was distilled from activated 4Å molecular sieves, EtN(*i*-Pr)$_2$ was distilled over KOH. ^{1}H and ^{13}C Nuclear Magnetic Resonance (NMR) spectra were acquired at various field strengths as indicated and were referenced to $CHCl_3$ (7.26 and 77.0 ppm for 1H and 13C respectively). ^{1}H NMR coupling constants are reported in Hertz and refer to apparent multiplicities and not true coupling constants. Data are reported as follows: chemical shift, integration, multiplicity (s = singlet, br s = broad singlet, d = doublet, t = triplet, q = quartet, qi = quintet, sx = sextet, sp = septet, m = multiplet, dd = doublet of doublets, etc.), proton assignment (determined by 2D NMR experiments: COSY, HSQC and HMBC) where possible. High-resolution mass spectra were obtained using a JEOL JMS-700 spectrometer or a Fissions VG Trio 2000 quadrupole mass spectrometer. Spectra were obtained using electron impact ionization (EI) and chemical ionization (CI) techniques, or positive electrospray (ES). Infra-red spectra were recorded using a JASCO FT/IR 410 spectrometer or using an ATI Mattson Genesis Seris FTIR spectrometer as evaporated films or liquid films. Analytical TLC: aluminum backed plates pre-coated (0.25 mm) with Merck Silica Gel 60 F254. Compounds were visualized by exposure to UV-light or by dipping the plates in permanganate ($KMnO_4$) stain followed by heating. Flash column chromatography was performed using Merck Silica Gel 60 (40–63 μm). All mixed solvent eluents are reported as v/v solutions. UV/Vis spectra were obtained using an Agilent 6453 spectrometer and 1 mm High Precision Cell made of quartz from Hellma Analytics. The LEDs used are Kessil H150-blue. All the reactions were conducted in CEM 10 mL glass microwave tubes.

2 Starting Material Synthesis

GP1 – General Procedure for Synthesis of Carboxylic Acids 7, 7a–j

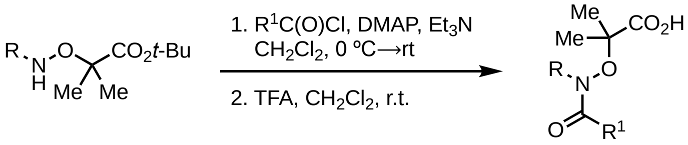

Step 1) To a solution of α-oxy-*tert*-butyl ester (1.0 equiv.) in CH_2Cl_2 (0.2 M) was added DMAP (0.25 equiv.) and Et_3N (2.0 equiv.). After cooling to 0 °C, the acid chloride (1.1 equiv.) was added dropwise to the solution and the reaction mixture was allowed to warm to room temperature overnight. The reaction was diluted with sat. aq. $NaHCO_3$ and extracted with CH_2Cl_2. The combined organic layers were dried ($MgSO_4$), filtered and evaporated. The crude was purified by column chromatography on silica gel eluting with petrol–EtOAc.

Step 2) The *tert*-butyl ester was diluted with CH_2Cl_2 (0.1 M), treated with TFA (15 equiv.) and stirred at room temperature. Once the reaction was complete by TLC analysis (30–60 mins), the reaction mixture was evaporated and the crude was purified by column chromatography on silica gel eluting with petrol–EtOAc.

2-Methyl-2-((*N*-methylbenzamido)oxy)propanoic Acid (7)

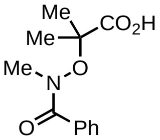

Following **GP1**, **7** (4.30 g) was obtained in 53% yield as a solid. FT-IR νmax (film)/cm^{-1} 2998, 1735, 1657, 1323, 1111, 978, 705; ^{1}H NMR (500 MHz, $CDCl_3$) δ 7.56–7.52 (3H, m), 7.49–7.46 (2H, m), 3.41 (1H, s), 1.67 (2H, s); ^{13}C NMR (126 MHz, $CDCl_3$) δ 174.89, 174.10, 132.27, 132.02, 128.99, 127.93, 87.21, 43.17, 24.39; HRMS (ESI) Found MNa^{+} 259.0828, $C_{12}H_{14}NO_4Na$ requires 259.0821.

2-((4-Methoxy-*N*-methylbenzamido)oxy)-2-methylpropanoic Acid (7a)

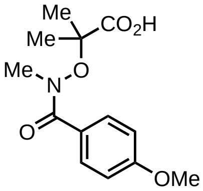

Following **GP1**, **7a** (864 mg) was obtained in 32% yield as a solid. FT-IR νmax (film)/cm^{-1} 1739, 1603, 1512, 1384, 1304, 1254, 1172, 1025, 961, 841, 758; ^{1}H NMR (400 MHz, $CDCl_3$) δ 7.52 (2H, d, J = 8.8 Hz), 6.95 (2H, d, J = 8.8 Hz), 3.86 (3H, s), 3.43 (3H, s), 1.64 (6H, s);

^{13}C NMR (101 MHz, $CDCl_3$) δ 175.2, 174.3, 162.6, 130.3, 123.9, 114.2, 86.9, 55.7, 43.7, 24.1; HRMS (ESI) Found MNa^+ 290.0984, $C_{13}H_{17}O_5NNa$ requires 290.0999.

2-Methyl-2-((*N*-methyl-4-(trifluoromethyl)benzamido)oxy)propanoic Acid (7b)

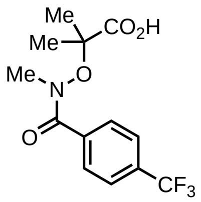

Following **GP1**, **7b** (393 mg) was obtained in 32% yield as a solid. FT-IR νmax (film)/cm^{-1} 2978, 1756, 1621, 1432, 1201, 1121, 755; ^1H NMR (500 MHz, $CDCl_3$) δ 7.75 (2H, d, *J* = 8.3 Hz), 7.67 (2H, d, *J* = 8.1 Hz), 3.40 (3H, s), 1.65 (6H, s); ^{13}C NMR (101 MHz, $CDCl_3$) δ 174.8, 172.4, 136.1, 133.69 (q, J = 32.7 Hz), 128.5, 126.0, 123.49 (q, J = 272.6 Hz), 87.1, 42.5, 24.3; HRMS (ESI) Found MNa^+ 327.0683, $C_{13}H_{13}NO_4F_3Na$ requires 327.0694.

2-((6-Chloro-N-methylnicotinamido)oxy)-2-methylpropanoic Acid (7c)

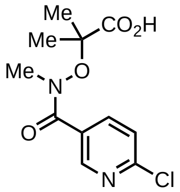

Following **GP1**, **7c** (228 mg) was obtained in 20% yield as a solid. FT-IR νmax (film)/cm^{-1} 2360, 1733, 1635, 1585, 1456, 1134, 1106, 910, 841, 754; ^1H NMR (400 MHz, $CDCl_3$) δ 9.69 (1H, bs), 8.67 (1H, d, *J* = 2.4 Hz), 7.96 (1H, dd, *J* = 8.3, 2.4 Hz), 7.43 (1H, d, *J* = 8.3 Hz), 3.44 (3H, s), 1.49 (6H, s); ^{13}C NMR (101 MHz, $CDCl_3$) δ 175.1, 169.5, 154.0, 149.5, 139.3, 128.5, 124.4, 85.9, 40.6, 23.8; HRMS (ESI) Found MH^+ 273.0623, $C_{11}H_{14}O_4N_2Cl$ requires 273.0637.

2-Methyl-2-((N-methylfuran-2-carboxamido)oxy)propanoic Acid (7d)

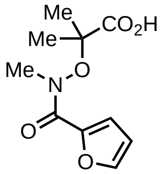

Following **GP1**, **7d** (418 mg) was obtained in 92% yield as a solid. FT-IR νmax (film)/cm^{-1} 2991, 2360, 1737, 1601, 1471, 1388, 1134, 1017, 939, 898, 758; ^1H NMR (500 MHz, $CDCl_3$) δ 7.60 (1H, dd, *J* = 1.7, 0.9 Hz), 7.31 (1H, dd, *J* = 3.6, 0.9 Hz), 6.58 (1H, dd, *J* = 3.6, 1.8 Hz),

3.69 (3H, s), 1.61 (6H, s); ^{13}C NMR (126 MHz, $CDCl_3$) δ 175.0, 162.1, 146.0, 144.5, 120.4, 112.5, 87.2, 42.2, 24.3; HRMS (ESI) Found MH^+ 228.0855, $C_{10}H_{14}O_5N$ requires 228.0866.

2-(((*N*,4-Dimethylphenyl)sulfonamido)oxy)-2-methylpropanoic Acid (7e)

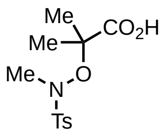

Following **GP1**, **7e** (850 mg) was obtained in 78% yield as an off-white solid. ^{1}H NMR (500 MHz, $CDCl_3$) δ 7.75 (2H, d, *J* = 7.9 Hz), 7.37 (2H, d, *J* = 8.0 Hz), 2.86 (3H, s), 2.46 (3H, s), 1.62 (6H, s); ^{13}C NMR (126 MHz, $CDCl_3$) δ 176.9, 145.3, 130.1, 129.2, 84.6, 42.0, 24.3, 21.9; HRMS (ESI) Found MH^+ 288.0917, $C_{12}H_{18}O_5NS$ requires 288.0906.

2-Methyl-2-((*N*-methylcyclobutanecarboxamido)oxy)propanoic Acid (7f)

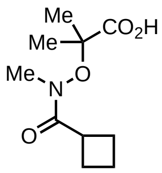

Following **GP1**, **7f** (381 mg) was obtained in 89% yield as an oil. FT-IR νmax (film)/cm^{-1} 2945, 2360, 1737, 1608, 1386, 1290, 1178, 1134, 976, 759; ^{1}H NMR (400 MHz, $CDCl_3$) δ 13.88 (1H, bs), 3.27 (3H, s), 3.32–3.17 (1H, m), 2.45–2.31 (2H, m), 2.29–2.16 (2H, m), 2.10–1.87 (2H, m), 1.55 (6H, s); ^{13}C NMR (101 MHz, $CDCl_3$) δ 177.0, 175.2, 86.9, 40.2, 36.4, 24.8, 24.3, 18.1; HRMS (ESI) Found MH^+ 216.1221, $C_{10}H_{18}O_4N$ requires 216.1230.

2-((*N*-((Benzyloxy)carbonyl)propionamido)oxy)-2-methylpropanoic Acid (7g)

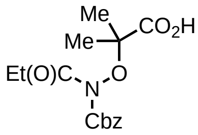

Following **GP1**, **7g** (540 mg) was obtained in 74% yield as an oil. FT-IR νmax (film)/cm^{-1} 2360, 1748, 1456, 1384, 1275, 1210, 1116, 972, 751, 698; ^{1}H NMR (400 MHz, $CDCl_3$) δ 12.40 (1H, bs), 7.62–7.29 (5H, m), 5.32 (2H, s), 2.94 (2H, q, *J* = 7.3 Hz), 1.45 (6H, bs), 1.20 (3H, t, *J* = 7.2 Hz); ^{13}C NMR (101 MHz, $CDCl_3$) δ 177.1, 174.2, 152.9, 133.7, 129.6, 129.2, 129.1, 89.1, 70.5, 30.6, 24.9, 21.7; HRMS (ESI) Found MNa^+ 332.1089, $C_{15}H_{19}O_6NNa$ requires 332.1105.

2-((((Benzyloxy)carbonyl)(methoxycarbonyl)amino)oxy)-2-methylpropanoic Acid (7h)

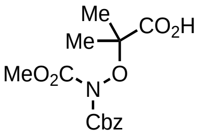

Following **GP1**, **7h** (370 mg) was obtained in 40% yield as an oil. FT-IR νmax (film)/cm^{-1} 2988, 1748, 1608, 1386, 1178, 962, 851, 745, 698; ^{1}H NMR (400 MHz, $CDCl_3$) δ 7.44–7.34 (5H, m), 5.34 (2H, s), 3.95 (3H, s), 1.52 (6H, s); ^{13}C NMR (101 MHz, $CDCl_3$) δ 174.1, 154.3, 153.4, 134.1, 129.2, 128.9, 128.7, 88.6, 70.6, 55.5, 23.2; HRMS (ESI) Found MNa^{+} 334.0883, $C_{14}H_{17}O_7NNa$ requires 334.0897.

2-Methyl-2-((N-methyl-[1,1'-biphenyl]-4-carboxamido)oxy)propanoic acid (7i)

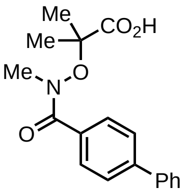

Following **GP1**, **7i** (1.27 g) was obtained in 26% yield as a solid. FT-IR νmax (film)/cm^{-1} 2360, 1748, 1456, 1384, 1275, 1210, 1116, 972, 751, 698; ^{1}H NMR (400 MHz, $CDCl_3$) δ 7.69 (2H, d, J=8.0 Hz), 7.66–7.57 (4H, m), 7.52–7.39 (3H, m), 3.47 (3H, s), 1.69 (6H, s); ^{13}C NMR (101 MHz, $CDCl_3$) δ 174.9, 174.1, 145.0, 139.7, 130.8, 129.2, 128.6, 128.5, 127.6, 127.4, 87.2, 43.4, 24.1 (bs); HRMS (ESI) Found MNa^{+} 336.1192, $C_{18}H_{19}O_4NNa$ requires 336.1206.

2-Methyl-2-(((1,1,1-trifluoro-*N*-methylmethyl)sulfonamido)oxy)propanoic Acid (7j)

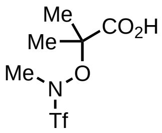

Following **GP1**, **7j** (670 mg) was obtained in 61% yield as an off-white solid. ^{1}H NMR (500 MHz, $CDCl_3$) δ 3.40 (3H, s), 1.60 (6H, s); ^{13}C NMR (126 MHz, $CDCl_3$) δ 176.5, 121.7 (q, J = 328.7 Hz), 85.6, 42.0, 23.6; ^{19}F NMR (376 MHz, $CDCl_3$) δ –68.1 HRMS (ESI) Found MH^{+} 266.0315, $C_6H_{11}F_3O_5NS$ requires 266.0310.

GP2 – General Procedure for Synthesis of Carboxylic Acids 7k–m

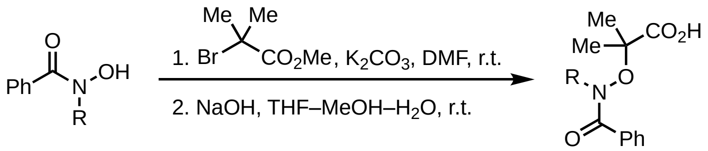

Step 1) A solution of *N*-hydroxybenzamide[1] (1.0 equiv.) in DMF (2.0 M) was treated with K_2CO_3 (1.05 equiv.) and methyl 2-bromo-2-methylpropanoate (1.05 equiv.). The reaction mixture was stirred at r.t. overnight. The reaction was diluted with brine and Et_2O. The organic layer was separated, dried ($MgSO_4$), filtered and evaporated. The crude was purified by column chromatography on silica gel eluting with petrol–EtOAc.

Step 2) The methyl ester was diluted with THF–MeOH (0.3 M, 2:1), treated with NaOH (2.0 equiv. in a small amount of H_2O) and stirred at room temperature. Once the reaction was complete, the reaction mixture was diluted in CH_2Cl_2 and washed with HCl_{aq} (1 N). The organic layers were separated, dried ($MgSO_4$), filtered and evaporated. The crude was purified by column chromatography on silica gel eluting with petrol–EtOAc.

2-((*N*-Cyclohexylbenzamido)oxy)-2-methylpropanoic Acid (7k)

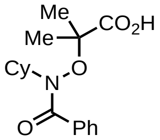

Following **GP2**, **7k** (1221 mg) was obtained in 18% yield as a solid. FT-IR νmax (film)/cm^{-1} 2935, 2858, 2359, 2341, 1748, 1622, 1448, 1398, 1178, 1144, 953, 697, 670; ^{1}H NMR (400 MHz, $CDCl_3$) δ 13.96 (1H, s), 7.62–7.39 (5H, m), 3.83–3.66 (1H, m), 1.94–1.46 (7H, m), 1.64 (6H, s) (6H, s), 1.19–0.98 (3H, m); ^{13}C NMR (101 MHz, $CDCl_3$) δ 177.2, 175.5, 133.3, 132.2, 129.1, 127.7, 87.3, 64.0, 30.9, 25.6, 25.0, 24.1; HRMS (ESI) Found MNa^+ 328.1506, $C_{17}H_{23}O_4NNa$ requires 328.1519.

2-((*N*-(*tert*-Butyl)benzamido)oxy)-2-methylpropanoic Acid (7l)

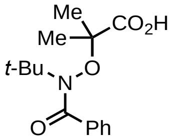

Following **GP2**, **7l** (1006 mg) was obtained in 14% yield as a solid. FT-IR νmax (film)/cm^{-1} 2984, 1748, 1608, 1367, 1175, 929, 749, 702; ^{1}H NMR (400 MHz, $CDCl_3$) δ 12.89 (1H, bs), 7.59–7.48 (3H, m), 7.48–7.38 (2H, m), 1.62 (6H, s), 1.23 (9H, s); ^{13}C NMR (126 MHz,

$CDCl_3$) δ 180.7, 175.6, 136.4, 132.2, 128.6, 128.4, 86.7, 64.7, 30.2, 24.0; HRMS (ESI) Found MH^- 278.1387, $C_{15}H_{20}O_4N$ requires 278.1398.

2-((((Benzyloxy)carbonyl)(methyl)amino)oxy)-2-methylpropanoic Acid (7m)

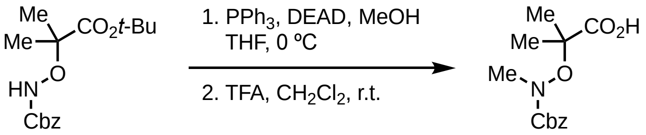

Step 1) A solution of *tert*-butyl 2-((((benzyloxy)carbonyl)amino)oxy)-2-methylpropanoate[2] (1.0 equiv.), MeOH (1.05 equiv.) and PPh_3 (2.0 equiv.) in THF (0.05 M) was cooled to 0 °C and treated with DEAD (1.5 equiv.). The mixture was stirred at stirred at 0 ºC for 2 h and then evaporated. The crude was purified by column chromatography on silica gel eluting with petrol-EtOAc.

Step 2) The *tert*-butyl ester was diluted with CH_2Cl_2 (0.1 M), treated with TFA (15 equiv.) and stirred at room temperature. Once the reaction was complete by TLC analysis (30–60 mins), the reaction mixture was evaporated and the crude was purified by column chromatography on silica gel eluting with petrol–EtOAc to give **7m** (664 mg, 34%) as a solid. FT-IR νmax (film)/cm^{-1} 2990, 2360, 1705, 1455, 1384, 1337, 1140, 1027, 748, 697; ^{1}H NMR (500 MHz, $CDCl_3$) δ 12.26 (1H, bs), 7.45–7.29 (5H, m), 5.24 (2H, s), 3.23 (3H, s), 1.56 (6H, s); ^{13}C NMR (126 MHz, $CDCl_3$) δ 174.9, 161.4, 134.8, 129.0, 128.9, 128.6, 86.5, 69.8, 40.8, 24.3; HRMS (ESI) Found MNa^+ 290.0984, C13H17O5NNa requires 290.0999.

GP 3 – General Procedure for the preparation of 7n–o

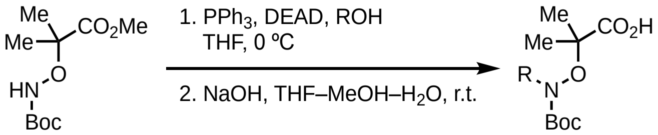

Step 1) A solution of methyl 2-(((*tert*-butoxycarbonyl)amino)oxy)-2-methylpropanoate[2] (1.0 equiv.), the alcohol (1.05 equiv.) and PPh_3 (2.0 equiv.) in THF (0.05 M) was cooled to 0 °C and treated with DEAD (1.5 equiv.). The mixture was stirred at stirred at 0 ºC for 2 h and then evaporated. The crude was purified by column chromatography on silica gel eluting with petrol-EtOAc.

Step 2) The methyl ester was diluted with THF–MeOH (0.3 M, 2:1), treated with NaOH (2.0 equiv. in a small amount of H_2O) and stirred at room temperature. Once the reaction was complete, the reaction mixture was diluted in CH_2Cl_2 and washed with HCl_{aq} (1 N). The

organic layers were separated, dried ($MgSO_4$), filtered and evaporated. The crude was purified by column chromatography on silica gel eluting with petrol–EtOAc.

2-(((*tert*-Butoxycarbonyl)(methyl)amino)oxy)-2-methylpropanoic Acid (7n)

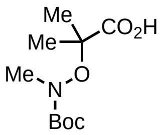

Following **GP3**, **7n** (1.97 g) was obtained in 65% yield as an oil. FT-IR νmax (film)/cm–1 1742, 1704, 1368, 1257, 1143, 848, 772; ^{1}H NMR (400 MHz, $CDCl_3$) δ 3.16 (3H, s), 1.54 (6H, s), 1.50 (9H, s); ^{13}C NMR (101 MHz, $CDCl_3$) δ 175.5, 160.9, 86.2, 84.9, 41.0, 28.2; HRMS (ESI) Found MH^{-} 232.1185, $C_{10}H_{18}O_5N$ requires 232.1190.

2-((Benzyl(*tert*-butoxycarbonyl)amino)oxy)-2-methylpropanoic Acid (7o)

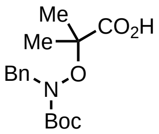

Following **GP3**, **7o** (278 mg) was obtained in 33% yield as an oil. FT-IR νmax (film)/cm^{-1} 2981, 2359, 1748, 1683, 1368, 1246, 1148, 749, 700; ^{1}H NMR (400 MHz, $CDCl_3$) δ 12.74 (1H, bs), 7.39–7.27 (5H, m), 4.61 (2H, s), 1.49 (6H, s), 1.46 (9H, s); ^{13}C NMR (101 MHz, $CDCl_3$) δ 175.3, 160.4, 135.8, 128.7, 128.6, 128.3, 86.5, 85.1, 57.3, 28.2, 24.2; HRMS (ESI) Found MNa^{+} 332.1455, $C_{16}H_{23}O_5NNa$ requires 332.1468.

2-((*N*-(*tert*-Butoxycarbonyl)benzamido)oxy)-2-methylpropanoic acid (7p)

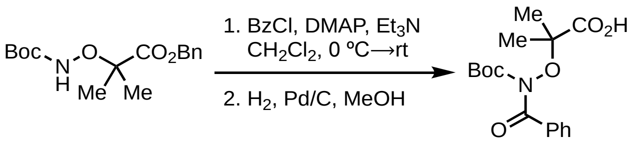

Step 1) To a solution of benzyl 2-(((*tert*-butoxycarbonyl)amino)oxy)-2-methylpropanoate (1.0 equiv.) in CH_2Cl_2 (0.2 M) was added DMAP (0.25 equiv.) and Et_3N (2.0 equiv.). After cooling to 0 °C, benzoyl chloride (1.1 equiv.) was added dropwise to the solution and the reaction mixture was allowed to warm to room temperature overnight. The reaction was diluted with sat.aq. $NaHCO_3$ and extracted with CH_2Cl_2. The combined organic layers were dried ($MgSO_4$), filtered and evaporated. The crude was purified by column chromatography on silica gel eluting with petrol–EtOAc.

Step 2) To a solution of benzyl ester (1.0 equiv.) in MeOH (0.2 M) was added Pd/C (10 wt % on charcoal, 10% by weight). The resulting suspension was stirred under H_2 (balloon pressure) overnight, then filtered through a plug of celite. The filtrate was evaporated and the crude was purified by column chromatography on silica gel eluting with petrol–EtOAc to give **7p** (647 mg, 98%) as a solid. FT-IR νmax (film)/cm^{-1} 2982, 2360, 1756, 1706, 1450, 1369, 1307, 1223, 1146, 957, 834, 695, 670; ^{1}H NMR (400 MHz, $CDCl_3$) δ 7.66–7.61 (2H, m), 7.61–7.54 (1H, m), 7.46 (2H, t, J=7.7 Hz), 1.65 (6H, s), 1.22 (9H, s); ^{13}C NMR (101 MHz, $CDCl_3$) δ 174.4, 171.7, 153.3, 134.5, 133.0, 128.6, 128.5, 88.6, 87.4, 27.5, 23.8; HRMS (ESI) Found MNa$^+$ 346.1244, $C_{16}H_{21}O_6NNa$ requires 346.1261.

2-((Bis(*tert*-butoxycarbonyl)amino)oxy)-2-methylpropanoic Acid (7q)

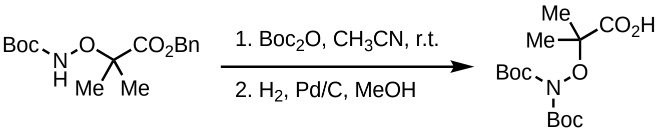

Step 1') To a solution of α-oxybenzyl ester (1.0 equiv.) in MeCN (0.5 M) was added Boc_2O (2.0 equiv.) and DMAP (0.3 equiv.). The reaction mixture was stirred at room temperature overnight. The solvent was removed and the crude was purified by column chromatography on silica gel eluting with petrol–EtOAc to give **7q** (900 mg, 86%) yield as a solid. FT-IR νmax (film)/cm^{-1} 2998, 1773, 1585, 1513, 1391, 1234, 1189; ^{1}H NMR (500 MHz, $CDCl_3$) δ 1.58 (6H, s), 1.56 (18H, s); ^{13}C NMR (126 MHz, $CDCl_3$) δ 174.6, 153.0, 88.4, 86.6, 28.1, 23.5; HRMS (ESI) Found MNa$^+$ 341.1455, $C_{14}H_{24}NO_7Na$ requires 341.1450.

Caesium 2-Methyl-2-((*N*-methylbenzamido)oxy)propanoate (7-Cs)

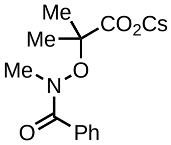

To a solution of α-oxymethyl ester (838 mg, 3.3 mmol, 1.0 equiv.) in THF (1.0 M) was added 1 N aq. CsOH (3.3 mL, 3.3 mmol, 1.0 equiv.). The mixture was stirred for 15 minute at room temperature and then concentrated under reduced pressure. Toluene (3 mL) was added and evaporated (x 3) to give **7-Cs** as a solid (1.21 g, 99%). FT-IR νmax (film)/cm^{-1} 2923, 1746, 1664, 1334, 1127, 912, 734 ; ^{1}H NMR (500 MHz, d_6-DMSO) δ 7.59 (2H, d, J = 7.2 Hz), 7.43–7.35 (3H, m), 3.31 (3H, s), 0.92 (6H, s); ^{13}C NMR (126 MHz, d_6-DMSO) δ 173.6, 169.6, 135.7, 129.7, 128.6, 127.5, 86.2, 36.7, 24.3; HRMS (ESI) Found M$^-$ 236.0918, $C_{12}H_{14}NO_4$ requires 236.0928.

3 Propellane Preparation

1,3-Diiodobicyclo[1.1.1]pentane (S1)

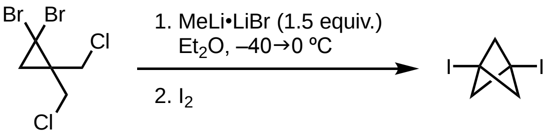

A solution of 1,1-dibromo-2,2-bis(chloromethyl)cyclopropane (21 g, 70.6 mmol, 1.0 equiv.) in Et_2O (33 mL) under argon was cooled to –40 ºC and treated with MeLi•LiBr by slow dropwise (100 mL, 148 mmol, 2.1 equiv., 1.5 M in Et_2O) maintaining the temperature below –30 ºC. The reaction was then warmed to 0 ºC and stirred at the same temperature for 3 h. Sublimed iodine crystals (18.5 g, 74 mmol, 1.1 equiv.) were slowly added maintaining the temperature below 0 ºC. After the addition was completed the reaction was slowly allowed to reach r.t. and stirred overnight. The mixture was diluted with EtOAc and $Na_2S_2O_3$. The layers were separated and the organic layer was washed with brine and dried ($MgSO_4$). After filtration through a pad of celite the solution was concentrated to a minimum amount of solvent and stored at –20 ºC overnight. Off white crystals of **S1** were formed are collected by filtration (13.5 g, 42 mmol, 60%). ^{1}H NMR (500 MHz, $CDCl_3$) δ 2.67 (6H, s); ^{13}C NMR (126 MHz, $CDCl_3$) δ 68.1. Data in accordance with the literature.[3]

Propellane (1)

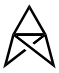

To a stirred solution of NaCN (1.69 g, 34.5 mmol, 2.2. equiv.) in sulfolane (50 mL) at 30 ºC under an Ar atmosphere (balloon to avoid evaporation of propellane) in a flame dry falsk was added **S1** (5.00 g, 15.6 mmol, 1.0 equiv.) and the mixture was stirred for 1 h. The flask was connected to a trap pre-cooled to –78 ºC as in Figure S1 and CH_2Cl_2 (12 mL) was slowly added. Distillation of volatile material from 70 to 0 mbar was maintained for 1 h, while the reaction flask was kept at room temperature. After completion of the distillation the cooling trap was warmed to 0 ºC and benzene (12 mL) was added. **1** was collected as solution in CH_2Cl_2–benzene (1:1) and approximate concentration of the solution (0.5 M) was calculated using quantitative NMR . The solution was stored at –20 ºC and its concentration did not change over a period of 1 month.

Propellane stock solution in DCM : Fluorobenzene, DCM : Nitrobenzene, DCM : Trifluorotoluene, have been prepared following the same procedure adding different aromatic

solvent instead of benzene. Stability of all of them have been tested over 2 weeks at -20 C and they did not show any sign of degradation.

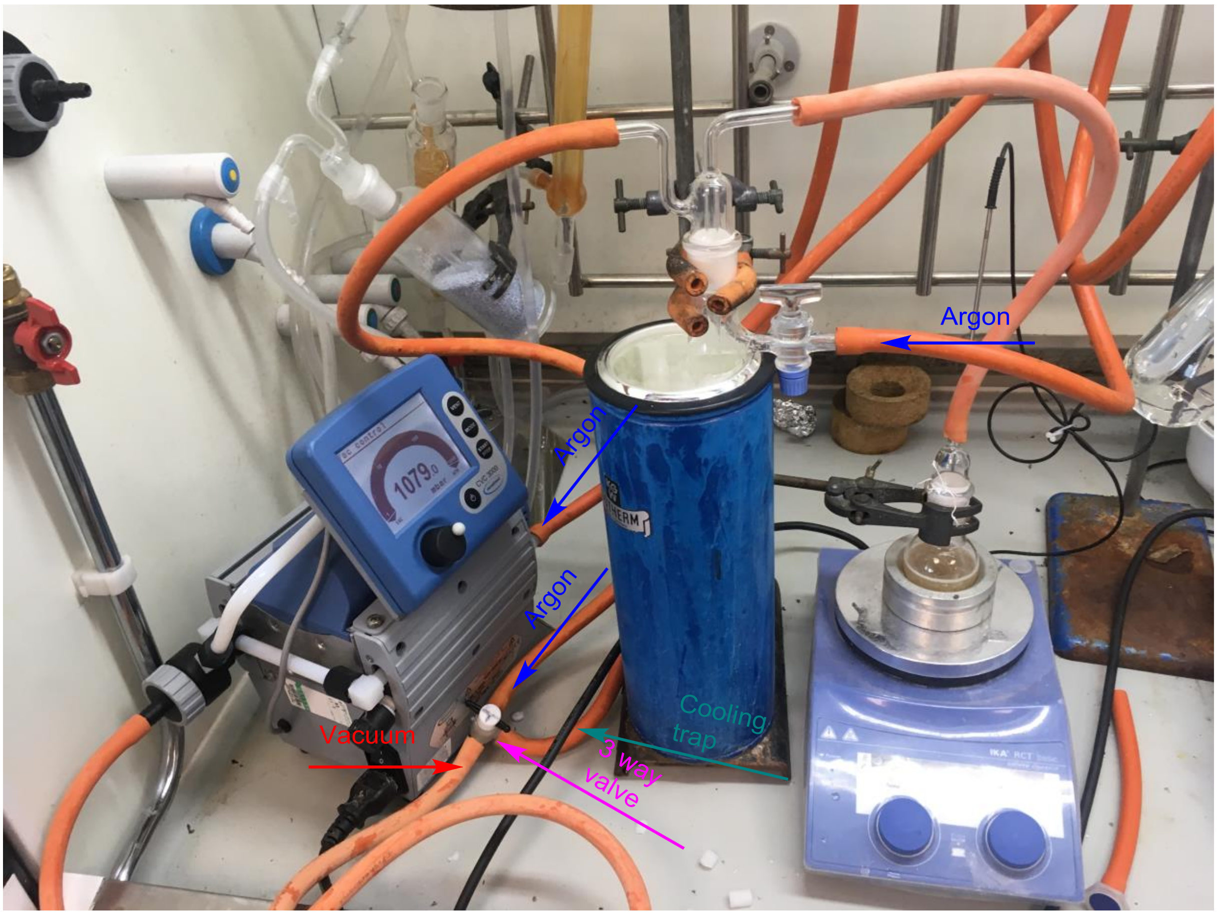

Figure S1.

Quantitative ^{1}H NMR Spectroscopy Experiments

An oven dried NMR tube was charged with **1** (200 μL, as a CH_2Cl_2–benzene (1:1) solution), DCE (50 μL) and $CDCl_3$ (500 μL). The molar ration of **1**:DCE was determined by ^{1}H NMR spectroscopy and used to extrapolate the concentration of the stock solution of **1**.

4 Emission Quenching Experiments

Emission intensities were recorded using a Steady-State emission spectra were recorded on an Edinburgh Instrument FP920 Phosphorescence Lifetime Spectrometer equipped with a 5 watt microsecond pulsed xenon flash lamp and a 450 watt steady state xenon lamp and a red sensitive photomultiplier in peltier (air cooled) housing, (Hamamatsu R928P) spectrophotometer.

Photocatalyst	Solutions excited at	Emission intensity collected at
$Ir[dF(CF_3)ppy]_2(dtbpy))PF_6$	380	466
$MesAcr•ClO_4$	430	510
4CzIPN	435	540
$Ru(bpy)_3(PF_6)_2$	450	600

Experimental procedures:

A screw-top quartz cuvette was charged with a 0.01 mM solution of the photocatalyst in CH_2Cl_2 (2.0 mL) and the initial emission was collected then the appropriate amount of the quencher as a 0.1 M solution in CH_2Cl_2 was added. The sample was shaken for 1 min and then the emission of the sample was collected.

For **7**–Cs and NCS the experiments have been conducted in DMSO instead of CH_2Cl_2 due to solubility issue.

The quenching constants were obtained using the Stern-Volmer relationship:

$$I_0/I = 1 + k_q\tau_0[\text{quencher}]$$

Photocatalyst	Quencher	k_q (M^{-1} s^{-1})
$Ir[dF(CF_3)ppy]_2(dtbpy))PF_6$	**7**–Cs	2.6 10^{7}
$Ir[dF(CF_3)ppy]_2(dtbpy))PF_6$	**1**	6.8 10^{6}
$MesAcr•ClO_4$	**1**	7.9 10^{9}
4CzIPN	**1**	4.1 10^{6}
$Ru(bpy)_3(PF_6)_2$	**1**	8.1 10^{6}

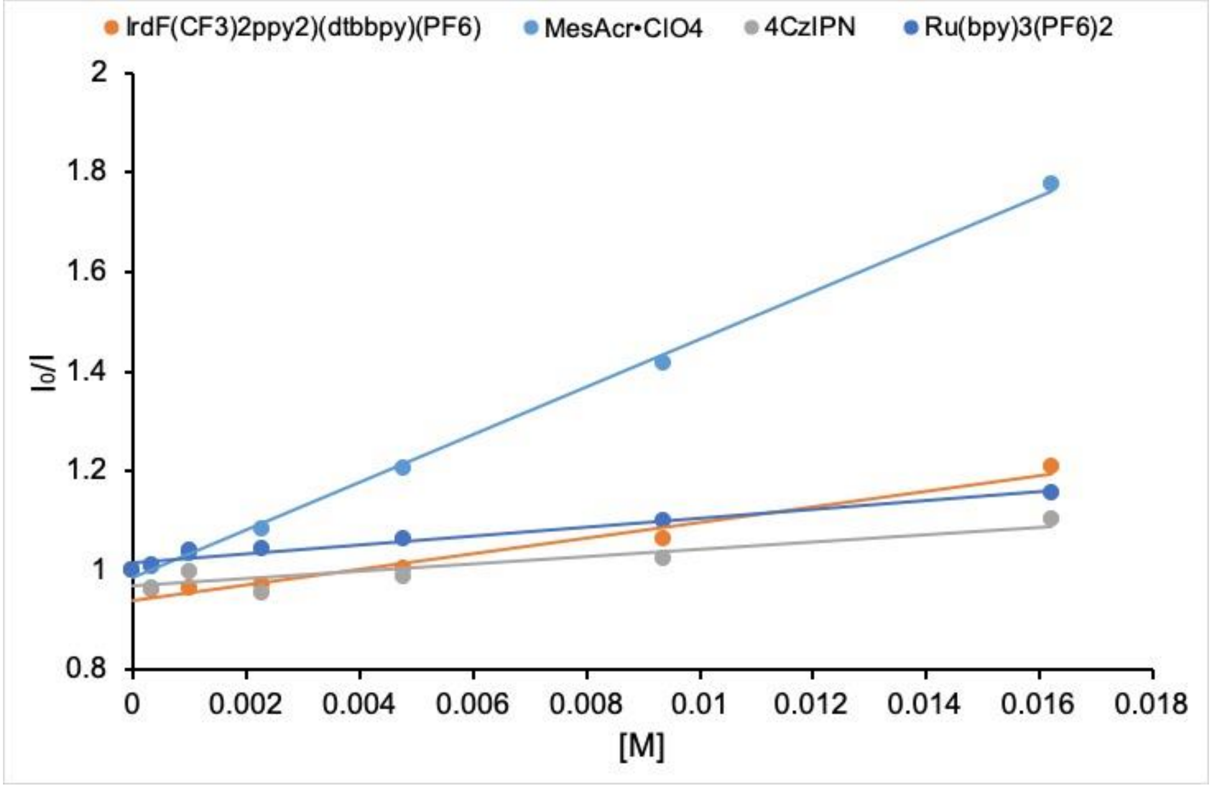

Scheme S1. Stern-Volmer plot for the quench of excited photocatalysts by **1**.

\

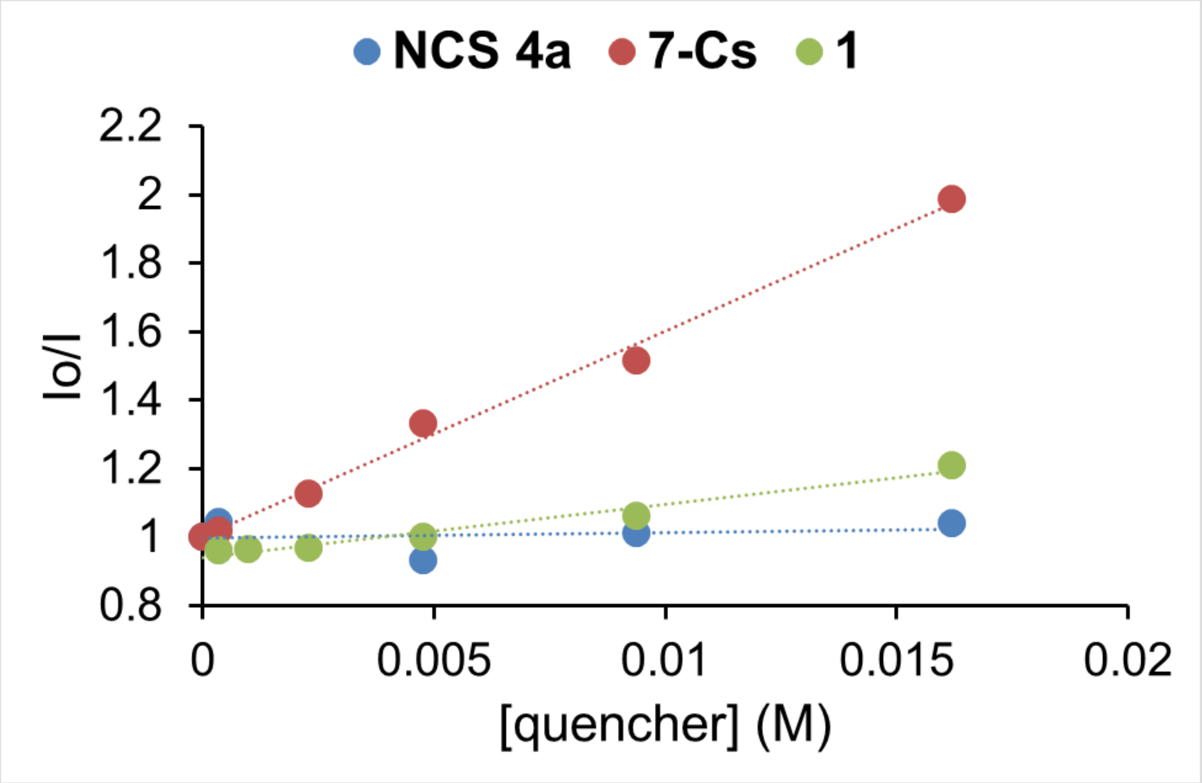

Scheme S2. Stern-Volmer plot for the quench of excited Ir[dF(CF_3)ppy]$_2$(dtbpy))PF_6 by **1**, **7-Cs** and NCS.

5 Pictures of Reaction Set-Up

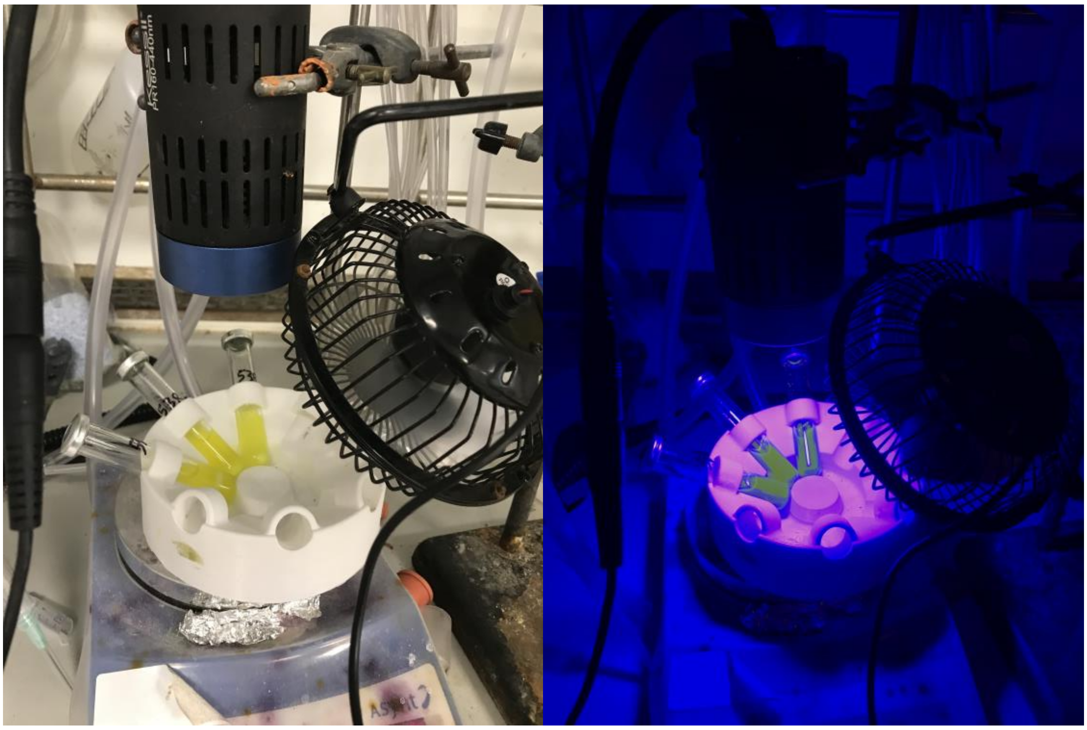

Figure S1.

6 Reaction Products

GP5 – General Procedure for Radical Strain-Release Amino-Functionalization

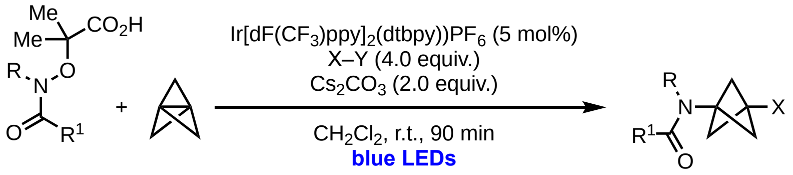

A dry tube equipped with a stirring bar was charged with the starting material (0.1 mmol, 1.0 equiv.), Ir[dF(CF_3)ppy_2](dtbpy)](PF_6) (5 µmol, 5 mol%), Cs_2CO_3 (0.12 mmol, 1.2 equiv.) and the SOMOphile (0.4 mmol, 4.0 equiv.). The reaction vessel was sealed, evacuated and back-filled with N_2 (x 3). The degassed CH_2Cl_2 (0.05 M) was added and the mixture was stirred for 10 min. The propellane stock solution (0.5 M in benzene–CH_2Cl_2 (3:1), 0.2 mmol, 2.0 equiv.) was added, the blue LEDs were switched on and the reaction was stirred under irradiation for 2 hours. The mixture was diluted with H_2O (1 mL) and CH_2Cl_2 (1 mL) and the layers were separated. The aqueous layer was extracted with CH_2Cl_2 (x 2). The combined organic layers were dried ($MgSO_4$), filtered and evaporated. The crude was purified by column chromatography on silica gel.

N-(3-Chlorobicyclo[1.1.1]pentan-1-yl)-*N*-methylbenzamide (9)

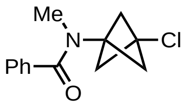

Following **GP5**, **9** (21 mg, 90%) was obtained as white solid R_f 0.33 [petrol-Et_2O (3:1)]; FT-IR ν_{max} (film)/cm^{-1} 2978, 2920, 2359, 1640, 1380, 1201, 758 ^{1}H NMR (500 MHz, $CDCl_3$) δ 7.43–7.34 (5H, m), 2.96 (3H, s), 2.29 (6H, br s); ^{13}C NMR (126 MHz, $CDCl_3$) δ 172.1, 136.9, 130.0, 128.4, 127.1, 58.7, 50.6, 47.0, 34.2; HRMS (ESI) Found MH^+ 236.0831, $C_{13}H_{15}ONCl$ requires 236.0837

N-(3-Bromobicyclo[1.1.1]pentan-1-yl)-*N*-methylbenzamide (10)

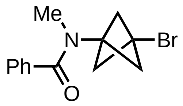

Following **GP5**, **10** (20%) was obtained as an oil. FT-IR ν_{max} (film)/cm^{-1} 3006, 2359, 1646, 1275, 750, 669; ^{1}H NMR (400 MHz, $CDCl_3$) δ 7.43–7.35 (5H, m), 2.97 (3H, s), 2.36 (6H, br s); ^{13}C NMR (101 MHz, $CDCl_3$) δ 172.1, 137.0, 130.1, 128. 5, 127.2, 59.9, 53.2, 34.0; HRMS (ESI) Found MNa^+ 302.0141, $C_{13}H_{14}ONBrNa$ requires 302.0151.

***N*-Methyl-*N*-(3-(phenylthio)bicyclo[1.1.1]pentan-1-yl)benzamide (11)**

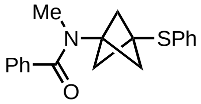

Following **GP5**, **11** (24 mg, 77%) was obtained as oil. R$_f$ 0.42 [petrol-EtOAc (4:1)]; FT-IR ν_{max} (film)/cm^{-1} 3002, 2987, 2401, 1656, 1298, 1212, 648; ^{1}H NMR (500 MHz, $CDCl_3$) δ 7.41–7.29 (10H, m), 2.94 (3H, s), 2.02 (6H, br s); ^{13}C NMR (126 MHz, $CDCl_3$) δ 172.1, 133.8, 133.4, 129.8, 129.1, 128.3, 128.1, 127.2, 124.9, 56.3, 52.9, 39.6; 32.2; HRMS (ESI) Found MNa$^+$ 332.1072, $C_{19}H_{19}ONSNa$ requires 332.1080.

***N*-Methyl-*N*-(3-((trifluoromethyl)thio)bicyclo[1.1.1]pentan-1-yl)benzamide (12)**

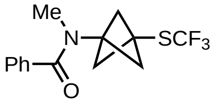

Following **GP5**, **12** (20 mg, 66%) was obtained as oil. R$_f$ 0.22 [toluene-EtOAc (8:1)]; FT-IR ν_{max} (film)/cm^{-1} 2921, 2359, 1644, 1381, 1227, 1112; ^{1}H NMR (500 MHz, $CDCl_3$) δ 7.45–7.36 (5H, m), 2.98 (3H, s), 2.28 (6H, br s); ^{13}C NMR (126 MHz, $CDCl_3$) δ 172.1, 136.7, 131.5, 130.3 (q, *J* = 307.3 Hz), 130.1, 128.5, 127.2, 57.7, 53.2, 35.1, 31.1; ^{19}F NMR (376 MHz, $CDCl_3$) δ –39.9; HRMS (ESI) Found MH$^+$ 302.0828, $C_{14}H_{15}ONF_3$ requires 302.0821

***N*-Methyl-*N*-(3-(phenylselanyl)bicyclo[1.1.1]pentan-1-yl)benzamide (13)**

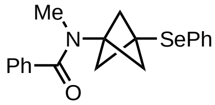

Following **GP5**, **13** (72%) was obtained as an oil. FT-IR ν_{max} (film)/cm^{-1} 2962, 2360, 1637, 1376, 1259, 1021, 792, 748, 692; ^{1}H NMR (400 MHz, $CDCl_3$) δ 7.55–7.44 (2H, m), 7.43–7.22 (8H, m), 2.93 (3H, s), 2.04 (6H, br s); ^{13}C NMR (101 MHz, $CDCl_3$) δ 172.0, 135.5, 129.8, 129.1, 128.3, 128.1, 127.2, 57.3, 54.2, 32.4; HRMS (ESI) Found MNa$^+$ 380.0513, $C_{19}H_{19}ONSeNa$ requires 380.0524.

***N*-Methyl-*N*-(3-(phenylsulfonyl)bicyclo[1.1.1]pentan-1-yl)benzamide (14)**

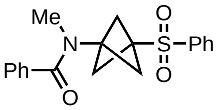

To a solution of **11** (38 mg, 0.12 mmol, 1 equiv) in CH_2Cl_2 (0.4 mL) at 0 ºC was added 3-chloroperoxybenzoic acid (84 mg, 0.48 mmol, 4.0 equiv.). The mixture was stirred 10 min at room temperature. The precipitate was filtered and the filtrate was washed with $Na_2S_2O_3$

solution and 1 M NaOH solution. The organic phase was dried (Na_2SO_4) and the solvent evaporated to give **14** (41 mg, quant.) as an oil. FT-IR ν_{max} (film)/cm^{-1} 2982, 2922, 1646, 1322, 1124, 851; ^{1}H NMR (500 MHz, $CDCl_3$) δ 7.83 (2H, d, *J* = 7.5 Hz), 7.66 (1H, t, *J* = 7.4 Hz), 7.56 (2H, t, *J* = 7.7 Hz), 7.46–7.28 (5H, m), 2.91 (3H, s), 2.27 (6H, br s); ^{13}C NMR (126 MHz, $CDCl_3$) δ 172.3, 137.0, 136.5, 134.0, 130.3, 129.4, 128.6, 128.5, 127.2, 53.3, 52.0, 49.6, 33.8; HRMS (ESI) Found MH$^+$ 342.1169, $C_{19}H_{20}O_3NS$ requires 342.1158.

N-Methyl-*N*-(3-(phenylsulfinyl)bicyclo[1.1.1]pentan-1-yl)benzamide (15)

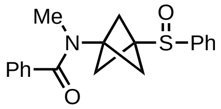

A solution of **11** (100 mg, 1.0 equiv.) in 2,2,2-trifluoroethanol (0.16 mL, 2 M) was cooled to 0 ºC and treated with H_2O_2 (56 µL, 30% in water, 1.7 equiv.). The reaction mixture was allowed to warm to room temperature overnight. Solid Na_2SO_3 was added to quench the reaction and the resulting mixture was stirred for 30 min. The resulting mixture was dried over $MgSO_4$, filtered and evaporated. Purified by column chromatography on silica gel eluting with CH_2Cl_2–MeOH, gave **15** (78%) as an oil. FT-IR ν_{max} (film)/cm^{-1} 2961, 2361, 2340, 1642, 1259, 660, 798, 621; ^{1}H NMR (500 MHz, $CDCl_3$) δ 7.54–7.46 (5H, m), 7.43–7.29 (5H, m), 2.90 (3H, s), 2.06 (6H, br s); ^{13}C NMR (126 MHz, $CDCl_3$) δ 172.2, 141.5, 136.8, 131.4, 130.1, 129.2, 128.5, 127.2, 124.2, 53.1, 51.6, 49.6, 33.7; HRMS (APCI) Found MH$^+$ 326.1209, $C_{19}H_{20}O_2NS$ requires 326.1209.

N-(Bicyclo[1.1.1]pentan-1-yl)-*N*-methylbenzamide (16)

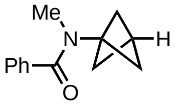

Following **GP5**, **16** (10 mg, 49%) was obtained as oil. R_f 0.28 [petrol-EtOAc (4:1)]; FT-IR ν_{max} (film)/cm^{-1} 2969, 2875, 1633, 1375, 1301, 1206. 699; ^{1}H NMR (500 MHz, $CDCl_3$) δ 7.39–7.34 (5H, m), 2.98 (3H, s), 2.30 (1H, s), 1.80 (6H, br s); ^{13}C NMR (126 MHz, $CDCl_3$) δ 172.1, 137.9, 129.5, 128.1, 127.2, 54.8, 52.8, 31.5, 23.6; HRMS (ESI) Found MH$^+$ 202.1234, $C_{13}H_{16}ON$ requires 202.1226.

N-(3-Chlorobicyclo[1.1.1]pentan-1-yl)-4-methoxy-N-methylbenzamide (17)

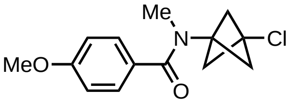

Following **GP5**, **17** (84%) was obtained as an oil. FT-IR ν_{max} (film)/cm^{-1} 2921, 2359, 1715, 1638, 1377, 1253, 1200, 1028, 842, 750; ^{1}H NMR (400 MHz, $CDCl_3$) δ 7.35 (2H, d, *J* = 8.7 Hz), 6.89 (2H, d, *J* = 8.7 Hz), 3.83 (3H, s), 2.97 (3H, s), 2.33 (6H, s); ^{13}C NMR (101 MHz, $CDCl_3$) δ 172.2, 161.1, 129.3, 129.0, 113.7, 58.7, 55.5, 50.9, 47.2, 34.5 (bs); HRMS (ESI) Found MNa$^+$ 288.0752, $C_{14}H_{16}O_2NClNa$ requires 288.0762.

N-(3-Chlorobicyclo[1.1.1]pentan-1-yl)-N-methyl-4-(trifluoromethyl)benzamide (18)

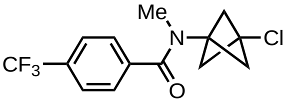

Following **GP5**, **18** (24 mg, 78%) was obtained as oil. R_f 0.27 [petrol-Et_2O (3:1)]; FT-IR ν_{max} (film)/cm^{-1} 2982, 2922, 1646, 1322, 1124, 851; ^{1}H NMR (500 MHz, $CDCl_3$) δ 7.67 (2H, d, *J* = 7.9 Hz), 7.49 (2H, d, *J* = 7.9 Hz), 2.97 (3H, s), 2.33 (6H, br s); ^{13}C NMR (126 MHz, $CDCl_3$) δ 170.6, 140.4, 132.0 (q, *J* = 32.8 Hz), 127.5, 125.6 (q, *J* = 3.8 Hz), 123.8 (q, *J* = 272.4 Hz), 58.8, 50.5, 47.0, 35.9; ^{19}F NMR (376 MHz, $CDCl_3$) δ –62.8. HRMS (ESI) Found MNa$^+$ 304.0712, $C_{14}H_{14}NOF_3Cl$ requires 304.0711

6-Chloro-N-(3-chlorobicyclo[1.1.1]pentan-1-yl)-N-methylnicotinamide (19)

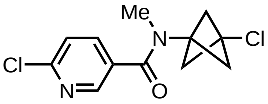

Following **GP5**, **19** (64%) was obtained as an oil. FT-IR ν_{max} (film)/cm^{-1} 2961, 2359, 1641, 1394, 1258, 1018, 794, 765, 668; ^{1}H NMR ($CDCl_3$, 500 MHz) δ 8.43 (1H, s), 7.70 (1H, d, *J* = 7.7 Hz), 7.40 (1H, d, *J* = 8.2 Hz), 2.99 (3H, s), 2.38 (6H, br s); ^{13}C NMR (101 MHz, $CDCl_3$) δ 168.4, 153.0, 148.2, 137.8, 131.2 (br s), 124.4, 58.8, 50.6, 47.0, 34.1 (br s); HRMS (APCI) Found MH$^+$ 271.0399, $C_{12}H_{13}ON_2Cl_2$ requires 271.0399.

N-(3-Chlorobicyclo[1.1.1]pentan-1-yl)-N-methylfuran-2-carboxamide (20)

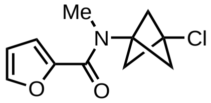

Following **GP5**, **20** (59%) was obtained as an oil. FT-IR ν_{max} (film)/cm^{-1} 2360, 1635, 1486, 1392, 1201, 1063, 985, 914, 800, 750; ^{1}H NMR (400 MHz, $CDCl_3$) δ 7.49 (1H, dd, *J* = 1.8,

0.9 Hz), 6.99 (1H, dd, J = 3.5, 0.9 Hz), 6.48 (1H, dd, J = 3.5, 1.8 Hz), 3.16 (3H, s), 2.51 (6H, s); ^{13}C NMR (101 MHz, $CDCl_3$) δ 161.0, 148.1, 144.0, 116.6, 111.6, 58.5, 51.1, 47.1, 34.2; HRMS (ESI) Found MNa^+ 248.0439, $C_{11}H_{12}O_2NClNa$ requires 248.0449.

***N*-(3-Chlorobicyclo[1.1.1]pentan-1-yl)-*N*-methylcyclobutanecarboxamide (21)**

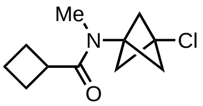

Following **GP5**, **21** (56%) was obtained as an oil. FT-IR ν_{max} (film)/cm^{-1} 2923, 2359, 1653, 1394, 1275, 750, 668; ^{1}H NMR ($CDCl_3$, 500 MHz) δ 3.30–3.15 (1H, m), 2.82 (3H, br s), 2.48 (6H, s), 2.37–2.26 (2H, m), 2.19–2.07 (2H, m), 1.98–1.91 (1H, m), 1.90–1.79 (1H, m); ^{13}C NMR (126 MHz, $CDCl_3$) δ 175.5, 58.6, 50.4 (br s), 47.0 (br s), 38.3, 32.9 (br s), 24.9, 17.9; HRMS (APCI) Found MH^+ 214.0990, $C_{11}H_{17}ONCl$ requires 214.0993.

***N*-(3-Chlorobicyclo[1.1.1]pentan-1-yl)-*N*-cyclohexylbenzamide (22)**

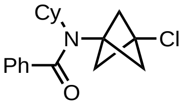

Following **GP5**, **22** (23 mg, 74%) was obtained as oil. R_f 0.50[toluene-EtOAc (8:1)]; FT-IR ν_{max} (film)/cm^{-1}2996, 2341, 2366, 1289, 1206, 712; ^{1}H NMR (500 MHz, $CDCl_3$) δ 7.43–7.32 (5H, m), 3.62 (1H, t, J = 10.9 Hz), 2.42 (6H, s), 1.88–1.77 (9H, m), 1.61–1.58 (1H, m), 1.18–1.05 (3H, m); ^{13}C NMR (126 MHz, $CDCl_3$) δ 173.0, 138.2, 129.8, 128.5, 126.7, 60.4, 59.8, 49.6, 47.3, 31.6, 26.5, 25.5; HRMS (ESI) Found MNa^+304.1471., $C_{18}H_{23}NOCl$ requires 304.1468.

***N*-(*tert*-Butyl)-*N*-(3-chlorobicyclo[1.1.1]pentan-1-yl)benzamide (23)**

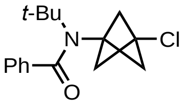

Following **GP5**, **23** (86%) was obtained as an oil. FT-IR ν_{max} (film)/cm^{-1} 2988, 2360, 2341, 1275, 1260, 764, 750; ^{1}H NMR ($CDCl_3$, 500 MHz) δ 7.52–7.44 (3H, m), 7.38 (2H, t, J = 7.4 Hz), 2.17 (6H, s), 1.51 (9H, s); ^{13}C NMR (126 MHz, $CDCl_3$) δ 175.7, 140.3, 131.2, 129.0, 128.4, 62.0, 58.8, 50.7, 47.5, 29.4; HRMS (APCI) Found MH^+ 278.1301, $C_{16}H_{21}ONCl$ requires 278.1306.

Benzyl (3-Chlorobicyclo[1.1.1]pentan-1-yl)(methyl)carbamate (25)

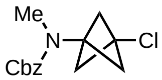

Following **GP5**, **25** (48%) was obtained as an oil. FT-IR ν_{max} (film)/cm^{-1} 2961, 2359, 1703, 1395, 1260, 1204, 1027, 907, 799, 731; ^{1}H NMR ($CDCl_3$, 500 MHz) δ 7.39–7.31 (5H, m), 5.11 (2H, s), 2.88 (3H, s), 2.39 (6H, s); ^{13}C NMR (126 MHz, $CDCl_3$) δ 155.9 (br s), 136.5, 128.7, 128.32, 128.26, 67.5 (br s), 58.8, 49.8 (br s), 46.9, 33.0 (br s); HRMS (APCI) Found MH$^+$ 266.0941, $C_{14}H_{17}O_2NCl$ requires 266.0942.

***tert*-Butyl (3-Chlorobicyclo[1.1.1]pentan-1-yl)(methyl)carbamate (26)**

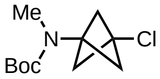

Following **GP5**, **26** (65%) was obtained as an oil. FT-IR ν_{max} (film)/cm^{-1} 2979, 2359, 1700, 1366, 1260, 1158, 1023, 750; ^{1}H NMR ($CDCl_3$, 500 MHz) δ 2.80 (3H, s), 2.38 (6H, s), 1.46 (9H, s); ^{13}C NMR (101 MHz, $CDCl_3$) δ 155.2, 80.5, 58.8, 49.8, 47.0, 32.6, 28.6; HRMS (ESI) Found MNa$^+$ 254.0913, $C_{11}H_{18}O_2NClNa$ requires 254.0918.

***N*-(3-Chlorobicyclo[1.1.1]pentan-1-yl)-*N*,4-dimethylbenzenesulfonamide (27)**

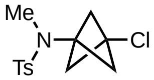

Following **GP5**, **27** (73%) was obtained as an oil. R_f 0.40 [toluene-EtOAc (8:1)]; FT-IR ν_{max} (film)/cm^{-1} 2978, 2334, 1733, 1242, 1134, 756, ^{1}H NMR (500 MHz, $CDCl_3$) δ 7.67 (2H, d, *J* = 7.9 Hz), 7.32 (2H, d, *J* = 7.9 Hz), 2.83 (3H, s), 2.44 (3H, s), 2.26 (6H, s); ^{13}C NMR (126 MHz, $CDCl_3$) δ 144.0, 135.8, 129.9, 127.6, 58.1, 49.9, 46.5, 34.3, 21.7; HRMS (ESI) Found MNa$^+$ 308.0494, $C_{13}H_{16}O_2SNClNa$ requires 308.0488.

***tert*-Butyl Benzyl(3-chlorobicyclo[1.1.1]pentan-1-yl)carbamate (28)**

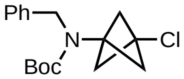

Following **GP5**, **28** (66%) was obtained as an oil. FT-IR ν_{max} (film)/cm^{-1} 2962, 2359, 1703, 1391, 1259, 1084, 1017, 794, 750; ^{1}H NMR ($CDCl_3$, 400 MHz) δ 7.31 (2H, t, *J* = 7.3 Hz), 7.25–7.20 (1H, m), 7.16 (2H, d, *J* = 7.3 Hz), 4.44 (2H, s), 2.31 (6H, s), 1.48 (9H, s); ^{13}C NMR (101 MHz, $CDCl_3$) δ 155.4 (br s), 139.0, 128.6, 127.2, 126.7, 80.8, 59.2, 50.1, 50.0, 47.1, 28.6; HRMS (ESI) Found MNa$^+$ 330.1219, $C_{17}H_{22}O_2NClNa$ requires 330.1231.

tert-Butyl Benzoyl(3-chlorobicyclo[1.1.1]pentan-1-yl)carbamate (29)

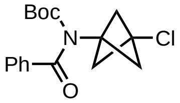

Following **GP5**, **29** (53%) was obtained as an oil. FT-IR ν_{max} (film)/cm^{-1} 2979, 2359, 1737, 1688, 1342, 1261, 1202, 1152, 750, 668; ^{1}H NMR (400 MHz, $CDCl_3$) δ 7.62–7.57 (2H, m), 7.51 (1H, t, *J* =7.5 Hz), 7.41 (2H, t, *J* = 7.7 Hz), 2.63 (6H, s), 1.16 (9H, s); ^{13}C NMR (101 MHz, $CDCl_3$) δ 172.9, 152.5, 137.1, 132.2, 128.5, 128.2, 83.6, 59.8, 48.1, 46.9, 27.6; HRMS (ESI) Found MNa$^+$ 344.1030, $C_{17}H_{20}O_3NClNa$ requires 344.1024.

Benzyl (3-Chlorobicyclo[1.1.1]pentan-1-yl)(propionyl)carbamate (30)

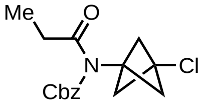

Following **GP5**, **30** (64%) was obtained as an oil. FT-IR ν_{max} (film)/cm^{-1} 2963, 2359, 1736, 1276, 1202, 1014, 977, 908, 750; ^{1}H NMR ($CDCl_3$, 400 MHz) δ 7.44–7.34 (5H, m), 5.21 (2H, s), 2.74 (2H, q, *J* = 7.3 Hz), 2.54 (6H, s), 1.09 (3H, t, *J* = 7.3 Hz); ^{13}C NMR (101 MHz, $CDCl_3$) δ 177.1, 154.2, 134.6, 129.1, 129.0, 128.9, 69.2, 60.2, 48.0, 46.7, 32.1, 9.4; HRMS (ESI) Found MNa$^+$ 330.0853, $C_{16}H_{18}O_3NClNa$ requires 330.0867.

Benzyl (3-Chlorobicyclo[1.1.1]pentan-1-yl)(methoxycarbonyl)carbamate (31)

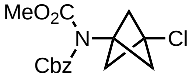

Following **GP5**, **31** (69%) was obtained as an oil. FT-IR ν_{max} (film)/cm^{-1} 3005, 2359, 2342, 1275, 1260, 750; ^{1}H NMR (400 MHz, $CDCl_3$) δ 7.43–7.31 (5H, m), 5.22 (2H, s), 3.80 (3H, s), 2.51 (6H, s); ^{13}C NMR (101 MHz, $CDCl_3$) δ 153.9, 153.2, 135.1, 128.93, 128.90, 128.6, 69.2, 60.0, 54.1, 48.1, 46.6; HRMS (ESI) Found MNa$^+$ 332.0650, $C_{15}H_{16}O_4NClNa$ requires 332.0660.

tert-Butyl (_tert_-Butoxycarbonyl)(3-chlorobicyclo[1.1.1]pentan-1-yl)carbamate (32)

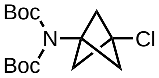

Following **GP5**, **32** (45%) was obtained as an oil. FT-IR ν_{max} (film)/cm^{-1} 2923, 2359, 1714, 1275, 1129, 750, 669; ^{1}H NMR (400 MHz, $CDCl_3$) δ 2.50 (6H, s), 1.49 (18H, s); ^{13}C NMR (101 MHz, $CDCl_3$) δ 152.0, 83.1, 59.8, 47.5, 46.8, 28.1; HRMS (ESI) Found MNa$^+$ 340.1268, $C_{15}H_{24}O_4NClNa$ requires 340.1286.

_N_-(Bicyclo[1.1.1]pentan-1-yl)-_N_-methyl-[1,1'-biphenyl]-4-carboxamide (35)

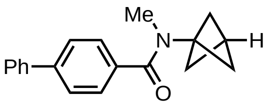

Following **GP5**, **35** (11mg, 38%) was obtained as a white solid. R_f 0.45 [toluene-EtOAc (8:1)]; FT-IR ν_{max} (film)/cm^{-1} 2978, 2865, 1621, 1389, 1312, 1245. 659; ^{1}H NMR (500 MHz, $CDCl_3$) δ 7.61 (4H, d, *J* = 8.1 Hz), 7.47–7.44 (4H, m), 7.37 (1H, t, *J* = 7.4 Hz), 3.02 (3H, s), 2.34 (1H, br s), 1.86 (6H, br s); ^{13}C NMR (126 MHz, $CDCl_3$) δ 172.0, 142.4, 140.5, 137.0, 136.7, 129.0, 127.8, 127.3, 126.8, 54.9, 52.8, 31.3 23.7; HRMS (ESI) Found MH^+ 278.1548, $C_{19}H_{20}NO$ requires 278.1545.

Single crystal has been grown via slow evaporation from a solution [CH_3CN–$CHCl_3$ (10:1)]

_N_-(3-Chlorobicyclo[1.1.1]pentan-1-yl)-_N_-methyl-[1,1'-biphenyl]-4-carboxamide (36)

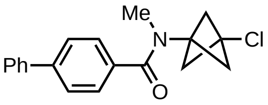

Following **GP5**, **36** (26 mg, 84%) was obtained as a white solid. R_f 0.43 [petrol-EtOAc (4:1)]; FT-IR ν_{max} (film)/cm^{-1} 2989, 2934, 2365, 1678, 1333, 1212, 712; ^{1}H NMR (500 MHz, $CDCl_3$) δ 7.67–7.56 (4H, m), 7.52–7.35 (5H, m), 3.02 (3H, s), 2.36 (6H, s); ^{13}C NMR (126 MHz, $CDCl_3$) δ 172.0, 143.0, 140.2, 135.7, 129.0, 128.0, 127.8, 127.3, 127.1, 58.8, 50.8, 47.1, 34.4; HRMS (ESI) Found MNa^+ 334.0971, $C_{19}H_{18}ONClNa$ requires 334.0975.

Single crystal has been grown via slow evaporation from a solution [CH_3CN–$CHCl_3$ (10:1)].

_N_-Methyl-_N_-(3-(phenylthio)bicyclo[1.1.1]pentan-1-yl)-[1,1'-biphenyl]-4-carboxamide (37)

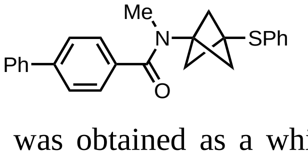

Following **GP5**, **37** (24 mg, 61%) was obtained as a white solid. R_f 0.44 [toluene-EtOAc (1:8)]; FT-IR ν_{max} (film)/cm^{-1} 2998, 2897, 2378, 1690, 1310, 1198, 670; ^{1}H NMR (500 MHz, $CDCl_3$) δ 7.60 (4H, d, *J* = 7.5 Hz), 7.54–7.35 (7H, m), 7.30–7.28 (3H, m), 2.97 (3H, s), 2.10 (6H, br s); ^{13}C NMR (126 MHz, $CDCl_3$) δ 171.9, 142.7, 140.4, 135.9, 133.9, 129.1, 129.0, 128.2, 128.1, 127.9, 127.8, 127.3, 127.0, 56.3, 53.0, 39.6, 34.0; HRMS (ESI) Found MNa^+ 408.1382, $C_{25}H_{23}NOSNa$ requires 408.1393.

Single crystal has been grown via slow evaporation from a solution [CH_3CN–$CHCl_3$ (10:1)].

N-Methyl-N-(3-((trifluoromethyl)thio)bicyclo[1.1.1]pentan-1-yl)-[1,1'-biphenyl]-4-carboxamide (38)

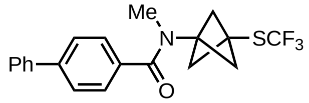

Following **GP5**, **38** (25 mg, 66%) was obtained a white solid. R_f 0.22 [toluene-EtOAc (8:1]; FT-IR ν_{max} (film)/cm^{-1} 3001, 2956, 2359, 1681, 1327, 1213, 1108; ^{1}H NMR (500 MHz, $CDCl_3$) δ 7.67–7.58 (4H, m), 7.53–7.44 (4H, m), 7.41–7.35 (1H, m), 3.02 (3H, s), 2.35 (6H, br s); ^{13}C NMR (126 MHz, $CDCl_3$) δ 172.0, 143.0, 140.2, 135.6, 131.5, 129.0, 127.8, 127.3, 127.1, 57.7, 53.2, 35.1, 35.5;(SCF_3 not visible) ^{19}F NMR (376 MHz, $CDCl_3$) δ –39.9; HRMS (ESI) Found MNa^+ 400.0951, $C_{20}H_{18}F_3NOSNa$ requires 400.0959.

Single crystal has been grown via slow evaporation from a solution [CH_3CN–CH_2Cl_2 (10:1)].

N-Methyl-N-(3-(phenylselanyl)bicyclo[1.1.1]pentan-1-yl)-[1,1'-biphenyl]-4-carboxamide (39)

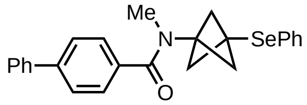

Following **GP5**, **39** (25 mg, 58%) was obtained a white solid. R_f 0.46 [toluene-EtOAc (8:1)]; FT-IR ν_{max} (film)/cm^{-1} 2989, 2375, 1689, 1401, 1296, 1021, 748, 692; ^{1}H NMR (500 MHz, $CDCl_3$) δ 7.62–7.57 (4H, m), 7.53–7.48 (2H, m), 7.46 (2H, t, J = 7.6 Hz), 7.42–7.37 (3H, m), 7.33–7.23 (3H, m), 2.96 (3H, s), 2.09 (6H, s); ^{13}C NMR (126 MHz, $CDCl_3$) δ 171.8, 142.7, 140.3, 136.1, 135.5, 129.1, 129.0, 128.3, 128.1, 127.9, 127.8, 127.3, 127.0, 57.3, 54.2, 32.4, 31.6; HRMS (ESI) Found MNa^+ 456.0835, $C_{25}H_{23}NOSeNa$ requires 456.0843.

Single crystal has been grown via slow evaporation from a solution [CH_3CN–Et_2O (10:1)].

N-Methyl-N-(3-(phenylsulfinyl)bicyclo[1.1.1]pentan-1-yl)-[1,1'-biphenyl]-4-carboxamide (40)

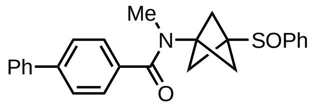

To a solution of **37** (46 mg, 0.12 mmol, 1 equiv) in CH_2Cl_2 (400 μL) at 0 C was added 3-chloroperoxybenzoic acid (21 mg, 0.12 mmol, 1.0 equiv.). The mixture was stirred 10 min at room temperature. The precipitate was filtered and the filtrate was washed with $Na_2S_2O_3$ solution and 1 M NaOH solution. The organic phase was dried (Na_2SO_4) and the solvent evaporated to obtain **40** (50 mg, quant.) as a white solid. R_f 0.43 [toluene-EtOAc (2:1)]; FT-

IR ν_{max} (film)/cm^{-1} 2930, 2897, 2375, 1680, 1229, 789, 621; ^{1}H NMR (500 MHz, $CDCl_3$) δ 7.62–7.55 (4H, m), 7.50 (5H, br s), 7.46 (2H, t, *J* = 7.6 Hz), 7.41–7.36 (3H, m), 2.94 (3H, s), 2.11 (6H, br s); ^{13}C NMR (126 MHz, $CDCl_3$) δ 172.0, 143.0, 141.6, 140.2, 135.5, 131.3, 129.2, 129.0, 128.0, 127.8, 127.3, 127.1, 124.2, 53.1, 51.6, 49.6, 33.8; HRMS (ESI) Found MH^+ 402.1516, $C_{25}H_{24}$O2NS requires 402.1522.

Single crystal has been grown via slow evaporation from a solution [CH_3CN–$CHCl_3$ (10:1)].

_N_-Methyl-_N_-(3-(phenylsulfonyl)bicyclo[1.1.1]pentan-1-yl)-[1,1'-biphenyl]-4-carboxamide (41)

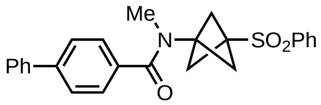

To a solution of **37** (46 mg, 0.12 mmol, 1.0 equiv.) in CH_2Cl_2 (400 μL) at 0 C was added 3-chloroperoxybenzoic acid (84 mg, 0.48 mmol, 4 equiv.). The mixture was stirred 10 min at room temperature. The precipitate was filtered and the filtrate was washed with $Na_2S_2O_3$ solution and 1 M NaOH solution. The organic phase was dried (Na_2SO_4) and the solvent evaporated to give **41** (50 mg, quant.) as a white solid; (FT-IR ν_{max} (film)/cm^{-1} 3006, 2897, 1646, 1379, 1303, 1171, 1143 720, 615; ^{1}H NMR (500 MHz, $CDCl_3$) δ 7.85 (2H, d, *J* = 7.7 Hz), 7.66 (1H, t, *J* = 7.4 Hz), 7.61–7.55 (6H, m), 7.48–7.37 (5H, m), 2.96 (3H, s), 2.33 (6H, br s); ^{13}C NMR (126 MHz, $CDCl_3$) δ 172.1, 143.3, 140.2, 137.1, 134.9, 134.0, 129.4, 129.1, 128.7, 128.1, 127.8, 127.3, 127.2, 53.3, 52.1, 49.7, 33.8; HRMS (ESI) Found MH^+ 418.1472, $C_{25}H_{24}NO_3S$ requires 418.1476.

Single crystal has been grown via slow evaporation from a solution [CH_3CN–$CHCl_3$ (10:1)].

We have evaluated the use of $CF_3C_6H_5$–CH_2Cl_2 instead of C_6H_6–CH_2Cl_2 due to its reduced safety concerns. However, in each case the radical strain-release amino-functionalization was less effective. As a result, we believe the C_6H_6–CH_2Cl_2 represents the optimum solvent for this transformation while $CF_3C_6H_5$–CH_2Cl_2 should only be used in the case of very electrophilic nitrogen-radicals that can directly react with C_6H_6.

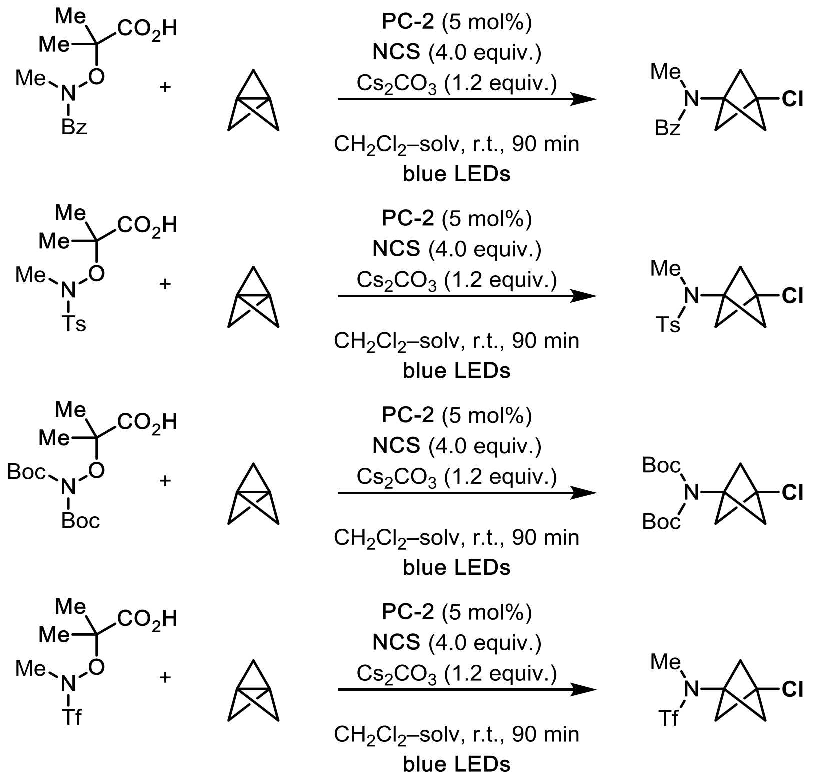

N-substituents	benzene	CF_3–C_6H_5
Me, Bz	90%	58%
Me, Ts	73%	38%
Boc, Boc	45%	25%
Me, Tf	–	27%

7 X-Ray Crystal Structures

Compound	Crystal Structure
**35**	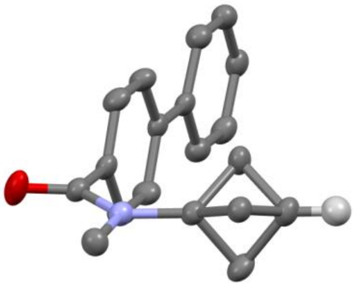
**36**	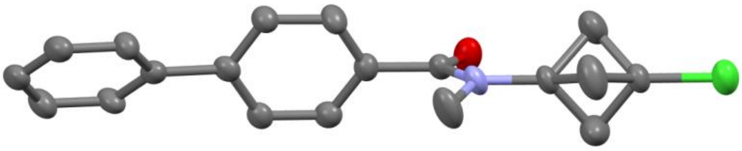
**37**	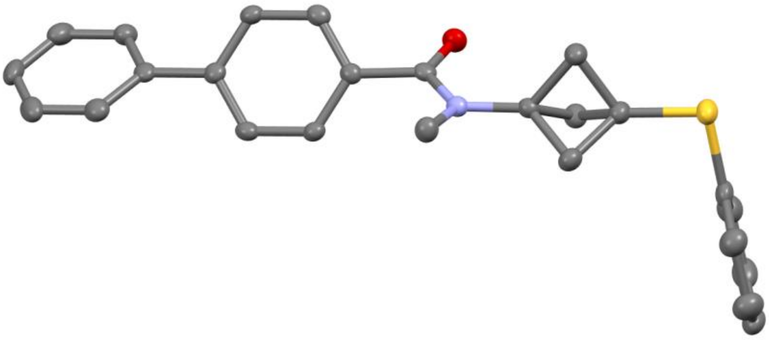
**38**	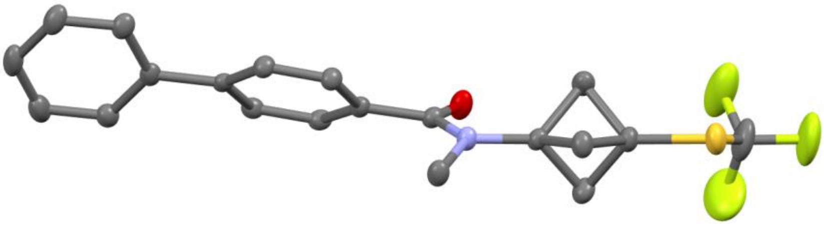
**39**	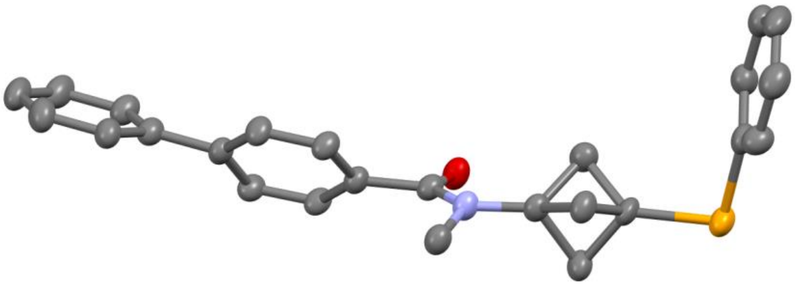

**40**	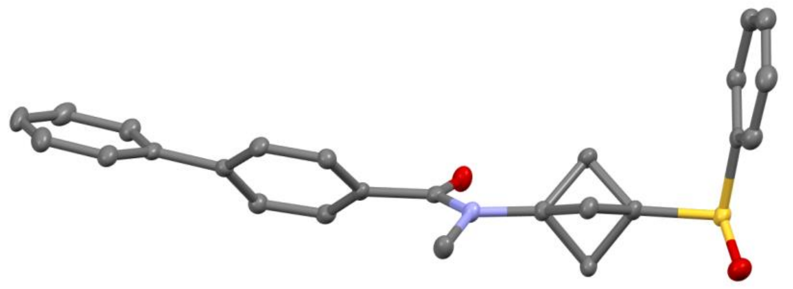
**41**	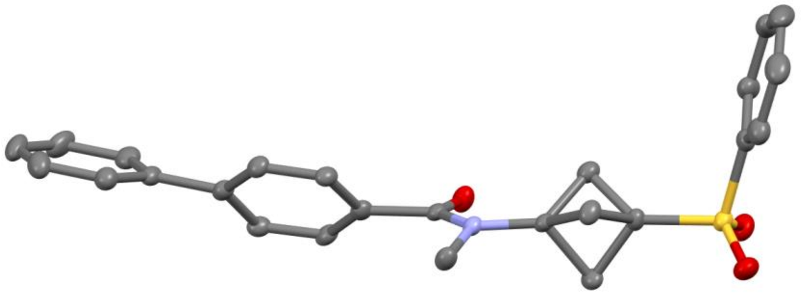

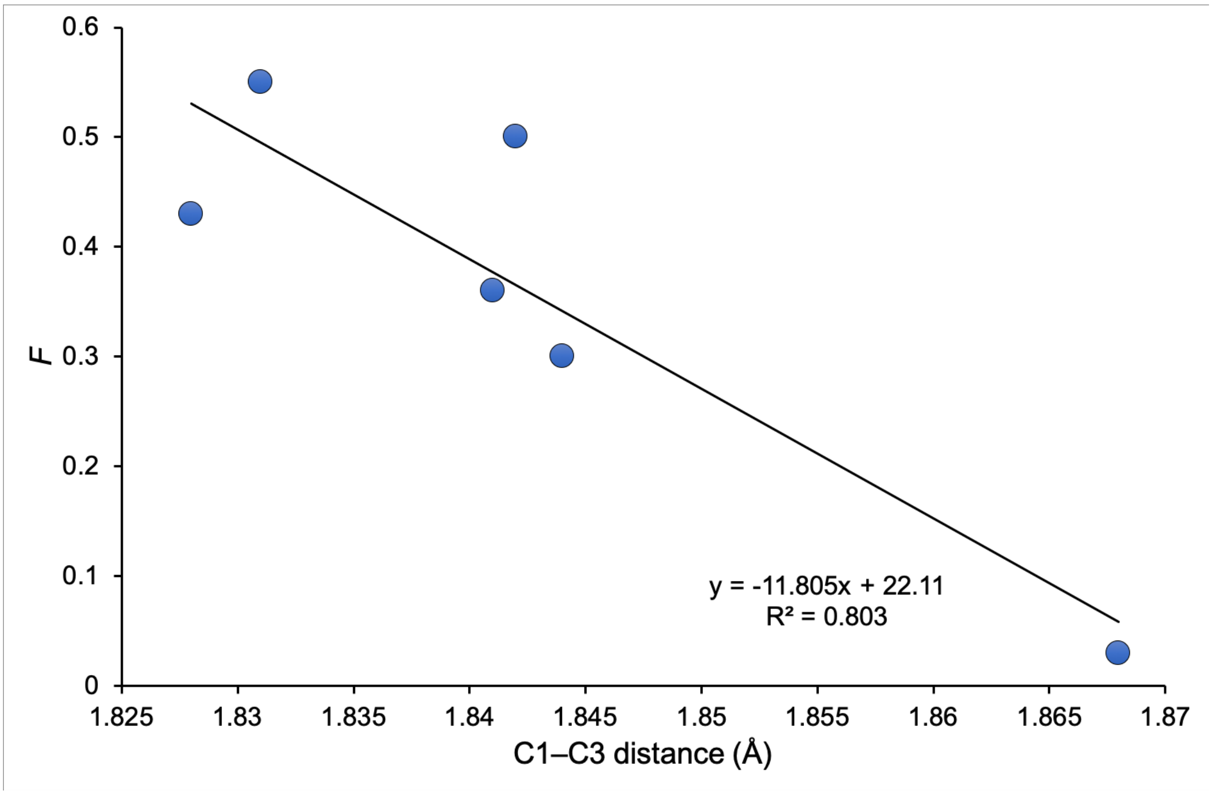

Scheme S3. Correlation between the C1–C3 non-bonded distances and the F parameter for the C3-substituents.

8 Correlations

8.1 Radical Strain-Release Step

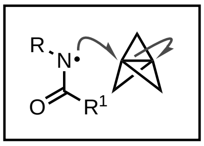

Table S1. Comparison of radical strain-release with radical addition to olefins.

	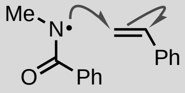	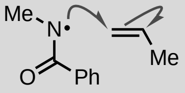	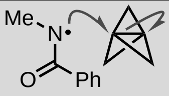
**ΔG° (Kcal mol^{-1})**	–17.8	–15.6	–12.4
**ΔG‡ (Kcal mol^{-1})**	18.9	16.9	15.3

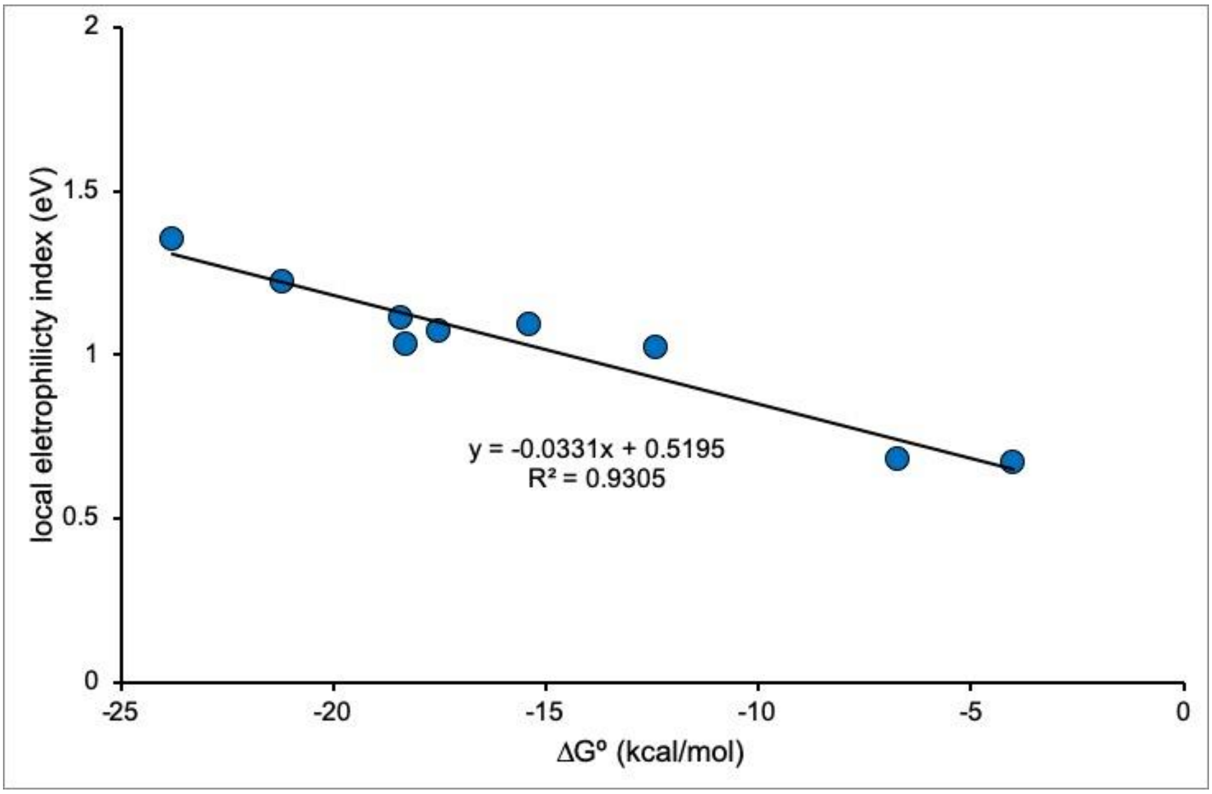

Scheme S4. Correlation between the nitrogen-radical electrophilicity and the strain-release free energy.

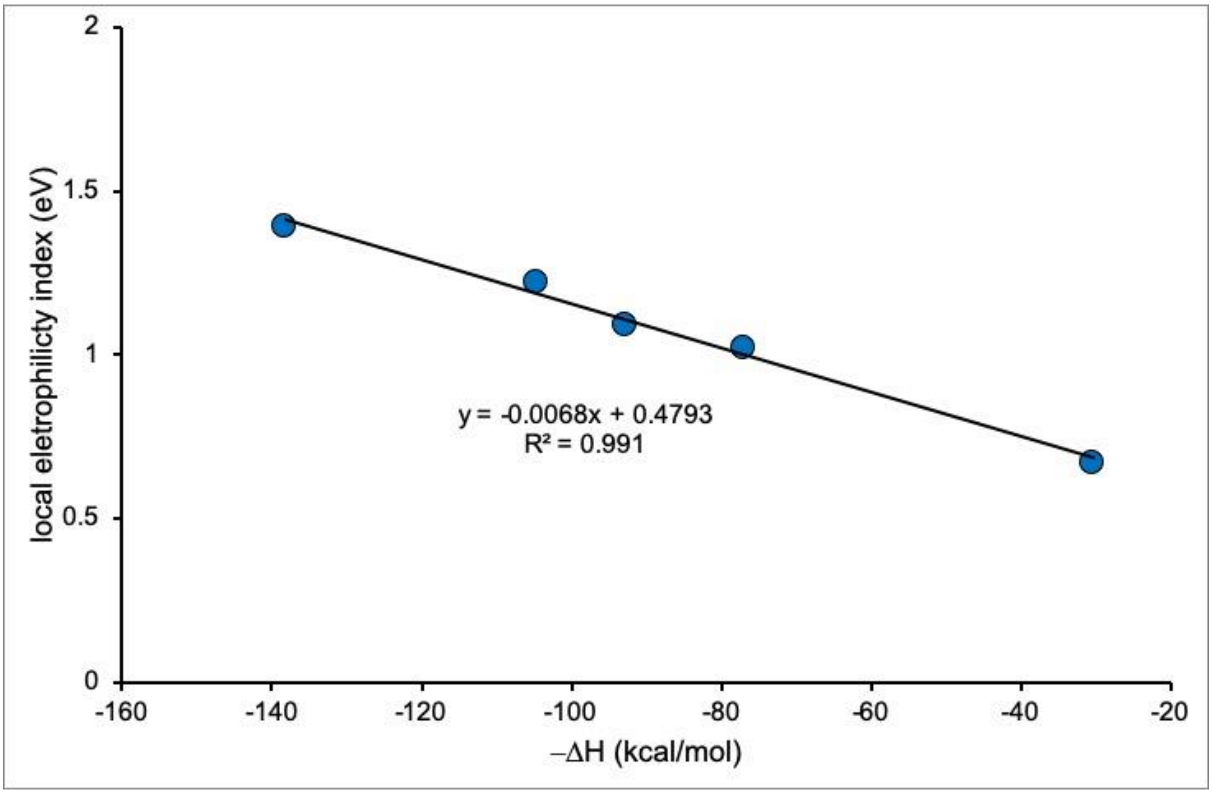

Scheme S5. Correlation between the strain-release enthalpy and the nitrogen radical electrophilicity.

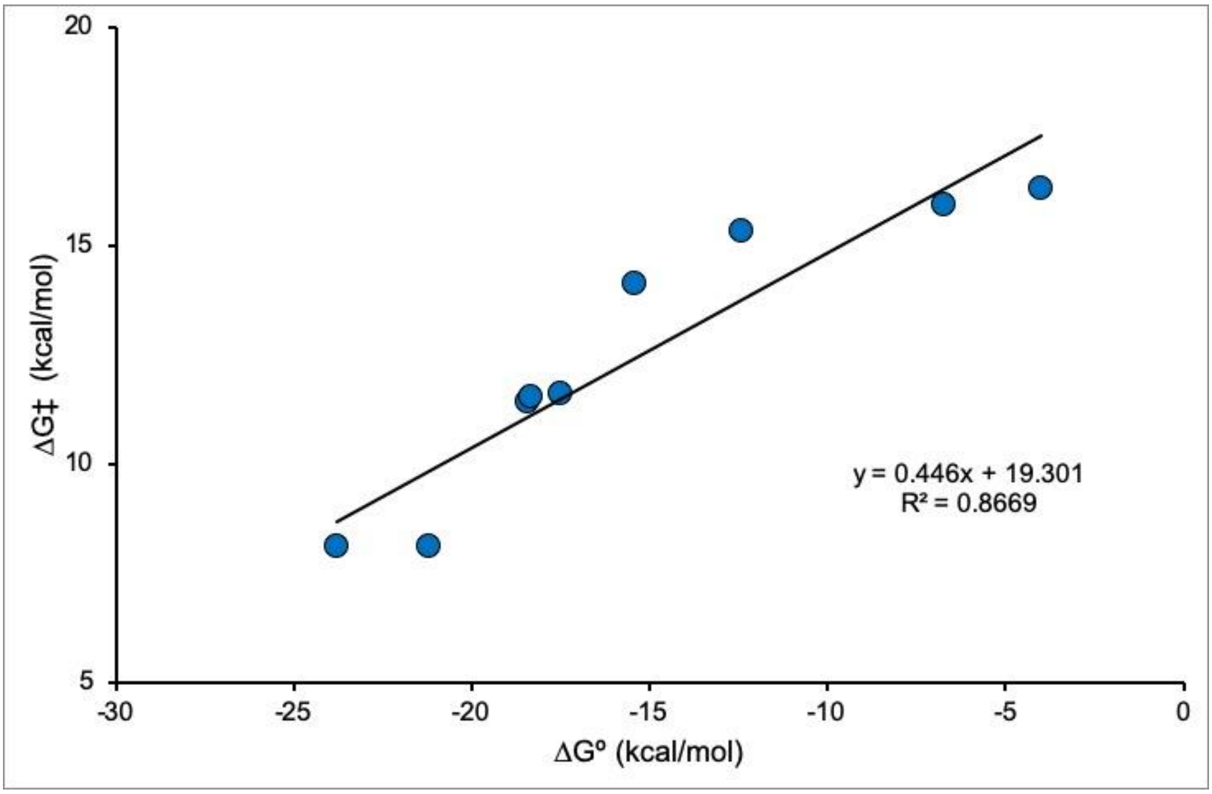

Scheme S6. Correlation between the strain-release free energy and barrier.

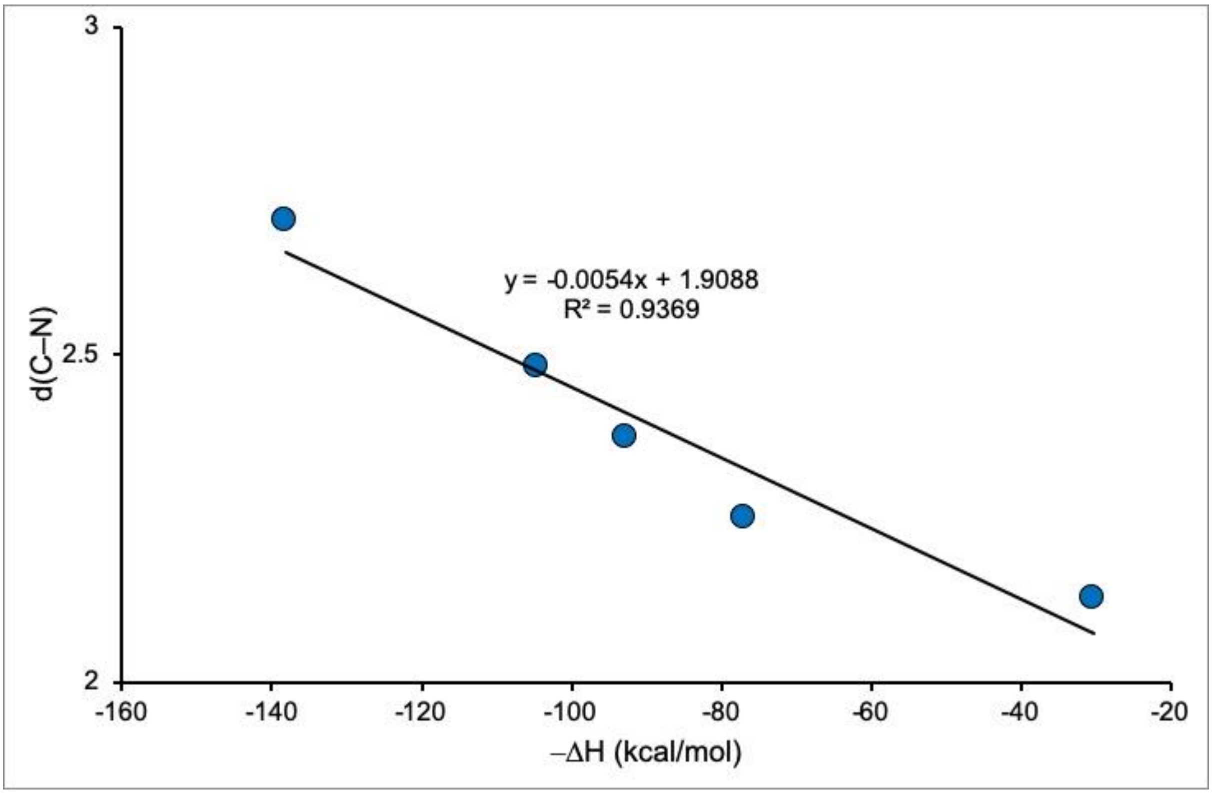

Scheme S7. Correlation between the strain-release enthalpy and the bond formation distance in the transition state.

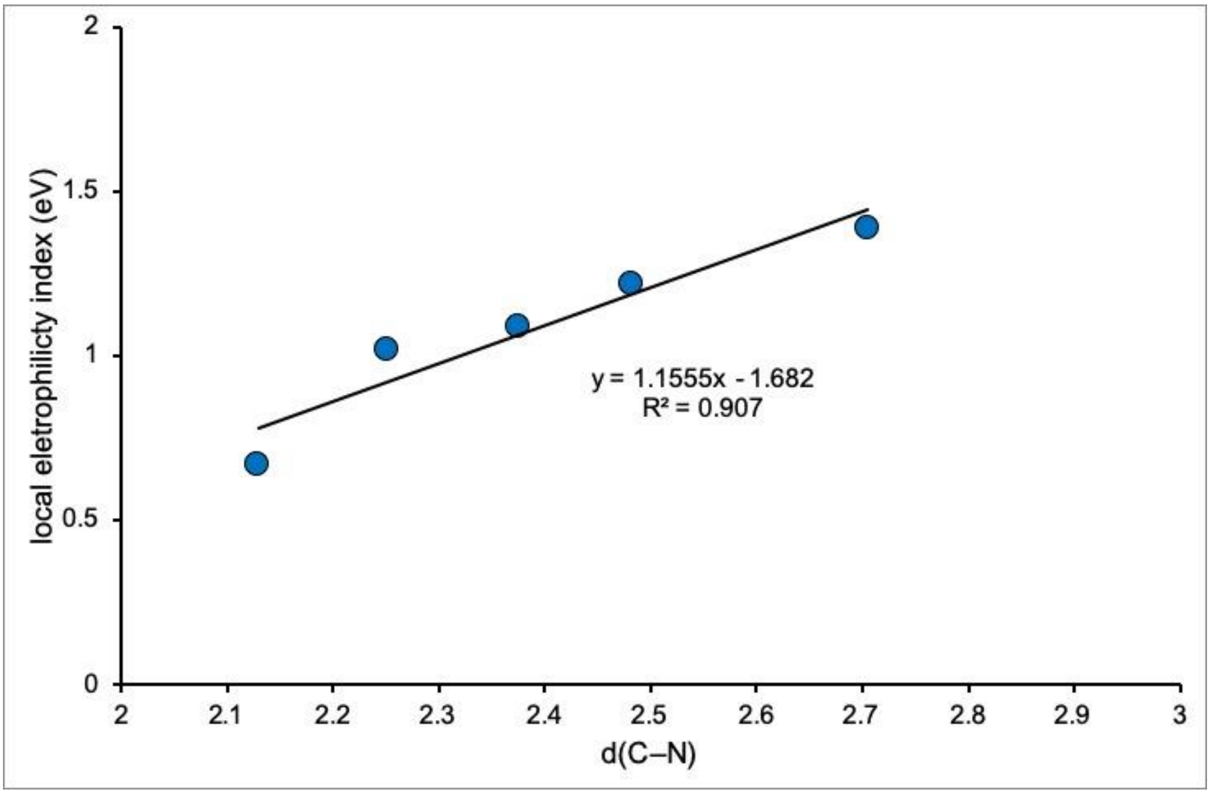

Scheme S8. Correlation between the nitrogen-radical electrophilicity and the bond formation distance in the transition state.

8.2 Atom/Group-Transfer Step

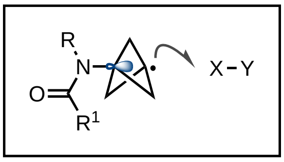

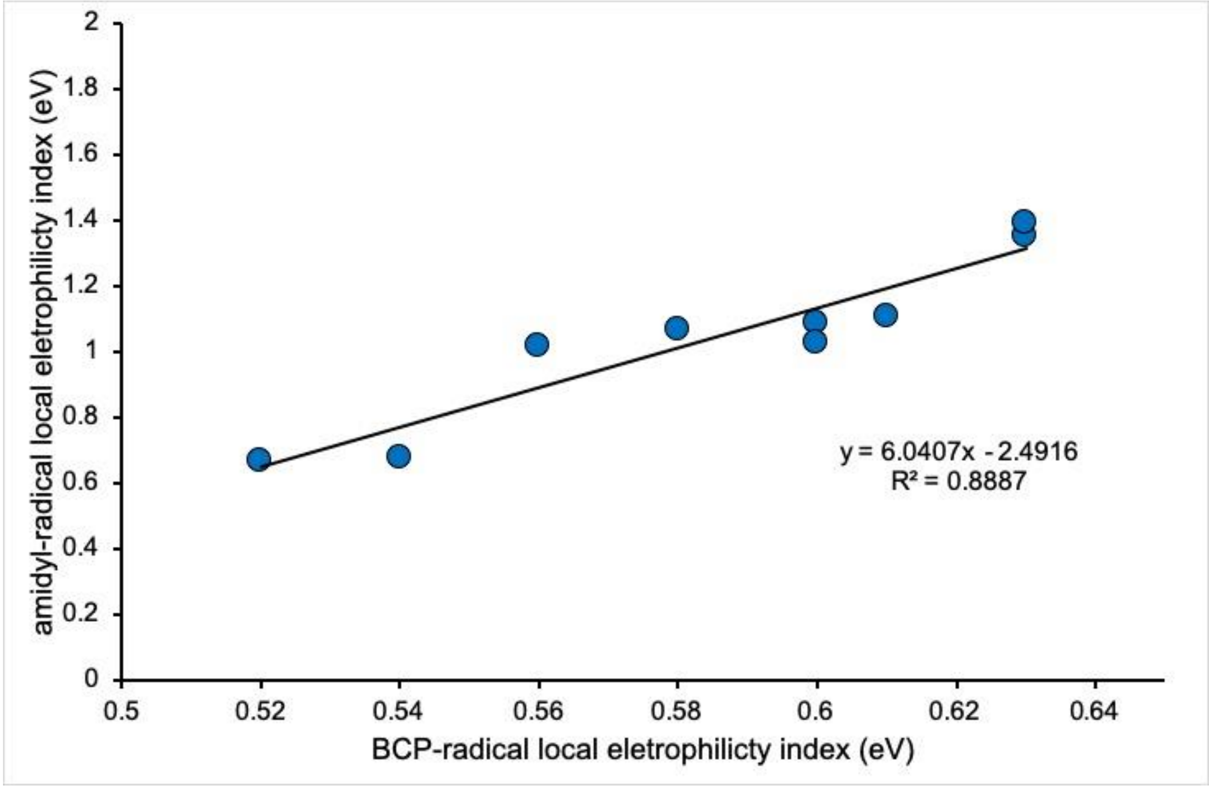

Scheme S9. Correlation between the nitrogen-radical electrophilicity and the 1-amide-BCP-radical electrophilicity.

Table S2. Reaction parameters for the chlorination.

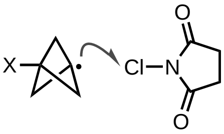

	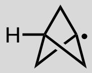	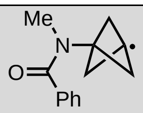
**electrophilicity index**	0.44	0.55
**ΔG° (Kcal mol^{-1})**	–20.4	–20.9
**ΔG‡ (Kcal mol^{-1})**	7.7	8.7

Table S3. Reaction parameters for the bromination and the H-abstraction.

	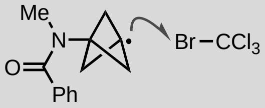	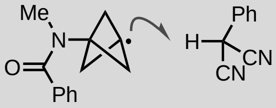
**ΔG° (Kcal mol^{-1})**	–30.2	–32.5
**ΔG‡ (Kcal mol^{-1})**	11.2	7.3

8.3 Side Reactions

Table S4. Kinetic stability of BCP-radicals.

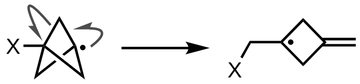			
	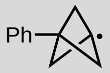	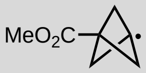	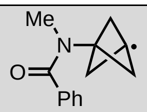
**ΔG‡ (Kcal mol–1) experimental**	25	21	–
**ΔG‡ (Kcal mol–1) calculated**	23.9	25.3	24.0

Table S5. Staffane formation.

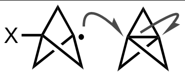			
	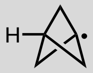	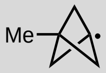	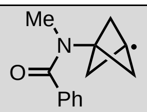
**ΔGº (Kcal mol–1)**	–24.3	–25.1	–26.4
**ΔG‡ (Kcal mol–1)**	11.4	10.2	9.5

Table S6. Strain-release with the succinimidyl radical.

	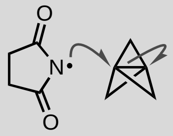
**ΔGº (Kcal mol–1)**	–35.6
**ΔG‡ (Kcal mol–1)**	8.0

9 Computational Studies

9.1 Computational Methods

Density functional theory (DFT)[4] calculations were performed using Gaussian 09 (revision E.01)[5] and the Gaussview[6] was used to generate input geometries and visualize output structures. Geometry optimizations and frequency calculations for the radical addition reactions and cyclobutyl radicals formation were performed using UM06-2X[7] levels of theory with the 6-31G* basis set.[8] For the structures containing Br atom, the LanL2DZ[9-11] basis set was used to describe the Br atom, while 6-31G* basis set was used for all the other atoms in the molecule. All stationary points were characterized as minima or transitions states based on normal vibrational mode analysis. Thermal corrections were computed from unscaled frequencies, assuming a standard state of 298.15 K and 1 atm.

For calculation of electronic properties of radicals, global and local electrophilicity index, B3LYP functional[12-15] was used and the geometries of studied radicals were optimized at the UB3LYP/6-311+G(d,p) level of theory, followed by frequency calculations at the same level.[16] For the structure containing I atom, the LanL2DZ[9-11] basis set was used to describe the I atom, while 6-311+G(d,p) basis set was used for all the other atoms in the molecule. The computed Hirshfeld charges[17] on the radicals were also calculated at the same level of theory. Homolytic bond dissociation enthalpies (BDE) were calculated using (RO)B3P86/6-311G(d,p) for the determination of geometries, frequencies (scaled by a factor of 0.9806) and molecular energies.[18-20]

For the calculation of reaction enthalpy (ΔHr) for the radical addition reactions, the reactants, transition states and products were fully optimized at the UB3LYP/6-31G(d) level of theory and the reaction enthalpy (ΔHr) was computed at the same level as the energy difference between the products and the reactants (ZPE corrected).[21] The bond distance [*d*(C–C)] between the radical centre and the double bond was calculated from the UB3LYP/6-31G(d) transition states structures and the amount of charge transfer (δ^{TS}) from the radical to the double bond in the transition states was evaluated from the Mulliken charges. The electronic properties such as adiabatic electron affinities and adiabatic ionization potentials for propellane and alkenes were computed from the energies of relaxed neutral molecules and corresponding relaxed ions at UB3LYP/6-31+G(d) level of theory and were ZPE corrected at the same level.[21] Spin density calculations were carried out at UB3LYP/6-31G(d) level of theory on the basis of Mulliken charges.[22] For substrates having more than one conformations, low energy conformation of the transition state could possibly be different

from the low energy ground state.[23] The structures described herein are the lowest energy-optimized conformers.

9.2 Activation Energy (ΔG≠) and Reaction Energy (ΔG)

DFT Method: UM062X/6-31G* [LanL2DZ basis set for Br atom, values are in Kcal mol^{-1}]

Radical Addition Reactions

Radical Addition Reactions	ΔG≠	ΔG
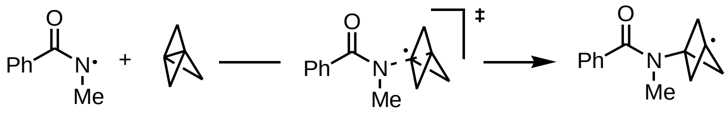	15.3	-12.4
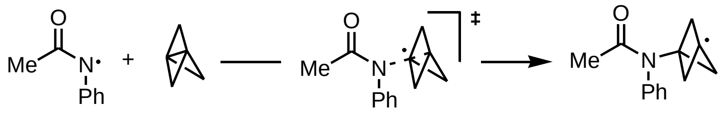	15.9	-6.7
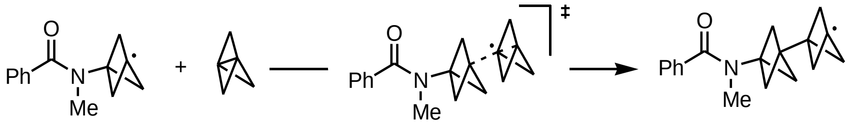	9.5	-26.4
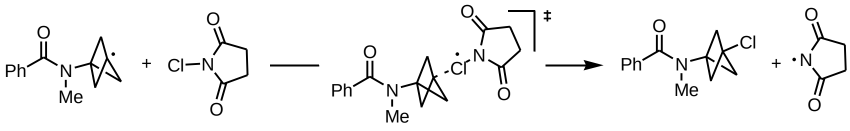	8.7	-20.9
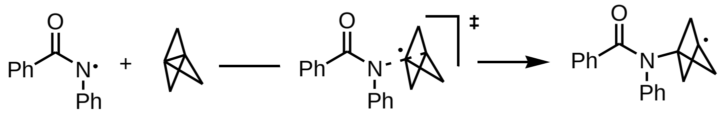	16.3	-4.0
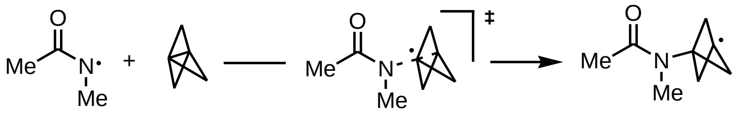	14.1	-15.4
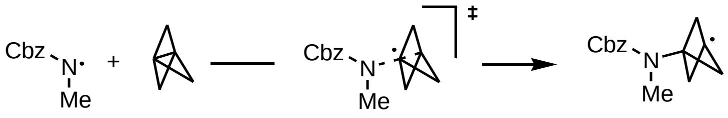	11.6	-17.5
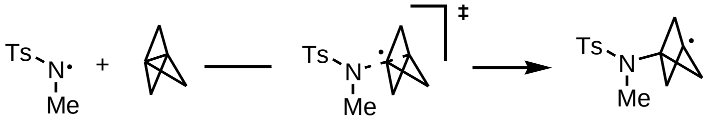	13.7	-9.9
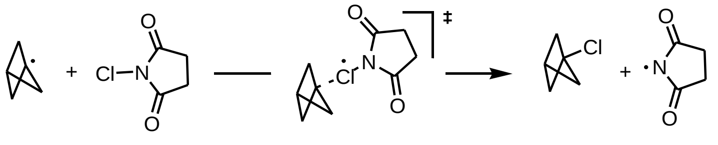	7.7	-20.4

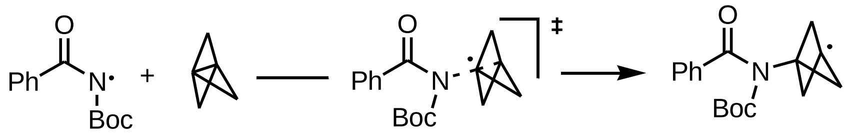	8.1	-21.2
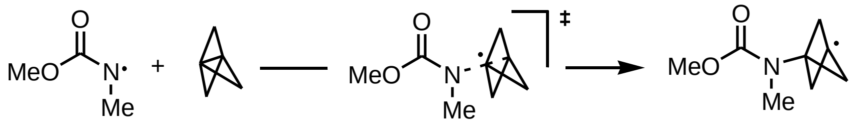	11.4	-18.4
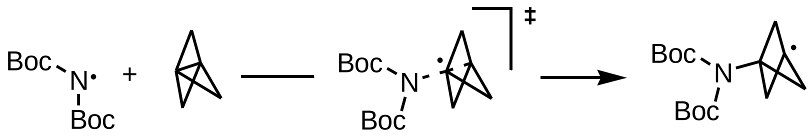	-1.2	-32.3
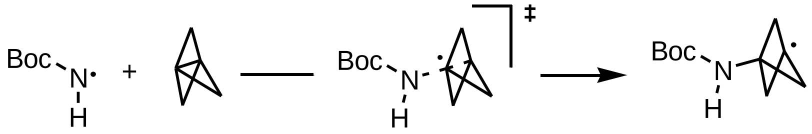	8.1	-23.8
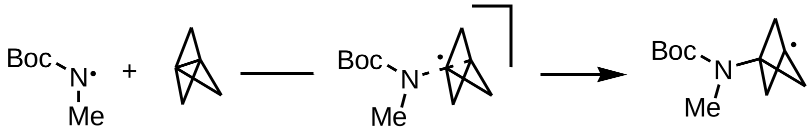	11.5	-18.3
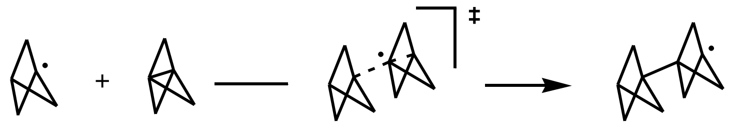	11.4	-24.3
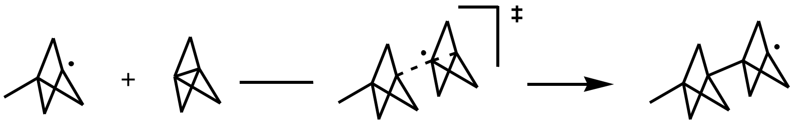	10.2	-25.1
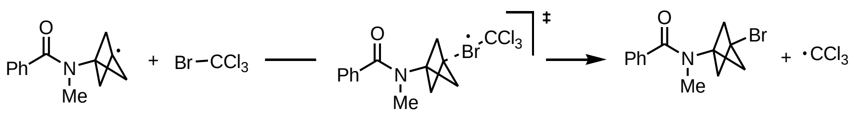	11.2	-30.2

Radical Addition Reactions to Olefins

DFT Method: UM062X/6-31G* [values are in Kcal mol^{-1}]

Radical Addition to Olefins	ΔG$^{\neq}$	ΔG
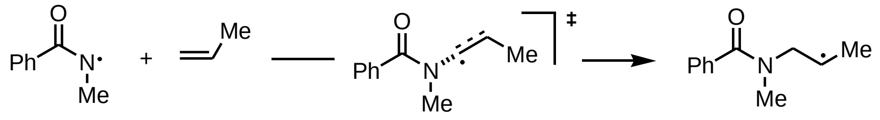	16.9	-15.6
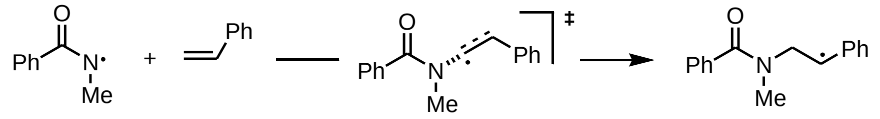	18.9	-17.8

Radical Addition Side-Reactions

DFT Method: UM062X/6-31G* [values are in Kcal mol^{-1}]

The values in parenthesis are for the reverse reactions.

**Radical Addition Side-Reactions**	**ΔG$^{\neq}$**	**ΔG**
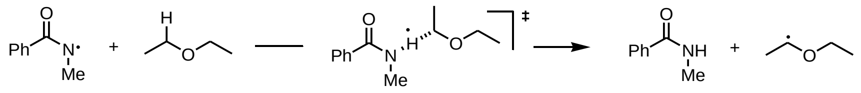	17.3<br>(25.3)	-8.0<br>(8.0)
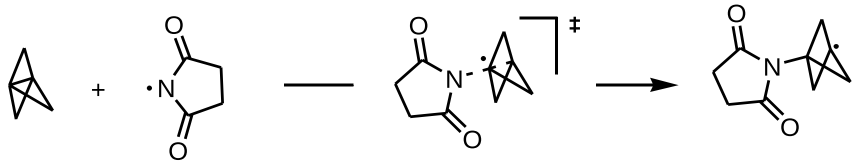	8.0<br>(43.6)	-35.6<br>(35.6)
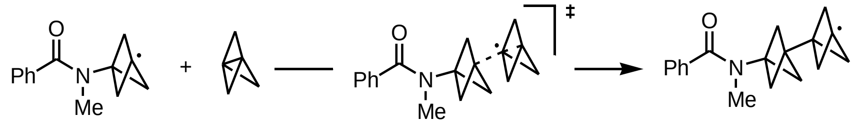	9.5<br>(35.9)	-26.4<br>(26.4)

Cyclobutyl Radicals Formation

DFT Method: mentioned in the table [values are in Kcal mol^{-1}]

Cyclobutyl radicals formation	UM062X	
	6-31G*	6-31+G*
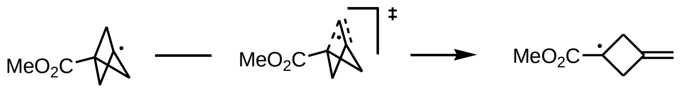	26.4	25.3
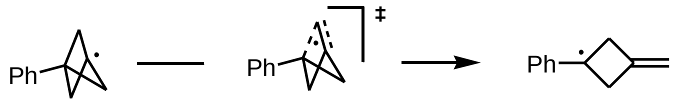	25.0	23.9
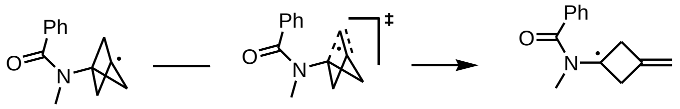	25.6	24.0

Computed Energies [values are in Hartree]

Species	Total Electronic Energy	Sum of Electronic and Zero-point Energies	Sum of Electronic and Thermal Enthalpies	Gibbs Free Energy
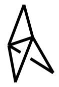	-193.9306631	-193.835553	-193.830744	-193.861799
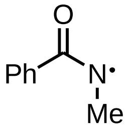	-439.394554	-439.250982	-439.241105	-439.286358
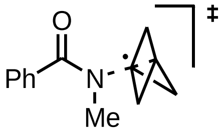	-633.3209283	-633.081238	-633.066715	-633.123762
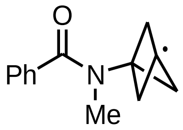	-633.3713273	-633.127109	-633.113391	-633.167874
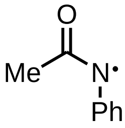	-439.4089763	-439.265942	-439.255957	-439.301991
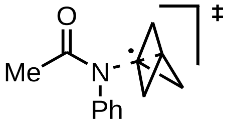	-633.3342134	-633.095289	-633.080648	-633.138498
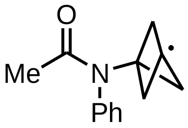	-633.3765166	-633.134210	-633.121045	-633.174403
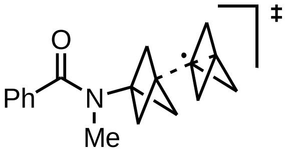	-827.3011271	-826.962001	-826.943098	-827.014578

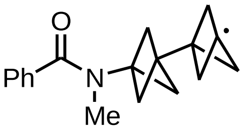	-827.3673504	-827.024746	-827.006696	-827.006696
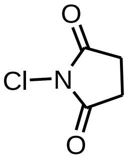	-820.038982	-819.955935	-819.948662	-819.987390
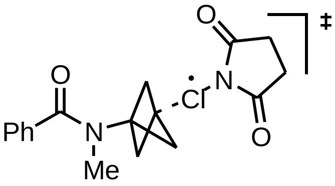	-1453.4114872	-1453.084800	-1453.062113	-1453.141456
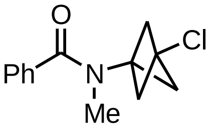	-1093.6249586	-1093.377507	-1093.362512	-1093.419714
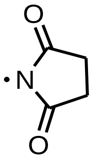	-359.8145493	-359.736503	-359.729416	-359.768882
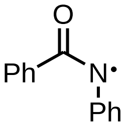	-631.0749688	-630.877485	-630.864861	-630.917773
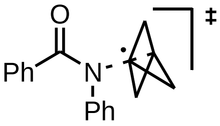	-824.9989929	-824.706024	-824.688592	-824.753658
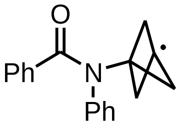	-825.0377524	-824.740716	-824.723990	-824.785980
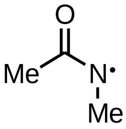	-247.728415	-247.639286	-247.632021	-247.669847

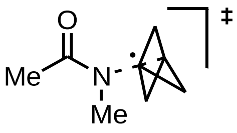	-441.6578096	-441.471779	-441.460256	-441.509252
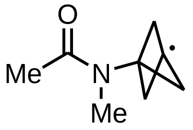	-441.7104369	-441.520337	-441.509555	-441.556247
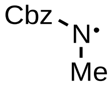	-553.8846114	-553.707080	-553.694733	-553.747570
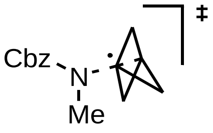	-747.8177216	-747.543601	-747.526814	-747.590889
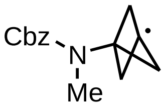	-747.8699511	-747.591735	-747.575577	-747.637249
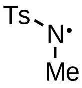	-913.8742794	-913.702439	-913.689244	-913.742944
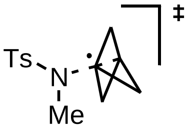	-1107.8049684	-1107.536377	-1107.518868	-1107.582909
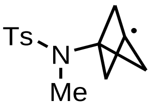	-1107.8489037	-1107.576261	-1107.559624	-1107.620491
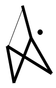	-194.4921076	-194.386558	-194.381705	-194.413566
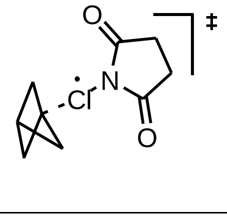	-1014.5353983	-1014.346919	-1014.334248	-1014.388615
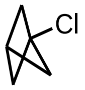	-654.7452323	-654.635736	-654.629831	-654.664537

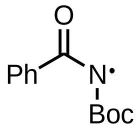	-745.779218	-745.534981	-745.518384	-745.580676
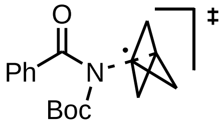	-939.7156705	-939.375264	-939.353842	-939.429614
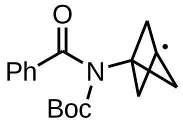	-939.7699497	-939.426077	-939.405569	-939.476283
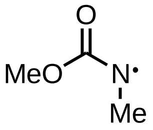	-322.9290974	-322.833375	-322.825521	-322.864871
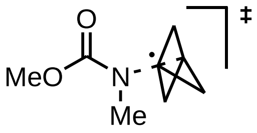	-516.8617201	-516.669562	-516.657233	-516.708439
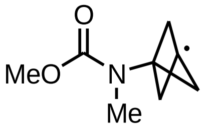	-516.9138487	-516.717927	-516.706073	-516.755926
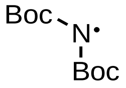	-747.1912889	-746.910121	-746.892186	-746.955153
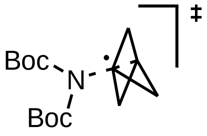	-941.1442496	-940.766421	-940.743512	-940.818910
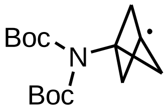	-941.1976116	-940.816810	-940.794324	-940.868498
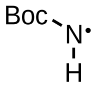	-401.5227445	-401.372034	-401.362200	-401.405790
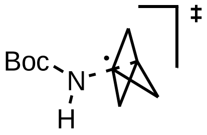	-595.4613135	-595.212752	-595.198076	-595.254632

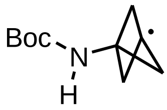	-595.5179505	-595.266271	-595.252661	-595.305556
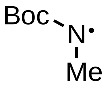	-440.8211671	-440.640627	-440.628881	-440.677208
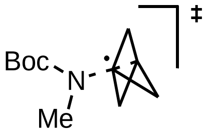	-634.7534542	-634.476481	-634.460162	-634.520668
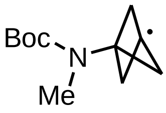	-634.8056732	-634.525217	-634.509412	-634.568128
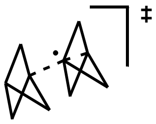	-388.4206708	-388.219783	-388.209917	-388.257212
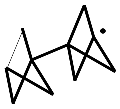	-388.484405	-388.279776	-388.270797	-388.314019
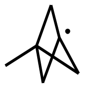	-233.7903977	-233.656451	-233.650033	-233.685505
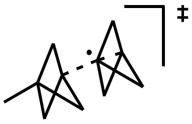	-427.7190165	-427.490171	-427.478567	-427.531069
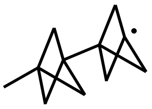	-427.7836458	-427.551225	-427.540580	-427.587282
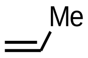	-117.8315453	-117.751199	-117.746718	-117.775784
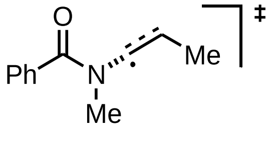	-557.2183557	-556.992669	-556.978132	-557.035248

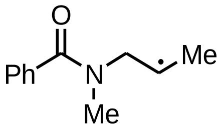	-557.2731083	-557.043980	-557.029563	-557.086976
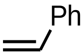	-309.4996504	-309.364797	-309.357048	-309.398016
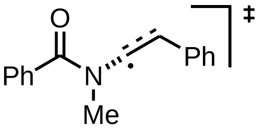	-748.8867257	-748.606615	-748.589341	-748.654321
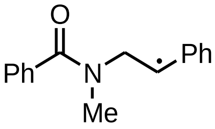	-748.9502454	-748.666233	-748.649365	-748.712699
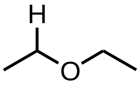	-233.5379643	-233.398873	-233.391162	-233.428946
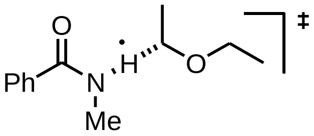	-672.9222946	-672.642097	-672.625021	-672.687721
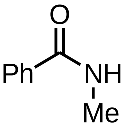	-440.0673549	-439.909178	-439.899188	-439.943748
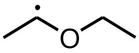	-232.8781373	-232.753268	-232.745355	-232.784245
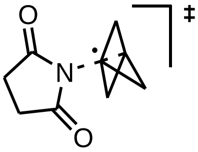	-553.7533007	-553.578214	-553.566471	-553.617934
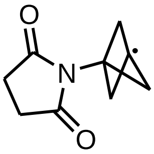	-553.8310099	-553.651661	-553.641609	-553.687485

Optimized Structures and Cartesian Coordinates

No.	Species	Optimized Structure
1	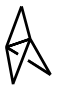	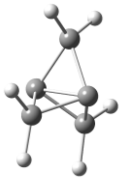

Cartesian Coordinates

C	-0.00015700	0.00030400	0.77503300
C	-1.13770700	0.61308100	-0.00021700
C	0.00012700	0.00029200	-0.77502100
C	1.10008000	0.67826300	0.00019400
C	0.03765400	-1.29168000	0.00001400
H	-0.86265400	-1.89959700	-0.00014200
H	0.97183100	-1.84614500	0.00018400
H	1.11356900	1.76456600	0.00018500
H	2.07658600	0.20208000	0.00038100
H	-1.21453400	1.69678000	-0.00024100
H	-2.08477700	0.08075700	-0.00038900

No.	Species	Optimized Structure
8	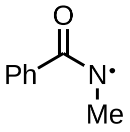	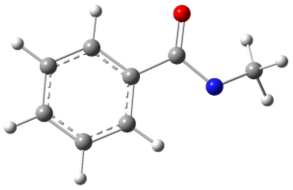

Cartesian Coordinates

N	-2.05900600	-0.77327600	-0.12888800
C	-1.25177000	0.36836200	-0.10208400
C	-3.42833600	-0.55767100	0.24611100
C	0.21507500	0.11166500	-0.05231400
C	1.07260600	1.20976700	0.05260500
C	0.73627400	-1.18381400	-0.09609000
C	2.44581800	1.01333200	0.11407300
H	0.64174500	2.20547500	0.08413900
C	2.11283000	-1.37596600	-0.03396100
H	0.05855600	-2.02572800	-0.18745600
C	2.96625100	-0.28014700	0.07128500
H	3.11334700	1.86551700	0.19476800
H	2.52045700	-2.38131700	-0.07174300
H	4.04030100	-0.43329900	0.11892600
O	-1.72256300	1.49461100	-0.17247300
H	-3.74397500	-1.39695600	0.87509200
H	-3.61132000	0.40174200	0.73744000
H	-4.03805600	-0.61255400	-0.66690900

9

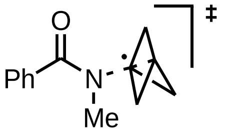

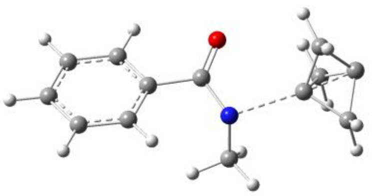

Cartesian Coordinates

C	4.07531700	-0.46361100	-0.12883800
C	3.10792100	-0.73044700	1.00145400
C	2.62301400	0.13507100	-0.13664900
C	3.83077200	1.03317700	-0.16423500
C	3.08777200	-0.78511600	-1.23222400
H	2.64954000	-1.77815900	-1.24276200
H	3.27159400	-0.33834700	-2.20587800
H	4.06124500	1.59193300	0.73992400
H	4.04730700	1.54908200	-1.09602600
H	3.31001600	-0.23600700	1.94931100
H	2.66702700	-1.72014100	1.06980500
N	0.52915000	0.80527000	-0.12483900
C	0.51620800	1.64595100	1.05658200
H	1.15434200	2.51495600	0.88141600
H	-0.50664600	1.99540400	1.25270000
H	0.86416100	1.11786300	1.95482300
C	-0.21385100	-0.36938600	-0.05942300
C	-1.70891300	-0.16383600	-0.08089200
C	-2.29661500	1.01749200	-0.53982200
C	-2.51454500	-1.21821800	0.35268300
C	-3.68218700	1.14584200	-0.55025400
H	-1.66594300	1.82057600	-0.90945200
C	-3.89804900	-1.08440100	0.34906600
H	-2.03540400	-2.13389000	0.68414100
C	-4.48276500	0.09843300	-0.10065400
H	-4.13695400	2.06102000	-0.91668200
H	-4.52260300	-1.90339600	0.69236500
H	-5.56375400	0.20114900	-0.10725100
O	0.25469300	-1.49307900	-0.05916500

10

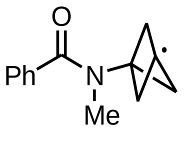

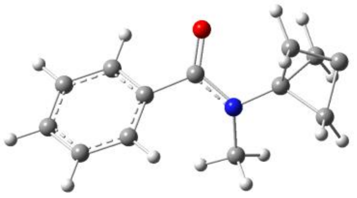

Cartesian Coordinates

C	3.93216400	-0.24491900	-0.26616600
C	3.08754200	-0.76280500	0.90665400
C	2.20487400	0.11044900	-0.02625500
C	3.33443500	1.16074200	-0.09339700
C	2.81227500	-0.60688300	-1.25198400
H	2.55390700	-1.65839800	-1.35933700

H	2.78309400	-0.04892500	-2.19008800
H	3.57188200	1.68657100	0.83414100
H	3.33981000	1.81187900	-0.97004900
H	3.31050300	-0.35002600	1.89331600
H	2.84546700	-1.82504900	0.92096900
N	0.80825700	0.45121200	0.15089700
C	0.56877400	1.58912700	1.02948900
H	-0.48063500	1.63231500	1.31561500
H	1.16947900	1.49016300	1.94219200
H	0.84434000	2.52529900	0.53330000
C	-0.09872200	-0.57066300	-0.03701100
C	-1.55990900	-0.21837800	-0.03077000
C	-2.04443800	0.97812500	-0.56421200
C	-2.45562100	-1.18167600	0.43510700
C	-3.41445400	1.21709700	-0.60953300
H	-1.34669700	1.71033500	-0.96200400
C	-3.82351700	-0.93516400	0.40445500
H	-2.06081400	-2.12123600	0.80844100
C	-4.30385200	0.26495500	-0.11634300
H	-3.78853900	2.14370100	-1.03374600
H	-4.51710200	-1.68146200	0.77908500
H	-5.37248300	0.45478300	-0.14648400
O	0.25658500	-1.71606100	-0.26272900

11

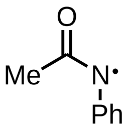

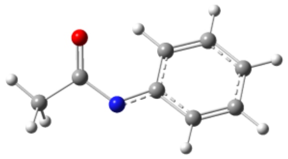

Cartesian Coordinates

N	-1.03393500	-0.81446600	0.12931700
C	-2.11730800	0.06815300	0.15278500
C	-3.30488900	-0.43328200	-0.63453100
O	-2.14053700	1.08972600	0.81237100
H	-3.65327300	-1.37640200	-0.20415400
H	-4.10189800	0.30954300	-0.60427600
H	-3.01480800	-0.63687500	-1.66966600
C	0.22436900	-0.35885800	0.05211100
C	1.26032600	-1.33112200	0.19592400
C	0.60432100	0.99727800	-0.20102700
C	2.58854700	-0.97278300	0.10585000
H	0.95414300	-2.35519000	0.38235400
C	1.93886600	1.33547800	-0.29010800
H	-0.16626500	1.75084400	-0.30600900
C	2.93559500	0.36223300	-0.13683300
H	3.36435600	-1.72248900	0.22282200
H	2.21949900	2.36639400	-0.48169200
H	3.98113200	0.64504700	-0.20859200

12

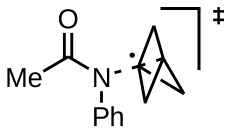

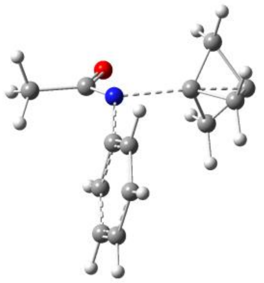

Cartesian Coordinates

C	-2.62826800	-1.80859600	0.47375100
C	-1.81944400	-0.90254000	1.37651800
C	-1.63084600	-0.74387900	-0.11108100
C	-1.44641700	-2.20620600	-0.39660800
C	-3.09556900	-0.69578800	-0.44638700
H	-3.66057600	0.11900500	-0.00477400
H	-3.38176700	-0.97848400	-1.45622300
H	-0.61495300	-2.70734900	0.09390900
H	-1.66829900	-2.55090200	-1.40356900
H	-0.99937200	-1.35813700	1.92758500
H	-2.34055300	-0.09466800	1.88152500
N	-0.18798200	0.76442100	-0.73111600
C	-0.68351500	1.93816600	-0.17600000
C	0.19131200	3.15820100	-0.44826000
O	-1.77608300	2.05264200	0.34153600
C	1.02153900	0.23443900	-0.34288800
C	1.64545400	-0.68119400	-1.21376800
C	1.62586200	0.50134100	0.90465300
C	2.84085900	-1.28719500	-0.86115400
H	1.15991000	-0.88902600	-2.16223400
C	2.82961800	-0.09988500	1.24323400
H	1.13128300	1.17752600	1.59839900
C	3.44171700	-0.99556800	0.36530600
H	3.31243200	-1.98813700	-1.54314200
H	3.28974200	0.11902200	2.20211500
H	4.37961100	-1.46866600	0.63849300
H	1.25487500	2.94310200	-0.32802700
H	0.02612100	3.47452100	-1.48284500
H	-0.11772900	3.96232900	0.22042300

13

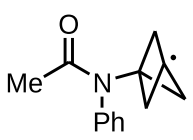

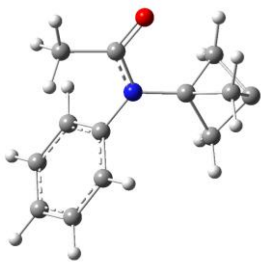

Cartesian Coordinates

C	-2.67420600	-1.83490700	0.19611300
C	-2.46983300	-0.70132400	1.20962200

C	-1.45088200	-0.55413500	0.05092700
C	-1.15363800	-2.06135000	0.12939900
C	-2.58430100	-0.83412900	-0.96487000
H	-3.35587500	-0.06909500	-1.03357500
H	-2.26920600	-1.23079000	-1.93269100
H	-0.66023500	-2.41125900	1.03853800
H	-0.76074200	-2.52752200	-0.77649100
H	-2.05194200	-0.97835800	2.17988300
H	-3.23416300	0.07242900	1.26361700
N	-0.42675600	0.47285700	-0.06344500
C	-0.84846900	1.78322500	-0.14875700
C	0.23179300	2.83647500	-0.30254400
O	-2.03222900	2.06656300	-0.11380500
H	0.78234900	2.96621800	0.63371700
H	0.95564500	2.56206500	-1.07432600
H	-0.25935800	3.77394300	-0.56126200
C	0.94467900	0.08175400	-0.02584600
C	1.52525400	-0.52371800	-1.14124300
C	1.69968600	0.27757000	1.13038900
C	2.85720600	-0.92453600	-1.10006800
H	0.92327800	-0.66819400	-2.03418700
C	3.03537900	-0.11248100	1.16392800
H	1.22789200	0.73278100	1.99668600
C	3.61489400	-0.71499700	0.04997700
H	3.30588100	-1.39428600	-1.96979300
H	3.62076100	0.04757400	2.06407200
H	4.65546400	-1.02269100	0.07820400

17	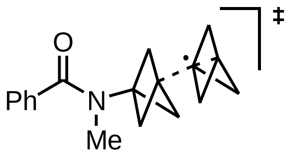	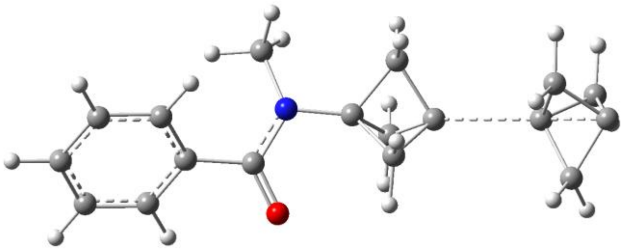

Cartesian Coordinates

C	2.18795300	-0.00309400	-0.48136600
C	1.18640700	-0.43558300	-1.56855100
C	0.42071900	0.27834400	-0.42290300
C	1.23836000	-0.52861400	0.60639400
C	1.50682700	1.37407900	-0.39867200
H	1.58106000	2.01498900	-1.28084300
H	1.62251100	1.92322200	0.53934100
H	1.04422300	-1.59931200	0.63259800
H	1.33322400	-0.07763900	1.59764400
H	0.98765000	-1.50221800	-1.67249500
H	1.24058400	0.09317800	-2.52346600
C	4.51599800	-0.13358300	0.12216000
C	4.88449000	0.79526200	1.24496200

C	5.04793700	-1.37878500	0.77261000
C	5.95612300	-0.16310300	0.75996300
C	5.73612400	0.14051100	-0.71053800
H	6.03601400	-0.62269000	-1.42403200
H	5.90086800	1.16481100	-1.03505300
H	5.31803400	-2.20678600	0.12243700
H	4.61466400	-1.66568800	1.72761600
H	4.44589200	0.59791300	2.22024000
H	5.01239400	1.84727300	1.00192600
N	-1.00050700	0.55565400	-0.35772000
C	-1.41386100	1.77041600	-1.04764200
C	-1.82898900	-0.52736200	-0.15961000
C	-3.28857100	-0.25688000	0.08154600
C	-3.73601100	0.84300800	0.81671300
C	-4.20539300	-1.20883500	-0.36583100
C	-5.09296000	0.99944800	1.08242100
H	-3.01663200	1.56332600	1.19738400
C	-5.56278100	-1.04296600	-0.11481400
H	-3.83334100	-2.07753700	-0.89994800
C	-6.00797200	0.06205500	0.60820500
H	-5.43493500	1.84993500	1.66412900
H	-6.27415500	-1.77913000	-0.47605900
H	-7.06730800	0.18837100	0.81033200
O	-1.40272900	-1.67039800	-0.11817500
H	-0.94573500	1.81401100	-2.03890100
H	-2.49427700	1.78298200	-1.18033900
H	-1.11174400	2.65866800	-0.48335100

18

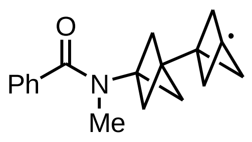

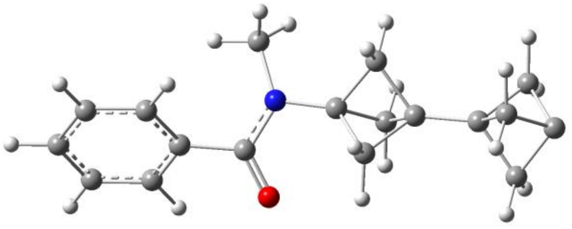

Cartesian Coordinates

C	2.39604900	0.09050300	-0.00654200
C	1.47184400	-0.44016500	-1.13148400
C	0.57056000	0.33814900	-0.13853300
C	1.32095500	-0.36019700	1.01359600
C	1.64600100	1.44338200	-0.10413400
H	1.79862600	2.01484900	-1.02547100
H	1.66898300	2.07741900	0.78779300
H	1.15150400	-1.43262700	1.11344900
H	1.31910300	0.16299300	1.97487800
H	1.31052100	-1.51911400	-1.17127800
H	1.61399700	0.00592500	-2.12133800
C	3.87772400	-0.09799300	0.10043100
C	4.61306800	-1.45738700	0.21694200

C	4.92724600	0.35635500	-0.94582900
C	5.64974300	-0.32275100	0.22823600
C	4.78478800	0.46507900	1.22424800
H	4.91229500	1.54944900	1.24442500
H	4.64949400	0.03121000	2.21701700
H	5.06405400	1.43412400	-1.05747800
H	4.91908200	-0.17540300	-1.89949100
H	4.58619700	-2.09831600	-0.66650300
H	4.46597000	-2.00667200	1.14896700
N	-0.84176500	0.58399900	-0.22367100
C	-1.21293500	1.75217700	-1.01241700
C	-1.66672800	-0.50482400	-0.03748000
C	-3.14707400	-0.25909800	0.04517300
C	-3.68604700	0.86622300	0.67240100
C	-3.99618000	-1.25577000	-0.43725800
C	-5.06534800	1.00264500	0.79549300
H	-3.02164700	1.62295500	1.08136100
C	-5.37457500	-1.11046700	-0.32964300
H	-3.55596900	-2.14153900	-0.88424100
C	-5.91017700	0.01935500	0.28564800
H	-5.48096400	1.87344400	1.29300800
H	-6.03305900	-1.88163700	-0.71708600
H	-6.98662400	0.12951800	0.37651000
O	-1.22107000	-1.63123500	0.11375000
H	-0.66052600	1.75353300	-1.96009300
H	-2.27749700	1.73548600	-1.23888600
H	-0.97609800	2.67499100	-0.47293700

19

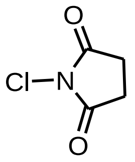

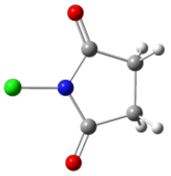

Cartesian Coordinates

C	0.42026100	1.18734300	-0.00018000
C	0.42026100	-1.18734300	-0.00023100
C	1.88295100	-0.76723300	-0.00004200
C	1.88295100	0.76723300	-0.00004700
H	2.36199000	-1.20090100	0.88081500
H	2.36218500	-1.20091500	-0.88078300
H	2.36201500	1.20090500	0.88079400
H	2.36216000	1.20091100	-0.88080400
N	-0.32468700	0.00000000	-0.00008900
O	-0.04098800	2.29330900	0.00003800
O	-0.04098800	-2.29330900	0.00003800
Cl	-2.00931100	0.00000000	0.00017600

20	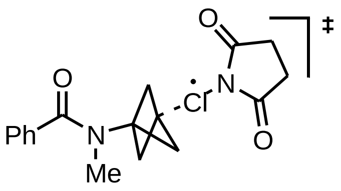	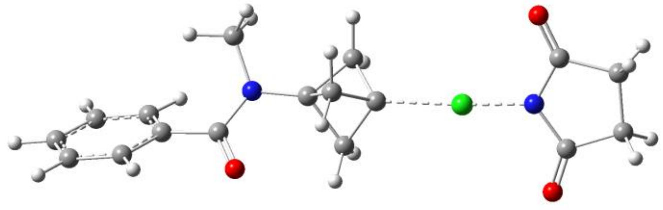

Cartesian Coordinates

C	0.71805200	0.18291500	-0.05596000
C	-0.12909300	0.38857300	1.19228300
C	-1.01485400	0.41628200	-0.09424900
C	0.02810900	1.33173800	-0.78269100
C	-0.26204400	-0.83349200	-0.62765100
H	-0.41495000	-1.75284100	-0.06834300
H	-0.25974500	-0.95926100	-1.71077700
H	0.16510200	2.32785700	-0.35973400
H	0.05188600	1.30714000	-1.87287300
H	-0.00019100	1.33655900	1.71652500
H	-0.27819700	-0.47098200	1.84306000
N	-2.43822200	0.63414500	-0.17053700
C	-2.83870900	2.03748700	-0.16889500
H	-3.90997600	2.12062500	0.00460400
H	-2.31774200	2.57819600	0.63066000
H	-2.59336400	2.50710900	-1.12639000
C	-3.22936000	-0.38387800	0.33299400
C	-4.71167000	-0.28217700	0.12191700
C	-5.26667900	0.26036700	-1.03937100
C	-5.54246000	-0.85220000	1.08753800
C	-6.64596300	0.25232500	-1.22135500
H	-4.61524600	0.66613300	-1.80889200
C	-6.92142700	-0.84499800	0.91154200
H	-5.08878700	-1.30437200	1.96378000
C	-7.47398200	-0.29128300	-0.24179800
H	-7.07353000	0.66380200	-2.13022900
H	-7.56665200	-1.27847800	1.66919200
H	-8.55054100	-0.29257500	-0.38240500
O	-2.74371900	-1.36208700	0.87448900
Cl	2.90255900	-0.03465200	-0.02455400
N	4.72830900	-0.12736200	-0.00823300
C	5.41870100	-1.26332700	-0.41094700
C	5.51054900	0.94478500	0.39709200
C	6.90591700	-0.94734600	-0.24800100
C	6.96584800	0.49573800	0.26648200
H	7.39369600	-1.08819000	-1.21531200
H	7.47387200	1.18096100	-0.41652300
H	7.44336300	0.59303300	1.24431600
H	7.33433800	-1.67489600	0.44548200
O	5.10895900	2.01452300	0.77434600

O	4.93062900	-2.28834500	-0.80769900

21

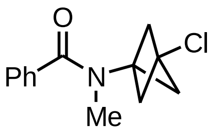

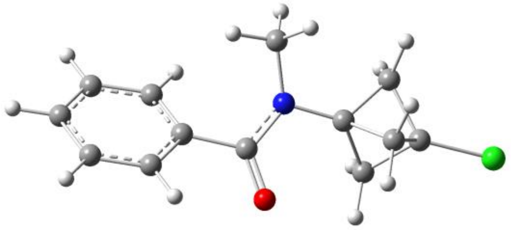

Cartesian Coordinates

C	3.18437900	-0.03246100	-0.08413100
C	2.29928700	-0.55049200	1.06014500
C	1.39692500	0.25968400	0.08518300
C	2.50242000	1.34019800	0.03151200
C	2.10319900	-0.45759200	-1.08964700
H	1.90938000	-1.52509900	-1.18347100
H	2.10234400	0.07163600	-2.04578900
H	2.69653500	1.89683700	0.95221900
H	2.52980200	1.96994600	-0.86132700
H	2.48241300	-0.11051700	2.04414900
H	2.11701500	-1.62502000	1.09182400
N	-0.00186900	0.54318700	0.19965200
C	-0.32487300	1.69971300	1.02776100
H	-1.38629900	1.70745400	1.26859000
H	0.24006100	1.65742500	1.96707800
H	-0.06829900	2.62907500	0.50935400
C	-0.85766900	-0.52581300	0.01525500
C	-2.33103300	-0.24206000	-0.03890200
C	-2.85055100	0.91214700	-0.62932400
C	-3.19705100	-1.22936900	0.43290400
C	-4.22758400	1.08667200	-0.72484000
H	-2.17384900	1.66150300	-1.03151400
C	-4.57277800	-1.04643300	0.35190500
H	-2.77305900	-2.13697800	0.85068700
C	-5.08881900	0.11214200	-0.22535100
H	-4.62871100	1.98020700	-1.19285300
H	-5.24437500	-1.81040100	0.73079700
H	-6.16333700	0.25192800	-0.29488900
O	-0.43784700	-1.65828900	-0.15598000
Cl	4.92124300	-0.30070700	-0.24878800

22

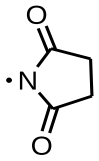

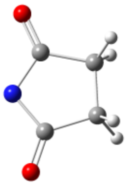

Cartesian Coordinates

C	1.13285700	0.24503700	-0.00249800
C	-1.13287600	0.24502400	0.00248700
C	-0.76305300	-1.23727000	0.02786200
C	0.76305500	-1.23726100	-0.02787300

H	-1.16607300	-1.68177100	0.94195200
H	-1.24163500	-1.73956600	-0.81611200
H	1.24165500	-1.73957900	0.81607700
H	1.16606600	-1.68173600	-0.94198100
N	0.00001000	1.08598300	-0.00009700
O	2.25749000	0.69672000	0.01277000
O	-2.25748700	0.69673000	-0.01266000

36	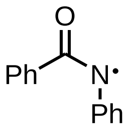	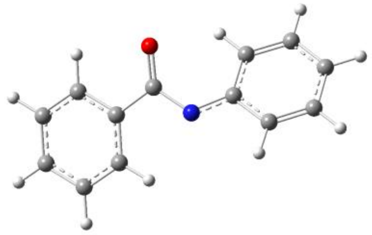

Cartesian Coordinates

N	-0.50479100	-0.42767100	-0.62133700
C	0.44135000	0.59660700	-0.56732100
O	0.19725500	1.75687200	-0.85771100
C	-1.76996500	-0.21937700	-0.23375600
C	-2.69400100	-1.27550800	-0.50143900
C	-2.25156400	0.93731900	0.45848100
C	-4.01448700	-1.17871300	-0.11850200
H	-2.30983200	-2.14751000	-1.02041600
C	-3.57600000	1.01326100	0.83646200
H	-1.56750700	1.74920900	0.66958700
C	-4.46312200	-0.03363700	0.55199900
H	-4.70573100	-1.98736400	-0.33298400
H	-3.93439200	1.89306300	1.36157000
H	-5.50223600	0.04224100	0.85665900
C	1.81998300	0.14806300	-0.20254100
C	2.12471200	-1.19556600	0.02613900
C	2.81275600	1.12219400	-0.07100700
C	3.41780500	-1.55997800	0.38798000
H	1.34718200	-1.94249400	-0.09123900
C	4.10269400	0.75509800	0.29026900
H	2.54952300	2.15842200	-0.25769300
C	4.40551500	-0.58661900	0.52077600
H	3.65616900	-2.60437000	0.56391400
H	4.87390600	1.51204800	0.39383900
H	5.41435700	-0.87338200	0.80256400

37	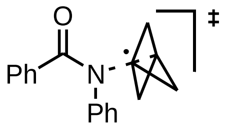	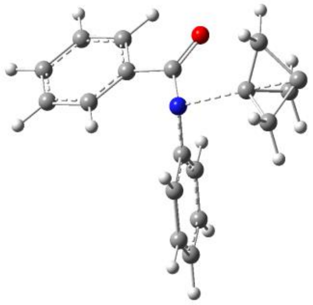

Cartesian Coordinates

C	3.70978500	-1.80877800	0.16405100
C	2.72163400	-1.32704000	1.20439100
C	2.32418800	-1.18488000	-0.24321400
C	3.61147400	-0.55504100	-0.69147500
C	2.68518100	-2.58984900	-0.63860500
H	2.14658800	-3.38400200	-0.13118700
H	2.91105700	-2.76617000	-1.68714000
H	3.89194800	0.38681700	-0.22511100
H	3.87636100	-0.65144100	-1.74163700
H	2.96904700	-0.41133700	1.73729200
H	2.18234700	-2.07754700	1.77451800
N	0.39361500	-0.28172400	-0.61855800
C	-0.50231500	-1.14431200	0.00231900
O	-0.19068900	-2.19892400	0.52497800
C	-1.95773200	-0.75559200	-0.11585900
C	-2.41069800	0.26231500	-0.95844200
C	-2.87408500	-1.48342700	0.64625700
C	-3.76941700	0.55254400	-1.02831300
H	-1.70421300	0.81864900	-1.56594500
C	-4.23020600	-1.18619700	0.58021100
H	-2.49973400	-2.28068900	1.27994500
C	-4.67928500	-0.16682200	-0.25717600
H	-4.11871500	1.33984600	-1.68908500
H	-4.93794800	-1.75132800	1.17895100
H	-5.73897600	0.06416600	-0.31189100
C	0.54747600	1.01567800	-0.18195700
C	1.17856500	1.92842500	-1.05138000
C	0.15910900	1.45688400	1.10060800
C	1.39215500	3.24058200	-0.65984900
H	1.48663700	1.56891200	-2.02862400
C	0.36655200	2.77564400	1.47817100
H	-0.30597100	0.75254400	1.78539000
C	0.98348600	3.67171500	0.60422100
H	1.87505400	3.93551900	-1.34010500
H	0.05328100	3.10771400	2.46321800
H	1.14824300	4.70072000	0.90773600

38

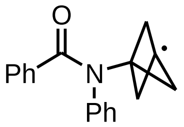

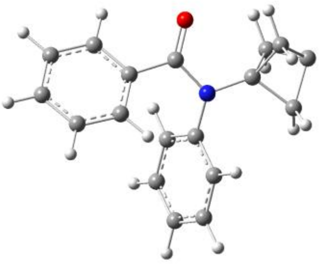

Cartesian Coordinates

C	3.80848300	-1.42591000	-0.04308400
C	2.85393500	-1.33982900	1.15457600
C	2.12282300	-0.87417300	-0.13322300

C	3.35553100	-0.03612600	-0.51899800
C	2.71068600	-2.08267900	-0.89364200
H	2.34849200	-3.05990800	-0.58082400
H	2.79418200	-1.96889800	-1.97639100
H	3.58768500	0.81294800	0.12640100
H	3.48174400	0.18446600	-1.58131900
H	3.06023200	-0.56417600	1.89529200
H	2.49857200	-2.27616600	1.58264400
N	0.74319600	-0.39856200	-0.21762000
C	-0.23367900	-1.34125300	0.07284800
O	0.05914300	-2.47821600	0.40027000
C	0.54808900	0.99760700	0.01967700
C	0.93499600	1.92102800	-0.95268800
C	0.01012400	1.44383000	1.22675800
C	0.77984600	3.28295700	-0.71833800
H	1.34434500	1.55687300	-1.89044500
C	-0.16106100	2.80705600	1.44872300
H	-0.27941700	0.71723400	1.98089000
C	0.22648900	3.72898900	0.48039300
H	1.08284900	3.99681200	-1.47812100
H	-0.58908700	3.14877000	2.38596200
H	0.09827200	4.79195000	0.65844100
C	-1.67277900	-0.94076300	-0.08648500
C	-2.10886100	-0.00755900	-1.02958900
C	-2.60596300	-1.61669400	0.70161400
C	-3.46732500	0.25655100	-1.16805700
H	-1.38983300	0.50331100	-1.66181700
C	-3.96190400	-1.33841800	0.57370600
H	-2.24820200	-2.36456400	1.40213000
C	-4.39368500	-0.40015800	-0.36125900
H	-3.80393700	0.97548900	-1.90846800
H	-4.68286500	-1.85884200	1.19648900
H	-5.45302300	-0.18637200	-0.46728800

39

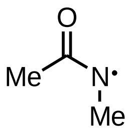

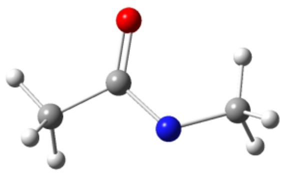

Cartesian Coordinates

N	-0.63459700	-0.67346800	-0.30678400
C	0.46418900	0.15426900	-0.04708600
C	-1.90446000	-0.17298300	0.14380200
O	0.37267800	1.36821500	-0.03975000
H	-2.20075200	-0.76377200	1.02343700
H	-1.87072500	0.88621500	0.41772700
H	-2.66003700	-0.35504000	-0.62570200
C	1.76600800	-0.58995700	0.10843800
H	2.00328000	-1.10338200	-0.82768000

H	2.56004200	0.11196500	0.36220500
H	1.67452000	-1.35540400	0.88458100

40

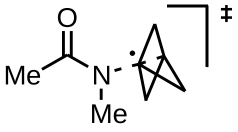

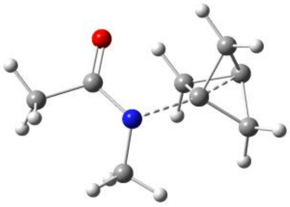

Cartesian Coordinates

C	2.56957800	-0.39062200	0.27082700
C	1.42471000	-0.43406600	1.25325000
C	1.15208000	0.14588000	-0.11494100
C	2.35531400	1.05287600	-0.13446600
C	1.79041700	-0.97910500	-0.88416200
H	1.34436500	-1.95893600	-0.74855300
H	2.14325600	-0.75143500	-1.88649000
H	2.43181200	1.80256700	0.64951700
H	2.73488200	1.35706900	-1.10634000
H	1.46449100	0.26162800	2.08843700
H	0.97215000	-1.39927400	1.45999900
N	-0.99468800	0.60335300	-0.62361400
C	-1.18497800	1.87924500	0.02885000
H	-2.18790200	2.26398000	-0.20560800
H	-1.08207500	1.84255700	1.12242100
H	-0.45859300	2.58828400	-0.37344100
C	-1.67763000	-0.49563700	-0.13306800
C	-3.15159900	-0.24714600	0.17848900
O	-1.19284700	-1.60627000	-0.02667700
H	-3.24639100	0.32327300	1.10886900
H	-3.63857600	0.32261600	-0.61756000
H	-3.63917600	-1.21419500	0.29879700

41

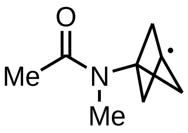

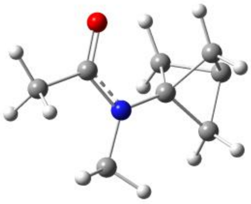

Cartesian Coordinates

C	2.50957300	-0.36128200	0.06060200
C	1.48392000	-0.56932200	1.18246600
C	0.79069200	0.09402600	-0.03803000
C	1.98169800	1.06380600	-0.17309500
C	1.51349300	-0.90664100	-0.97156300
H	1.20351900	-1.94516400	-0.87493900
H	1.64739100	-0.58084100	-2.00518700
H	2.12471300	1.78197800	0.63782200
H	2.15005600	1.49522300	-1.16258900
H	1.59308700	0.05447400	2.07254100
H	1.17694600	-1.59184600	1.39885300

N	-0.59872100	0.49572300	-0.13481600
C	-0.90739600	1.90465000	0.03449700
H	-1.88387500	2.13353800	-0.39344000
H	-0.90481600	2.21027200	1.08949500
H	-0.16183400	2.49815600	-0.49851900
C	-1.53696200	-0.50296300	-0.02531800
O	-1.21473800	-1.67728600	0.03432700
C	-2.99524000	-0.08032500	-0.00315100
H	-3.59395100	-0.98679900	0.07426200
H	-3.21149200	0.57141600	0.84898600
H	-3.26946200	0.45613600	-0.91664400

42	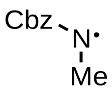	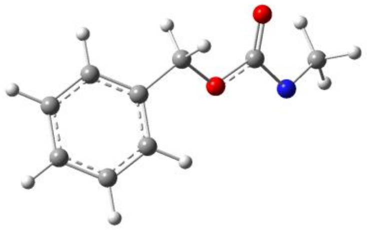

Cartesian Coordinates

N	3.15025900	-0.94902700	-0.16505700
C	2.32997400	0.14421000	0.13093900
C	4.48231800	-0.56955100	-0.54936200
O	2.71961400	1.23569300	0.48826100
H	4.87830200	-1.32888300	-1.22812700
H	4.56030300	0.43430100	-0.97786100
H	5.10515400	-0.59497500	0.35787400
O	1.04345400	-0.20045400	0.02069400
C	0.11602200	0.84004000	0.34646100
H	0.32426700	1.71890600	-0.27256400
H	0.28028200	1.13245400	1.39031200
C	-1.28206200	0.32919800	0.12737400
C	-1.61149600	-1.00290100	0.38278400
C	-2.27655000	1.21075300	-0.29418200
C	-2.92123800	-1.44222500	0.21886700
H	-0.83462200	-1.69305300	0.69686700
C	-3.58868100	0.77322600	-0.44995500
H	-2.02248500	2.24660100	-0.50642900
C	-3.91348600	-0.55589500	-0.19438500
H	-3.16783100	-2.48131900	0.41506900
H	-4.35396100	1.46833300	-0.78153500
H	-4.93456200	-0.90221900	-0.32109900

43	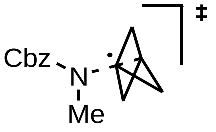	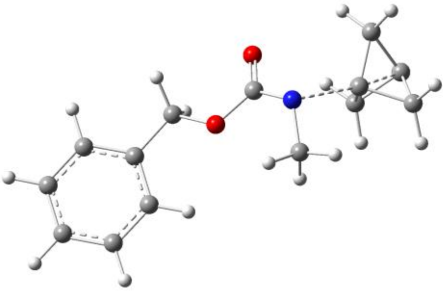

Cartesian Coordinates

C	4.62231200	-0.21931300	1.01886300
C	3.20775900	-0.15046200	1.53655500
C	3.43835900	0.17878700	0.07806100
C	4.58606600	1.12456500	0.32098900
C	4.28621600	-1.02918100	-0.21382100
H	3.80462600	-1.99207000	-0.07470800
H	4.96961900	-0.95750600	-1.05553000
H	4.39206400	1.99809900	0.93875300
H	5.28628700	1.27826000	-0.49569300
H	2.96164200	0.67651500	2.19864300
H	2.69899700	-1.08863400	1.73984400
N	1.63774300	0.44596300	-1.22663900
C	1.28723600	1.82503600	-0.96236400
H	0.40508900	2.07871400	-1.56466400
H	1.04351100	2.03080700	0.08779200
H	2.11723500	2.45945900	-1.27943800
C	0.81260400	-0.51908900	-0.67201500
O	1.14855500	-1.64713600	-0.38359300
O	-0.46527700	-0.08178100	-0.54554600
C	-1.39796100	-1.06524400	-0.10049200
H	-1.42590600	-1.89188800	-0.81857500
H	-1.04557200	-1.47808700	0.85162600
C	-2.75246300	-0.42242700	0.04650500
C	-2.89079900	0.94708600	0.27097700
C	-3.89417800	-1.22362000	-0.00139400
C	-4.15465000	1.50503300	0.44446900
H	-2.00536800	1.57388700	0.29700800
C	-5.15655200	-0.66775600	0.17980500
H	-3.79340300	-2.29084800	-0.18547600
C	-5.28993300	0.70048300	0.40228400
H	-4.25230400	2.57346800	0.61243700
H	-6.03680100	-1.30204100	0.13961400
H	-6.27422300	1.13807700	0.53742900

44

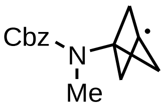

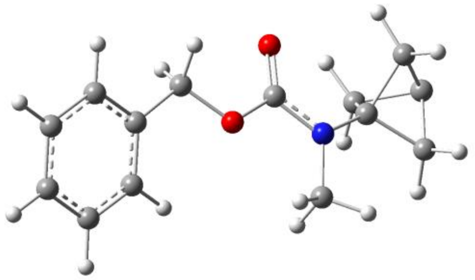

Cartesian Coordinates

C	4.85672800	-0.17347000	0.25989800
C	3.75230300	-0.18790600	1.32449100
C	3.13800400	0.16005100	-0.05837500
C	4.29862700	1.13905200	-0.31661500
C	3.96746800	-0.97912000	-0.69659800
H	3.68816600	-1.98781100	-0.39721500
H	4.17219700	-0.87763400	-1.76426100
H	4.34754800	2.02063800	0.32669400
H	4.52975900	1.35634100	-1.36178000
H	3.76743000	0.61761900	2.06191000
H	3.46434900	-1.15462400	1.73688800
N	1.74990100	0.45833200	-0.34096500
C	1.33973800	1.84606700	-0.48993200
H	0.40999400	1.89146300	-1.05557300
H	1.19081800	2.33952100	0.47903300
H	2.11614700	2.37944400	-1.04116600
C	0.85646500	-0.53522200	-0.05908300
O	1.15295700	-1.66951100	0.25508300
O	-0.41964300	-0.10366400	-0.18468400
C	-1.41240800	-1.09354800	0.08426000
H	-1.33875000	-1.89550200	-0.65765400
H	-1.20980400	-1.53904900	1.06443600
C	-2.76728000	-0.43695700	0.04701100
C	-2.93131500	0.90663700	0.38488600
C	-3.88865600	-1.19666200	-0.28713100
C	-4.19950200	1.47977900	0.38750500
H	-2.05932500	1.50152700	0.63760300
C	-5.15798300	-0.62655100	-0.27610400
H	-3.76625100	-2.24200800	-0.56109000
C	-5.31633000	0.71516600	0.06017500
H	-4.31603400	2.52790400	0.64652100
H	-6.02222800	-1.22840100	-0.53987800
H	-6.30499100	1.16375900	0.06277000

45

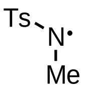

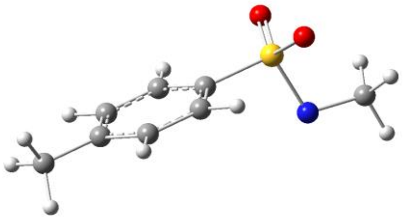

Cartesian Coordinates

N	2.09666500	0.00995000	1.21037900
C	3.54388300	0.01159300	1.25293300
H	3.93937600	0.89061400	0.72546200
H	3.86234300	0.02448000	2.29502200
H	3.93971300	-0.88037900	0.74788700
S	1.55482400	-0.00254600	-0.39516000
C	-0.20045100	-0.00364200	-0.18526500
C	-0.87402700	1.21251400	-0.11100400
C	-0.87067100	-1.21785700	-0.10293900
C	-2.25107500	1.20050100	0.06222700
H	-0.32197300	2.14258900	-0.20111900
C	-2.25086100	-1.20746400	0.07089500
H	-0.31772200	-2.14803800	-0.18635800
C	-2.95597200	-0.00516300	0.15762900
H	-2.79316000	2.14084800	0.11957800
H	-2.78978800	-2.14855100	0.13510400
C	-4.45166400	0.00781900	0.33802400
H	-4.84789200	-1.00288700	0.45906300
H	-4.73341800	0.59231900	1.21938200
H	-4.94376000	0.46342700	-0.52746400
O	1.95544200	1.26291800	-0.99787100
O	1.95924200	-1.27456100	-0.98108400

46

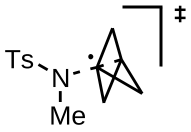

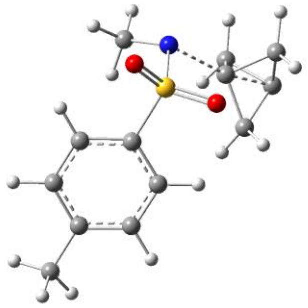

Cartesian Coordinates

C	-3.16941500	-2.08947100	-0.09832100
C	-2.85595500	-1.73480600	1.33805800
C	-2.31371200	-0.82619400	0.26077000
C	-1.75347800	-1.93828600	-0.59742100
C	-3.60727600	-0.69844500	-0.49849900
H	-4.44604800	-0.26361000	0.03810100
H	-3.52062700	-0.41854400	-1.54446100
H	-0.99389500	-2.57793600	-0.15428700
H	-1.62734500	-1.71010900	-1.65244400
H	-2.14462300	-2.36858400	1.86209400
H	-3.67326200	-1.35145300	1.94377200
N	-1.49792800	1.17585400	0.72210300
C	-0.90989100	1.02748000	2.04027600
H	-0.45131600	1.98366100	2.32903100
H	-1.71785700	0.80918500	2.74193600

H	-0.14726800	0.24067000	2.10675800
S	-0.43218700	1.51781200	-0.52312500
C	1.06772800	0.57658700	-0.29832100
C	2.03999400	1.05175900	0.57947900
C	1.26199200	-0.60435900	-1.00713800
C	3.20358900	0.31606900	0.76597400
H	1.88979800	1.99985100	1.08690000
C	2.43433000	-1.32678900	-0.81252700
H	0.51312200	-0.92656400	-1.72226100
C	3.41651900	-0.88172200	0.07648200
H	3.96551500	0.68175100	1.44930200
H	2.59738900	-2.24626500	-1.36886400
C	4.69973200	-1.65125600	0.25271600
H	4.55137100	-2.71875300	0.07028900
H	5.45962300	-1.29751700	-0.45290200
H	5.10305900	-1.52652900	1.26103400
O	-1.05693300	1.06232900	-1.75609000
O	-0.07333000	2.91909400	-0.32989500

47	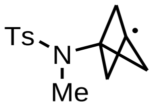	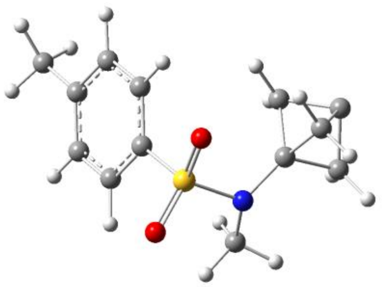

Cartesian Coordinates

C	2.87943500	-2.22466800	-0.05988600
C	1.37058700	-1.94750500	-0.10175200
C	2.13517700	-0.63682700	0.24130300
C	3.00375500	-1.42874500	1.24527800
C	3.15456500	-0.95191100	-0.87326700
H	2.77273300	-0.90972200	-1.89185100
H	4.13706000	-0.49193300	-0.75637300
H	2.49183400	-1.83295500	2.12139800
H	3.98114400	-0.99953000	1.47478300
H	0.75769500	-2.38267700	0.69061700
H	0.89918800	-1.98814300	-1.08392800
N	1.60931100	0.68384700	0.56544800
C	1.27064400	0.90486300	1.97017300
H	0.98395500	1.94956800	2.09873100
H	0.45906000	0.24878400	2.31686500
H	2.16403300	0.71423900	2.56793400
S	0.56896300	1.37586200	-0.54671500
C	-1.01619100	0.59703000	-0.31020200
C	-1.83052900	1.01390800	0.74002300
C	-1.42458400	-0.41475300	-1.17479300
C	-3.05057500	0.37848200	0.94405000

H	-1.52409400	1.84632000	1.36598100
C	-2.64966000	-1.03429300	-0.96007200
H	-0.79591200	-0.68155000	-2.01795800
C	-3.47234600	-0.65697300	0.10621700
H	-3.69271500	0.70151300	1.75889400
H	-2.98042100	-1.81708600	-1.63779300
C	-4.78471000	-1.35846700	0.34168700
H	-4.62447500	-2.32602700	0.82988500
H	-5.44273500	-0.76625000	0.98209300
H	-5.30413200	-1.54948200	-0.60156100
O	1.05834100	0.98951700	-1.85915100
O	0.42113200	2.76590300	-0.15096900

48	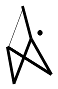	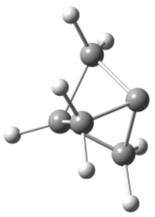

Cartesian Coordinates

C	0.00028700	0.00000300	-0.94287700
C	-0.62620600	1.08913300	-0.05717600
C	0.00018500	0.00006700	0.84290900
C	-0.63085800	-1.08654200	-0.05713600
C	1.25682500	-0.00267900	-0.05708500
H	1.86118900	0.90617700	-0.05257100
H	1.85754500	-0.91393500	-0.05265200
H	-1.72052100	-1.15066300	-0.05253300
H	-0.14618800	-2.06455700	-0.05229900
H	-1.71561600	1.15759800	-0.05260700
H	-0.13791100	2.06538800	-0.05243400
H	0.00010600	0.00010200	1.94329300

49	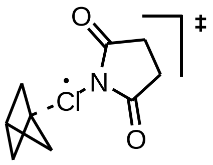	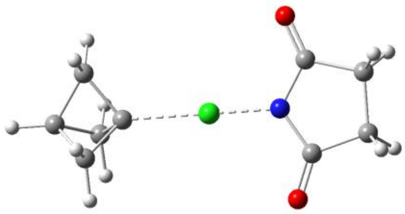

Cartesian Coordinates

C	-2.49832900	0.00002000	-0.00095400
C	-3.34544500	-0.33151100	-1.22229900
C	-4.24003900	0.00016300	0.00143900
C	-3.34224200	-0.89333800	0.89836500
C	-3.34271300	1.22463300	0.32449400
H	-3.33859900	2.03316800	-0.40591400
H	-3.33679900	1.56477600	1.35964200
H	-3.33769700	-1.95988100	0.67571100
H	-3.33693600	-0.66402300	1.96358600
H	-3.34163000	-1.36871500	-1.55628000

H	-3.34271700	0.39494200	-2.03450100
Cl	-0.26561700	0.00031500	-0.00062400
N	1.56160900	0.00007700	0.00035200
C	2.30015000	1.17487800	-0.00010500
C	2.29969200	-1.17497500	-0.00002800
C	3.77374500	0.76589200	0.00619000
C	3.77342900	-0.76656700	-0.00532800
H	4.24248300	1.19503900	0.89510300
H	4.25579200	-1.21163300	0.86812100
H	4.24268700	-1.19601500	-0.89381500
H	4.25693100	1.21085800	-0.86684500
O	1.85513100	-2.29322000	0.00182500
O	1.85594500	2.29325200	-0.00311200
H	-5.33738800	0.00017200	0.00298300

50

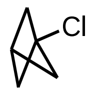

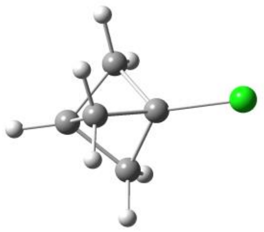

Cartesian Coordinates

C	-0.09607300	0.00001500	-0.00036700
C	0.80279500	-0.03665000	1.24282800
C	1.72792900	0.00000800	0.00038200
C	0.80358400	-1.05828300	-0.65312700
C	0.80357200	1.09492100	-0.58965600
H	0.79140800	2.05856600	-0.07531700
H	0.79212700	1.19605500	-1.67726700
H	0.79146800	-2.05045100	-0.19625600
H	0.79215900	-1.09532600	-1.74481400
H	0.79064900	-0.96365800	1.82042000
H	0.79064900	0.85472000	1.87402300
Cl	-1.87157700	0.00000100	-0.00011800
H	2.81751000	0.00000400	0.00085600

51

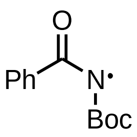

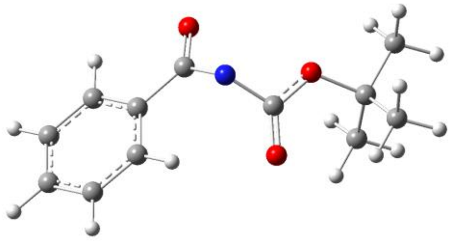

Cartesian Coordinates

N	0.04067300	-0.24067000	-1.32705700
C	1.04964500	0.40155300	-0.57715500
C	-0.88107000	-1.04342700	-0.59786300
O	0.91982400	1.54758100	-0.22109400
O	2.07899100	-0.40668400	-0.40620400
C	3.26882000	0.05005700	0.32429100

C	2.88529000	0.39876400	1.75783700
C	3.90339500	1.22419600	-0.41212700
C	4.17174400	-1.17519500	0.28366900
H	3.26118000	2.10560900	-0.38231500
H	4.85906800	1.46762700	0.06226500
H	4.09715800	0.95470500	-1.45479100
H	2.36562300	-0.44410400	2.22348400
H	3.79494200	0.60064800	2.33172200
H	2.24517000	1.28139700	1.79424100
H	3.68177700	-2.02491300	0.76649200
H	4.40044000	-1.44586600	-0.75066300
H	5.10769900	-0.96188100	0.80751100
O	-0.61381800	-2.20118200	-0.38228100
C	-2.15470900	-0.37669500	-0.22248000
C	-2.35542000	0.98944000	-0.43198500
C	-3.14778500	-1.15988000	0.37129500
C	-3.55878000	1.56984900	-0.04385800
H	-1.57117200	1.59227300	-0.88067600
C	-4.34777700	-0.57493900	0.75114900
H	-2.95693600	-2.21740300	0.52401100
C	-4.55208100	0.78952500	0.54282300
H	-3.72112600	2.63135000	-0.20011600
H	-5.12494300	-1.17788800	1.20993300
H	-5.49119900	1.24592800	0.84134600

52

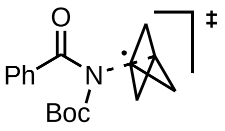

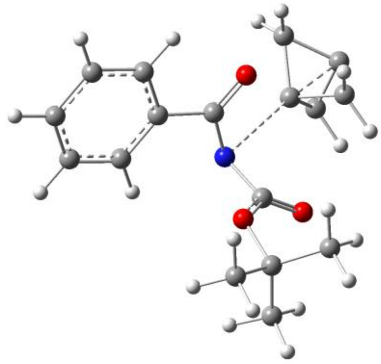

Cartesian Coordinates

C	4.41251100	-1.32172500	-0.25590100
C	3.45942500	-0.31090200	-0.83630100
C	3.03807500	-1.05261400	0.40839400
C	3.39535800	-2.42518100	-0.10113800
C	4.31702400	-0.82362200	1.16671600
H	4.59970200	0.20849000	1.35439600
H	4.56230100	-1.52716700	1.95782200
H	2.88102200	-2.76153300	-0.99676900
H	3.60326800	-3.19311900	0.63934600
H	2.93926800	-0.57403100	-1.75231400
H	3.70355700	0.73995900	-0.71284900
N	0.42924900	-0.24131200	0.69800100
C	-0.11987600	-1.07899700	-0.31066400
C	0.52624500	1.13132800	0.38778100

O	1.54338700	1.76678500	0.47544000
O	-0.68832900	1.58374200	0.06009600
C	-0.90644000	3.00588400	-0.21033200
C	-0.55748900	3.83140300	1.02352200
C	-0.09996700	3.42151800	-1.43593600
C	-2.40152000	3.06247200	-0.49655400
H	-0.86450800	4.86870900	0.85804000
H	-1.09657500	3.45018200	1.89645100
H	0.51390200	3.81084300	1.22664700
H	-0.37072300	4.44475900	-1.71418300
H	0.97154600	3.38311800	-1.23522500
H	-0.33198000	2.76298700	-2.27843900
H	-2.65072900	2.42823700	-1.35173600
H	-2.96940000	2.71473300	0.37118300
H	-2.69742200	4.09091300	-0.72225400
O	0.51341600	-1.41513700	-1.28524700
C	-1.50668300	-1.55030300	-0.03031100
C	-2.21288200	-1.15155100	1.10653700
C	-2.09581700	-2.40793400	-0.96222300
C	-3.50882200	-1.61451400	1.30803500
H	-1.74642100	-0.48521400	1.82498800
C	-3.38851800	-2.87031700	-0.75478800
H	-1.52241600	-2.69735200	-1.83680400
C	-4.09461800	-2.47352400	0.38051100
H	-4.06106300	-1.30968100	2.19125000
H	-3.84695800	-3.54047700	-1.47506200
H	-5.10494100	-2.83682600	0.54310000

53	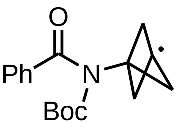	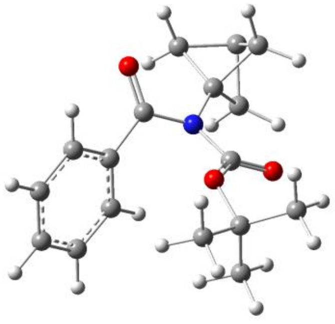

Cartesian Coordinates

C	-4.34565600	-0.52676400	-0.32501800
C	-3.67182500	0.27727800	0.80001300
C	-2.59353000	-0.35250000	-0.10819300
C	-3.36427500	-1.68653000	-0.09635600
C	-3.39313000	0.08958500	-1.35684200
H	-3.45519800	1.16295000	-1.52465000
H	-3.21249700	-0.48750500	-2.26619600
H	-3.41070000	-2.20459500	0.85951700
H	-3.18095900	-2.35264500	-0.94212300
H	-3.74018700	-0.13090300	1.80950500
H	-3.75827100	1.36348100	0.75509100

N	-1.14307500	-0.22720700	0.03308700
C	-0.42018000	-1.30373900	0.60813500
C	-0.62622100	1.06576300	-0.07392400
O	-1.24128000	1.98292900	-0.56682200
O	0.58531600	1.13317900	0.47934000
C	1.44940700	2.28871300	0.26593100
C	1.63141600	2.54220500	-1.22726400
C	0.87359200	3.49856900	0.99223100
C	2.76026300	1.83111600	0.89471100
H	2.36842300	3.33871700	-1.36801600
H	2.00910700	1.63760200	-1.71516100
H	0.69395000	2.84503300	-1.69668000
H	1.58575800	4.32798800	0.93750200
H	-0.07029400	3.80925400	0.54156300
H	0.70529700	3.25804800	2.04632200
H	2.61629500	1.61148000	1.95650000
H	3.12038200	0.92440400	0.39676500
H	3.51602500	2.61583400	0.79662100
O	-0.94679800	-2.04884300	1.40234000
C	0.97302600	-1.53789100	0.11596100
C	1.37571400	-1.17484000	-1.17079800
C	1.86092900	-2.19394900	0.96660100
C	2.66931300	-1.45355900	-1.59648700
H	0.67161500	-0.68290300	-1.83694800
C	3.16056500	-2.45612200	0.54567900
H	1.51890800	-2.48698800	1.95402200
C	3.56593200	-2.08543600	-0.73476900
H	2.97898000	-1.17978000	-2.60051900
H	3.85635200	-2.95318000	1.21431700
H	4.57860700	-2.29535400	-1.06555800

54	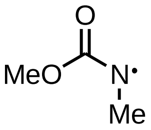	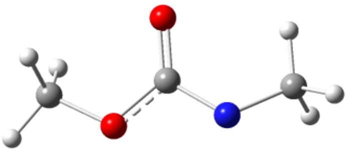

Cartesian Coordinates

N	-1.17724300	-0.65879200	-0.34189500
C	-0.00910000	0.08193800	-0.12983400
C	-2.34904700	-0.06588000	0.24323500
O	0.05793800	1.29191500	-0.12101600
H	-2.47941100	-0.49199200	1.25046400
H	-2.25821100	1.02102800	0.34317200
H	-3.22776400	-0.33770800	-0.34459500
O	1.04160100	-0.73459600	-0.01263000
C	2.29094100	-0.06667500	0.15646000
H	3.03179500	-0.85505100	0.27739400
H	2.51618300	0.54283200	-0.72143600
H	2.26503400	0.57757800	1.03827500

55

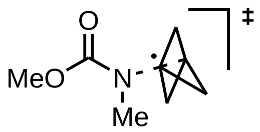

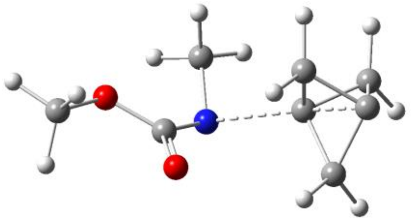

Cartesian Coordinates

C	2.76070800	-0.69454100	0.40622000
C	1.50491000	-0.66800700	1.24152900
C	1.48749500	0.07829800	-0.07373600
C	2.79183900	0.79841800	0.14852600
C	2.06285600	-1.04679600	-0.88944800
H	1.47435900	-1.95845500	-0.91931300
H	2.56708900	-0.77601800	-1.81311000
H	2.86267500	1.44916500	1.01677400
H	3.32912200	1.13804200	-0.73299600
H	1.52590800	-0.07120400	2.15045000
H	0.90863000	-1.57526100	1.28355500
N	-0.46902300	0.89927200	-0.76796600
C	-0.55720900	2.12967500	-0.01100900
H	-1.48446100	2.64395300	-0.29462400
H	-0.57744800	1.98547600	1.07632200
H	0.28788900	2.76447800	-0.28471200
C	-1.30908400	-0.13064200	-0.37367500
O	-1.08508100	-1.31287400	-0.51916500
O	-2.47296900	0.33292700	0.14402000
C	-3.42907400	-0.68009700	0.44146000
H	-3.03489000	-1.37004300	1.19092900
H	-4.30556000	-0.15745200	0.82297700
H	-3.68039400	-1.24585400	-0.45853500

56

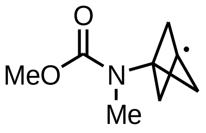

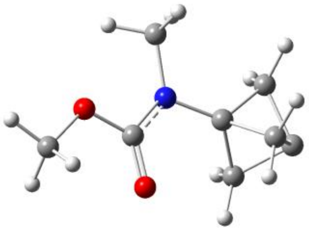

Cartesian Coordinates

C	2.79987000	-0.63523200	0.10265500
C	1.73935300	-0.65920800	1.21077500
C	1.16427400	0.05033000	-0.04512400
C	2.47904900	0.84093100	-0.18792600
C	1.75281500	-1.07248800	-0.93068000
H	1.29975200	-2.05439200	-0.80611200
H	1.94592800	-0.80586800	-1.97172800
H	2.70551900	1.56019700	0.60252900
H	2.71814700	1.21104500	-1.18740100
H	1.92233400	-0.02684100	2.08236600

H	1.28889300	-1.62150900	1.45352100
N	-0.14960600	0.64113200	-0.18377000
C	-0.28712200	2.07353700	0.02660600
H	-1.24464800	2.40730300	-0.36866600
H	-0.22777800	2.34061200	1.08951000
H	0.51567000	2.58404200	-0.50927700
C	-1.20522000	-0.21964800	-0.06667600
O	-1.11052600	-1.42883400	-0.01850100
O	-2.38679700	0.43593900	-0.02496300
C	-3.52117600	-0.42211100	0.05883100
H	-3.47347300	-1.03359900	0.96223600
H	-4.38698100	0.23826200	0.08734000
H	-3.56860000	-1.08067000	-0.81098300
57	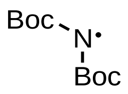	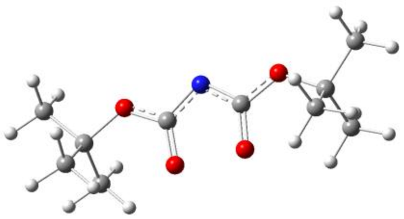	
Cartesian Coordinates			
N	-0.00000800	0.97704600	-0.00022000
C	1.07481900	0.18979000	-0.00014500
C	-1.07481400	0.18977900	-0.00011500
O	1.01725700	-1.08251600	-0.00011000
O	2.24046000	0.81037700	-0.00008200
C	3.50999200	0.08622800	0.00006700
C	3.63744800	-0.75071000	-1.26858100
C	3.63769800	-0.74988100	1.26925000
C	4.52750700	1.22063700	-0.00039900
H	2.92957300	-1.58022900	1.27851900
H	4.65199800	-1.15609600	1.33002600
H	3.46583000	-0.12267300	2.14930400
H	3.46459100	-0.12409300	-2.14888300
H	4.65196200	-1.15631500	-1.32982100
H	2.92986700	-1.58152200	-1.27687200
H	4.39977300	1.84528900	-0.88862500
H	4.39911100	1.84639800	0.88696100
H	5.54116700	0.81039600	0.00022700
O	-1.01713000	-1.08246800	-0.00006800
O	-2.24046900	0.81038400	-0.00008700
C	-3.51000700	0.08622200	0.00004200
C	-4.52756900	1.22061000	0.00066400
C	-3.63787500	-0.74990200	-1.26910900
C	-3.63729500	-0.75072200	1.26872400
H	-4.65180100	-1.15633700	1.32992900
H	-2.92965800	-1.58148900	1.27700300
H	-3.46444600	-0.12414000	2.14903400
H	-4.65223600	-1.15595300	-1.32988200

H	-3.46584800	-0.12270400	-2.14913600
H	-2.92993000	-1.58040800	-1.27836000
H	-5.54122700	0.81036700	0.00023200
H	-4.39966100	1.84519700	0.88892200
H	-4.39937600	1.84645700	-0.88665800

58	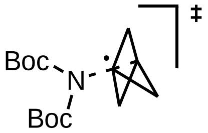	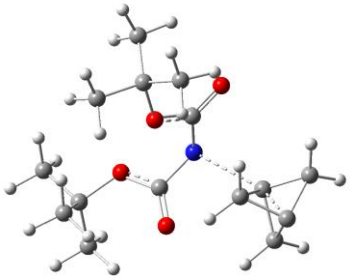

Cartesian Coordinates

C	3.43921700	-2.04538700	0.86601100
C	3.94392600	-1.43651600	-0.42209500
C	2.45789500	-1.54092800	-0.21983900
C	2.44822300	-2.98102300	0.21987100
C	2.45064600	-0.95514000	1.17540700
H	2.84704100	0.05104700	1.28613200
H	1.59697200	-1.20372500	1.79920100
H	2.84236300	-3.71333200	-0.47933000
H	1.59402300	-3.29821700	0.80928700
H	4.39591500	-2.10781300	-1.14678300
H	4.38213200	-0.44352700	-0.36860300
N	0.19581900	-0.58234400	-1.31731700
C	-0.77162600	-1.06371700	-0.40342700
C	0.72527600	0.70038200	-1.04337500
O	1.60848200	1.17437100	-1.71067700
O	0.11143100	1.28955800	-0.00539300
C	0.39245700	2.68559300	0.32072000
C	1.82520800	2.82003600	0.82433200
C	0.11735000	3.57461400	-0.88892400
C	-0.60356300	2.97853000	1.43573700
H	1.96935000	2.20866800	1.72046900
H	2.53878100	2.51601600	0.05613200
H	2.01886700	3.86421100	1.08896400
H	0.13713700	4.62204400	-0.57233200
H	0.86097000	3.42979700	-1.67284300
H	-0.87691800	3.35743400	-1.29215700
H	-1.62892100	2.90226000	1.06112300
H	-0.47523000	2.26647900	2.25624100
H	-0.44677400	3.99036000	1.82029400
O	-0.53906200	-1.85691600	0.47419600
O	-1.95422600	-0.56606300	-0.74899700
C	-3.14652800	-0.88250800	0.03777300
C	-2.94968500	-0.42904200	1.48077000
C	-4.22511500	-0.05339000	-0.64719800
C	-3.45413600	-2.37214500	-0.06746000

H	-3.90049800	-0.51371700	2.01606500
H	-2.20169900	-1.03813500	1.98984100
H	-2.63127400	0.61765000	1.50166800
H	-3.97248500	1.01010500	-0.60232300
H	-4.31992500	-0.34450800	-1.69700600
H	-5.18710000	-0.20827900	-0.15056400
H	-3.51831400	-2.66893800	-1.11867300
H	-2.68588200	-2.96846000	0.42658800
H	-4.41952000	-2.57476900	0.40697200

59	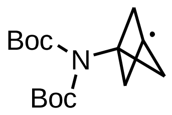	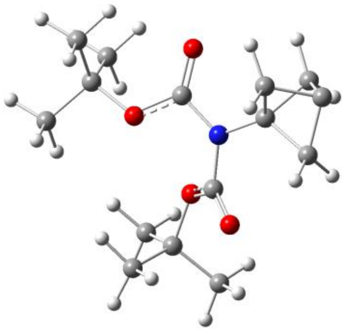

Cartesian Coordinates

C	-4.24978800	-0.16867800	0.07207300
C	-3.34372700	0.14879000	1.26922800
C	-2.47753100	-0.10705200	0.01220800
C	-3.33585700	-1.33891400	-0.32418100
C	-3.43038200	0.78057200	-0.81717700
H	-3.44139700	1.83941700	-0.55929000
H	-3.44751300	0.58731500	-1.89120300
H	-3.26703100	-2.17632300	0.37192400
H	-3.33734300	-1.65102100	-1.36764400
H	-3.28124900	-0.60778100	2.05390100
H	-3.34110800	1.17148100	1.64328600
N	-1.01793200	-0.06359700	-0.03148500
C	-0.30163200	-1.25004000	-0.27141900
C	-0.43480100	1.18907700	0.16682300
O	-1.03701600	2.10519700	0.67844400
O	0.79206900	1.24570300	-0.35443000
C	1.62713500	2.41862400	-0.12441500
C	1.01387800	3.64731600	-0.78958600
C	1.84068900	2.61064500	1.37349100
C	2.93285100	2.03855600	-0.81240100
H	0.80643800	3.43711100	-1.84340600
H	0.08889900	3.94349800	-0.29531100
H	1.72745900	4.47583300	-0.73916200
H	2.58872000	3.39234200	1.53752300
H	0.91315500	2.90135000	1.86934500
H	2.20830400	1.67959100	1.81739700
H	3.35742900	1.13837500	-0.35801400
H	2.76042800	1.84413000	-1.87512200
H	3.65677100	2.85281500	-0.71735100

O	-0.71816100	-2.13698600	-0.97580000
O	0.82621400	-1.27109000	0.43760400
C	1.84419300	-2.27784800	0.16101600
C	2.27437400	-2.18294200	-1.29914100
C	2.98091500	-1.86451200	1.08793700
C	1.33051100	-3.66592000	0.53104500
H	3.14925300	-2.81952100	-1.46305500
H	1.47568200	-2.50656400	-1.96861900
H	2.54516600	-1.14910800	-1.53780400
H	3.32446100	-0.85499500	0.84380600
H	2.64586200	-1.87498700	2.12906700
H	3.82079600	-2.55696700	0.98216900
H	0.96719200	-3.66623100	1.56342100
H	0.52315200	-3.97785400	-0.13161300
H	2.15217000	-4.38536000	0.45660500

60

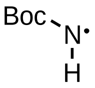

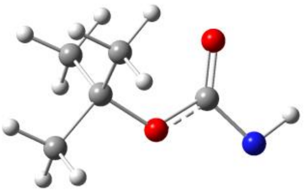

Cartesian Coordinates

N	2.53410000	0.94987600	0.00007700
C	1.44559100	0.05408600	-0.00037800
O	1.56387100	-1.15664200	0.00008500
O	0.30508600	0.73595500	-0.00030000
C	-0.97443100	0.03649900	0.00003100
C	-1.98031600	1.18095200	-0.00074900
C	-1.11236900	-0.80073300	1.26827600
C	-1.11208900	-0.80224000	-1.26724100
H	-2.99867100	0.78224900	-0.00060600
H	-1.84619800	1.80571200	0.88666600
H	-1.84603200	1.80465000	-0.88888600
H	-2.12929400	-1.20117300	1.32436400
H	-0.40773700	-1.63325700	1.27672700
H	-0.94186600	-0.17509800	2.15000300
H	-2.12897600	-1.20281500	-1.32303500
H	-0.94146000	-0.17764500	-2.14967900
H	-0.40739300	-1.63472500	-1.27456500
H	3.35895600	0.33708800	0.00055800

61

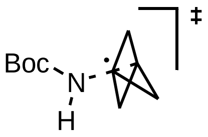

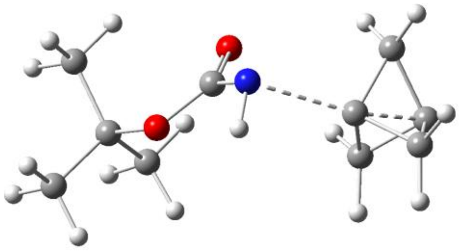

Cartesian Coordinates

C	-3.48595300	0.58653000	-0.58518700
C	-2.11925900	1.21632800	-0.52652500
C	-2.37308300	-0.00242700	0.33776000
C	-3.67625300	0.49690000	0.91246400
C	-2.99643200	-0.82834300	-0.76099300
H	-2.35268700	-1.04728300	-1.60735100
H	-3.66138800	-1.62891800	-0.44916000
H	-3.65299800	1.42861700	1.47149300
H	-4.37389100	-0.25008300	1.28083000
H	-2.03509200	2.17490800	-0.02051100
H	-1.46002100	1.06926100	-1.37819700
N	-0.60910000	-1.12805000	1.20008200
C	0.32358200	-0.74583200	0.23727800
O	0.20031300	-0.96675300	-0.94880700
O	1.39232700	-0.14840200	0.80601800
C	2.54804900	0.21930900	0.00110400
C	3.15994800	-1.02013500	-0.64554300
C	2.15044100	1.27212500	-1.03039300
C	3.50260600	0.81285000	1.03089300
H	3.37314100	-1.77328600	0.11948000
H	2.48828700	-1.44764700	-1.39056000
H	4.10228200	-0.74766400	-1.13127500
H	3.05051300	1.66839200	-1.51114400
H	1.49899400	0.84708300	-1.79462500
H	1.63340900	2.10114000	-0.53589500
H	3.04773700	1.68094200	1.51687900
H	3.74244400	0.07089800	1.79784500
H	4.43001900	1.12863300	0.54449800
H	-0.51004700	-0.50122500	2.00428300

62

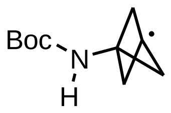

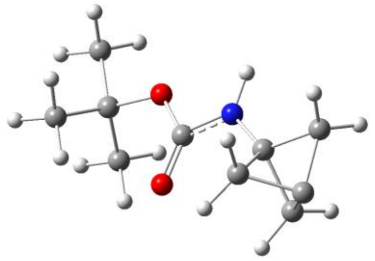

Cartesian Coordinates

C	-3.83292300	-0.34436500	0.00019700
C	-2.78855600	-0.62154700	-1.08954900
C	-2.16743600	0.28859200	-0.00011800
C	-3.44508900	1.14639800	-0.00004500
C	-2.78825100	-0.62127600	1.08971800
H	-2.37885300	-1.62922500	1.15011100
H	-2.95323100	-0.15621800	2.06368800
H	-3.65297200	1.70982000	-0.91224700
H	-3.65271600	1.71005100	0.91207200
H	-2.95381200	-0.15672800	-2.06358600
H	-2.37917000	-1.62950700	-1.14980800

N	-0.83856500	0.82923100	-0.00039300
C	0.24307100	0.00039700	-0.00042800
O	0.16497200	-1.21157500	-0.00010800
H	-0.69206600	1.82751400	-0.00033500
O	1.37555500	0.73026700	-0.00030700
C	2.66878500	0.06466700	0.00011900
C	3.64853500	1.23253600	-0.00004800
C	2.83335300	-0.77060200	-1.26639700
C	2.83292600	-0.76987700	1.26717100
H	4.67631300	0.85862500	0.00024000
H	3.50113200	1.85312800	-0.88868800
H	3.50081700	1.85363900	0.88818100
H	3.85853000	-1.15066700	-1.31953300
H	2.14199300	-1.61367600	-1.27442500
H	2.65218200	-0.14940700	-2.14942200
H	3.85809300	-1.14989100	1.32088700
H	2.65143200	-0.14818300	2.14977700
H	2.14158500	-1.61296400	1.27543900

63	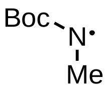	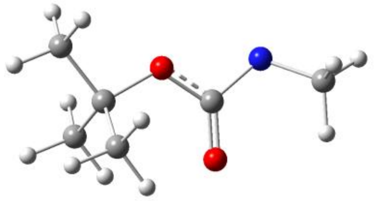

Cartesian Coordinates

N	-2.13227700	0.67896600	0.46904900
C	-1.00098400	-0.10317400	0.19355000
O	-1.03412700	-1.30955600	0.06942900
O	0.08243200	0.66955100	0.17088700
C	1.40143400	0.08146700	-0.03960600
C	2.31836000	1.29837100	-0.02187100
C	1.46020600	-0.61477100	-1.39624100
C	1.73582800	-0.86306100	1.11079400
H	3.35611700	0.98441600	-0.16591300
H	2.04623700	1.99217200	-0.82227400
H	2.23724700	1.82144000	0.93487800
H	2.49345700	-0.91023500	-1.60342900
H	0.83017400	-1.50458600	-1.41568900
H	1.13766700	0.07304700	-2.18439000
H	2.77625600	-1.19004400	1.01781100
H	1.62341300	-0.34257900	2.06677500
H	1.08857600	-1.74077000	1.10245500
C	-3.29425600	0.29316000	-0.28612000
H	-3.27219000	0.81439600	-1.25610400
H	-3.30393500	-0.78442100	-0.48590500
H	-4.19704900	0.60249300	0.24287700

64	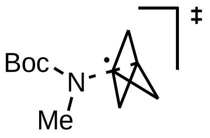	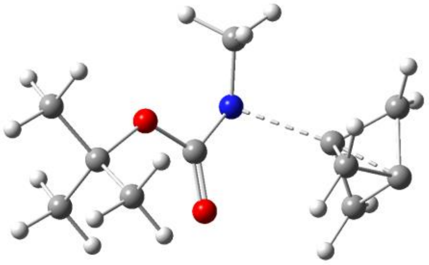

Cartesian Coordinates

C	3.54538100	-0.84746900	0.51426700
C	2.22265500	-0.73814100	1.23245400
C	2.39019500	0.04749600	-0.04875300
C	3.72293100	0.64509200	0.31919500
C	2.94397800	-1.09613600	-0.85255300
H	2.28789200	-1.95270000	-0.97133300
H	3.55190900	-0.84044000	-1.71609700
H	3.76579400	1.26011400	1.21488600
H	4.36595000	0.96491700	-0.49642400
H	2.20871800	-0.17360300	2.16196900
H	1.55213700	-1.59213100	1.18723800
N	0.59115200	1.07023700	-0.86323300
C	-0.36768600	0.10376700	-0.58253700
O	-0.22277000	-1.07997600	-0.80938000
O	-1.49547200	0.65598000	-0.08590200
C	-2.67005500	-0.16512800	0.16467100
C	-3.68456400	0.84827100	0.68301300
C	-2.36139800	-1.20900500	1.23490400
C	-3.16194600	-0.79932900	-1.13323100
H	-3.31119000	1.32962200	1.59164800
H	-3.86838300	1.62020600	-0.06976800
H	-4.63034500	0.34930100	0.91315600
H	-3.28763500	-1.71375500	1.52771200
H	-1.65380000	-1.95129900	0.86470000
H	-1.94218400	-0.72171000	2.12140100
H	-3.31404400	-0.02614400	-1.89280500
H	-2.44828200	-1.53325900	-1.50849200
H	-4.12090200	-1.29526600	-0.95216500
C	0.55845500	2.27240200	-0.05856600
H	-0.29228700	2.88402000	-0.38492400
H	0.43898100	2.08828700	1.01658800
H	1.47787500	2.83322400	-0.23959500

65	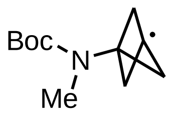	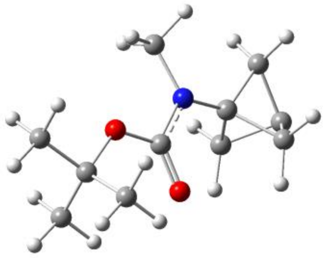

Cartesian Coordinates

C	-3.70752600	-0.69157600	0.11103800
C	-2.65427000	-1.07735200	-0.93608400
C	-2.09996200	0.05830200	-0.04398100
C	-3.44628000	0.79834500	-0.16674700
C	-2.63652100	-0.68565500	1.20919300
H	-2.14792100	-1.63229800	1.43879300
H	-2.83580300	-0.06946500	2.08875000
H	-3.70950600	1.16872800	-1.16011800
H	-3.69325200	1.50053200	0.63292500
H	-2.86723300	-0.80766400	-1.97252600
H	-2.16377200	-2.04278500	-0.82612400
N	-0.81229400	0.70263200	-0.18760900
C	0.28324600	-0.11389800	-0.08083800
O	0.21440900	-1.32741200	-0.03651900
O	1.42243600	0.60366800	-0.04455000
C	2.70833000	-0.07406900	0.01453000
C	2.82416700	-0.87508000	1.30837100
C	2.90904200	-0.94304100	-1.22353000
C	3.69727600	1.08640700	0.01680900
H	2.62122200	-0.22749300	2.16738800
H	2.12478100	-1.71126300	1.31729800
H	3.84360600	-1.26196000	1.40448900
H	3.93515500	-1.32425300	-1.23479600
H	2.21718100	-1.78548200	-1.23041400
H	2.75637600	-0.34541000	-2.12800900
H	3.59274700	1.67864300	-0.89701000
H	3.51791200	1.73720300	0.87754600
H	4.72101200	0.70554200	0.07221300
C	-0.73707800	2.14202500	0.00444200
H	0.19870100	2.51346900	-0.40838900
H	-1.57010900	2.60964800	-0.52446700
H	-0.79233200	2.41938500	1.06505700

66	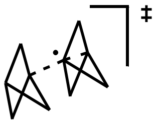	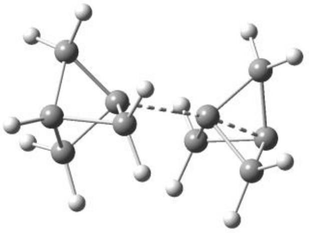

Cartesian Coordinates

C	-1.09471700	0.00278100	0.24555500
C	-2.27490200	0.21151400	1.21490200
C	-2.83247500	-0.00312900	-0.21051200
C	-1.75629000	0.96277100	-0.75619200
C	-1.84267600	-1.17455600	-0.40197300
H	-2.00685100	-2.05909400	0.21727600
H	-1.57316900	-1.42188700	-1.43144900
H	-1.84070800	2.00979000	-0.45691800
H	-1.48104000	0.84313800	-1.80689900
H	-2.39484900	1.20996200	1.64011200
H	-2.46606100	-0.58312700	1.93890100
C	1.30605100	0.00054200	0.15170800
C	2.03259000	1.27850900	-0.15320400
C	2.29958500	-0.50727000	1.15609500
C	2.85402300	0.00016200	-0.16450200
C	1.85670100	-0.77036800	-1.01337200
H	1.87069000	-1.85465700	-0.93404300
H	1.65194300	-0.38443400	-2.00939400
H	2.33402700	-1.58019000	1.32746500
H	2.48012800	0.10471100	2.03622400
H	2.20043800	1.96836900	0.66998600
H	1.83483300	1.74836100	-1.11379600
H	-3.89672100	-0.00669000	-0.48849800

67	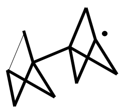	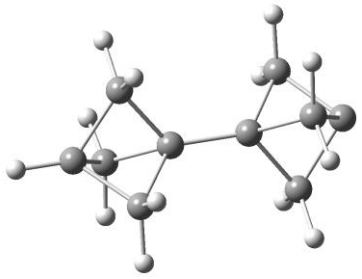

Cartesian Coordinates

C	0.68485500	0.00004900	0.00015400
C	1.61747200	0.16961900	-1.22466700
C	2.54585000	-0.00031300	0.00041600
C	1.61738400	0.97607500	0.75899300
C	1.61692600	-1.14576100	0.46541000
H	1.61182300	-2.05373400	-0.14538100
H	1.61172300	-1.37089700	1.53629400
H	1.61314600	2.01570700	0.41768400
H	1.61234700	0.90249600	1.85070300
H	1.61260200	1.15246800	-1.70580100
H	1.61251400	-0.64539600	-1.95489100
C	-0.81533700	0.00017800	-0.00007800
C	-1.72200500	1.16471000	-0.47344000
C	-1.72199700	-0.99247000	-0.77150700
C	-2.60599100	0.00011000	-0.00047500
C	-1.72239700	-0.17213800	1.24512400

H	-1.71349900	-1.15702600	1.71669200
H	-1.71363000	0.64780200	1.96627100
H	-1.71390300	-2.02675000	-0.42151800
H	-1.71308300	-0.90930800	-1.86019600
H	-1.71266800	1.37947600	-1.54406400
H	-1.71352500	2.06529800	0.14401400
H	3.63758300	-0.00049500	0.00061300

68

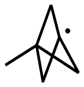

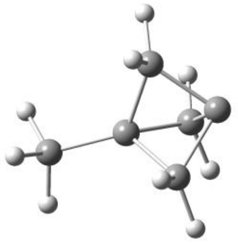

Cartesian Coordinates

C	-0.39526400	-0.00023200	0.00011000
C	0.51526700	1.22634800	-0.26550500
C	1.40020000	0.00042500	-0.00023900
C	0.51598800	-0.38317200	1.19448800
C	0.51590300	-0.84303600	-0.92906100
H	0.50815100	-0.57478900	-1.98784400
H	0.50937100	-1.92270400	-0.76385300
H	0.50872200	0.29965100	2.04692800
H	0.50927700	-1.43419800	1.49146200
H	0.50808800	2.00880600	0.49660200
H	0.50762900	1.62347100	-1.28300400
C	-1.91349300	-0.00027000	0.00006800
H	-2.29453900	-1.00192500	0.22055400
H	-2.29397300	0.69157700	0.75759900
H	-2.29432800	0.30973700	-0.97760800

69

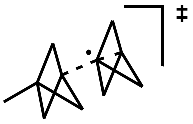

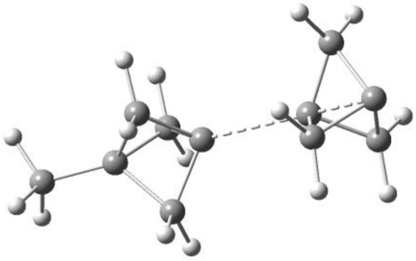

Cartesian Coordinates

C	-0.64231600	0.26926500	-0.05633200
C	-1.46074400	-0.46112700	-1.13115000
C	-2.42224200	-0.02569500	0.00389700
C	-1.37619100	-0.52682700	1.03222500
C	-1.72532600	1.35926000	0.01959200
H	-1.86577000	1.98270600	-0.86677200
H	-1.79464000	1.92608900	0.95110500
H	-1.20054200	-1.60548500	1.05971800
H	-1.42301400	-0.08060200	2.02888400
H	-1.29086000	-1.53574900	-1.23730600

H	-1.58361200	0.04392200	-2.09239700
C	1.77389800	0.09585500	-0.02519200
C	2.29423800	-1.31149700	-0.07661300
C	2.71552100	0.64705200	-1.05617500
C	3.32263900	-0.20005700	0.04928900
C	2.58355800	0.51670400	1.16649700
H	2.77965500	1.57894100	1.28863600
H	2.43266700	-0.03212200	2.09284800
H	2.91813700	1.71469400	-1.02978900
H	2.67850300	0.21051000	-2.05114600
H	2.23971800	-1.83076700	-1.03036800
H	2.13098400	-1.93698500	0.79788900
C	-3.91870100	-0.26987800	0.05480900
H	-4.41305900	0.20701900	-0.79706500
H	-4.13278600	-1.34265000	0.02438500
H	-4.34137500	0.14215300	0.97629700

70	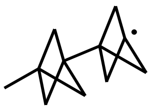	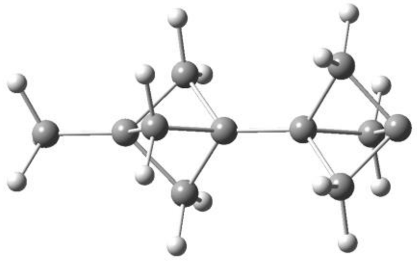

Cartesian Coordinates

C	-0.26426200	0.00005600	0.00070800
C	-1.19472900	-1.14429700	0.46565700
C	-2.13177000	-0.00002200	0.00006300
C	-1.19485500	0.97526900	0.75885900
C	-1.19418600	0.16945400	-1.22320600
H	-1.19174400	-0.64584900	-1.95420400
H	-1.19176400	1.15278900	-1.70487500
H	-1.19292400	0.90108200	1.85133600
H	-1.19258500	2.01589400	0.41806400
H	-1.19295000	-1.36973900	1.53716200
H	-1.19227600	-2.05313000	-0.14512100
C	1.23513800	0.00005400	0.00048600
C	2.14306900	-0.16627900	1.24579400
C	2.14160900	-0.99603400	-0.76714200
C	3.02623400	-0.00014000	-0.00088100
C	2.14201500	1.16212300	-0.47931400
H	2.13231200	1.37176900	-1.55091300
H	2.13390600	2.06575000	0.13371200
H	2.13186100	-0.91722000	-1.85623600
H	2.13282100	-2.02888700	-0.41275600
H	2.13451000	-1.14889400	1.72198800
H	2.13498200	0.65708200	1.96302400
C	-3.63687300	-0.00019000	-0.00074700
H	-4.02437000	-0.80578800	-0.63287800

H	-4.02545300	-0.14461600	1.01253200
H	-4.02465700	0.94979500	-0.38250200

81	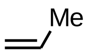	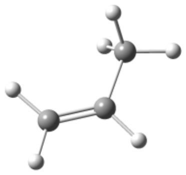

Cartesian Coordinates

C	0.00000000	0.48486000	0.00000000
C	-1.28638400	0.14969200	0.00000000
H	-1.60297200	-0.89126700	0.00000000
H	-2.07121500	0.89939200	0.00000000
H	0.26557800	1.54068700	0.00000000
C	1.13734200	-0.50703700	0.00000000
H	1.09746400	-1.15385900	0.88226600
H	1.09746400	-1.15385900	-0.88226600
H	2.10793700	-0.00618600	0.00000000

82	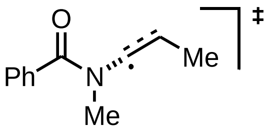	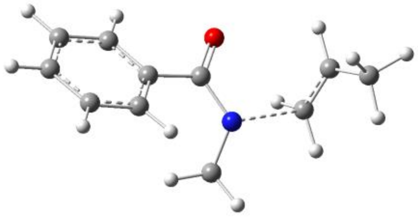

Cartesian Coordinates

C	-3.08736500	0.02698300	-0.77200500
N	-1.04694600	0.73441900	-0.30345500
C	-0.80341100	1.70035600	-1.35737700
H	-1.40490400	2.59454900	-1.17658600
H	0.25556000	1.99040100	-1.36506600
H	-1.04009000	1.30651600	-2.35618700
C	-0.26954100	-0.41370000	-0.30063100
C	1.19746100	-0.18648200	-0.01657500
C	1.67000700	0.95711100	0.63143800
C	2.09086800	-1.19055500	-0.39304100
C	3.03075100	1.09994300	0.88561900
H	0.96592800	1.71805500	0.95540500
C	3.45082000	-1.04162900	-0.14621200
H	1.69647300	-2.08105200	-0.87189400
C	3.92200700	0.10461400	0.49147300
H	3.39478000	1.98537400	1.39779500
H	4.14442400	-1.82064000	-0.44769500
H	4.98390200	0.21928800	0.68753900
O	-0.69786800	-1.54717100	-0.43044700
H	-3.41744400	1.05349000	-0.91277900
H	-2.68749800	-0.49574900	-1.63434400
C	-3.45173000	-0.66321300	0.33526400
H	-3.17815700	-1.71309900	0.39912500
C	-4.09092800	-0.03911300	1.53079100

H	-4.99804000	-0.57854900	1.82621800
H	-3.40714900	-0.07482700	2.38814600
H	-4.34986000	1.00679500	1.34561400

83	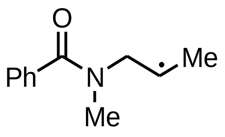	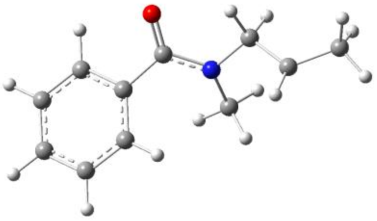

Cartesian Coordinates

C	-2.50114700	0.56340000	0.07685500
N	-1.14856400	0.04317900	0.25518400
C	-1.04550900	-1.09969900	1.15004100
H	-0.02689800	-1.20873500	1.51921700
H	-1.70996200	-0.94086000	2.00852400
H	-1.35087800	-2.02861200	0.65633800
C	-0.09591100	0.85439400	-0.09111900
C	1.28808700	0.26591600	-0.05484600
C	1.56253000	-1.03549600	-0.47939800
C	2.33809600	1.10860100	0.31223900
C	2.87513200	-1.49679500	-0.51439600
H	0.74869000	-1.67978800	-0.80136000
C	3.64731600	0.64110100	0.29604700
H	2.10795900	2.13077000	0.59562900
C	3.91693300	-0.66226100	-0.11680100
H	3.08483500	-2.50565000	-0.85631200
H	4.46024900	1.29580300	0.59444600
H	4.94026300	-1.02464700	-0.13798200
O	-0.25402000	2.00552000	-0.46937300
H	-2.88336600	0.94120300	1.03943400
H	-2.41778800	1.43054200	-0.58979700
C	-3.42444700	-0.47917800	-0.46108000
H	-3.07000900	-1.06070900	-1.30823500
C	-4.88733900	-0.41406600	-0.18642800
H	-5.08187900	-0.14620500	0.85871400
H	-5.39274200	0.34457700	-0.80550200
H	-5.37882000	-1.36960300	-0.39110900

84	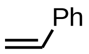	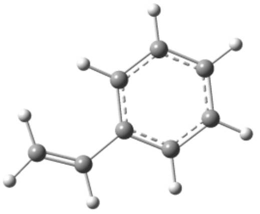

Cartesian Coordinates

C	-1.95250100	-0.53452000	-0.00004200
C	-2.96052000	0.33829900	0.00005600
H	-2.80568300	1.41315200	0.00016500

H	-3.99051000	-0.00121200	0.00003600
H	-2.18781900	-1.59826700	-0.00013400
C	-0.51099400	-0.22409900	-0.00002600
C	0.40735100	-1.27961000	-0.00000200
C	-0.01458300	1.08620800	-0.00003400
C	1.77818900	-1.04090800	0.00002800
H	0.03678200	-2.30186500	0.00000300
C	1.35280100	1.32769500	-0.00001400
H	-0.70308700	1.92586600	-0.00007000
C	2.25632700	0.26541000	0.00002100
H	2.47147900	-1.87650400	0.00005700
H	1.71762700	2.35045200	-0.00002100
H	3.32478300	0.45753700	0.00003900

85	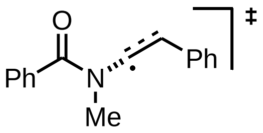	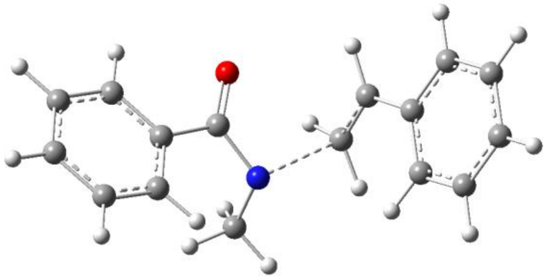

Cartesian Coordinates

C	-1.29153000	2.14787200	0.27472900
N	0.50629100	0.96058300	0.87804200
C	1.08207800	1.77856800	1.92924500
H	0.41475300	1.78405100	2.79438500
H	2.04834000	1.35604200	2.23959900
H	1.26695300	2.81399800	1.60900800
C	1.27510300	0.88626000	-0.28574600
C	2.47491100	-0.02098600	-0.18783800
C	2.61918900	-0.97306500	0.82430500
C	3.45789100	0.10474200	-1.17158200
C	3.74821900	-1.78667300	0.85478900
H	1.83543200	-1.08760300	1.56706900
C	4.58669700	-0.70472300	-1.13504000
H	3.31344100	0.84099800	-1.95544600
C	4.73317800	-1.65007400	-0.12096600
H	3.85624200	-2.53289100	1.63581700
H	5.35248400	-0.60159900	-1.89756300
H	5.61377400	-2.28487600	-0.09460000
O	1.00604000	1.45083000	-1.32892300
H	-1.60047500	2.13953300	1.31559800
H	-0.65452500	2.96317700	-0.04909500
C	-1.91898500	1.37973300	-0.65164500
H	-1.67879800	1.53792700	-1.70025900
C	-2.85233500	0.29943400	-0.34884300
C	-3.67570200	-0.21052000	-1.36335700
C	-2.94401100	-0.26395500	0.93401100
C	-4.58730000	-1.22534700	-1.09992600

H	-3.59842800	0.20659700	-2.36423300
C	-3.85272100	-1.28033400	1.19581300
H	-2.27146200	0.07959200	1.71452000
C	-4.68226800	-1.76072100	0.18272700
H	-5.22277900	-1.60185200	-1.89570200
H	-3.91009000	-1.70908900	2.19184900
H	-5.39170200	-2.55597900	0.39008400

86	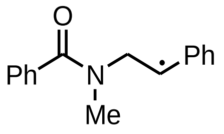	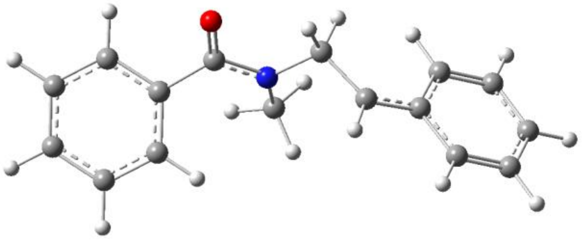

Cartesian Coordinates

C	-0.74201600	1.35815200	-0.28200200
N	0.43804900	0.56372300	0.06730000
C	0.19351300	-0.51893500	1.00750300
H	1.11981400	-0.82276700	1.49289000
H	-0.50346000	-0.16916000	1.77900100
H	-0.26037000	-1.38778600	0.51756600
C	1.67383300	1.06635300	-0.25558200
C	2.87207000	0.18008300	-0.05195400
C	2.86106200	-1.18394600	-0.35031700
C	4.06348800	0.79106900	0.34165800
C	4.02724900	-1.93415900	-0.23211000
H	1.94394700	-1.65347100	-0.69594300
C	5.22345300	0.03748500	0.47823200
H	4.06209900	1.86075100	0.52542600
C	5.20619200	-1.32648200	0.19258700
H	4.01658200	-2.99179300	-0.47686100
H	6.14497500	0.51472700	0.79713500
H	6.11451800	-1.91352100	0.28971600
O	1.82000500	2.18061100	-0.73625900
H	-1.13011100	1.84625200	0.62064900
H	-0.39490500	2.14638600	-0.95473100
C	-1.77654100	0.50686500	-0.94376700
H	-1.50451500	0.10918800	-1.91880600
C	-3.03423700	0.12520700	-0.41086100
C	-3.50398400	0.52946200	0.86537900
C	-3.88544200	-0.71202300	-1.17925200
C	-4.74154200	0.11788600	1.33239200
H	-2.89153100	1.17163400	1.49007800
C	-5.11895200	-1.11772400	-0.70429900
H	-3.54823400	-1.03375200	-2.16117000
C	-5.55896100	-0.70696600	0.55692900
H	-5.07687700	0.44278900	2.31284500
H	-5.74684900	-1.75831700	-1.31627100

H	-6.52657500	-1.02607700	0.93021700

99

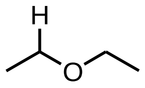

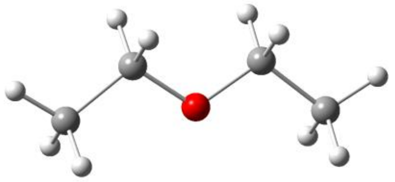

Cartesian Coordinates

C	1.17145700	0.51999900	-0.00015700
H	1.18910800	1.17469900	-0.88706700
H	1.18884600	1.17478700	0.88669900
O	-0.00000400	-0.26107300	-0.00032500
C	-1.17146000	0.52000600	0.00019300
H	-1.18877800	1.17444800	0.88729600
H	-1.18918500	1.17505500	-0.88647000
C	-2.36603700	-0.41279300	0.00003700
H	-2.34436400	-1.05186100	-0.88632500
H	-2.34411700	-1.05260100	0.88584600
H	-3.29993600	0.15598000	0.00037800
C	2.36604200	-0.41279000	0.00016400
H	2.34428600	-1.05255900	-0.88567500
H	3.29993300	0.15599800	0.00000000
H	2.34422100	-1.05189500	0.88649900

100

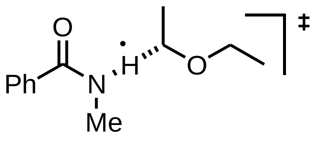

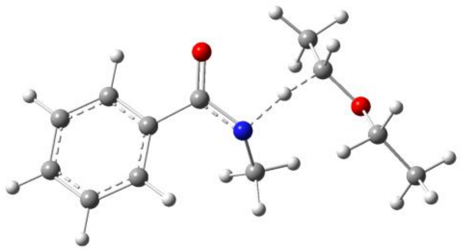

Cartesian Coordinates

N	-0.38534700	-0.31122400	-0.45434000
C	-0.54468600	-1.16194600	0.71076000
H	0.24402600	-1.02285000	1.45787100
H	-1.51036400	-0.92492100	1.18219100
H	-0.57681300	-2.21681800	0.41433300
C	0.64359400	0.60617400	-0.53935900
C	2.02961300	0.10205600	-0.22810300
C	2.38205600	-1.24507900	-0.32962100
C	2.99483500	1.04743600	0.12281200
C	3.68917200	-1.64434200	-0.06784300
H	1.63900900	-1.97549500	-0.63698000
C	4.29643000	0.64516400	0.39800800
H	2.70354200	2.09196700	0.16607400
C	4.64436000	-0.70123300	0.30334400
H	3.96315000	-2.69096900	-0.15786600
H	5.04297700	1.38130400	0.68024200
H	5.66281000	-1.01435500	0.51295300
O	0.47330700	1.75409300	-0.91820600

H	-1.52844100	0.54318000	-0.49889100
C	-2.53914600	1.15511500	-0.18878200
H	-2.90613800	1.54459700	-1.15014900
C	-2.16823600	2.21355800	0.81376400
H	-3.05361400	2.78205100	1.11736900
H	-1.73741400	1.75013800	1.70729100
H	-1.42977100	2.88809700	0.37748900
O	-3.39931600	0.22176100	0.36582300
C	-3.82377000	-0.76669100	-0.56096400
H	-4.39274800	-0.28508500	-1.36960000
H	-2.94058200	-1.24249700	-1.01307600
C	-4.67329400	-1.77554700	0.18236000
H	-5.04842000	-2.53861900	-0.50468800
H	-4.08604200	-2.26697500	0.96298100
H	-5.52522500	-1.27899500	0.65364300

101

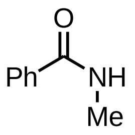

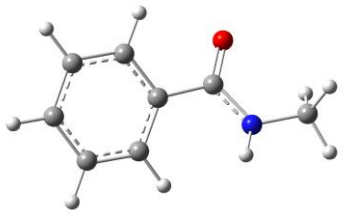

Cartesian Coordinates

N	-2.04620700	-0.69299400	-0.14899600
C	-1.23167600	0.37097300	0.11394600
C	-3.48116200	-0.49411900	-0.20205400
C	0.24509200	0.10239100	0.05249800
C	1.08469500	1.19943800	-0.14326400
C	0.79627900	-1.17193500	0.20361800
C	2.46111100	1.02175500	-0.21456600
H	0.63348600	2.18227100	-0.23340700
C	2.17537800	-1.34810700	0.13914200
H	0.15594200	-2.02524500	0.41088200
C	3.00788600	-0.25303700	-0.07670900
H	3.11049000	1.87688800	-0.37501000
H	2.60038200	-2.33881600	0.26708100
H	4.08348700	-0.39209100	-0.12894100
O	-1.67530300	1.47367500	0.38940800
H	-3.97964900	-1.46423300	-0.16778200
H	-3.78163200	0.09910300	0.66223000
H	-3.78930100	0.04334400	-1.10632100
H	-1.65296400	-1.48382500	-0.63668400

102

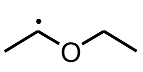

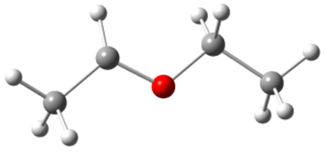

Cartesian Coordinates

C	1.18010300	0.48481800	-0.07766000

O	0.03006500	-0.23948700	-0.00866600
C	-1.15636000	0.53553600	-0.03172500
H	-1.15836600	1.22360300	0.82695000
H	-1.17810400	1.14237700	-0.94684600
C	-2.33851000	-0.40895000	0.02763500
H	-2.32510700	-1.08500600	-0.83083400
H	-2.29923500	-1.00907600	0.94021300
H	-3.27665500	0.15244000	0.01708000
C	2.41856500	-0.33339800	0.01378900
H	2.38923200	-1.14917400	-0.71599400
H	3.29831800	0.28216900	-0.19070900
H	2.54737500	-0.78925000	1.00742400
H	1.13924000	1.47977600	0.36980700
114	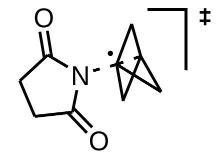	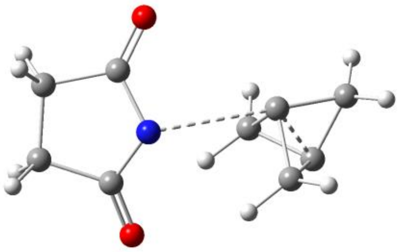	
Cartesian Coordinates			
C	1.90010200	0.49672600	0.22092400
C	2.11265100	-0.85209200	0.88896300
C	2.82896600	-0.58602400	-0.40148000
C	1.64201300	-0.08222400	-1.16363500
C	3.36995000	0.76114900	-0.02622400
H	4.01585100	0.81584300	0.84588800
H	3.62365200	1.44249300	-0.83368900
H	0.81444800	-0.77629900	-1.27867400
H	1.82879700	0.57232700	-2.01071200
H	1.28523100	-1.55633900	0.86209800
H	2.70834400	-0.85763700	1.79779500
N	-0.83544500	0.00515700	0.67979400
C	-1.43092300	1.19175600	0.21221200
C	-1.42526200	-1.11729700	0.09326300
C	-2.87128600	0.81226100	-0.16777600
C	-2.86686800	-0.72307800	-0.24542200
H	-3.10793200	1.30153600	-1.11510500
H	-3.10240400	-1.11974200	-1.23552000
H	-3.54080500	-1.19619400	0.47310200
H	-3.55070600	1.20689000	0.59139200
O	-0.90250700	-2.18936400	-0.07777600
O	-0.93279500	2.27985900	0.16276500

115	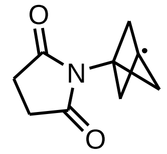	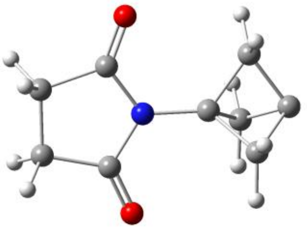

Cartesian Coordinates

C	1.07429200	0.02556400	-0.00010600
C	1.96755400	-0.60512200	1.09154200
C	2.84823800	0.02509200	-0.00009500
C	1.96752400	-0.61026600	-1.08868700
C	1.96090000	1.28293200	-0.00290900
H	1.94249100	1.88620300	0.90466300
H	1.94243800	1.88198700	-0.91327300
H	1.94626900	-1.69847500	-1.14540900
H	1.95594200	-0.11797000	-2.06275200
H	1.94626300	-1.69303400	1.15344000
H	1.95603900	-0.10802700	2.06315900
N	-0.37175900	0.02107500	-0.00058600
C	-1.17652100	1.15968600	0.00007500
C	-1.08723900	-1.17256100	0.00000500
C	-2.62969400	0.69708000	0.00545100
C	-2.57133300	-0.83249600	-0.00571400
H	-3.12999100	1.12356600	-0.86716600
H	-3.02469100	-1.28006400	-0.89356600
H	-3.03767300	-1.29597300	0.86687000
H	-3.11644400	1.10889500	0.89287000
O	-0.58405000	-2.26865200	0.00294400
O	-0.77853200	2.29689200	-0.00195800

118	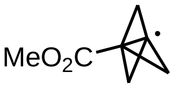	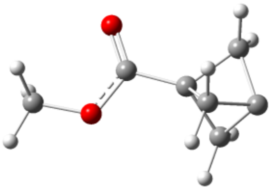

Cartesian Coordinates

C	2.39595200	-0.34268600	0.00322400
C	1.40271900	-0.69906800	1.12173700
C	0.66215700	0.06894500	-0.00318900
C	1.81527200	1.08290100	-0.06890200
C	1.38083400	-0.81882500	-1.04979400
H	1.11254700	-1.87592000	-1.04583000
H	1.47004300	-0.39767700	-2.05246100
H	1.94601000	1.72003100	0.80666700
H	1.92960300	1.62026800	-1.01114700
H	1.50990900	-0.17087900	2.07050000
H	1.13586200	-1.75021200	1.23852100
C	-0.80612200	0.41557100	-0.00312800

O	-1.25399200	1.53281500	-0.00017700
O	-1.55566300	-0.69789300	-0.00254600
C	-2.96495300	-0.46755500	0.00147500
H	-3.42721800	-1.45339400	-0.00843400
H	-3.25650500	0.08701600	0.89612400
H	-3.25816800	0.10569100	-0.88069300

119

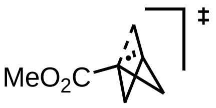

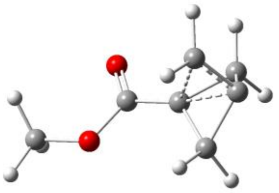

Cartesian Coordinates

C	-2.35613800	-0.27911100	0.02340700
C	-1.29816100	-1.10140000	0.78005100
C	-0.55320300	0.10645700	0.23593600
C	-1.92181800	-0.37266500	-1.30443800
C	-1.71991900	1.04179200	0.49230500
H	-1.87397800	1.29195600	1.54639800
H	-1.83140200	1.91217100	-0.15527000
H	-1.67514500	-1.33567900	-1.74931200
H	-2.02908300	0.46402400	-1.99307000
H	-1.04795500	-2.08521500	0.38125500
H	-1.40090600	-1.11401000	1.86953000
C	0.87373200	0.41156300	0.07984200
O	1.32730600	1.51668400	-0.12907600
O	1.62144800	-0.71545500	0.09620300
C	3.00867900	-0.50151500	-0.14458700
H	3.42927600	0.17433500	0.60381800
H	3.47703700	-1.48297300	-0.08074000
H	3.16309100	-0.06517200	-1.13471900

120

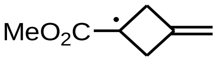

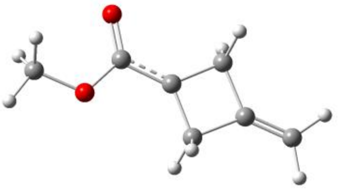

Cartesian Coordinates

C	2.31808600	-0.13143600	-0.00018600
C	1.12540800	-1.08943400	0.00116200
C	0.26037000	0.13873100	0.00157400
C	1.41137300	1.10069400	0.00103600
H	1.47904200	1.74431800	-0.88558400
H	1.48067900	1.74312200	0.88842400
H	1.02921200	-1.72841500	0.88872300
H	1.02733600	-1.72914600	-0.88565700
C	3.63003600	-0.30285000	-0.00176900
H	4.30960900	0.54431900	-0.00254800
H	4.07086600	-1.29550100	-0.00249700

C	-1.16039800	0.37301500	0.00018800
O	-1.67641500	1.47496800	-0.00077900
O	-1.86571700	-0.78232500	0.00013000
C	-3.27867200	-0.60613500	-0.00077600
H	-3.59585800	-0.05510600	-0.88962200
H	-3.70451200	-1.60872300	0.00102200
H	-3.59653700	-0.05152200	0.88554900

121

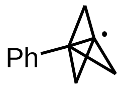

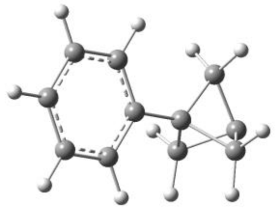

Cartesian Coordinates

C	2.94928600	-0.00383900	-0.00053700
C	2.06467300	-0.62870200	-1.08716700
C	1.15354600	0.00819500	0.00032700
C	2.06558400	-0.62976900	1.08627600
C	2.06941900	1.25595900	0.00056100
H	2.06692000	1.85907000	-0.90994300
H	2.06771300	1.85818800	0.91165100
H	2.05149900	-1.72002000	1.14906000
H	2.05387200	-0.14547100	2.06449900
H	2.05042100	-1.71889500	-1.15101800
H	2.05192500	-0.14341600	-2.06488300
C	-0.34855900	0.01016300	0.00032100
C	-1.04711100	-1.19949500	0.00024500
C	-1.06035900	1.20831100	0.00019900
C	-2.43712500	-1.20924400	0.00001700
H	-0.49317100	-2.13539800	0.00033600
C	-2.45362100	1.20031300	-0.00004000
H	-0.51737000	2.14996500	0.00031000
C	-3.14427000	-0.00753900	-0.00013000
H	-2.97156800	-2.15458300	-0.00004500
H	-2.99913200	2.13928200	-0.00012200
H	-4.22989100	-0.01484000	-0.00027500

122

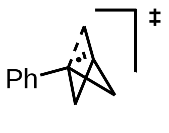

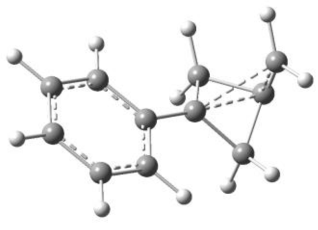

Cartesian Coordinates

C	2.92751500	-0.00009000	-0.04099300
C	2.01657100	-1.09470900	-0.61935800
C	1.04263600	-0.00002700	-0.19634500
C	2.57527900	0.00072000	1.31682700

C	2.01659800	1.09394200	-0.62055300
H	2.07887700	1.23809700	-1.70554900
H	1.99909800	2.05017500	-0.09182000
H	2.55189300	-0.92304300	1.89305300
H	2.55201800	0.92515200	1.89198800
H	1.99894100	-2.05035000	-0.08956100
H	2.07885700	-1.24000600	-1.70419600
C	-0.40853800	0.00000700	-0.11236200
C	-1.12236900	-1.20903700	-0.04634300
C	-1.12234700	1.20906700	-0.04638300
C	-2.50512100	-1.20594600	0.07502100
H	-0.57849500	-2.14851400	-0.09786600
C	-2.50509200	1.20600900	0.07498700
H	-0.57844900	2.14852700	-0.09792800
C	-3.20340200	0.00003800	0.13757000
H	-3.04429500	-2.14774100	0.11793100
H	-3.04424600	2.14781600	0.11787900
H	-4.28456700	0.00005200	0.23366900

123	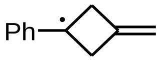	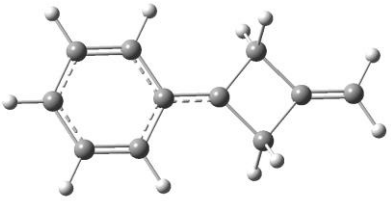

Cartesian Coordinates

C	-2.87661800	0.00000200	0.00001000
C	-1.82117100	1.10356700	-0.00012100
C	-0.79164500	0.00001300	-0.00031000
C	-1.82115900	-1.10355200	-0.00041500
H	-1.82495000	-1.75100700	-0.88883100
H	-1.82470600	-1.75168900	0.88750500
H	-1.82469300	1.75129500	0.88809400
H	-1.82499900	1.75143400	-0.88823900
C	-4.20015200	-0.00001500	0.00052200
H	-4.76587000	-0.92724800	0.00061800
H	-4.76590100	0.92719900	0.00087400
C	0.61694100	0.00000600	-0.00015900
C	1.34640300	-1.21457900	0.00003300
C	1.34641700	1.21458300	-0.00012200
C	2.73068600	-1.20808700	0.00020400
H	0.80222100	-2.15552700	0.00002400
C	2.73070100	1.20807700	0.00005200
H	0.80224500	2.15553800	-0.00022700
C	3.43317000	-0.00000900	0.00020900
H	3.27340400	-2.14880800	0.00037600
H	3.27342800	2.14879100	0.00002900
H	4.51837200	-0.00001800	0.00034900

124

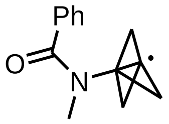

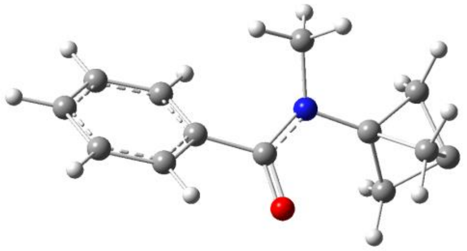

Cartesian Coordinates

C	3.93223900	-0.24492800	-0.26576700
C	3.08718400	-0.76344600	0.90646300
C	2.20488500	0.11044000	-0.02622000
C	3.33458300	1.16068100	-0.09236200
C	2.81264100	-0.60617300	-1.25218900
H	2.55423200	-1.65760300	-1.36031400
H	2.78384300	-0.04762100	-2.18995600
H	3.57175600	1.68592800	0.83557500
H	3.34029100	1.81233800	-0.96863100
H	3.30982800	-0.35125600	1.89344600
H	2.84502900	-1.82568200	0.92009600
N	0.80827200	0.45124000	0.15077600
C	0.56868100	1.58915200	1.02935100
C	-0.09870900	-0.57058100	-0.03740900
H	-0.48085900	1.63262800	1.31493400
H	1.16891300	1.48997200	1.94234100
H	0.84477700	2.52524400	0.53331100
O	0.25660700	-1.71590800	-0.26346900
C	-1.55991300	-0.21834700	-0.03096800
C	-2.45548400	-1.18157300	0.43535400
C	-2.04461300	0.97798400	-0.56460700
C	-3.82339300	-0.93512100	0.40497300
H	-2.06056700	-2.12103200	0.80882600
C	-3.41465500	1.21688100	-0.60967900
H	-1.34699600	1.71014600	-0.96271000
C	-4.30390100	0.26484200	-0.11602800
H	-4.51686000	-1.68135600	0.77994800
H	-3.78886900	2.14336200	-1.03404600
H	-5.37254400	0.45463400	-0.14596700

125

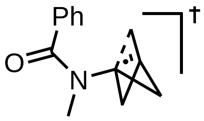

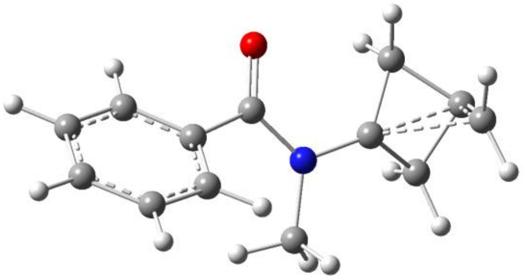

Cartesian Coordinates

C	3.96080900	-0.18645400	-0.27158900
C	3.32821200	0.53929000	0.92682000

C	2.12915500	0.04843800	0.12739700
C	3.60419900	0.63214700	-1.35499600
C	2.84146400	-1.23885600	-0.24635600
H	2.88270000	-1.96579500	0.57250000
H	2.57903800	-1.72049900	-1.18758600
H	3.79632200	1.70413000	-1.33595100
H	3.38422100	0.20217000	-2.33085500
H	3.51239500	1.61398400	1.00465300
H	3.44433100	0.04013500	1.89794900
N	0.77672400	0.26117300	0.43994000
C	0.47838900	1.51545800	1.12088400
C	-0.18186800	-0.67474900	0.06310600
H	-0.54578300	1.51007800	1.48852300
H	1.15537000	1.62057400	1.97305100
H	0.62276000	2.37460100	0.45553100
O	0.12140200	-1.81836000	-0.22457600
C	-1.61628600	-0.23587800	-0.00511200
C	-2.58855900	-1.15261200	0.39890300
C	-2.00590800	0.98363300	-0.56335800
C	-3.93715400	-0.83527500	0.28736000
H	-2.26699800	-2.11313100	0.78904900
C	-3.35646800	1.29167900	-0.69380800
H	-1.25362500	1.68241500	-0.91914900
C	-4.32246300	0.38784100	-0.25828000
H	-4.68906700	-1.54547900	0.61704800
H	-3.65505200	2.23514200	-1.14020800
H	-5.37603300	0.63237600	-0.35334300

126

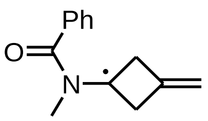

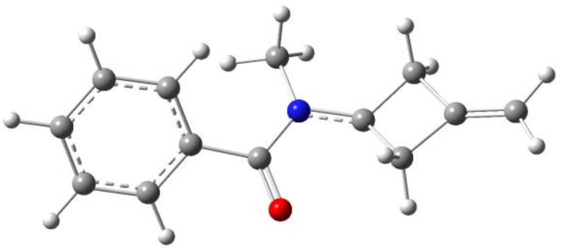

Cartesian Coordinates

C	-3.90813600	-0.09770000	-0.31506800
C	-3.13728900	1.01764400	0.39203800
C	-1.96887300	0.06345700	0.37851500
C	-2.67724700	-0.99284600	-0.42399700
H	-2.29974700	-1.12076500	-1.44931000
H	-2.74401500	-1.98630600	0.03186800
H	-3.52678300	1.32074800	1.37375000
H	-3.01574800	1.92677300	-0.21831100
C	-5.17435100	-0.22634900	-0.67987000
H	-5.52810400	-1.12356000	-1.17954300
H	-5.90196300	0.55839100	-0.49310200
N	-0.62536400	0.38233900	0.42278300
C	-0.29032100	1.70457500	0.94630600

C	0.30669900	-0.62701000	0.21748400
H	-0.93868000	1.91762100	1.80064400
H	0.74477000	1.72520800	1.28146100
H	-0.44054300	2.48327900	0.19127700
O	-0.03706900	-1.79808000	0.16300300
C	1.74234900	-0.24094100	0.02384400
C	2.71277100	-1.12031000	0.50955700
C	2.13636500	0.88253800	-0.70762700
C	4.06187200	-0.85602600	0.30525800
H	2.38778000	-2.01084800	1.03816300
C	3.48684600	1.13821500	-0.92488900
H	1.38547900	1.54618800	-1.12761600
C	4.45108100	0.27532500	-0.40994800
H	4.81111100	-1.53717200	0.69650200
H	3.78521600	2.00726800	-1.50296900
H	5.50472700	0.47800800	-0.57593200

9.3 Electronic Properties of Radicals

DFT Method: UB3LYP/6-311+G(d,p) [LanL2DZ basis set for I atom]

Radical	Ionization Potential (IP, eV)	Electron affinity (EA, eV)	Electronegativity (χ, eV)	Electronic Chemical Potential (μ, eV)	Chemical Hardness (η, eV)	Chemical Softness (S, meV)	Global Electrophilicity Index (ω, eV)	Local Electrophilicity Index (ω^{+}_{rc}, eV)	Hirshfeld Charge
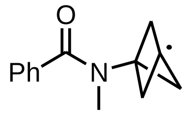	7.73	0.42	4.07	-4.07	7.30	136.89	1.14	0.555	-0.034784
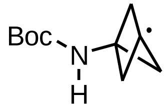	8.01	0.35	4.18	-4.18	7.66	130.51	1.14	0.62	-0.029492
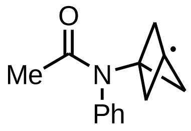	7.60	0.47	4.04	-4.04	7.13	140.26	1.14	0.54	-0.035912
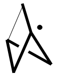	7.67	-0.18	3.75	-3.75	7.85	127.40	0.89	0.44	-0.051618
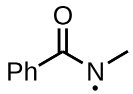	9.44	2.10	5.77	-5.77	7.34	136.17	2.27	1.02	-0.103162

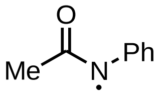	8.27	2.160	5.21	-5.21	6.11	163.70	2.23	0.68	-0.11877
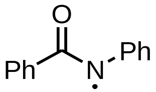	8.05	2.36	5.20	-5.20	5.69	175.68	2.38	0.67	-0.111484
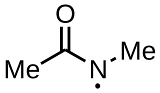	10.36	1.69	6.03	-6.03	8.67	115.36	2.10	1.09	-0.111029
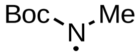	9.88	1.31	5.60	-5.60	8.56	116.80	1.83	1.03	-0.082878
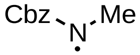	9.21	1.93	5.57	-5.57	7.28	137.38	2.13	1.07	-0.074683
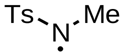	9.81	2.05	5.93	-5.93	7.76	128.81	2.27	1.31	-0.083927
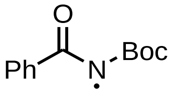	9.52	2.93	6.23	-6.23	6.59	151.77	2.94	1.22	-0.066946
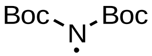	9.81	2.13	5.97	-5.97	7.68	130.18	2.32	1.13	-0.072858
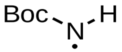	10.67	1.67	6.17	-6.17	9.00	111.15	2.12	1.35	-0.006485
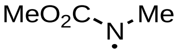	10.33	1.48	5.91	-5.91	8.84	113.10	1.97	1.11	-0.076238

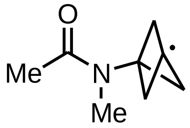	7.80	0.27	4.04	-4.04	7.52	132.97	1.08	0.60	-0.035286
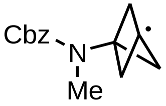	7.63	0.38	4.00	-4.00	7.26	137.83	1.10	0.58	-0.034615
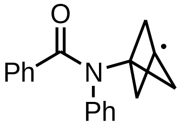	7.53	0.60	4.07	-4.07	6.93	144.33	1.19	0.52	-0.03425
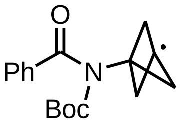	7.64	0.43	4.04	-4.04	7.20	138.79	1.13	0.56	-0.035458
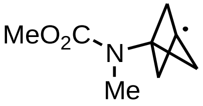	7.96	0.31	4.13	-4.13	7.65	130.65	1.12	0.63	-0.034323
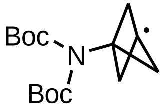	7.80	0.41	4.10	-4.10	7.39	135.31	1.14	0.58	-0.033722
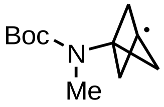	7.83	0.27	4.05	-4.05	7.56	132.28	1.09	0.60	-0.035934

Computed Energies [values are in Hartree]

Species	Total Electronic Energy	Sum of Electronic and Zero-point Energies	Sum of Electronic and Thermal Enthalpies	Gibbs Free Energy
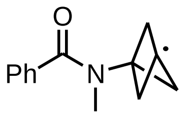	-633.8015793	-633.562613	-633.548549	-633.603965
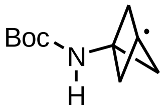	-595.9419038	-595.695639	-595.680869	-595.737513
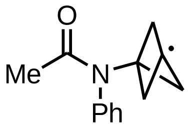	-633.8066312	-633.568816	-633.554503	-633.611353
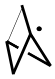	-194.6342322	-194.531089	-194.526125	-194.558151
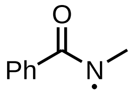	-439.712049	-439.571464	-439.561377	-439.607432
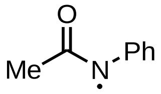	-439.7295417	-439.589257	-439.579192	-439.625077
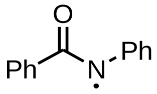	-631.5139352	-631.319966	-631.307158	-631.360470
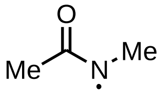	-247.9273344	-247.840527	-247.833082	-247.871641
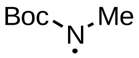	-441.1567648	-440.980737	-440.968628	-441.017954
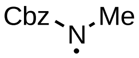	-479.0314515	-478.863183	-478.851602	-478.902603

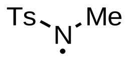	-914.3069941	-914.139235	-914.125607	-914.181161
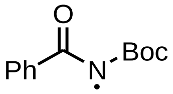	-746.3104095	-746.071505	-746.054652	-746.117349
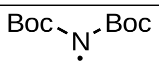	-747.7550322	-747.480848	-747.461870	-747.529131
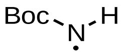	-401.8312772	-401.683279	-401.672815	-401.717566
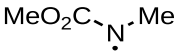	-323.1746454	-323.082030	-323.073766	-323.114430
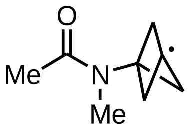	-442.0226401	-441.836824	-441.825720	-441.873257
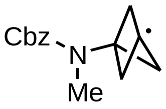	-673.1250576	-672.858246	-672.842669	-672.903253
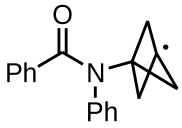	-825.5843383	-825.293227	-825.276121	-825.339210
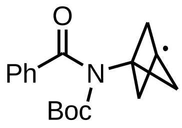	-827.0352179	-826.709025	-826.689684	-826.758099
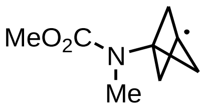	-517.2726559	-517.081595	-517.069359	-517.120224
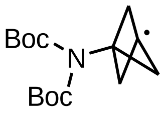	-941.8521657	-941.480274	-941.457254	-941.532576
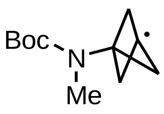	-635.2534759	-634.979263	-634.963081	-635.022721

Optimized Structures and Cartesian Coordinates

No.	Species	Optimized Structure
3	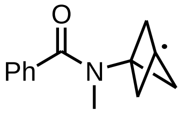	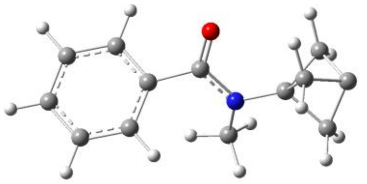

Cartesian Coordinates

C	-3.97379000	-0.19869200	0.26903000
C	-3.13792100	-0.83266800	-0.86431700
C	-2.22153900	0.10491200	-0.00330000
C	-3.34573600	1.18294800	-0.02259200
C	-2.85291500	-0.50065900	1.28723300
H	-2.62192400	-1.54245900	1.48709700
H	-2.81442600	0.12907500	2.17655200
H	-3.58010600	1.63513500	-0.98643700
H	-3.34225300	1.90457400	0.79499200
H	-3.35676500	-0.50136200	-1.87997300
H	-2.92644600	-1.89705800	-0.79408700
N	-0.81129800	0.40891700	-0.20915800
C	-0.52922200	1.54515300	-1.08830400
H	0.53279200	1.59113900	-1.31163600
H	-1.07176700	1.43719900	-2.03338100
H	-0.83294100	2.48496400	-0.61991400
C	0.10542500	-0.57873800	0.08751600
C	1.56873900	-0.22216500	0.04849700
C	2.07237600	0.92612800	0.66903800
C	2.45739100	-1.13371700	-0.53054700
C	3.44485400	1.16401000	0.69965900
H	1.39278300	1.62339100	1.14619300
C	3.82648900	-0.88299300	-0.52033200
H	2.06488200	-2.03973500	-0.97654800
C	4.32328800	0.26538400	0.09607000
H	3.82775600	2.04799400	1.19714500
H	4.50718400	-1.58859400	-0.98314600
H	5.39070200	0.45461000	0.11376600
O	-0.23937700	-1.70433800	0.42094800

No.	Species	Optimized Structure
4	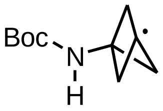	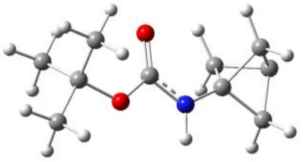

Cartesian Coordinates

C	3.90522800	0.28485100	-0.00007500
C	2.87022700	0.61725300	-1.09676100
C	2.19244900	-0.26839500	0.00003000

C	3.43856500	-1.19264000	-0.00064600
C	2.87073900	0.61625900	1.09737200
H	2.52320600	1.64471400	1.16297200
H	3.01486500	0.14612700	2.07049500
H	3.61908200	-1.76622000	-0.91049200
H	3.61948100	-1.76703900	0.90860700
H	3.01393500	0.14801800	-2.07037900
H	2.52264000	1.64575400	-1.16126700
N	0.84323900	-0.76978500	0.00005700
C	-0.24839900	0.05448500	-0.00001300
O	-0.19107100	1.26674700	0.00001100
H	0.68755600	-1.76551000	0.00006100
O	-1.37657400	-0.69648300	-0.00012400
C	-2.71631900	-0.07357000	0.00000100
C	-3.64454200	-1.28852400	0.00002500
C	-2.90989600	0.75577900	-1.27217000
C	-2.90976600	0.75568500	1.27225500
H	-4.68712000	-0.96160200	0.00006500
H	-3.47383600	-1.90326200	-0.88652100
H	-3.47377100	-1.90325000	0.88656800
H	-3.94345500	1.10978300	-1.32069900
H	-2.24492900	1.61794200	-1.28957900
H	-2.72046100	0.14318600	-2.15758300
H	-3.94333800	1.10964300	1.32093500
H	-2.72019000	0.14307100	2.15761900
H	-2.24489400	1.61792300	1.28959300

5

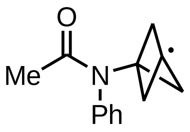

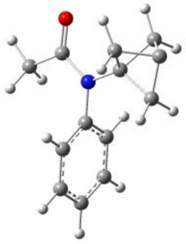

Cartesian Coordinates

C	-2.69090000	-1.86398000	0.00004400
C	-2.54957700	-0.78782800	1.09768900
C	-1.45999400	-0.55596700	0.00000100
C	-1.15862100	-2.07816100	0.00011100
C	-2.54949100	-0.78797000	-1.09773900
H	-3.32664900	-0.03143100	-1.16237600
H	-2.18670700	-1.12380700	-2.06970700
H	-0.71677900	-2.48832400	0.90799400
H	-0.71670200	-2.48845400	-0.90767500
H	-2.18685800	-1.12353900	2.06972600
H	-3.32673400	-0.03127700	1.16217500
N	-0.42937500	0.48748100	-0.00004800
C	-0.83957600	1.80850200	-0.00003600
C	0.24891400	2.86758000	-0.00001700
O	-2.02419800	2.10305800	-0.00002400

H	-0.23771400	3.84088600	-0.00014000
H	0.89064200	2.78034500	0.87974300
H	0.89084900	2.78019900	-0.87961000
C	0.94997400	0.08739100	-0.00003000
C	1.62047100	-0.12181500	-1.20731700
C	1.62045200	-0.12171800	1.20728700
C	2.95382800	-0.52650900	-1.20631000
H	1.09226200	0.03666600	-2.14067600
C	2.95381000	-0.52639800	1.20634500
H	1.09221200	0.03683900	2.14061800
C	3.62289700	-0.72869900	0.00003000
H	3.46904200	-0.68418700	-2.14708200
H	3.46900800	-0.68399100	2.14713900
H	4.66021400	-1.04333200	0.00004900

6

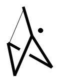

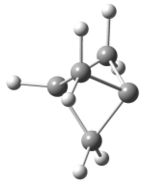

Cartesian Coordinates

C	0.00012500	0.00015300	-0.94996400
C	-0.20396900	-1.24813400	-0.05713000
C	0.00046700	0.00066800	0.85082800
C	1.18341100	0.44742200	-0.05724900
C	-0.97978100	0.80025800	-0.05723900
H	-2.02124400	0.47855000	-0.05407400
H	-0.87373800	1.88503300	-0.05471600
H	2.06894200	-0.18809500	-0.05459200
H	1.42717000	1.50970100	-0.05472900
H	0.59441200	-1.99010700	-0.05423600
H	-1.19709700	-1.69733000	-0.05390500
H	0.00003800	0.00004800	1.95077200

7

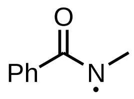

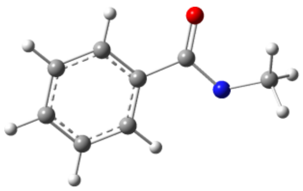

Cartesian Coordinates

C	3.39499200	0.59977900	-0.36719100
N	2.07324400	0.73288200	0.17725300
C	1.25537200	-0.38734000	0.15593500
C	-0.20850600	-0.12531000	0.08090000
C	-1.07675000	-1.21101900	-0.09194400
C	-0.72845300	1.17328700	0.16093600
C	-2.44708600	-0.99967100	-0.18600500
H	-0.65924900	-2.20893200	-0.14949000
C	-2.10200800	1.38039800	0.06671300
H	-0.05325800	2.00591900	0.31158700

C	-2.96153600	0.29636200	-0.10715800
H	-3.11641300	-1.84167200	-0.32085100
H	-2.50227900	2.38549300	0.13499200
H	-4.03120000	0.45925400	-0.17898100
O	1.73022000	-1.51446900	0.27430500
H	3.59526400	-0.37801400	-0.81301100
H	3.52725400	1.39572800	-1.11282500
H	4.12926800	0.80890400	0.42024900

8	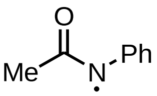	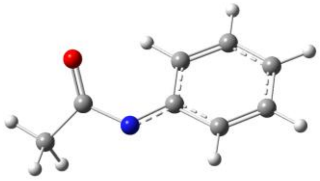

Cartesian Coordinates

N	1.03107500	0.79547500	0.13513200
C	2.13569100	-0.05042100	0.16571600
C	3.26216000	0.39018100	-0.74251100
O	2.22815300	-0.99338100	0.93343000
C	-0.23203700	0.35385200	0.05653900
C	-1.27121800	1.32412400	0.21066800
C	-0.61169600	-1.00233600	-0.20561100
C	-2.59908500	0.96161100	0.11068800
H	-0.97417100	2.34678800	0.40842700
C	-1.94588000	-1.34331400	-0.30404000
H	0.15838900	-1.75508900	-0.30640400
C	-2.94636800	-0.37215100	-0.14782600
H	-3.37573000	1.70793500	0.23225400
H	-2.22357500	-2.37260600	-0.50101400
H	-3.98990300	-0.65440800	-0.22672900
H	4.03725800	-0.37484600	-0.76177100
H	2.89361400	0.58438700	-1.75333200
H	3.68197200	1.32729000	-0.36653600

18	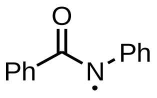	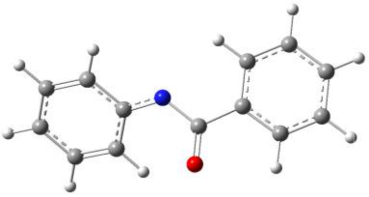

Cartesian Coordinates

N	-0.51671200	0.36414800	0.71620800
C	0.45407100	-0.62933500	0.64311800
C	1.81150400	-0.15739900	0.22937900
C	2.08940700	1.19588800	-0.00078400
C	2.82217200	-1.11032300	0.04956400
C	3.36191800	1.58828900	-0.40800100
H	1.30972500	1.93053900	0.15496600
C	4.09137200	-0.71554900	-0.35750000
H	2.59211500	-2.15231000	0.23557500
C	4.36299700	0.63455500	-0.58778400

H	3.57417700	2.63739000	-0.58040900
H	4.87037500	-1.45683800	-0.49557600
H	5.35389900	0.94169000	-0.90373800
O	0.23737500	-1.78882400	0.97126700
C	-1.76970500	0.19374500	0.27164600
C	-2.71860500	1.21094600	0.60353400
C	-2.21219000	-0.89912400	-0.54218500
C	-4.02139700	1.13905200	0.15411600
H	-2.37474800	2.03259200	1.21996300
C	-3.51861800	-0.94951900	-0.98512000
H	-1.51423600	-1.68626800	-0.79152700
C	-4.43048500	0.06066900	-0.64338500
H	-4.72988300	1.91625600	0.41658300
H	-3.84449600	-1.77960000	-1.60165500
H	-5.45358400	0.00673400	-0.99737200

19

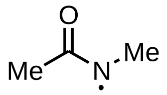

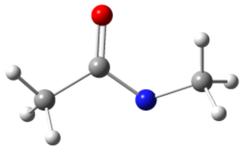

Cartesian Coordinates

C	1.92011600	-0.18377900	0.15712200
N	0.64358000	-0.61676700	-0.34054000
C	-0.47060700	0.15154200	-0.04312100
C	-1.75695600	-0.61917300	0.11531600
O	-0.40566800	1.37410600	-0.03438600
H	1.88527000	0.79644500	0.64141900
H	2.26893200	-0.93672800	0.87956700
H	2.65099900	-0.18475300	-0.65741800
H	-2.51751300	0.02358700	0.55707100
H	-2.09633200	-0.95597900	-0.86835300
H	-1.60638400	-1.50959200	0.73068400

20

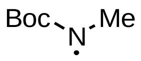

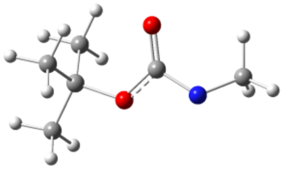

Cartesian Coordinates

C	-3.27859900	-0.42186300	0.31825200
N	-2.16213000	-0.51593900	-0.58461600
C	-1.02144100	0.19098400	-0.20792400
O	-1.03651900	1.38822500	0.00078900
H	-3.36346800	0.58618900	0.74236900
H	-4.19931200	-0.69732500	-0.19625000
H	-3.13122100	-1.11903500	1.15862800
O	0.04449300	-0.61214200	-0.20853100
C	1.41662800	-0.09824600	0.04126500
C	1.49946500	0.50894200	1.44304300
C	1.79435500	0.90042400	-1.05425700

C	2.26258100 -1.36653100 -0.05790900	
H	2.54008200 0.75945100 1.66608000	
H	0.89984400 1.41481900 1.52221600	
H	1.15832000 -0.21077900 2.19171800	
H	2.84652600 1.17627100 -0.94260000	
H	1.66488400 0.44957600 -2.04119200	
H	1.19000400 1.80462900 -0.99551500	
H	3.31560400 -1.12471600 0.10522700	
H	1.95327600 -2.09626300 0.69352800	
H	2.15864700 -1.82217200 -1.04477800	
21	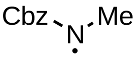	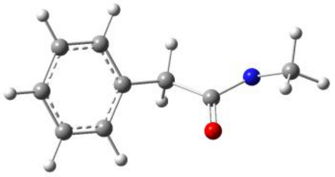

Cartesian Coordinates

C	-3.88136200	0.36777700	-0.77748100
N	-2.99683800	0.33330800	0.35517200
C	-1.76007400	-0.26356500	0.20134700
C	-0.65948600	0.34714600	1.06352100
O	-1.62182600	-1.27554200	-0.47090900
H	-3.51396300	-0.21472900	-1.62729800
H	-4.87619200	0.02643900	-0.47571600
H	-3.99776600	1.41920300	-1.07975400
H	-0.90002600	1.39813800	1.24104800
H	-0.73768200	-0.14738700	2.04022800
C	0.73399700	0.19690600	0.50131500
C	1.34654300	-1.05829200	0.40937900
C	1.44082600	1.32158300	0.06482100
C	2.63428600	-1.18190900	-0.10606900
H	0.80875000	-1.94102700	0.73382300
C	2.73036800	1.20018500	-0.45110900
H	0.98103200	2.30262500	0.13270600
C	3.33087800	-0.05368800	-0.53801200
H	3.09454100	-2.16160000	-0.17083700
H	3.26376100	2.08431500	-0.78224900
H	4.33416500	-0.15164700	-0.93715800

22	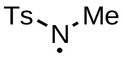	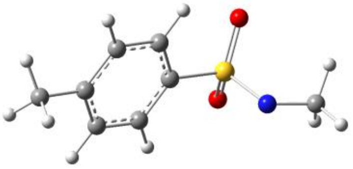

Cartesian Coordinates

C	3.54860600	0.00154700	1.31144700
N	2.10419800	0.00020200	1.21304900
H	3.97501500	0.88422000	0.81578800
H	3.82176600	0.00308300	2.36605400
H	3.97619800	-0.88197600	0.81827200

S	1.56835100	-0.00064500	-0.40670300
C	-0.20617800	0.00009600	-0.19799600
C	-0.88293000	1.21386300	-0.11812200
C	-0.88406000	-1.21359600	-0.11686900
C	-2.26331500	1.20396500	0.05555300
H	-0.33882900	2.14561200	-0.20758100
C	-2.26385700	-1.20256500	0.05651300
H	-0.34041300	-2.14570100	-0.20535900
C	-2.97326500	0.00126500	0.15172300
H	-2.79731700	2.14652500	0.11045200
H	-2.79859600	-2.14477600	0.11211400
O	1.97383000	-1.27414600	-1.00934300
O	1.97467900	1.27159400	-1.01141400
C	-4.46564000	-0.00003900	0.36930800
H	-4.93983800	-0.85031500	-0.12580700
H	-4.70029600	-0.07024200	1.43701900
H	-4.92492200	0.91567500	-0.00833800

23	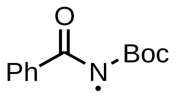	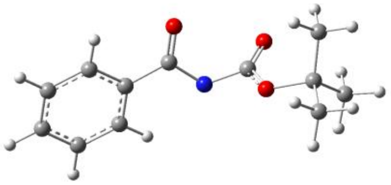

Cartesian Coordinates

C	1.30510900	-0.08662600	-0.83186300
N	-0.06062600	-0.38898100	-0.79877000
C	-0.93073200	0.63614900	-0.39638500
O	-0.57407200	1.80361800	-0.32163000
O	1.79863300	0.29101100	-1.86925800
O	1.89858600	-0.33728100	0.32322100
C	3.37633800	-0.19272400	0.50634300
C	3.77025600	1.26766000	0.28667700
C	3.56347900	-0.60471200	1.96423300
C	4.10028500	-1.15162200	-0.43857200
H	2.99663600	0.05288900	2.62634900
H	3.22661800	-1.63136300	2.12239000
H	4.62050800	-0.54068900	2.23304900
H	4.82405400	1.39622100	0.54811900
H	3.62990100	1.56889200	-0.75073300
H	3.17693500	1.92445000	0.92676100
H	3.73891200	-2.17349200	-0.29931600
H	3.96505100	-0.86679100	-1.48148300
H	5.16956900	-1.13719000	-0.21160700
C	-2.31663100	0.18210600	-0.10214200
C	-2.73716600	-1.12845600	-0.36809500
C	-3.21479200	1.10050500	0.45892500
C	-4.04390300	-1.51107800	-0.07778500
H	-2.04148900	-1.83057000	-0.80840200
C	-4.51673900	0.71317800	0.74795900
H	-2.87403200	2.10943300	0.65642900

C	-4.93290500	-0.59364900	0.47970700
H	-4.36917100	-2.52309400	-0.28949200
H	-5.20973200	1.42499100	1.18151800
H	-5.95015500	-0.89399900	0.70513300

24	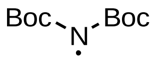	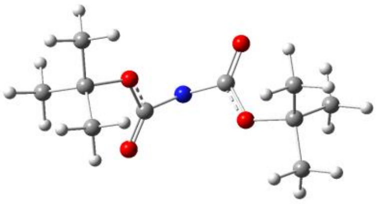

Cartesian Coordinates

C	-1.09009900	0.97017200	-0.51758900
N	-0.00000100	1.68467200	0.00015300
C	1.09007800	0.97003700	0.51779600
O	1.39921400	1.09823400	1.67839600
O	1.69269900	0.23978900	-0.41632500
C	2.97777800	-0.47575300	-0.15673700
C	2.75503800	-1.55349000	0.90439500
C	4.05071800	0.53604600	0.24612500
C	3.28468400	-1.09396600	-1.51858100
H	3.66326100	-2.15463200	0.99941700
H	2.52381800	-1.11687700	1.87509500
H	1.93920800	-2.21809700	0.60973400
H	5.01665700	0.02772900	0.30690300
H	4.13146800	1.32730800	-0.50308600
H	3.83677900	0.98572700	1.21520900
H	4.21899200	-1.65802400	-1.46625200
H	2.48576800	-1.77320000	-1.82318900
H	3.38795400	-0.31864100	-2.28027600
O	-1.39931100	1.09869200	-1.67814500
O	-1.69264500	0.23961900	0.41634000
C	-2.97776100	-0.47581700	0.15663100
C	-2.75503500	-1.55337700	-0.90468500
C	-3.28469900	-1.09424000	1.51837500
C	-4.05066500	0.53609900	-0.24604700
H	-2.48577700	-1.77350900	1.82289700
H	-3.38799200	-0.31903900	2.28019500
H	-4.21899900	-1.65829800	1.46594000
H	-3.66331500	-2.15439900	-0.99991700
H	-2.52367500	-1.11658100	-1.87527200
H	-1.93930900	-2.21812600	-0.61006700
H	-4.13127800	1.32729400	0.50324800
H	-3.83678200	0.98586800	-1.21510400
H	-5.01665200	0.02786700	-0.30677500

25	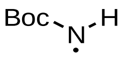	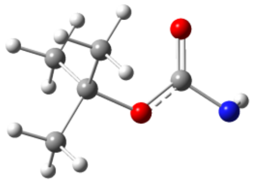

Cartesian Coordinates

N	2.56014700	-0.90425800	0.08598100
C	1.46514100	-0.05530600	-0.00845300
O	1.61872700	1.15329200	0.02329900
O	0.31709800	-0.72186200	-0.01822000
C	-1.00137200	-0.03110600	-0.00334400
C	-1.13742900	0.85548600	-1.24221400
C	-1.14744900	0.75277600	1.30186400
C	-1.98213100	-1.20078300	-0.05489700
H	-2.15387900	1.25535200	-1.28719800
H	-0.43880500	1.69104700	-1.21755300
H	-0.96413800	0.27280600	-2.15058900
H	-2.16297100	1.15233300	1.36901900
H	-0.98374700	0.09698600	2.16040500
H	-0.44424700	1.58330700	1.35170500
H	-3.00804700	-0.82518500	-0.04299600
H	-1.83681500	-1.78601900	-0.96534000
H	-1.84215600	-1.85865300	0.80516200
H	3.24662300	-0.63000900	-0.62285800

26

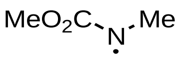

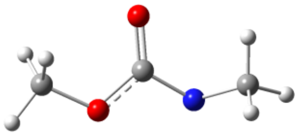

Cartesian Coordinates

C	2.34024600	-0.15979700	0.31490400
N	1.19516900	-0.52014200	-0.47712400
C	0.01338700	0.13824200	-0.16295400
O	-0.09057600	1.34604300	-0.09211400
H	2.32120200	0.90063400	0.59298000
H	2.33040500	-0.74408800	1.24918200
H	3.25651900	-0.40092400	-0.22415800
O	-1.00275900	-0.72824400	-0.04685600
C	-2.29876800	-0.14563600	0.19428100
H	-2.59302600	0.48177400	-0.64788900
H	-2.28726700	0.45392300	1.10548900
H	-2.97652300	-0.98957200	0.29863500

27

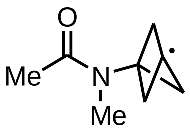

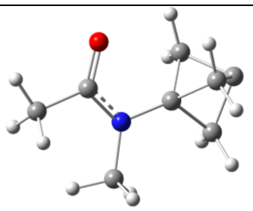

Cartesian Coordinates

C	-2.53637700	-0.35870300	0.03798400
C	-1.51988600	-0.90741300	-0.98613200
C	-0.79498200	0.09015600	-0.02724300
C	-1.99148200	1.07202700	-0.17761300
C	-1.52780900	-0.58215600	1.18463100
H	-1.23901200	-1.60688600	1.40394900

H	-1.65214300	0.03395900	2.07587900
H	-2.14528100	1.51046100	-1.16437900
H	-2.14985200	1.78472600	0.63253600
H	-1.63586600	-0.57730700	-2.01892700
H	-1.22705000	-1.94907300	-0.89546200
N	0.60619500	0.49102800	-0.09659000
C	1.55840500	-0.50149200	-0.01677600
C	3.01935500	-0.07604200	-0.01007000
O	1.24777200	-1.68272900	0.03479400
H	3.62039900	-0.98129200	0.04342600
H	3.28489600	0.47402000	-0.91703600
H	3.25286700	0.55903900	0.84876900
C	0.91033600	1.91401200	0.03237300
H	1.90257900	2.12813900	-0.35841700
H	0.19275400	2.49236800	-0.54975900
H	0.86480900	2.25414900	1.07427700

28	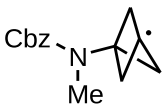	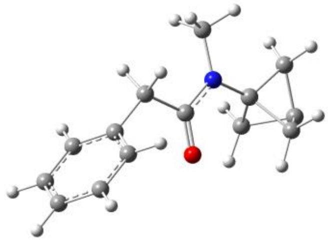

Cartesian Coordinates

C	-4.11120700	-1.21216700	0.22897100
C	-3.04052700	-1.38328200	-0.86924700
C	-2.68444700	-0.13875700	0.00632800
C	-4.17250100	0.30619300	-0.05737100
C	-3.01357500	-0.97947800	1.28802200
H	-2.33896200	-1.80500100	1.49898400
H	-3.29839100	-0.41333100	2.17560700
H	-4.55455300	0.60423500	-1.03462000
H	-4.53140000	0.94377100	0.75136100
H	-3.34916900	-1.17340800	-1.89390400
H	-2.36619800	-2.23040900	-0.78544900
N	-1.54830100	0.75866700	-0.18693800
C	-0.28781200	0.20376600	-0.12583700
C	0.89504900	1.18337300	-0.23506400
O	-0.12197000	-0.99771200	-0.00348300
H	0.82902700	1.69704900	-1.20042300
H	0.78520800	1.95893600	0.52935400
C	-1.79412200	2.19980100	-0.17533900
H	-1.07925700	2.71986400	-0.81158900
H	-2.78889000	2.39299200	-0.57358700
H	-1.73950500	2.62614800	0.83397700
C	2.24048300	0.51390000	-0.09881500
C	2.86626500	-0.06511700	-1.20612300
C	2.88650900	0.45956500	1.13868100
C	4.10650000	-0.68602200	-1.08028800
H	2.37662000	-0.03622500	-2.17421300

C	4.12738800	-0.16100200	1.27024600
H	2.41427200	0.90314800	2.00972700
C	4.74153900	-0.73624400	0.15973600
H	4.57681900	-1.13115600	-1.95009500
H	4.61357900	-0.19405300	2.23897600
H	5.70739500	-1.21871300	0.25892200

29	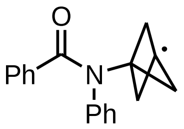	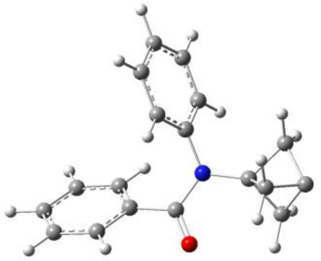

Cartesian Coordinates

C	-3.87093800	-1.37683500	0.09499900
C	-2.94525700	-1.36166600	-1.13816200
C	-2.15634600	-0.84510900	0.11558000
C	-3.37926700	0.03291000	0.49639500
C	-2.75573600	-2.01702300	0.95086100
H	-2.42537400	-3.01533700	0.68001100
H	-2.81057000	-1.85519600	2.02780300
H	-3.62052600	0.85333100	-0.17822900
H	-3.47829300	0.30473500	1.54735300
H	-3.16546400	-0.62099800	-1.90719500
H	-2.62532600	-2.32096500	-1.53656700
N	-0.75764900	-0.38794100	0.14844800
C	0.21478100	-1.35138000	-0.09443000
O	-0.08662300	-2.49857700	-0.38800400
C	-0.52608800	1.01628700	-0.04652200
C	-0.81369700	1.92158900	0.97841100
C	-0.05282700	1.49155200	-1.27198800
C	-0.62327600	3.28639600	0.78025200
H	-1.17581300	1.54955200	1.92944000
C	0.15096000	2.85681900	-1.46185400
H	0.15825600	0.79043900	-2.07088900
C	-0.13594200	3.75788200	-0.43861900
H	-0.84690500	3.98137900	1.58176300
H	0.52659800	3.21504200	-2.41361100
H	0.01774400	4.82023200	-0.58914300
C	1.66075000	-0.96808100	0.06471200
C	2.12233100	-0.13271000	1.08722500
C	2.58533100	-1.57244500	-0.79483400
C	3.48676500	0.10245900	1.23890900
H	1.42131300	0.32229400	1.77485400
C	3.94605200	-1.31777700	-0.65676400
H	2.22364700	-2.24803800	-1.56047000
C	4.40021200	-0.47999500	0.36180600
H	3.83599300	0.73993100	2.04332600
H	4.65309100	-1.78081300	-1.33584800

H	5.46129900	-0.28864000	0.47643500

30	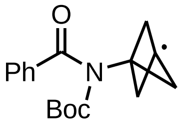	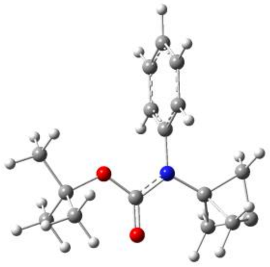

Cartesian Coordinates

C	2.95530400	-2.63367200	-0.00093500
C	1.87366100	-2.51989700	1.09426700
C	1.62011900	-1.43079900	0.00061200
C	3.13578900	-1.09719000	0.00636100
C	1.87996400	-2.51025300	-1.10128700
H	1.14165500	-3.30481500	-1.17210900
H	2.21012000	-2.13606400	-2.07079900
H	3.53241000	-0.64944000	0.91721000
H	3.53767900	-0.64129700	-0.89811600
H	2.19825000	-2.15418400	2.06889100
H	1.13478000	-3.31478600	1.15382100
N	0.56368300	-0.41996900	0.00238300
C	-0.74301700	-0.85994700	0.00033300
O	-1.05274000	-2.03535700	-0.00257700
C	0.92105100	0.97209700	0.00107400
C	1.10937200	1.64488300	-1.20660000
C	1.11262100	1.64626200	1.20744600
C	1.48409300	2.98677100	-1.20710600
H	0.95380000	1.11392700	-2.13861700
C	1.48738500	2.98815000	1.20536200
H	0.95947300	1.11637800	2.14046400
C	1.67453200	3.66065200	-0.00151100
H	1.62669300	3.50551200	-2.14845800
H	1.63251900	3.50799000	2.14572000
H	1.96645000	4.70471800	-0.00250000
O	-1.59948400	0.18273200	0.00244600
C	-3.06324000	-0.02271300	0.00040000
C	-3.49119800	-0.75491100	-1.27397800
C	-3.49366600	-0.76288500	1.26942100
C	-3.59351700	1.41164200	0.00444400
H	-3.12929900	-0.22316200	-2.15784700
H	-3.11003000	-1.77465500	-1.29262300
H	-4.58314900	-0.78790700	-1.32287300
H	-3.25019400	1.94679400	0.89240000
H	-3.24712700	1.95289400	-0.87860400
H	-4.68630300	1.40497400	0.00256000
H	-3.13119300	-0.23783900	2.15708200
H	-4.58567700	-0.79396500	1.31746900
H	-3.11435700	-1.78342500	1.28147400

31	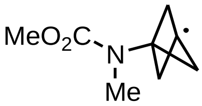	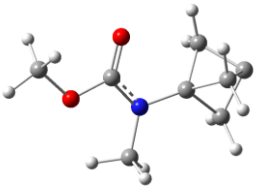

Cartesian Coordinates

C	-2.84372600	-0.61606900	0.06407200
C	-1.76271500	-1.07948800	-0.93572700
C	-1.17424900	0.04788800	-0.03024900
C	-2.48544900	0.86039200	-0.22438200
C	-1.81907200	-0.65155200	1.21699100
H	-1.40398700	-1.62034600	1.48573300
H	-2.02345900	-0.01415400	2.07782700
H	-2.68821000	1.22721500	-1.23113000
H	-2.73354400	1.58659300	0.55013300
H	-1.91643100	-0.81820800	-1.98304700
H	-1.33934200	-2.06993400	-0.79612900
N	0.15795400	0.62570000	-0.12683200
C	1.21953200	-0.23476600	-0.04044900
O	1.13338600	-1.44608700	-0.00364000
C	0.30245500	2.07426100	0.02132600
H	1.27392200	2.38247700	-0.35467700
H	-0.47434300	2.57145700	-0.55992900
H	0.21473000	2.38780600	1.06809600
O	2.40737500	0.42853000	-0.01143800
C	3.57260600	-0.41077800	0.03222500
H	4.41774600	0.27387100	0.07060500
H	3.55548000	-1.04809800	0.91707700
H	3.62940000	-1.03745200	-0.85894700

32	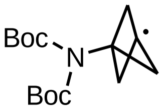	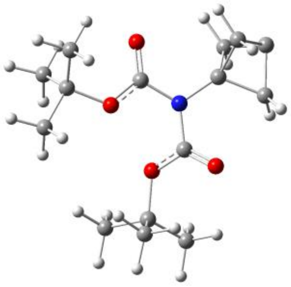

Cartesian Coordinates

C	-4.24756400	-0.05598600	0.11284800
C	-3.32071000	0.31794800	1.28724000
C	-2.45744900	-0.00692800	0.02532800
C	-3.34080500	-1.25745400	-0.23538800
C	-3.43182100	0.84567300	-0.84249700
H	-3.44111100	1.91494600	-0.64421700
H	-3.46799300	0.59818700	-1.90307900
H	-3.27720000	-2.05657200	0.50291900
H	-3.36434600	-1.62637100	-1.25717900
H	-3.25502400	-0.39705600	2.10763500

H	-3.31367100	1.35407800	1.61385000
N	-0.98632700	0.01448100	-0.02590300
C	-0.30680900	-1.18466600	-0.36585800
C	-0.37263100	1.25454800	0.18031600
O	-0.97276500	2.20323400	0.63443600
O	0.89401800	1.25951900	-0.25519100
C	1.74924300	2.46307300	-0.11192900
C	1.18192500	3.60813000	-0.95426800
C	1.89227400	2.83560100	1.36516300
C	3.08438900	1.98489200	-0.68253500
H	1.04824200	3.28792900	-1.99076200
H	0.22635000	3.95413400	-0.56403400
H	1.88662300	4.44428700	-0.94687300
H	2.63585100	3.63109600	1.46423300
H	0.94979900	3.18745300	1.78163800
H	2.23718800	1.97481600	1.94347900
H	3.47669800	1.14938300	-0.09914800
H	2.96714300	1.66164800	-1.71918300
H	3.81307000	2.79844100	-0.65426700
O	-0.72014500	-1.95316600	-1.19993100
O	0.76611300	-1.36074400	0.40722600
C	1.70163800	-2.49264000	0.18280100
C	2.27131900	-2.43327100	-1.23580200
C	2.79145800	-2.22491600	1.22045400
C	0.98942400	-3.81545300	0.47334900
H	3.06013700	-3.18365900	-1.33593400
H	1.50612400	-2.63453700	-1.98411800
H	2.70910700	-1.45150200	-1.43023400
H	3.27733400	-1.26545000	1.03239100
H	2.36871200	-2.20756000	2.22729800
H	3.54789100	-3.01196300	1.17528300
H	0.55131200	-3.80065200	1.47459300
H	0.20468800	-4.01309200	-0.25506500
H	1.71631100	-4.63139900	0.43445400

33

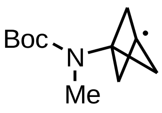

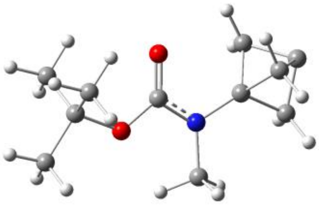

Cartesian Coordinates

C	3.76488300	-0.67269200	-0.07788900
C	2.68573200	-1.07329300	0.95042300
C	2.12280600	0.05737400	0.03194500
C	3.46809700	0.82179300	0.18540300
C	2.71893300	-0.69211700	-1.21144700
H	2.26259300	-1.64924200	-1.45332000
H	2.93181600	-0.08007500	-2.08860900
H	3.70390200	1.20109400	1.18035700

H	3.72940600	1.52218100	-0.60840700
H	2.86908900	-0.79669400	1.98914000
H	2.22223200	-2.04928200	0.83950200
N	0.81601100	0.68821400	0.13852000
C	0.72996800	2.14116400	-0.00662200
C	-0.28300900	-0.13326800	0.05501600
O	-0.21751900	-1.34775500	0.02363100
O	-1.43048200	0.58612000	0.02291800
C	-2.75129300	-0.07539600	-0.01038500
C	-2.89874800	-0.89508100	-1.29498800
C	-2.95007800	-0.92249000	1.24920600
C	-3.71296900	1.11386200	-0.01730600
H	-2.70924900	-0.26743600	-2.16987200
H	-2.21076300	-1.73866000	-1.30873500
H	-3.92183300	-1.27473400	-1.36675000
H	-3.97665300	-1.29866700	1.27426600
H	-2.26677500	-1.76984900	1.27022400
H	-2.79094700	-0.31543500	2.14443300
H	-3.58157900	1.72144200	0.88092200
H	-3.53874500	1.74468600	-0.89185400
H	-4.74580900	0.75858700	-0.04669000
H	-0.21043600	2.49272000	0.40819300
H	1.55146500	2.60288300	0.54135500
H	0.78828100	2.45292500	-1.05632500

9.4 Reaction Enthalpy (– ΔH_r), Bond distance [d(C–C)] and Charge Transfer (δ^{TS}) for Radical Addition Reactions

DFT Method: UB3LYP/6-31G(d)

Radical addition reaction	**– ΔH_r (kJ/mol)**	***d* (C–N) (Å)**	**δ^{TS}**
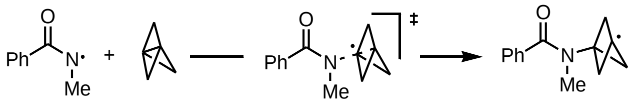	77.0	2.25107	-0.028329
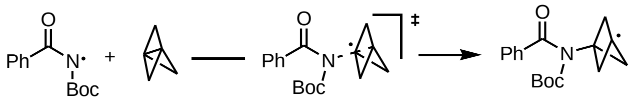	104.7	2.48194	0.124158
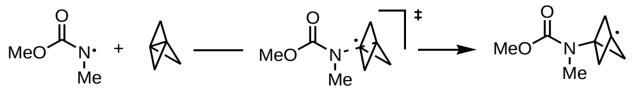	101.8	2.33155	-0.051719
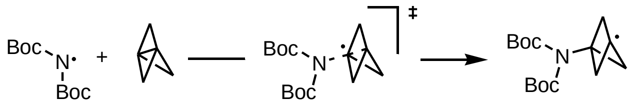	138.2	2.70520	0.168397
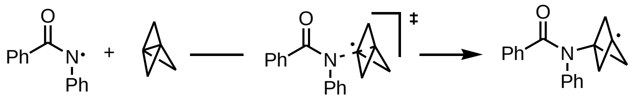	30.5	2.12931	0.002574
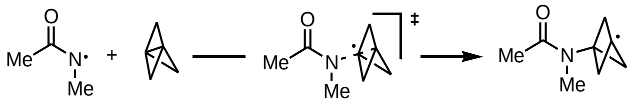	92.9	2.37529	-0.015192
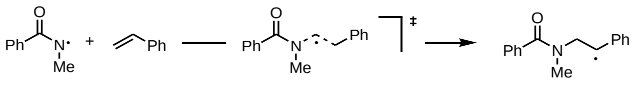	94.1	2.29031	-0.039279

Computed Energies [values are in Hartree]

Species	Total Electronic Energy	Sum of Electronic and Zero-point Energies	Sum of Electronic and Thermal Enthalpies	Gibbs Free Energy
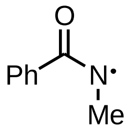	-439.5926742	-439.450824	-439.440834	-439.486402
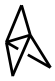	-194.0089531	-193.915103	-193.910218	-193.941394
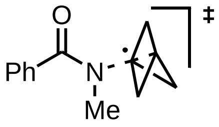	-633.5913526	-633.354393	-633.339681	-633.397495
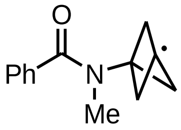	-633.6364151	-633.395253	-633.381295	-633.436451
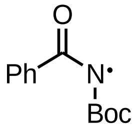	-746.100821	-745.858386	-745.842259	-745.902028
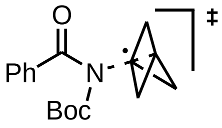	-940.1120374	-939.775835	-939.754132	-939.829674
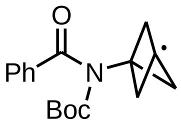	-940.1533611	-939.813380	-939.792552	-939.863952
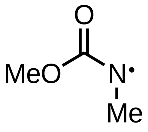	-323.0746211	-322.980927	-322.972702	-323.013349

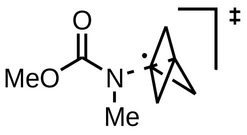	-517.0807446	-516.891661	-516.878861	-516.931747
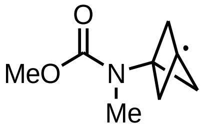	-517.1279377	-516.934787	-516.922605	-516.973384
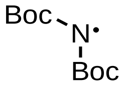	-747.5247292	-747.246321	-747.228226	-747.291139
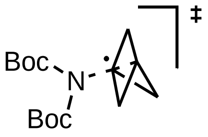	-941.544678	-941.172291	-941.148534	-941.227571
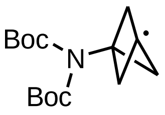	-941.5904364	-941.214061	-941.191321	-941.265719
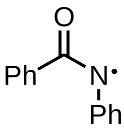	-631.34994	-631.154542	-631.141808	-631.194873
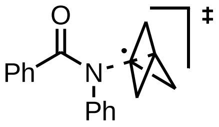	-825.3431024	-825.053128	-825.035599	-825.100450
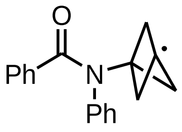	-825.3747677	-825.081279	-825.064289	-825.127025
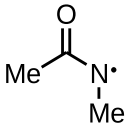	-247.8510321	-247.763154	-247.755772	-247.794005
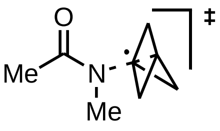	-441.853605	-441.670410	-441.658409	-441.709199

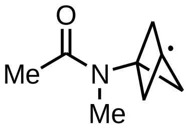	-441.9014825	-441.713638	-441.702609	-441.750018
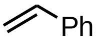	-309.6482589	-309.514528	-309.506814	-309.546006
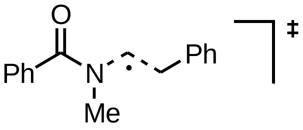	-749.2283637	-748.951382	-748.933887	-748.999787
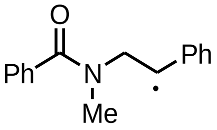	-749.2820983	-749.001209	-748.984118	-749.048578

Optimized Structures and Cartesian Coordinates

No.	Species	Optimized Structure
1	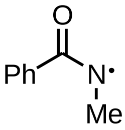	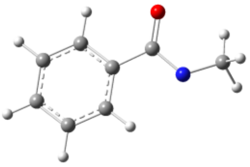

Cartesian Coordinates

N	-2.07046600	-0.74503600	-0.17205500
C	-1.25453400	0.37986200	-0.12919900
C	-3.41560300	-0.58824500	0.31163500
C	0.21002100	0.12034000	-0.06930400
C	1.07889100	1.21293900	0.06914600
C	0.73183900	-1.18057800	-0.12924700
C	2.45276000	1.00650200	0.14959300
H	0.65628400	2.21170500	0.11145000
C	2.10856600	-1.38320200	-0.04720400
H	0.05376400	-2.01842300	-0.25101100
C	2.96933000	-0.29204700	0.09220800
H	3.12336700	1.85462700	0.25716500
H	2.51076900	-2.39129500	-0.09753800
H	4.04258100	-0.45189300	0.15459900
O	-1.73090100	1.51591400	-0.21777900
H	-3.59394700	-1.37699900	1.05772500
H	-3.62403900	0.39820700	0.73948000
H	-4.11593600	-0.79141400	-0.51101500

No.	Species	Optimized Structure
2	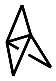	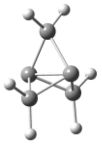

Cartesian Coordinates

C	-0.00011700	0.00010500	0.78971800
C	-1.01164700	0.81183900	-0.00021900
C	0.00016600	0.00004000	-0.78970300
C	1.20896400	0.47007900	0.00019300
C	-0.19734500	-1.28202100	0.00001400
H	-1.19181400	-1.72304000	-0.00014700
H	0.61859700	-2.00153800	0.00019200
H	1.42423700	1.53645900	0.00018800
H	2.08810500	-0.17066900	0.00037800
H	-0.89653900	1.89363500	-0.00024200
H	-2.04271400	0.46490700	-0.00038900

No.	Species	Optimized Structure
3	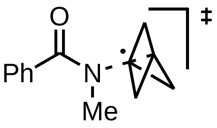	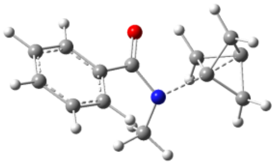

Cartesian Coordinates

C	4.15302900	-0.44855100	-0.27930300
C	3.28263900	-0.78280800	0.92414600
C	2.66184400	0.12409200	-0.12848400
C	3.86069400	1.05017000	-0.22762800
C	3.06211300	-0.74442800	-1.30466800
H	2.64847400	-1.74857400	-1.32917800
H	3.14679000	-0.25802600	-2.27469600
H	4.16279400	1.58400900	0.67194900
H	3.98369800	1.61417200	-1.15032400
H	3.55750000	-0.32944800	1.87544900
H	2.87423700	-1.78696100	0.99536300
N	0.51572500	0.78771800	0.01679000
C	0.50619100	1.65173400	1.18130400
H	-0.51607300	2.00852500	1.37462800
H	0.85150700	1.14260400	2.09336400
H	1.14521900	2.51938500	0.99496600
C	-0.24642200	-0.37580500	0.05309100
C	-1.73905700	-0.16333700	-0.04094300
C	-2.30799800	1.01433800	-0.54667300
C	-2.57513100	-1.21051100	0.37134600
C	-3.69460700	1.14556800	-0.62581300
H	-1.66203100	1.81130700	-0.90151500
C	-3.95933300	-1.07342400	0.30031300
H	-2.11810500	-2.12313400	0.73982700
C	-4.52179200	0.10549500	-0.19723700
H	-4.12881700	2.05654700	-1.02909100
H	-4.60135700	-1.88646400	0.62879500
H	-5.60200800	0.20995500	-0.25686600
O	0.21938500	-1.51039100	0.07163500

4

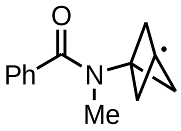

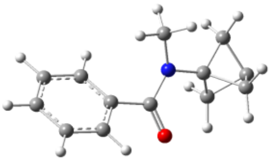

Cartesian Coordinates

C	-3.96967500	-0.23384400	0.28231200
C	-3.12776500	-0.81278600	-0.87551300
C	-2.21962500	0.10727600	0.01080700
C	-3.36068200	1.16580500	0.03538900
C	-2.83558300	-0.55167700	1.28107500
H	-2.58348800	-1.59756000	1.44630200
H	-2.79904400	0.04687400	2.19541700
H	-3.60646300	1.65216600	-0.91236800
H	-3.36271600	1.86395700	0.87702000
H	-3.35709400	-0.45195800	-1.88215000
H	-2.89488100	-1.87754300	-0.84767900
N	-0.81548000	0.43812200	-0.18807800
C	-0.56001700	1.58523100	-1.05962500
H	0.50013700	1.64475900	-1.29941200

H	-1.11828000	1.48333500	-1.99937300
H	-0.86696700	2.51953100	-0.57666500
C	0.10617400	-0.56747400	0.04860400
C	1.56970900	-0.21310000	0.03241700
C	2.07118700	0.96391300	0.60563900
C	2.46585400	-1.16264500	-0.47682600
C	3.44647100	1.19496500	0.65054600
H	1.38570000	1.68736000	1.03808900
C	3.83778700	-0.92207400	-0.44942600
H	2.07030300	-2.08853300	-0.88229700
C	4.33107700	0.25768000	0.11401100
H	3.82647500	2.10407400	1.10886200
H	4.52408800	-1.65851800	-0.85863800
H	5.40182000	0.44124500	0.14316200
O	-0.24258900	-1.71295900	0.32122800

5	t-Bu−O•	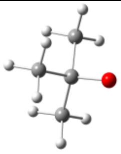

Cartesian Coordinates

O	-0.00000700	0.26187800	1.43188200
C	0.00000100	-0.02664200	0.08145400
C	1.27781900	-0.79250500	-0.31550600
C	-0.00003500	1.38883300	-0.57899800
C	-1.27778000	-0.79256900	-0.31550600
H	1.31561600	-0.96802300	-1.39677400
H	2.16595600	-0.22685900	-0.01721400
H	1.30584700	-1.76456600	0.18884900
H	-0.00002800	1.26207700	-1.66702900
H	-0.89066300	1.94964100	-0.28321400
H	0.89056100	1.94969000	-0.28320800
H	-1.31557200	-0.96807800	-1.39677600
H	-1.30575400	-1.76463600	0.18884000
H	-2.16594400	-0.22697200	-0.01720300

6	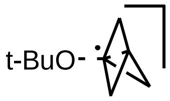	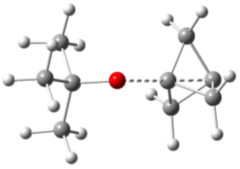

Cartesian Coordinates

C	2.86569300	0.00078200	-0.15576100
C	1.87434900	-0.77195700	-1.01665600
C	1.28626800	0.00056900	0.15767700
C	2.29150400	-0.51148500	1.16499000
C	2.03965600	1.28344200	-0.14548300
H	1.85053400	1.76310400	-1.10388000
H	2.19522000	1.97272500	0.68184100
H	2.31960400	-1.58509600	1.33968900
H	2.45745500	0.09575500	2.05237900

H	1.88717900	-1.85753500	-0.94186900
H	1.67940800	-0.39017200	-2.01720600
O	-0.68500600	0.04094900	0.94932800
C	-1.72691400	-0.00276600	0.01632900
C	-1.65703100	1.17235500	-0.97686000
C	-1.77863600	-1.35408600	-0.72106000
C	-2.98816800	0.14945800	0.91044600
H	-2.54313300	1.20366300	-1.62227600
H	-1.58567500	2.12013300	-0.43318100
H	-2.66475000	-1.42822800	-1.36322000
H	-0.89221100	-1.48144400	-1.35113800
H	-1.80243100	-2.17494200	0.00338100
H	-0.77426500	1.07940700	-1.61856800
H	-3.88985300	0.11920000	0.28778100
H	-3.03654300	-0.66350000	1.64108700
H	-2.96081600	1.10146900	1.44881900
7	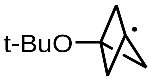	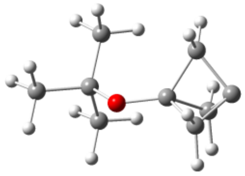	
Cartesian Coordinates			
C	2.67360400	0.00235800	-0.28235600
C	1.56183000	-0.86651000	-0.90607400
C	0.94938300	-0.00685300	0.25533700
C	2.18048700	-0.36955100	1.13060200
C	1.74534200	1.23257800	-0.29148600
H	1.45600500	1.62631100	-1.26806600
H	1.96077900	2.02360000	0.43141300
H	2.26524700	-1.41642900	1.43274700
H	2.41589300	0.32345300	1.94199500
H	1.61841500	-1.94472300	-0.73634900
H	1.25963200	-0.61870400	-1.92689200
O	-0.32489000	-0.03854400	0.83017800
C	-1.52774500	0.00072800	0.01823800
C	-1.50696700	1.15678900	-0.99127300
C	-1.73578600	-1.34959200	-0.68422300
C	-2.63102400	0.22980800	1.05833400
H	-2.49103500	1.25671700	-1.46316100
H	-1.27029900	2.10147500	-0.49068200
H	-2.70617100	-1.36936500	-1.19380100
H	-0.96229300	-1.54162400	-1.43328600
H	-1.71554100	-2.16269000	0.04882400
H	-0.77466900	0.99347500	-1.78770100
H	-3.61712400	0.23825400	0.58128900
H	-2.61350800	-0.56592300	1.80979100
H	-2.48095900	1.18599100	1.56986500

8	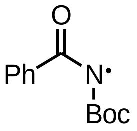	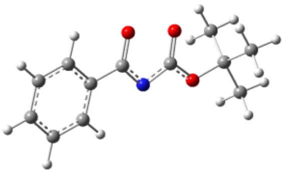

Cartesian Coordinates

N	-0.03681700	0.43956500	-0.00021000
C	1.18694700	-0.14057600	-0.00007300
C	-0.97647700	-0.49887400	-0.00011100
O	1.37046000	-1.39012000	-0.00000200
O	2.19586800	0.72584500	-0.00000900
C	3.61381700	0.30039200	0.00007400
C	3.91947700	-0.49128500	-1.27431900
C	3.91935200	-0.49085400	1.27470900
C	4.34787100	1.64329900	-0.00019600
H	3.38866200	-1.44498500	1.29048300
H	4.99510800	-0.69092500	1.32939900
H	3.63509900	0.08778500	2.16001500
H	3.63524400	0.08706100	-2.15983200
H	4.99524800	-0.69125500	-1.32882100
H	3.38882100	-1.44543300	-1.28984000
H	4.08501100	2.22704300	-0.88782600
H	4.08508000	2.22743700	0.88719200
H	5.43011400	1.47747800	-0.00018300
O	-0.72336000	-1.75648600	-0.00006600
C	-2.40914100	-0.09609900	-0.00004600
C	-2.74728900	1.26511500	-0.00005800
C	-3.41878300	-1.06990800	0.00003100
C	-4.08734000	1.64451100	0.00001200
H	-1.95170900	2.00155100	-0.00012900
C	-4.75651900	-0.68272900	0.00009800
H	-3.14729700	-2.12038700	0.00003900
C	-5.09233500	0.67353700	0.00009100
H	-4.34873000	2.69907800	0.00001100
H	-5.53725600	-1.43812300	0.00015600
H	-6.13689400	0.97362800	0.00014600

9	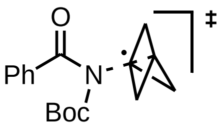	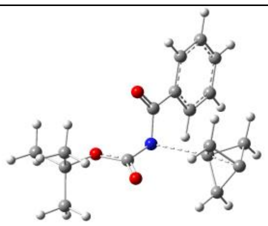

Cartesian Coordinates

C	-1.42584800	-3.61409900	-0.32110600
C	-0.65978400	-2.75546000	-1.30601400
C	-0.99110100	-2.13895700	0.04986700
C	-2.45483000	-2.55045600	0.03227700
C	-0.51725500	-3.33260000	0.86309400
H	0.53343000	-3.59062000	0.77182400

H	-0.94271600	-3.45605000	1.85683600
H	-3.07313300	-2.16212900	-0.77381000
H	-2.96048900	-2.64129100	0.99106300
H	-1.20413900	-2.37479300	-2.16763600
H	0.38618700	-2.98694000	-1.48247400
N	0.17573300	0.00388800	0.50454600
C	-0.50622400	0.73547000	-0.48060600
C	1.53054500	-0.28675500	0.23884600
O	1.96572000	-1.39918700	0.01526400
O	2.24030800	0.83854900	0.39076900
C	3.70065900	0.87496500	0.15197100
C	4.41702600	-0.01289700	1.17340600
C	3.99108100	0.46493900	-1.29466900
C	4.03162000	2.35134100	0.37964100
H	5.50099400	0.10921900	1.06687100
H	4.14101400	0.27839300	2.19274300
H	4.16592600	-1.06450100	1.02584400
H	5.04758000	0.64753000	-1.52183900
H	3.77524600	-0.59244900	-1.45673500
H	3.38016900	1.05775200	-1.98300000
H	3.48273600	2.98269700	-0.32597800
H	3.76311900	2.65568100	1.39640900
H	5.10435700	2.52053000	0.23832600
O	0.00143600	1.03475200	-1.56042400
C	-1.88680500	1.16368100	-0.10745400
C	-2.40949700	0.95638600	1.17744100
C	-2.66641900	1.81356400	-1.07572700
C	-3.69783100	1.39113700	1.48550100
H	-1.79666700	0.46026400	1.92162000
C	-3.95402400	2.24306700	-0.76617800
H	-2.24207800	1.97361800	-2.06158600
C	-4.47181800	2.03262300	0.51543100
H	-4.09753400	1.23293300	2.48356800
H	-4.55470000	2.74378400	-1.52068100
H	-5.47610800	2.37054200	0.75762100

10

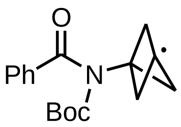

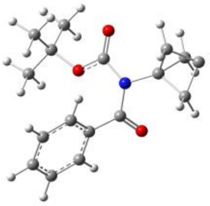

Cartesian Coordinates

C	4.27647200	-0.82434900	0.31069700
C	3.66310400	0.07063200	-0.79146800
C	2.52294100	-0.48963400	0.11090400
C	3.18833500	-1.89199800	0.05704300
C	3.37837200	-0.15513000	1.37159900
H	3.53503800	0.90226100	1.57709100
H	3.15681000	-0.73729800	2.27025500
H	3.18677600	-2.39187400	-0.91060700

H	2.95665100	-2.56827700	0.88445500
H	3.69238400	-0.30755600	-1.81538200
H	3.84889200	1.14400500	-0.72314600
N	1.07607700	-0.24812800	-0.01433000
C	0.29611200	-1.22810800	-0.70638700
C	0.64651200	1.07262200	0.17438500
O	1.32862900	1.91907900	0.71877300
O	-0.57133200	1.25667600	-0.36281200
C	-1.29008300	2.54253300	-0.22521700
C	-1.51174000	2.86304700	1.25569900
C	-0.51809900	3.64545400	-0.95503500
C	-2.61828300	2.24640800	-0.92530800
H	-2.02811700	2.03398900	1.75150500
H	-0.56570400	3.05140600	1.76608300
H	-2.14056100	3.75597100	1.34436500
H	-1.11203500	4.56645200	-0.95639800
H	0.43875900	3.84497500	-0.47039200
H	-0.33610900	3.35730100	-1.99600200
H	-2.45099800	1.97653900	-1.97286000
H	-3.13753900	1.41738300	-0.43462400
H	-3.26250200	3.13136100	-0.89395600
O	0.77736100	-1.85406900	-1.63318100
C	-1.06555400	-1.53514300	-0.17513600
C	-1.42290500	-1.30068900	1.16003200
C	-1.97808100	-2.16195400	-1.03488700
C	-2.68252900	-1.67650500	1.62337800
H	-0.70780300	-0.84327600	1.83652100
C	-3.24208100	-2.52196300	-0.57424500
H	-1.67883600	-2.35768200	-2.05939700
C	-3.59677000	-2.27940600	0.75560600
H	-2.94959400	-1.50386800	2.66238800
H	-3.94910700	-2.99665200	-1.24898000
H	-4.58055200	-2.56664600	1.11718800

11

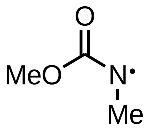

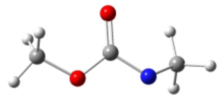

Cartesian Coordinates

N	-1.17971100	-0.61629900	-0.37419300
C	-0.01091400	0.09803100	-0.13175400
C	-2.36314300	-0.09547000	0.25967500
O	0.06433300	1.31687500	-0.11338000
H	-2.58032800	-0.72599100	1.13882800
H	-2.25398000	0.94309500	0.59350300
H	-3.21689500	-0.19760300	-0.41873700
O	1.03829500	-0.73512100	-0.02185000
C	2.30859700	-0.09033600	0.16478300
H	3.02769000	-0.89983800	0.29161500
H	2.56165000	0.51496100	-0.70982900
H	2.29158600	0.55208500	1.04958400

12	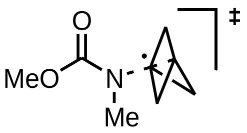	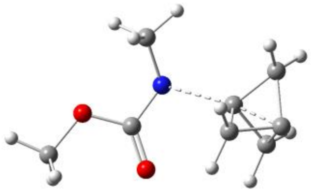

Cartesian Coordinates

C	2.91458900	-0.67362900	0.31216600
C	1.70932300	-0.72599900	1.23558000
C	1.55930300	0.08606700	-0.04444100
C	2.86861200	0.83888900	0.12116600
C	2.12595700	-0.98632800	-0.95274500
H	1.57515200	-1.92154400	-0.99420900
H	2.55131500	-0.65883600	-1.89910900
H	2.98735800	1.45674700	1.00938400
H	3.33103400	1.24210800	-0.77752600
H	1.77574800	-0.17530300	2.17230700
H	1.14966500	-1.65640100	1.28650900
N	-0.53310700	0.89007400	-0.68593300
C	-0.65776400	2.14920000	0.01893500
H	-1.58020100	2.64519600	-0.31693600
H	-0.71744800	2.05620300	1.11176200
H	0.18795300	2.78758300	-0.25004300
C	-1.37296600	-0.14489700	-0.31299100
O	-1.12223800	-1.33227600	-0.43025100
O	-2.58133500	0.31227700	0.13333700
C	-3.55867400	-0.70826700	0.37541300
H	-3.22451500	-1.38413500	1.16753800
H	-4.46375900	-0.18017500	0.67955200
H	-3.74224600	-1.29218100	-0.53087500

13	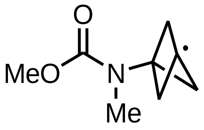	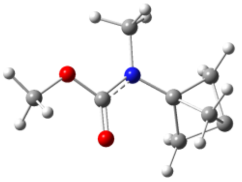

Cartesian Coordinates

C	2.83913900	-0.62557600	0.07238500
C	1.78844300	-0.69495300	1.19967500
C	1.17152000	0.05338300	-0.03136800
C	2.49036100	0.86278800	-0.16835600
C	1.77452000	-1.04294200	-0.96424600
H	1.33946400	-2.03715100	-0.87270700
H	1.94969900	-0.74618200	-2.00193800
H	2.72622400	1.56358200	0.63712600
H	2.71646400	1.27019900	-1.15766500
H	1.97659400	-0.09059200	2.09143700
H	1.35740400	-1.67012600	1.42856100
N	-0.15686700	0.63532200	-0.13372000
C	-0.30976000	2.08036100	0.01661600

H	-1.26429300	2.38956600	-0.40685000
H	-0.27386500	2.39527600	1.06877600
H	0.49579300	2.58162300	-0.52507500
C	-1.21704100	-0.23110300	-0.04602500
O	-1.12446100	-1.44698300	-0.01620300
O	-2.40648100	0.43140000	-0.00837400
C	-3.55765200	-0.42142000	0.04024100
H	-3.52744500	-1.06367100	0.92423800
H	-4.41455000	0.25234400	0.08546800
H	-3.61306900	-1.05068700	-0.85223500

14	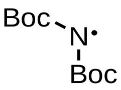	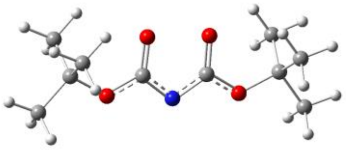

Cartesian Coordinates

N	-0.00000500	0.91230800	-0.00019400
C	-1.08703400	0.13598300	-0.00020100
C	1.08703000	0.13589200	-0.00012600
O	-1.05695600	-1.14217700	-0.00014200
O	-2.24666300	0.78772100	-0.00027000
C	-3.55942400	0.10660400	0.00007600
C	-3.71566400	-0.72659200	1.27521300
C	-3.71609700	-0.72710200	-1.27469100
C	-4.52922200	1.29084000	-0.00004100
H	-3.01766200	-1.56645200	-1.29475000
H	-4.73590600	-1.12408200	-1.32739500
H	-3.54680500	-0.10486300	-2.15986000
H	-3.54613200	-0.10403200	2.16010500
H	-4.73541900	-1.12366300	1.32837200
H	-3.01719900	-1.56592000	1.29530800
H	-4.37744800	1.91308800	0.88718400
H	-4.37775700	1.91259500	-0.88766700
H	-5.56207000	0.92785100	0.00024000
O	1.05692000	-1.14221000	0.00008200
O	2.24664300	0.78766800	-0.00022100
C	3.55944100	0.10661000	0.00004600
C	4.52920800	1.29087300	-0.00021200
C	3.71592300	-0.72651700	1.27518700
C	3.71590600	-0.72715500	-1.27469500
H	4.73566000	-1.12426300	-1.32749300
H	3.01739400	-1.56645300	-1.29460300
H	3.54657700	-0.10496100	-2.15988400
H	4.73568300	-1.12358200	1.32814000
H	3.54654500	-0.10389700	2.16006400
H	3.01742500	-1.56580600	1.29549100
H	5.56207400	0.92792800	0.00010800
H	4.37773700	1.91255000	-0.88788800
H	4.37739200	1.91318200	0.88696300

15	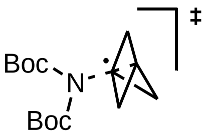	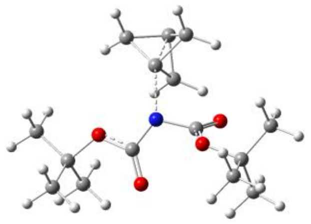

Cartesian Coordinates

C	-1.58544900	3.76603200	-0.23643600
C	-0.33771200	3.49246500	0.58387200
C	-1.20673400	2.30073500	0.21954600
C	-2.51405100	2.93829100	0.64373300
C	-1.33611700	2.66557000	-1.25136900
H	-0.41383400	2.69779600	-1.82400400
H	-2.20082900	2.27792300	-1.78669500
H	-2.61378000	3.23634400	1.68517600
H	-3.42785100	2.56113300	0.18936400
H	-0.34401400	3.81211900	1.62384100
H	0.62358200	3.56448900	0.08330300
N	0.17378700	0.02262900	0.69127500
C	-0.60836600	-0.97286800	0.10009800
C	1.41452200	0.26890200	0.06284200
O	1.58628300	1.14399900	-0.76227100
O	2.33417700	-0.53509900	0.59698300
C	3.72245000	-0.57910300	0.07700700
C	3.70073300	-0.95786500	-1.40633800
C	4.41122500	0.76354200	0.33594100
C	4.34938100	-1.69173200	0.91871100
H	3.10834900	-1.86646500	-1.55422600
H	3.27405000	-0.15827700	-2.01452500
H	4.72373100	-1.15043800	-1.74857000
H	5.46790000	0.69142800	0.05416300
H	3.94742600	1.56276000	-0.24455100
H	4.35984800	1.02066500	1.39944400
H	4.30093600	-1.44448900	1.98397400
H	3.82393000	-2.63842000	0.75943700
H	5.40014200	-1.82426600	0.63997300
O	-0.13914600	-1.86695800	-0.59241800
O	-1.88356400	-0.84555900	0.47831200
C	-2.91177100	-1.80766200	0.03374800
C	-2.59554400	-3.19876800	0.59276300
C	-4.18390800	-1.24215300	0.66911200
C	-3.01480400	-1.80075400	-1.49486500
H	-3.42301100	-3.87984600	0.36418400
H	-1.68009700	-3.60231600	0.15715700
H	-2.48183900	-3.15434700	1.68123300
H	-4.08508400	-1.20025500	1.75827700
H	-4.38134500	-0.22983700	0.30234200
H	-5.04170600	-1.87578500	0.42086100
H	-3.18559400	-0.78237300	-1.86069800

H	-2.10870300	-2.19499100	-1.95803200
H	-3.86384300	-2.41980200	-1.80538700

16	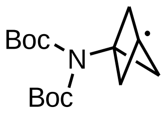	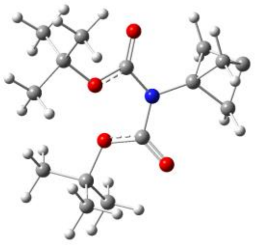

Cartesian Coordinates

C	-4.25486400	-0.19559400	0.09456100
C	-3.33506500	0.15110500	1.28352400
C	-2.46153900	-0.11064300	0.01528300
C	-3.32168500	-1.36410600	-0.29488600
C	-3.44942800	0.75416900	-0.82201300
H	-3.47593300	1.81880100	-0.58867800
H	-3.47716300	0.54802000	-1.89492600
H	-3.24144900	-2.19461100	0.40998300
H	-3.33423600	-1.69783300	-1.33203200
H	-3.25754000	-0.59215200	2.08163200
H	-3.34562300	1.17589000	1.65262300
N	-0.99131800	-0.04979100	-0.03442300
C	-0.27306700	-1.23651000	-0.32162500
C	-0.42400200	1.21529600	0.18111400
O	-1.06389800	2.13304400	0.65934600
O	0.83397300	1.28259600	-0.27792100
C	1.63070100	2.51755500	-0.11925600
C	0.99391100	3.65524700	-0.92403500
C	1.78031100	2.86034900	1.36595500
C	2.97867700	2.11753700	-0.72379700
H	0.86682300	3.35642700	-1.97031600
H	0.02188600	3.93216900	-0.51406400
H	1.65097400	4.53190400	-0.89886100
H	2.48015100	3.69603300	1.47820800
H	0.82332600	3.14480100	1.80593300
H	2.18285800	2.00234100	1.91515300
H	3.42269000	1.28659100	-0.16636300
H	2.85829700	1.81016900	-1.76762500
H	3.67074900	2.96543100	-0.69033500
O	-0.70028700	-2.09614800	-1.06417900
O	0.85980500	-1.29415400	0.38802300
C	1.83862700	-2.38101800	0.16912400
C	2.30950400	-2.37835700	-1.28821900
C	2.97794700	-1.98991000	1.11303700
C	1.22639100	-3.72219700	0.58579800
H	3.12819500	-3.09667400	-1.40765800
H	1.50183700	-2.65570000	-1.96768000
H	2.68182900	-1.38644600	-1.56529500
H	3.39328300	-1.01566100	0.83682000

H	2.62133500	-1.93293000	2.14645100
H	3.77815100	-2.73561300	1.06280800
H	0.85402900	-3.66712800	1.61461400
H	0.40435200	-4.00336300	-0.07368800
H	1.99513200	-4.50216600	0.54470900

17	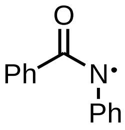	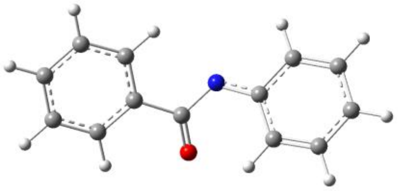

Cartesian Coordinates

N	0.50656400	0.39264600	-0.62581500
C	-0.45264900	-0.61699100	-0.57120800
O	-0.21819900	-1.78543600	-0.87832800
C	1.78011900	0.20374800	-0.23857200
C	2.69872400	1.26245600	-0.53025600
C	2.27311200	-0.93683600	0.47838200
C	4.02185000	1.18621800	-0.13675400
H	2.31293600	2.12069900	-1.07108000
C	3.59967400	-0.99279700	0.86541600
H	1.59661700	-1.75402900	0.69390500
C	4.48108400	0.05934000	0.56407600
H	4.70644500	1.99746800	-0.36770500
H	3.96481000	-1.86031400	1.40831000
H	5.52034200	0.00025900	0.87512400
C	-1.82595300	-0.15542900	-0.20326100
C	-2.12476000	1.19677800	0.01896500
C	-2.83402600	-1.12002900	-0.05936100
C	-3.41552600	1.57609500	0.38479100
H	-1.34383400	1.93869800	-0.10813300
C	-4.12207500	-0.73839700	0.30493000
H	-2.58364900	-2.16060200	-0.23885000
C	-4.41452300	0.61063300	0.52871700
H	-3.64386100	2.62514800	0.55296500
H	-4.89975900	-1.48939400	0.41510100
H	-5.42071300	0.90829900	0.81249700

18	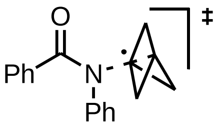	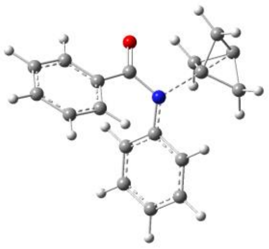

Cartesian Coordinates

C	3.78678300	-1.88548100	-0.00001000
C	2.91755400	-1.45647100	1.17842500
C	2.34849900	-1.17148800	-0.20394700
C	3.62357300	-0.55240600	-0.73985000

C	2.63485300	-2.55856200	-0.74857600
H	2.12761700	-3.38549800	-0.26011800
H	2.73597100	-2.65565600	-1.82832700
H	3.99518900	0.34153000	-0.24203000
H	3.77286000	-0.56345300	-1.81844000
H	3.25737000	-0.60022100	1.75920100
H	2.41952400	-2.23273800	1.75267500
N	0.44232500	-0.25095800	-0.43432000
C	-0.46932800	-1.12565400	0.14020700
O	-0.15810600	-2.18166300	0.68294500
C	-1.93059600	-0.78712500	-0.06135200
C	-2.38618600	0.15586300	-0.99337100
C	-2.86453300	-1.49931700	0.70516300
C	-3.75264600	0.38946100	-1.14539400
H	-1.67494300	0.69672800	-1.60787800
C	-4.22836300	-1.25731500	0.55892300
H	-2.49728300	-2.24240000	1.40508900
C	-4.67586700	-0.31130200	-0.36690300
H	-4.09639100	1.11708200	-1.87577500
H	-4.94339200	-1.80923800	1.16335900
H	-5.74021100	-0.12494600	-0.48473800
C	0.53969400	1.08068100	-0.08762200
C	1.13715400	1.97068000	-1.01241800
C	0.11501600	1.59444900	1.16238400
C	1.28512900	3.31752000	-0.70598500
H	1.46763700	1.57244900	-1.96693200
C	0.25474700	2.94683600	1.45281000
H	-0.32553300	0.91931600	1.89006700
C	0.84138900	3.81494100	0.52538000
H	1.74177100	3.98806600	-1.42923300
H	-0.08685600	3.32767300	2.41173700
H	0.95400900	4.86946800	0.76084400

19	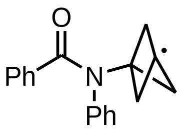	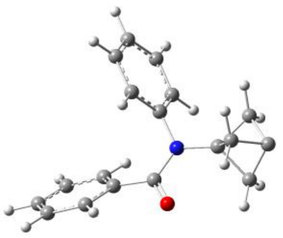

Cartesian Coordinates

C	3.83294300	-1.47235300	-0.09698300
C	2.90168600	-1.43031700	1.13135000
C	2.13146600	-0.88635900	-0.12042200
C	3.38219300	-0.04743100	-0.49497800
C	2.69854500	-2.07241200	-0.95672200
H	2.33283800	-3.06323000	-0.69266400
H	2.75903300	-1.91109100	-2.03655000
H	3.64734300	0.76802000	0.18161000
H	3.49249800	0.22910900	-1.54680600
H	3.14001800	-0.69808300	1.90744500

H	2.54714000	-2.37990400	1.53108200
N	0.74752800	-0.38885700	-0.15659200
C	-0.24567500	-1.33191000	0.10008000
O	0.03984000	-2.48525400	0.40621600
C	0.55634400	1.01923000	0.04515800
C	0.89648600	1.92312700	-0.96918600
C	0.06895400	1.50330300	1.26511000
C	0.74559100	3.29427900	-0.76516700
H	1.26803800	1.54286200	-1.91582100
C	-0.09649500	2.87500200	1.45848300
H	-0.18232900	0.80204400	2.05514500
C	0.24453900	3.77429200	0.44704200
H	1.01119400	3.98765500	-1.55853400
H	-0.48339700	3.24004300	2.40594400
H	0.12133400	4.84250600	0.60185600
C	-1.68662900	-0.93257700	-0.06450700
C	-2.13716900	-0.03277100	-1.04023800
C	-2.62466100	-1.59319100	0.74197800
C	-3.50152900	0.21254700	-1.19291500
H	-1.42642300	0.46647200	-1.68931300
C	-3.98548700	-1.33179200	0.60178200
H	-2.26809300	-2.31722800	1.46741800
C	-4.42738900	-0.42736800	-0.36695900
H	-3.84100300	0.90362300	-1.95972600
H	-4.70236100	-1.84026600	1.24085000
H	-5.48951600	-0.22829300	-0.48296800

20	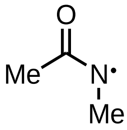	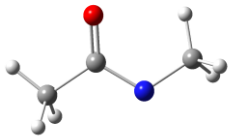

Cartesian Coordinates

N	-0.63310900	-0.65668500	-0.29644100
C	0.46693500	0.14532700	-0.03404700
C	-1.91919200	-0.18128700	0.14222800
O	0.37286700	1.37208200	-0.03900700
H	-2.35519100	-0.95395400	0.79381600
H	-1.87969300	0.77762000	0.67095000
H	-2.59038300	-0.10429900	-0.72382800
C	1.77959700	-0.59096700	0.10324700
H	2.08863100	-0.97651900	-0.87523600
H	2.54501000	0.09038800	0.47986300
H	1.67641600	-1.45153100	0.77301500

21	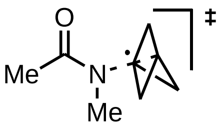	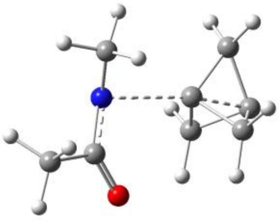

Cartesian Coordinates

C	2.68237400	-0.40471500	0.18902400
C	1.58765500	-0.49480400	1.23802300
C	1.22223700	0.16663600	-0.08372700
C	2.44556300	1.06778300	-0.12585900
C	1.81999400	-0.92308700	-0.95280000
H	1.38014800	-1.91084500	-0.85346000
H	2.11270200	-0.64583100	-1.96349300
H	2.57800500	1.77824200	0.68814600
H	2.77017600	1.42831900	-1.09993000
H	1.68174400	0.15041000	2.10992700
H	1.14311000	-1.46748100	1.42886700
N	-1.06442400	0.61618800	-0.54314600
C	-1.75071200	-0.49824400	-0.10190100
O	-1.24648600	-1.60715200	0.02313000
C	-3.25440900	-0.29186700	0.12050000
C	-1.29766100	1.90456700	0.06958400
H	-0.55770100	2.61509700	-0.30806600
H	-2.29027200	2.27879600	-0.22764600
H	-1.25872000	1.89909300	1.16908100
H	-3.71157400	0.29695000	-0.68159000
H	-3.72656600	-1.27440300	0.17276200
H	-3.42844100	0.23794000	1.06531500

22	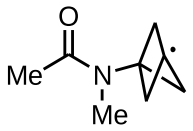	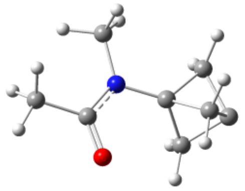

Cartesian Coordinates

C	2.53659400	-0.36384600	0.03949100
C	1.52044700	-0.59054400	1.17876000
C	0.79302200	0.09445700	-0.02711100
C	1.99449700	1.07055200	-0.16540600
C	1.51868100	-0.89691800	-0.99096400
H	1.21705000	-1.94005500	-0.91378100
H	1.63755900	-0.55997500	-2.02448500
H	2.15282100	1.78072300	0.65104600
H	2.15254200	1.52193600	-1.14902300
H	1.64291600	0.01661700	2.08013600
H	1.22194200	-1.61666800	1.39265200
N	-0.60602000	0.49702700	-0.09902400
C	-0.91343000	1.91687200	0.03150700
H	-1.90221000	2.13184200	-0.37546400
H	-0.88580300	2.25935800	1.07623300
H	-0.18534500	2.49928300	-0.53820700
C	-1.55436600	-0.50192500	-0.01653100
O	-1.23682400	-1.68501300	0.03319200
C	-3.01979000	-0.08210800	-0.00837000
H	-3.61675600	-0.99281400	0.05066800
H	-3.25860500	0.55798800	0.84863700

H	-3.29331100	0.46343700	-0.91904300

23	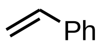	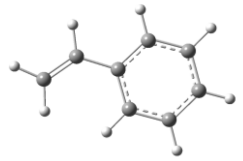

Cartesian Coordinates

C	-1.95473600	-0.52923300	-0.00000300
C	-2.97743100	0.33497900	0.00000700
H	-2.84062400	1.41305800	0.00001600
H	-4.00449700	-0.01691600	0.00000600
H	-2.18619600	-1.59465800	-0.00001300
C	-0.51524800	-0.22036100	-0.00000400
C	0.40644100	-1.28137000	-0.00000400
C	-0.00877100	1.09240000	-0.00000200
C	1.78076600	-1.04621600	0.00000500
H	0.03489000	-2.30382800	-0.00000400
C	1.36236500	1.32955000	-0.00000400
H	-0.69383000	1.93533800	-0.00000700
C	2.26535200	0.26181100	0.00000300
H	2.47187300	-1.88506800	0.00001500
H	1.73045000	2.35231700	-0.00000100
H	3.33550400	0.45040100	0.00000600

24	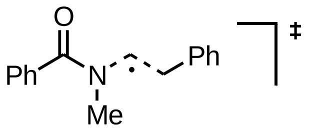	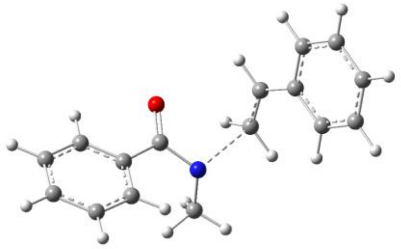

Cartesian Coordinates

C	-1.29266700	2.09869500	-0.02154500
N	0.61851300	1.08245000	0.72689500
C	1.15959800	2.01919500	1.69351100
H	0.48519600	2.09591500	2.55189700
H	2.13742100	1.66729600	2.05457500
H	1.31649900	3.02542000	1.27370600
C	1.38594600	0.82211200	-0.40581000
C	2.59588500	-0.05025500	-0.17310200
C	2.73368700	-0.86774300	0.95790400
C	3.60118000	-0.04986300	-1.15039300
C	3.86925400	-1.66283500	1.11382100
H	1.93847600	-0.89661900	1.69621300
C	4.73757400	-0.83858700	-0.98853100
H	3.46837500	0.57346200	-2.02863500
C	4.87446800	-1.64529900	0.14480100
H	3.96551700	-2.30204800	1.98743200
H	5.51619000	-0.82833500	-1.74667300
H	5.75960900	-2.26386500	0.26838000

O	1.09224100	1.19312900	-1.53756600
H	-1.57968000	2.28394900	1.00837400
H	-0.64685300	2.83060500	-0.49026900
C	-1.95885900	1.20140300	-0.80408500
H	-1.68589300	1.16107400	-1.85604200
C	-2.96466300	0.24095500	-0.37443600
C	-3.62585000	-0.53584300	-1.34819100
C	-3.31577500	0.04180500	0.97793300
C	-4.60405200	-1.45999300	-0.99221300
H	-3.36230600	-0.40217600	-2.39468300
C	-4.29051800	-0.88365900	1.33299400
H	-2.80839300	0.60684800	1.75389400
C	-4.94254100	-1.63766800	0.35105600
H	-5.10104600	-2.04437900	-1.76199900
H	-4.54288200	-1.02378300	2.38089500
H	-5.70374500	-2.36005800	0.63291100

25

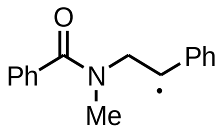

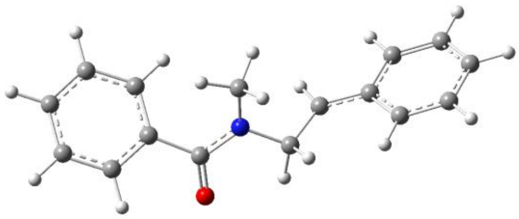

Cartesian Coordinates

C	0.76967000	-1.01174100	-0.20484100
N	-0.50631100	-0.39544600	0.18571900
C	-0.45588800	0.43475900	1.38445800
H	-1.46068900	0.66564400	1.73352100
H	0.07243600	-0.10351700	2.18357400
H	0.08244400	1.37161800	1.19895800
C	-1.66126300	-0.88832100	-0.38679500
C	-2.96386100	-0.18419600	-0.10831100
C	-3.09913300	1.21037400	-0.14750000
C	-4.10561600	-0.97673000	0.07239900
C	-4.35283900	1.80200700	0.01321700
H	-2.22723800	1.83325800	-0.32685700
C	-5.35353000	-0.38474600	0.25392400
H	-3.99892100	-2.05676600	0.05819800
C	-5.48005300	1.00655100	0.22501800
H	-4.44862300	2.88359100	-0.03142000
H	-6.23023700	-1.00827800	0.40703900
H	-6.45499700	1.46810800	0.35728800
O	-1.65146000	-1.85629300	-1.14591400
H	1.15099900	-1.60654100	0.63584700
H	0.52614500	-1.71451300	-1.01015600
C	1.76560400	0.01119100	-0.65692800
H	1.38684500	0.74719100	-1.36435200
C	3.13305200	0.08909700	-0.30377800
C	3.77544300	-0.81903100	0.59009000
C	3.94229600	1.12520600	-0.86152600

C	5.12174400	-0.68938300	0.89840900
H	3.20717500	-1.62942300	1.03554300
C	5.28558500	1.24488400	-0.54573000
H	3.48137200	1.82991500	-1.54971500
C	5.89023800	0.33947500	0.33806200
H	5.58353900	-1.39726600	1.58212900
H	5.87295100	2.04569500	-0.98769100
H	6.94395700	0.43338400	0.58436900

9.5 Electronic Properties of Propellane and Alkenes

DFT Method: UB3LYP/6-31+G(d)

species	Ionization Potential (IP, eV)	Electron affinity (EA, eV)	Electronegativity (χ, eV)	Electronic Chemical Potential (μ, eV)	Chemical Hardness (η, eV)	Chemical Softness (S, meV)
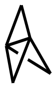	9.50	-1.24	4.13	-4.13	5.370	186.2
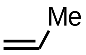	9.36	-1.48	3.94	-3.94	5.420	184.5
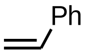	8.08	-0.13	3.97	-3.97	4.102	243.8

Computed Energies [values are in Hartree]

Species	Total Electronic Energy	Sum of Electronic and Zero-point Energies	Sum of Electronic and Thermal Enthalpies	Gibbs Free Energy
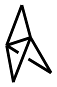	-194.0177723	-193.924090	-193.919192	-193.950388
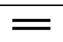	-78.5932689	-78.542171	-78.538185	-78.563051
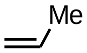	-117.9107905	-117.831375	-117.826891	-117.855969
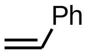	-309.6612743	-309.527757	-309.520057	-309.559043

Optimized Structures and Cartesian Coordinates

No.	Species	Optimized Structure
1	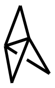	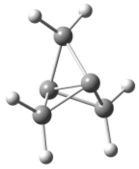

Cartesian Coordinates

C	0.00017000	-0.00007800	0.78889000
C	1.11633500	-0.66452800	-0.00022100
C	-0.00012100	-0.00005400	-0.78886900
C	-1.13376000	-0.63442700	0.00019100
C	0.01737500	1.29903800	0.00001300
H	0.94165800	1.87290000	-0.00014800
H	-0.89134700	1.89715900	0.00018900
H	-1.19747200	-1.72046300	0.00018600
H	-2.09276300	-0.12080600	0.00037800
H	1.15124700	-1.75185500	-0.00024200
H	2.08867600	-0.17663800	-0.00039100

No.	Species	Optimized Structure
3	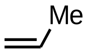	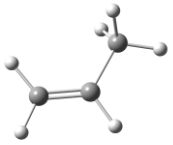

Cartesian Coordinates

C	0.00000000	0.48040500	0.00000000
C	-1.29642500	0.15340000	0.00000000
H	-1.62827400	-0.88447100	0.00000000
H	-2.07530400	0.91176000	0.00000000
H	0.26788400	1.53752300	0.00000000
C	1.14518100	-0.50841700	0.00000000
H	1.11414500	-1.15872800	0.88369300
H	1.11414500	-1.15872800	-0.88369300
H	2.11486900	0.00031800	0.00000000

No.	Species	Optimized Structure
4	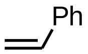	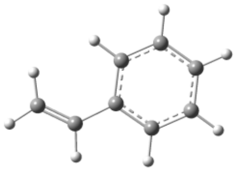

Cartesian Coordinates

C	-1.95667800	-0.52977300	0.00000300
C	-2.98231300	0.33556700	-0.00000200
H	-2.84700400	1.41425200	-0.00001000
H	-4.00871700	-0.01950100	0.00000100
H	-2.18894500	-1.59528100	0.00000500
C	-0.51584000	-0.22036500	0.00000000
C	0.40660700	-1.28250800	-0.00000200

C	-0.00861800	1.09342800	0.00000300
C	1.78305200	-1.04746000	0.00000000
H	0.03597500	-2.30565000	-0.00000200
C	1.36432900	1.33096700	-0.00000100
H	-0.69235900	1.93769800	0.00000500
C	2.26870300	0.26194500	-0.00000100
H	2.47400700	-1.88682100	0.00000600
H	1.73241000	2.35405300	0.00000500
H	3.33917600	0.45044300	-0.00000100

10 NMR Spectra

7 – ^{1}H NMR (500 MHz, $CDCl_3$)

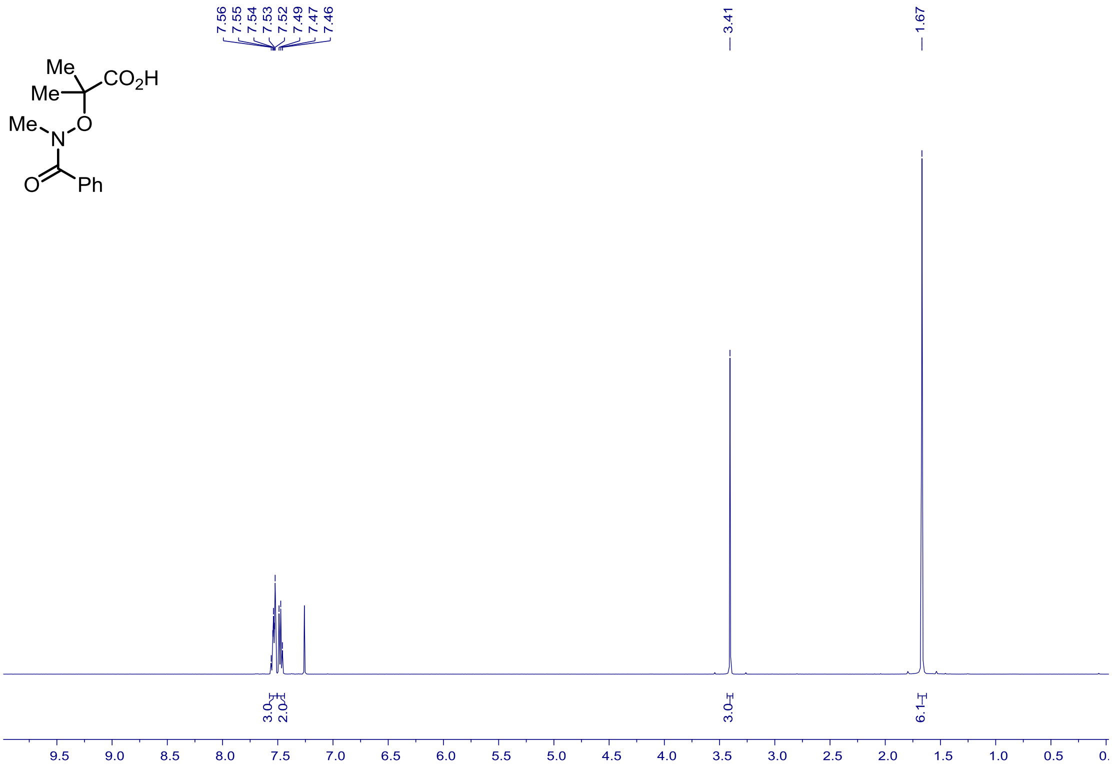

7 – ^{13}C NMR (126MHz, $CDCl_3$)

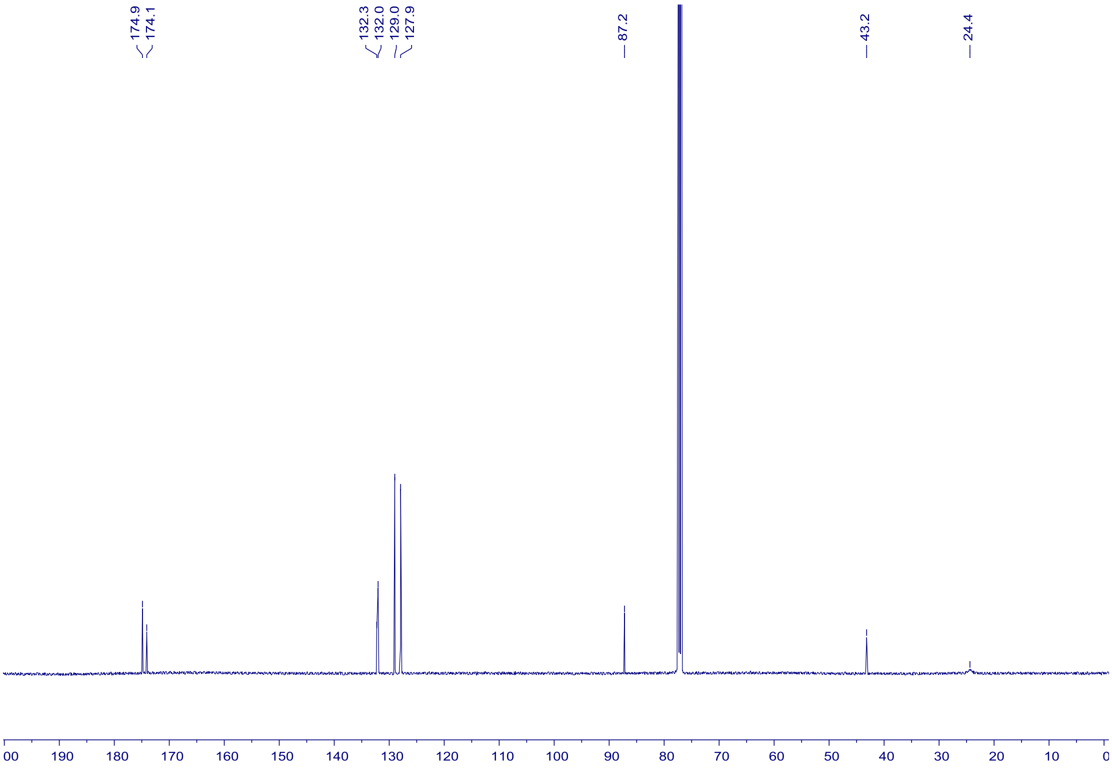

7 – HSQC (500 MHz, $CDCl_3$)

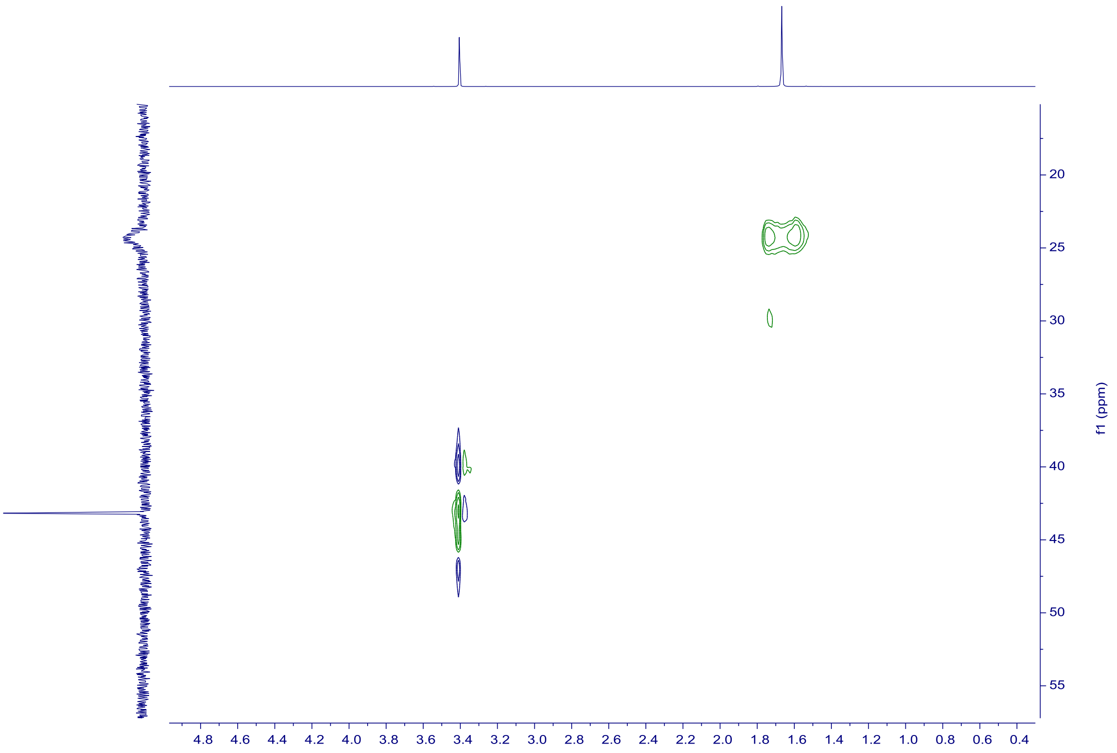

7 – HMBC (500 MHz, $CDCl_3$)

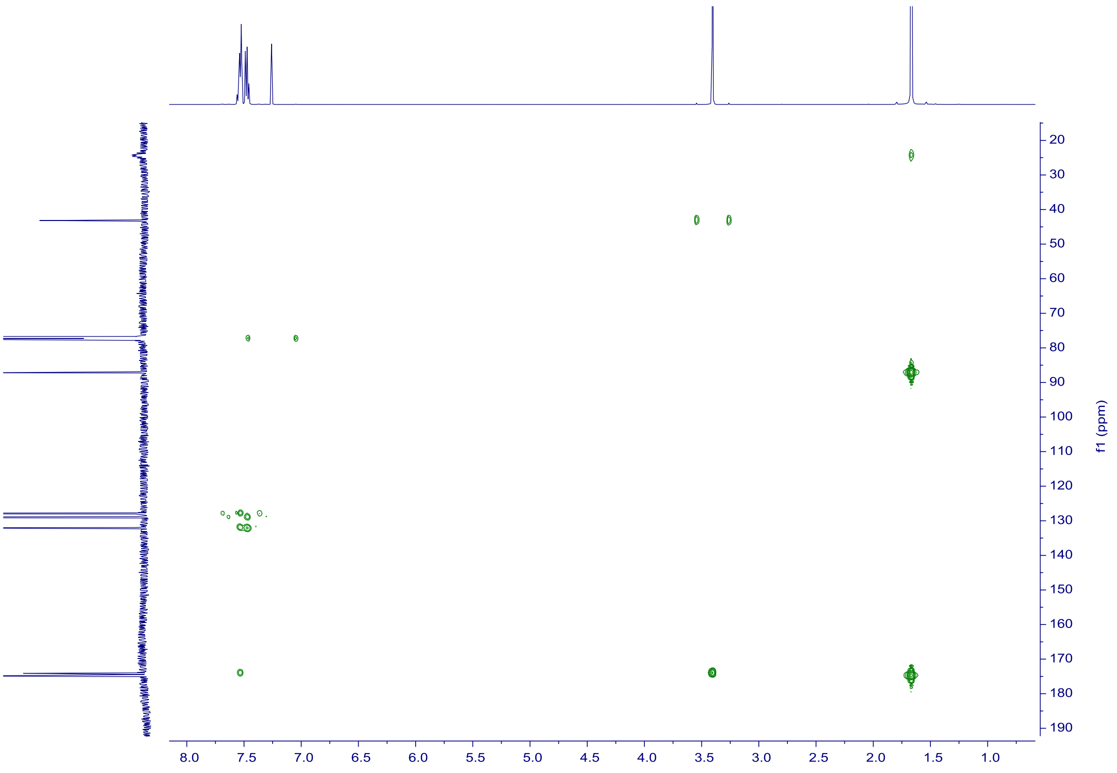

7a – ^{1}H NMR (400 MHz, $CDCl_3$)

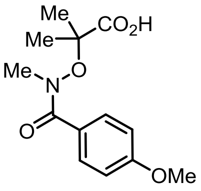

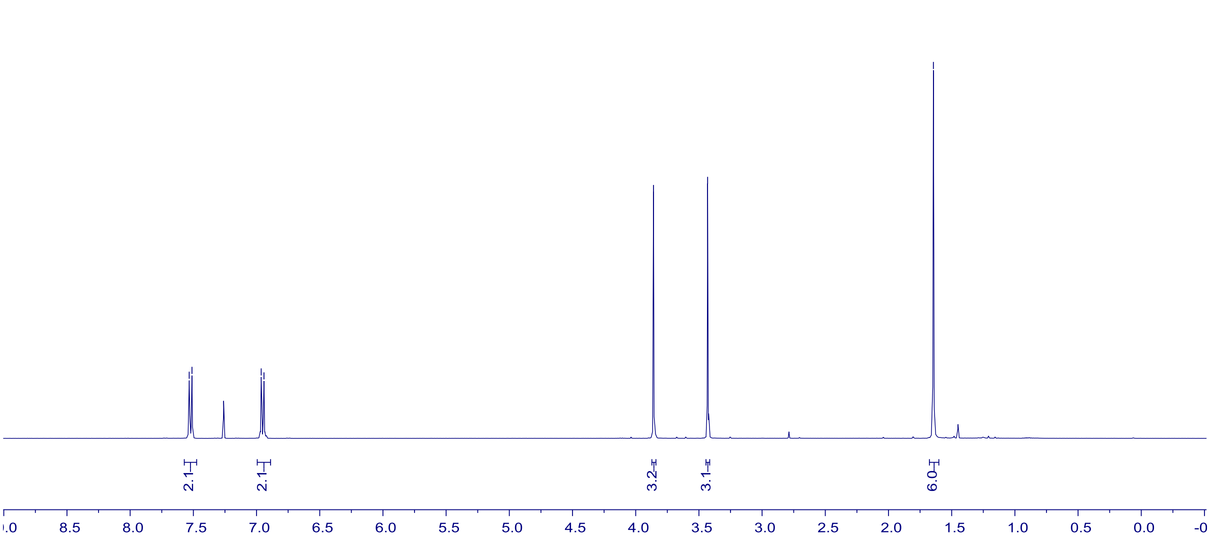

7a – ^{13}C NMR (101 MHz, $CDCl_3$)

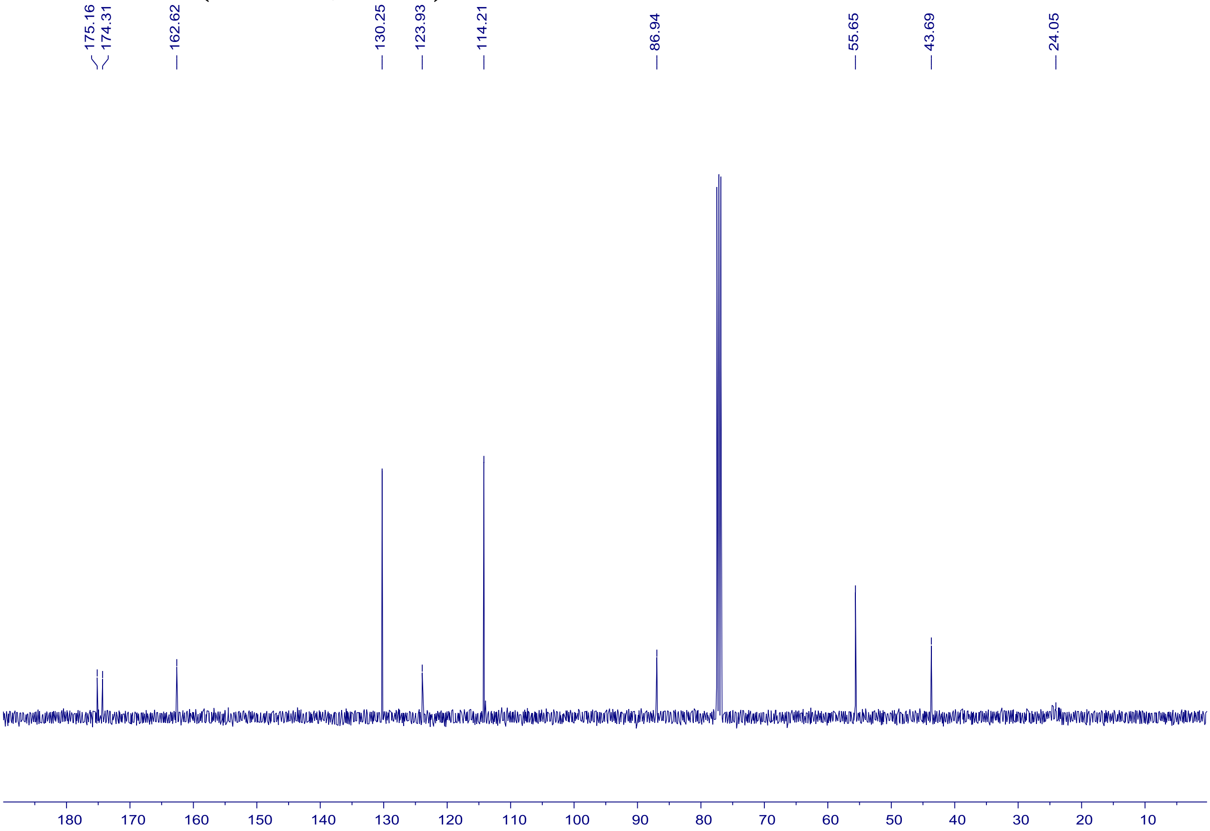

7b – ^{1}H NMR (500 MHz, $CDCl_3$)

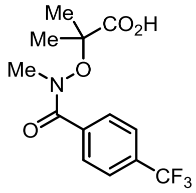

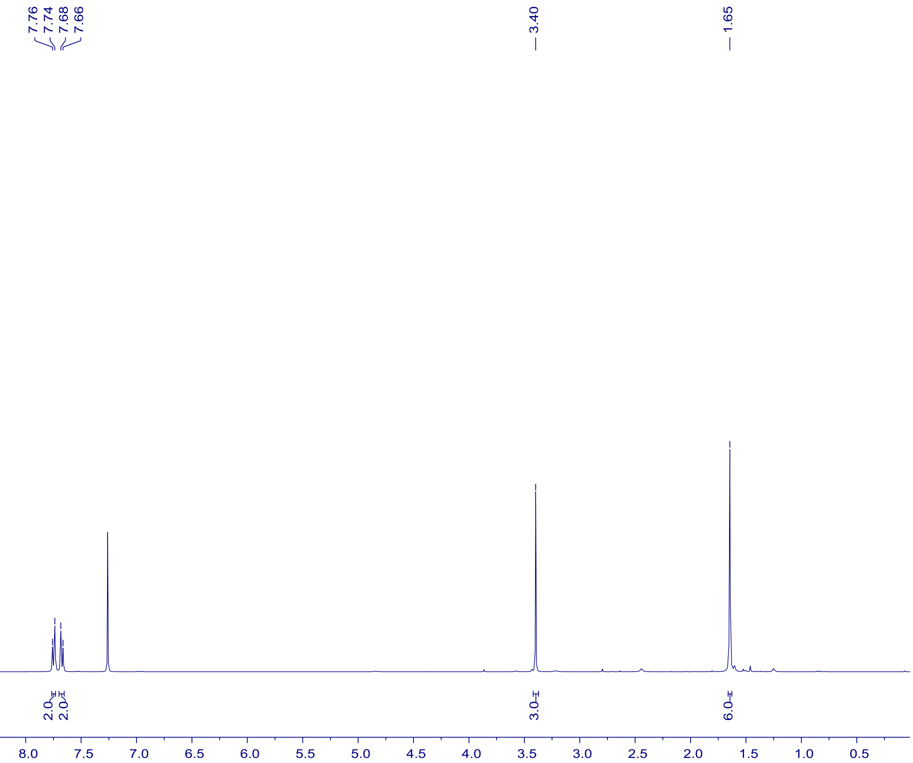

7b – ^{13}C NMR (126MHz, $CDCl_3$)

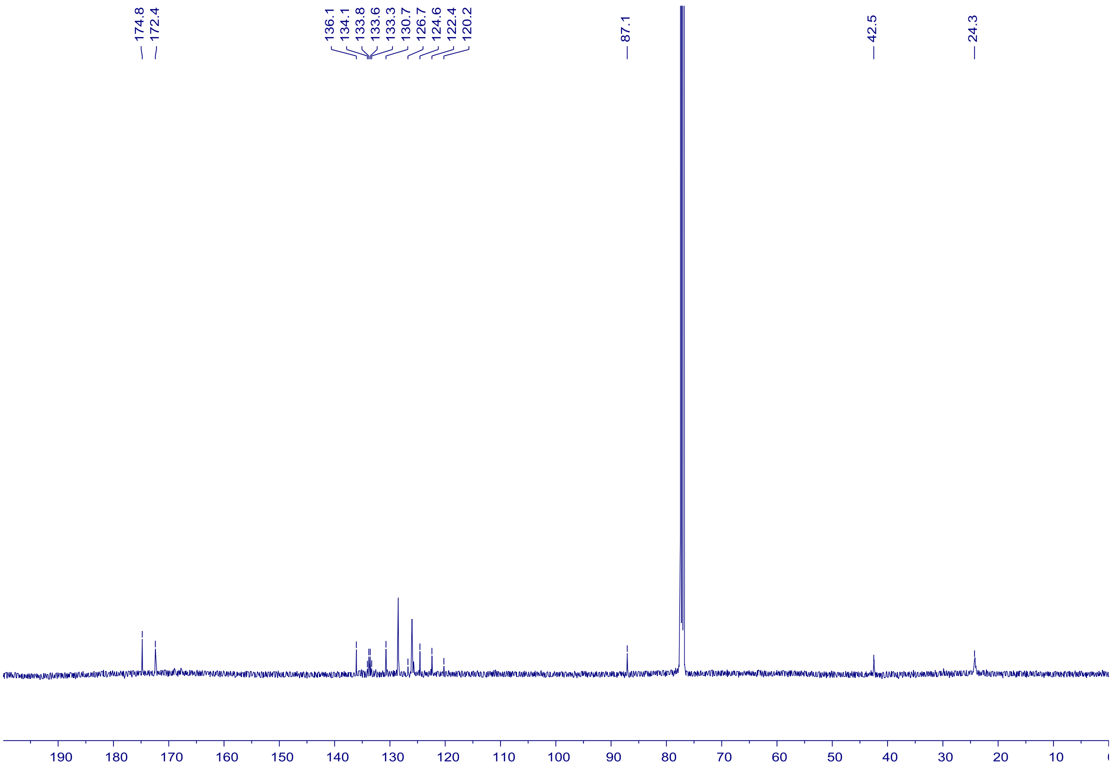

7b – ^{19}F NMR (376MHz, $CDCl_3$)

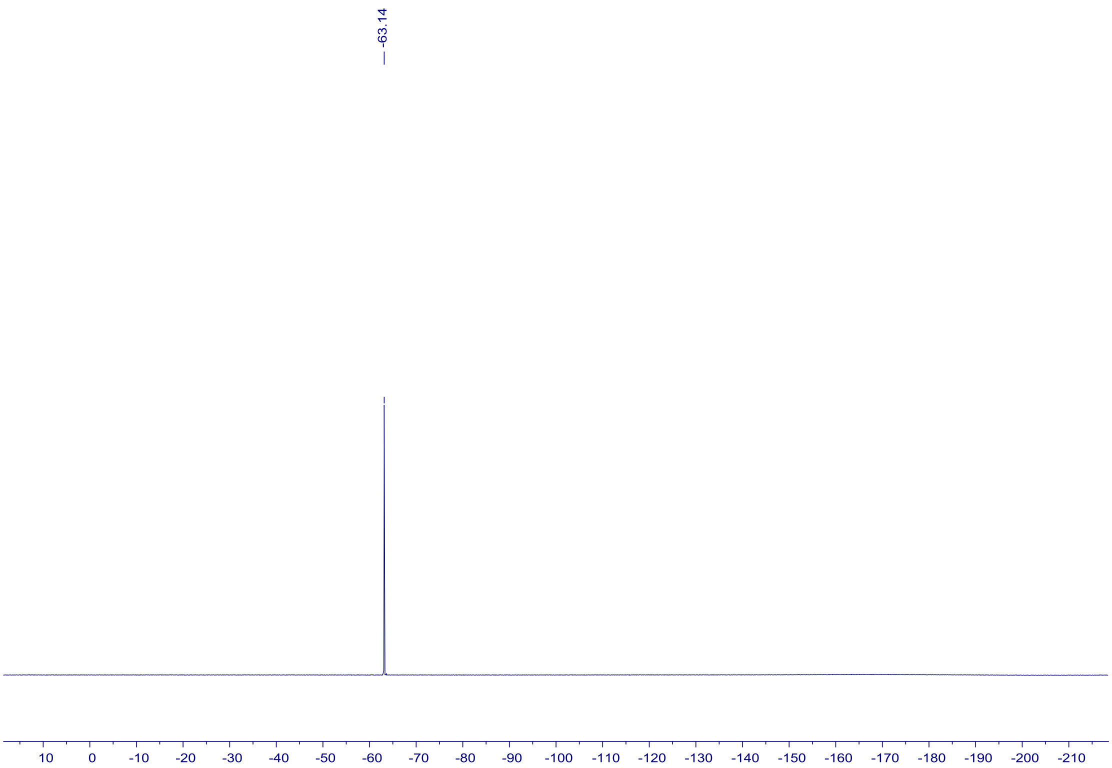

7c – ^{1}H NMR (400 MHz, $CDCl_3$)

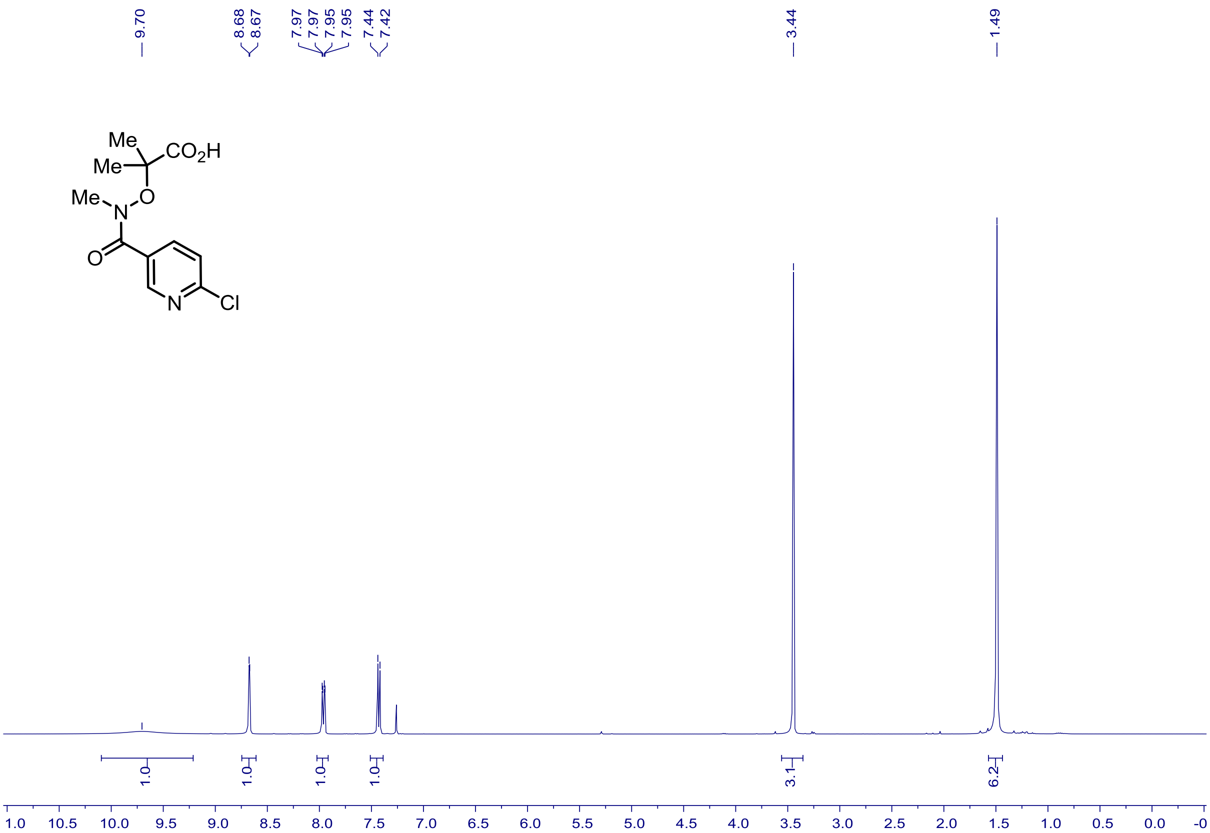

7c – ^{13}C NMR (101 MHz, $CDCl_3$)

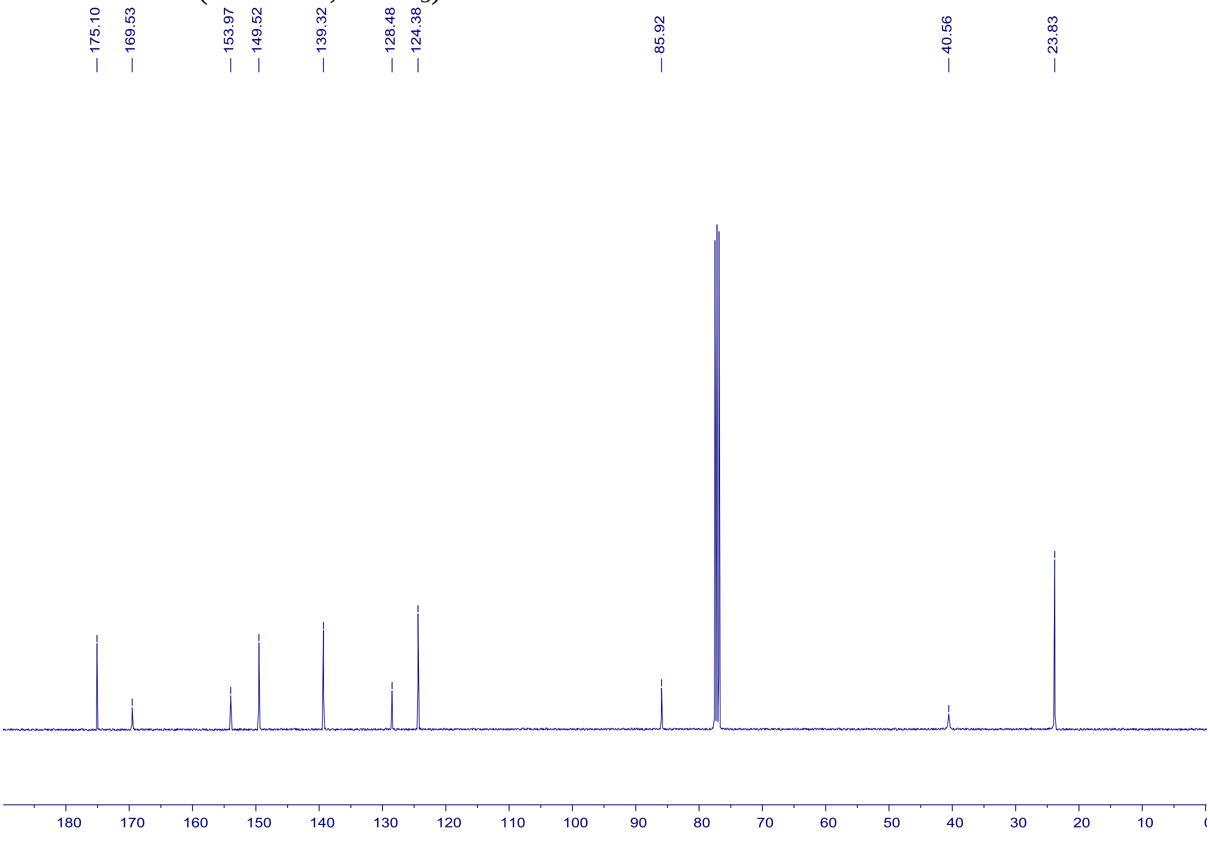

7d – ^{1}H NMR (500 MHz, $CDCl_3$)

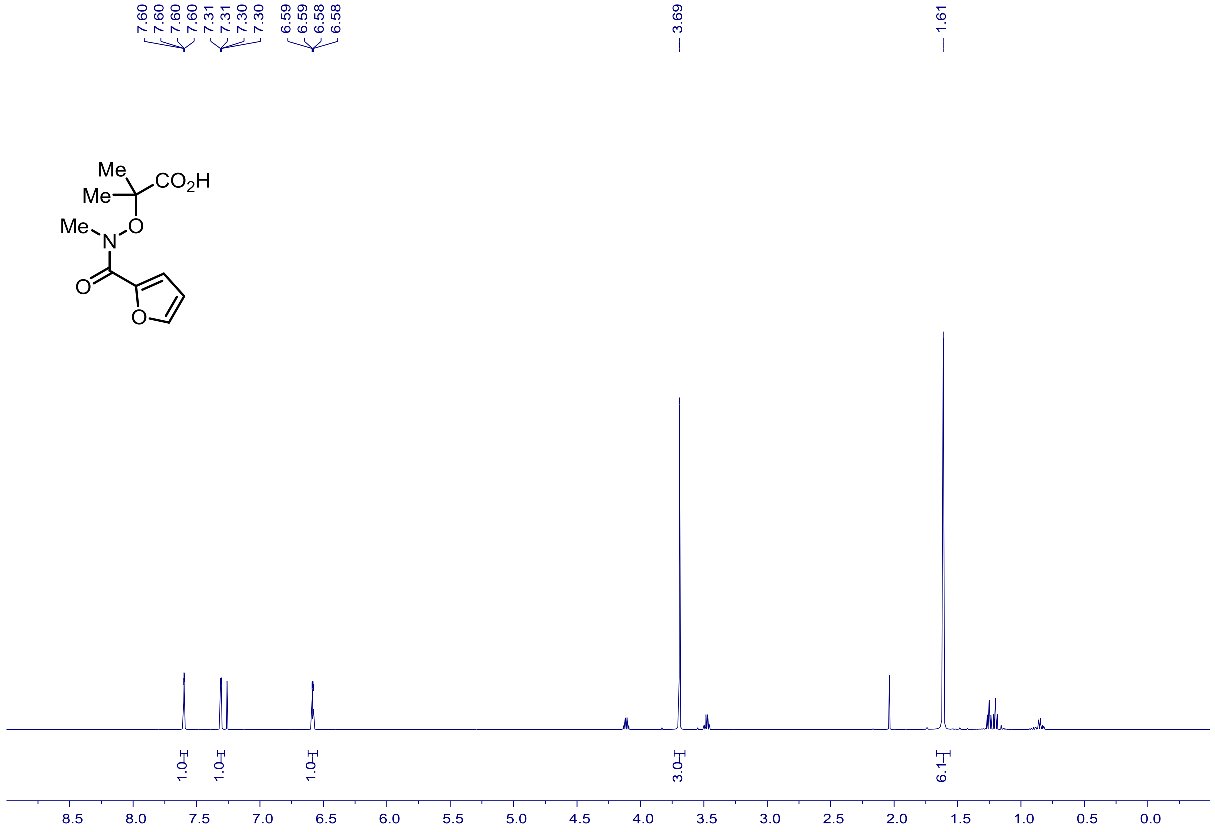

7d – ^{13}C NMR (126 MHz, $CDCl_3$)

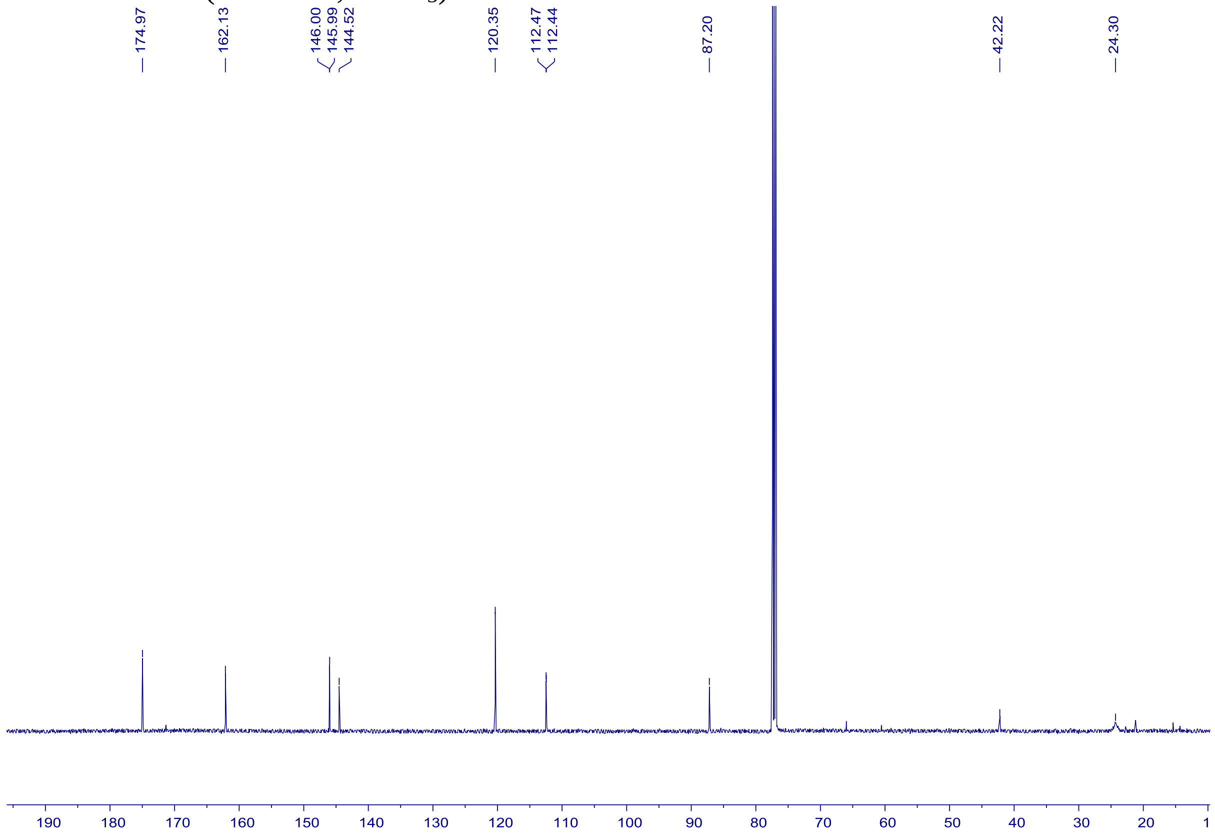

7e – ^{1}H NMR (500 MHz, $CDCl_3$)

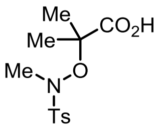

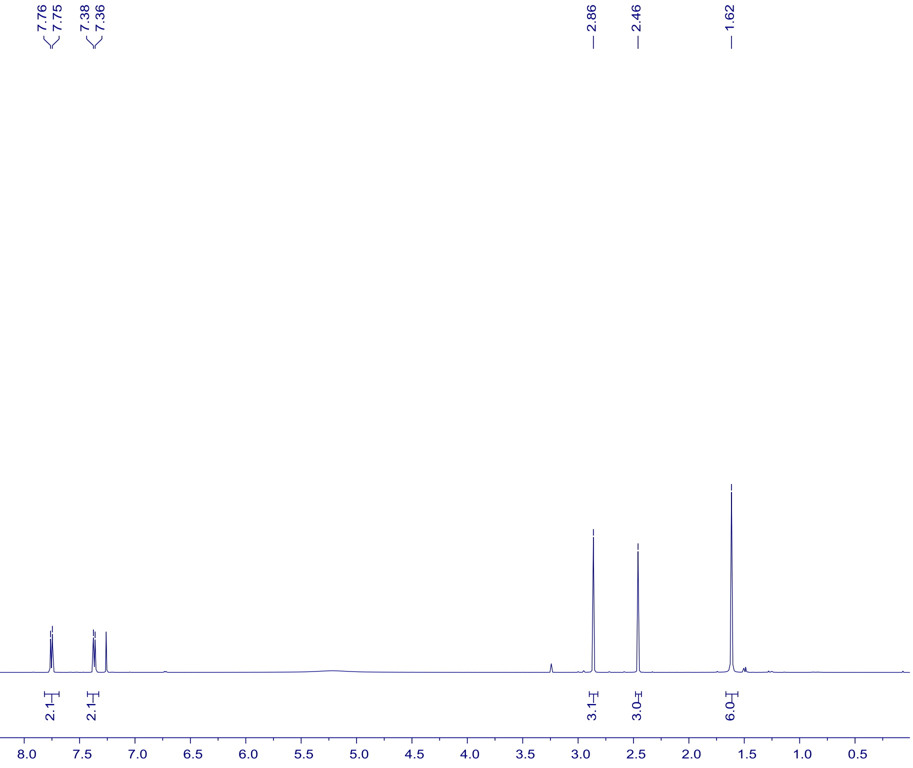

7e – ^{13}C NMR (126MHz, $CDCl_3$)

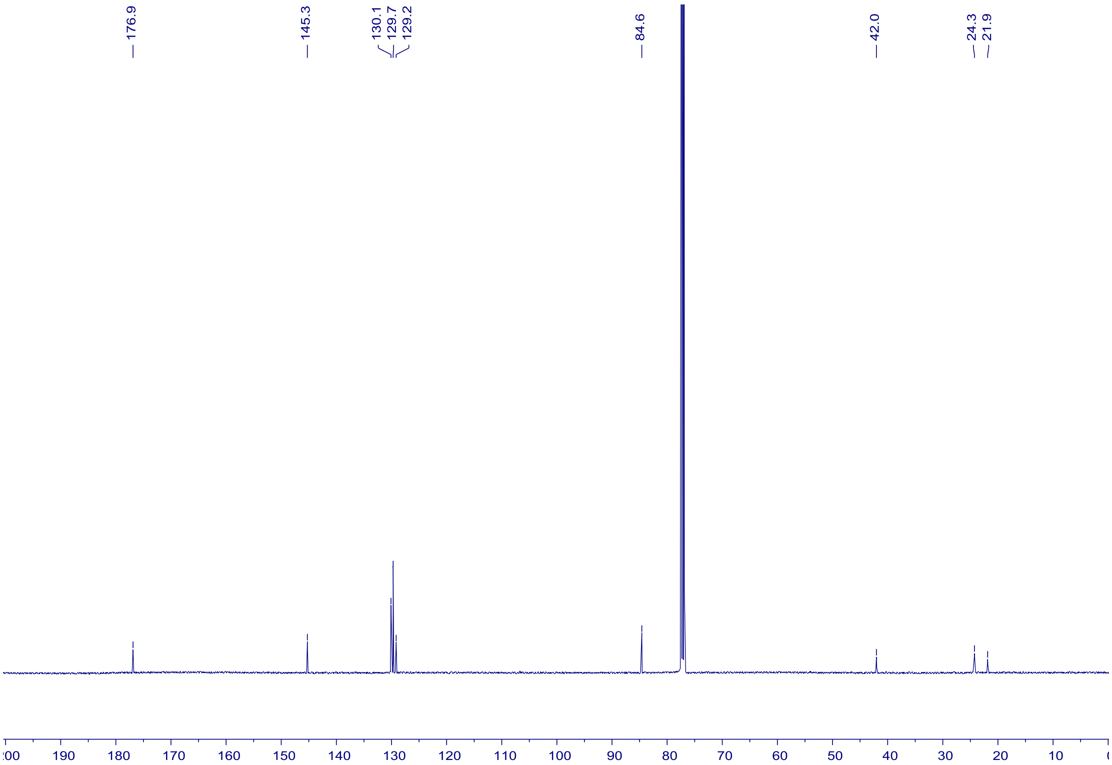

7e – HSQC (500 MHz, $CDCl_3$)

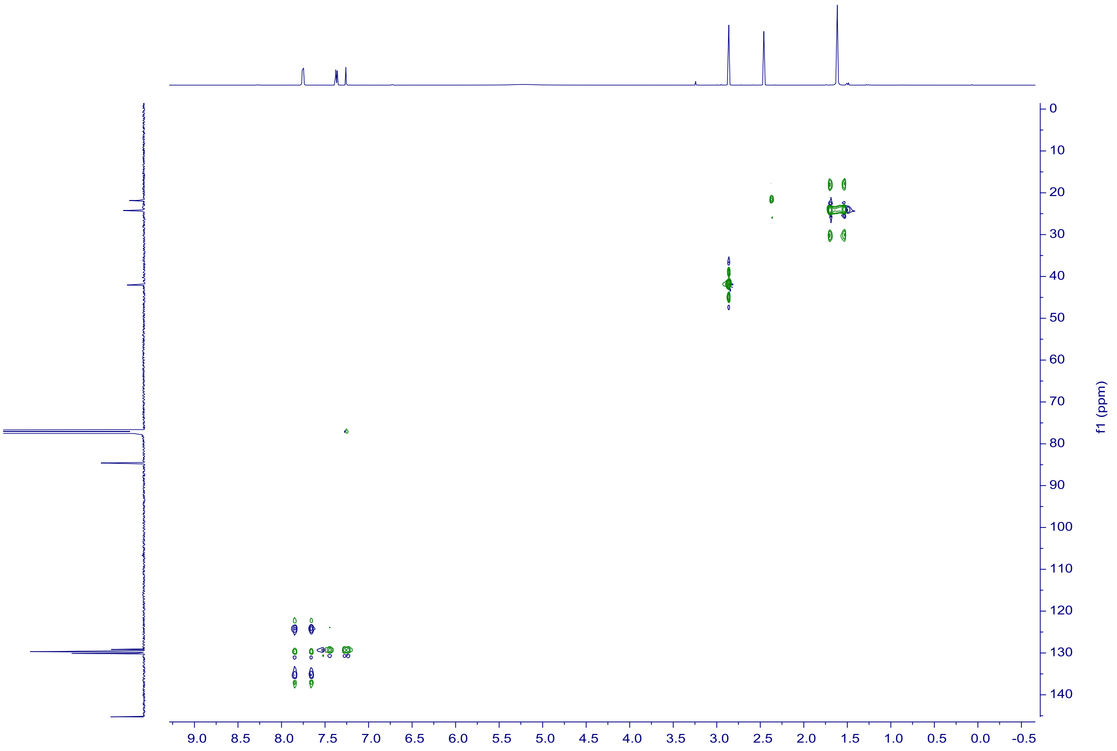

7e – HMBC (500 MHz, $CDCl_3$)

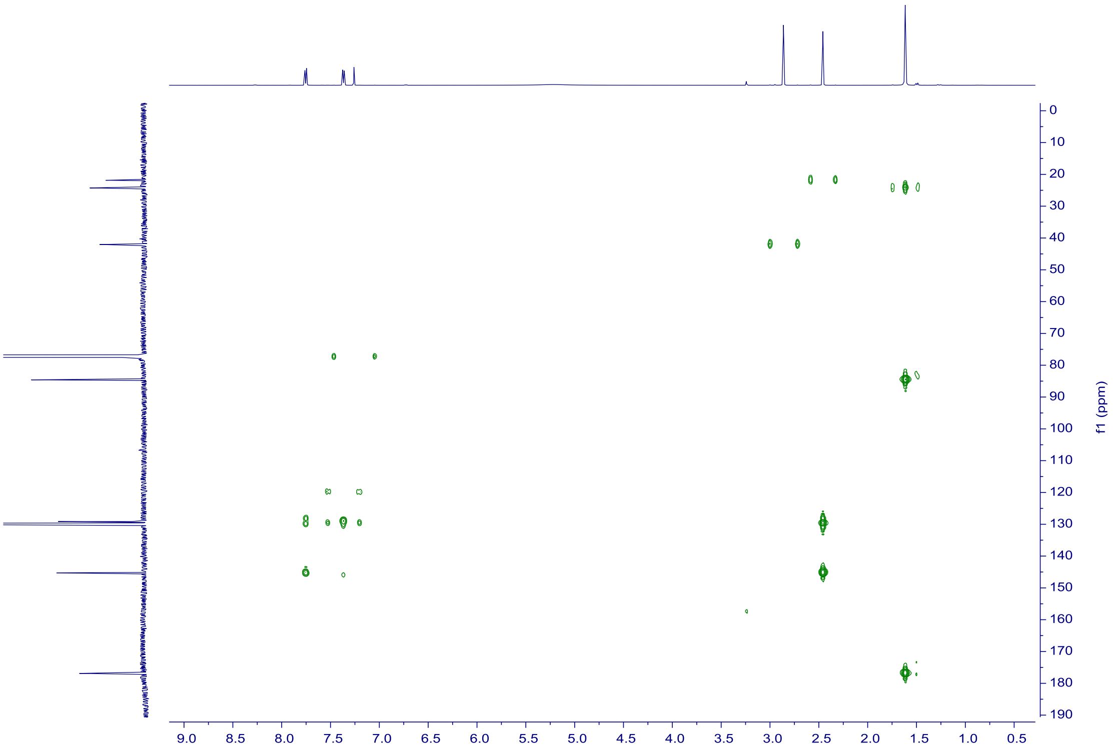

7f – ^{1}H NMR (400 MHz, $CDCl_3$)

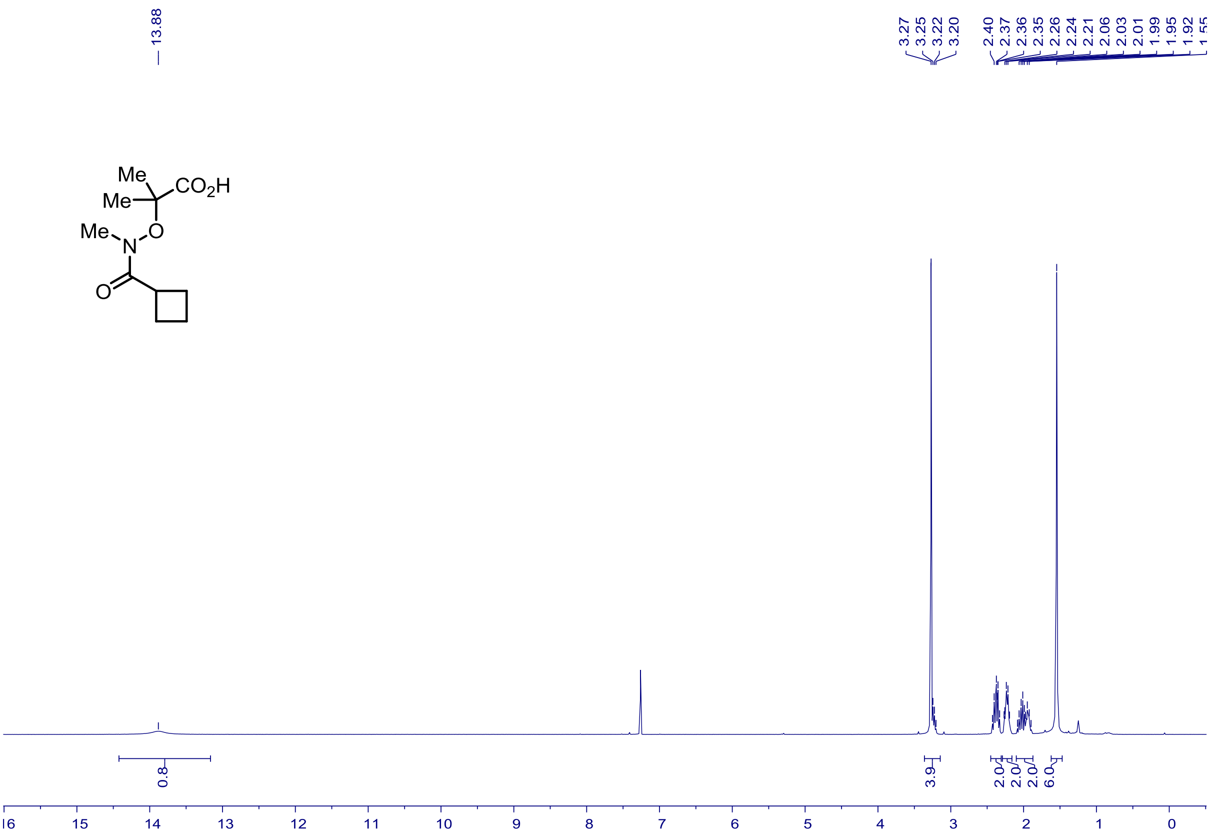

7f – ^{13}C NMR (101 MHz, $CDCl_3$)

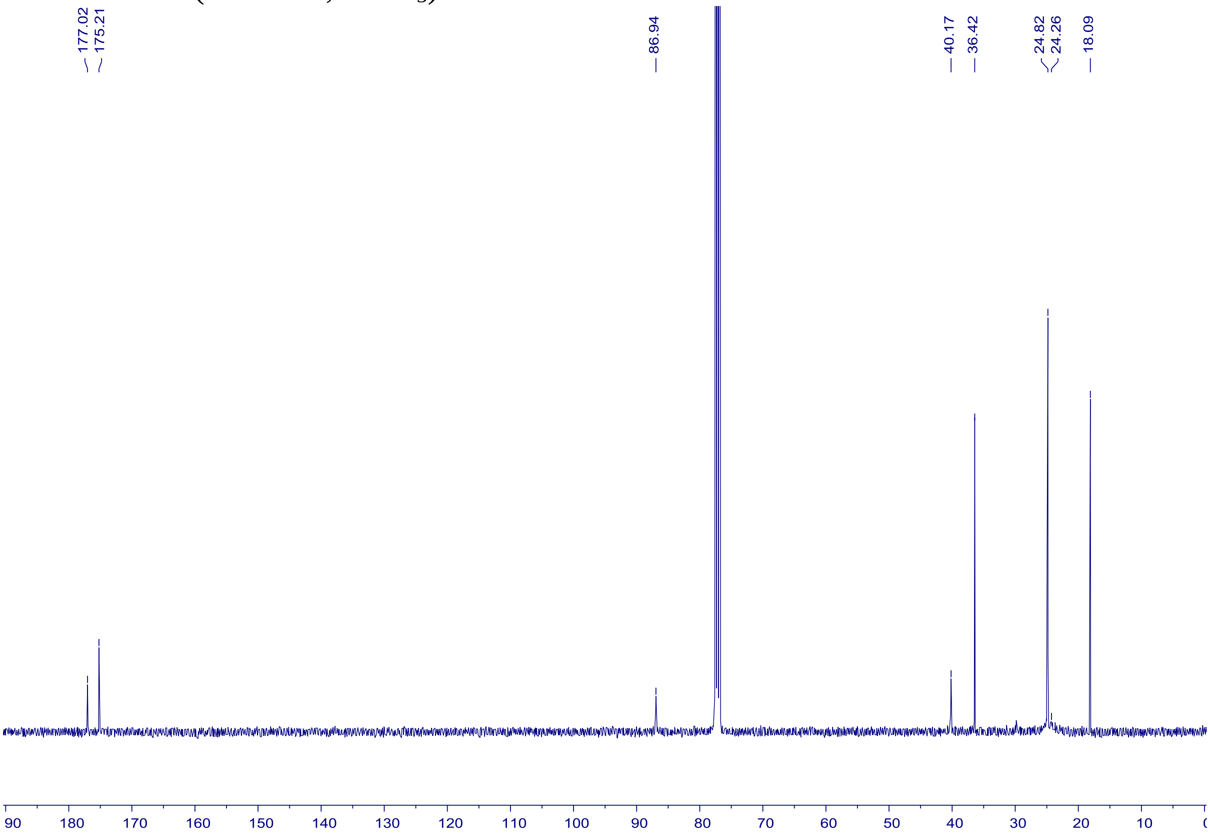

7g – ^{1}H NMR (400 MHz, $CDCl_3$)

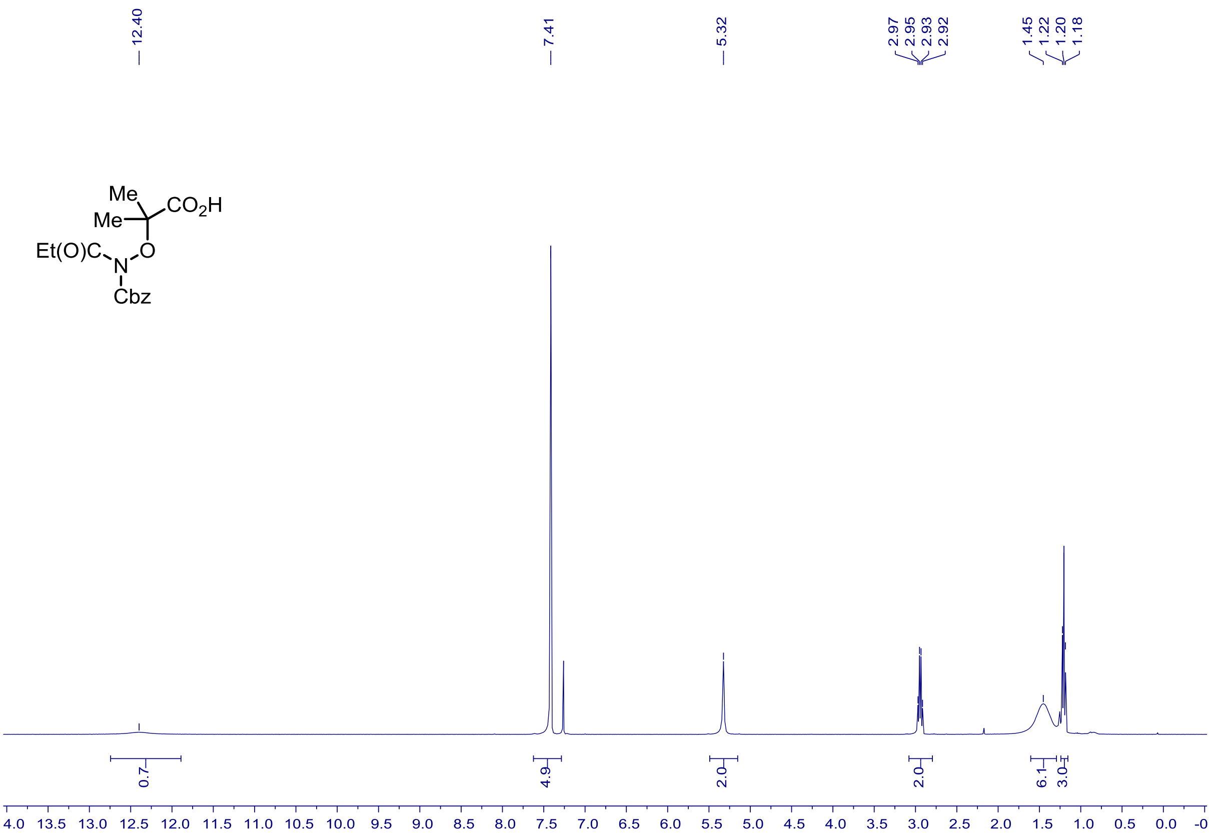

7g – ^{13}C NMR (101 MHz, $CDCl_3$)

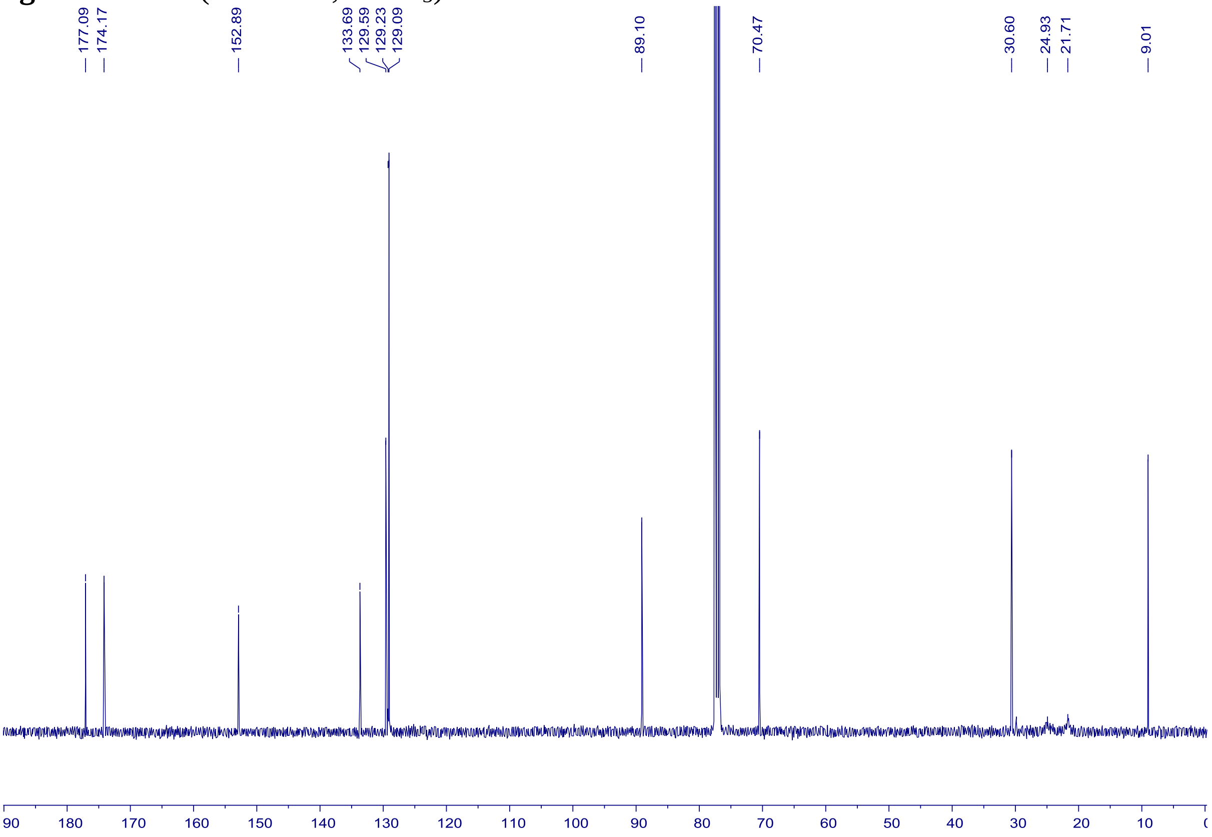

7h – ^{1}H NMR (400 MHz, $CDCl_3$)

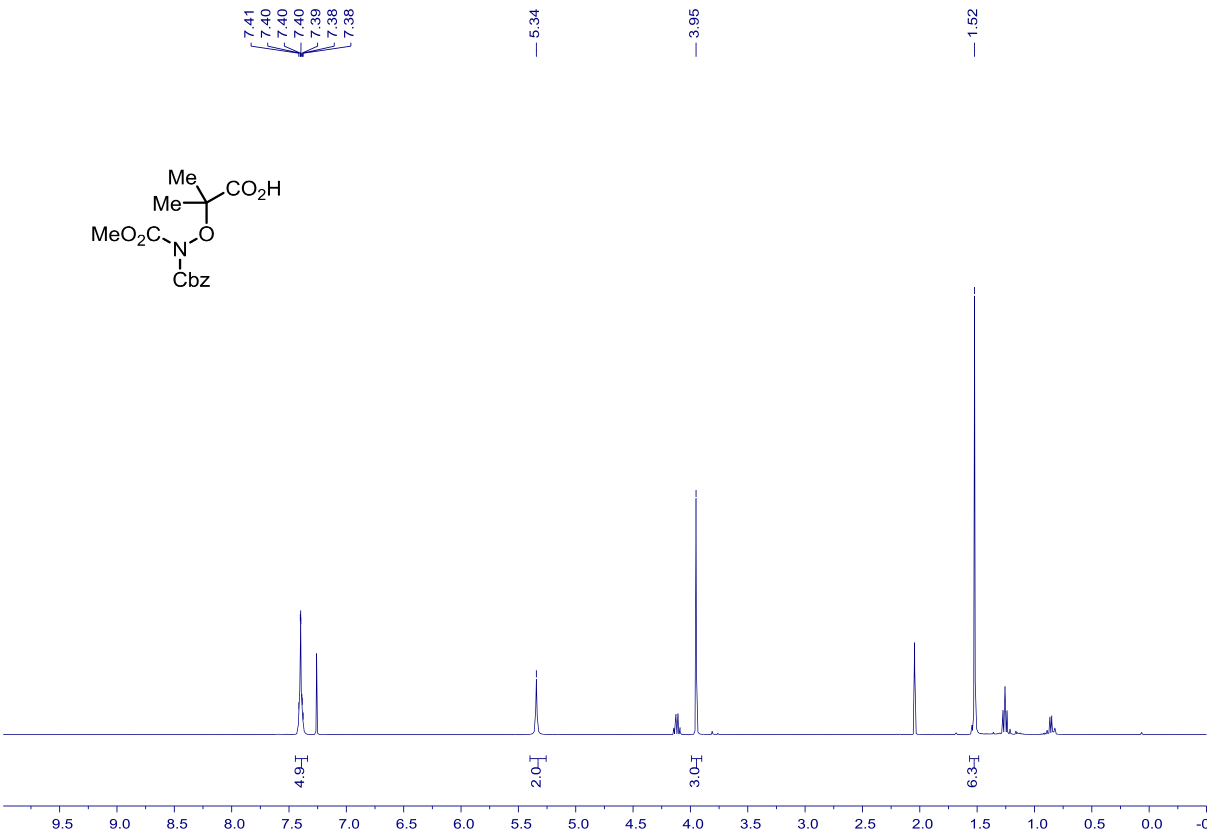

7h – ^{13}C NMR (101 MHz, $CDCl_3$)

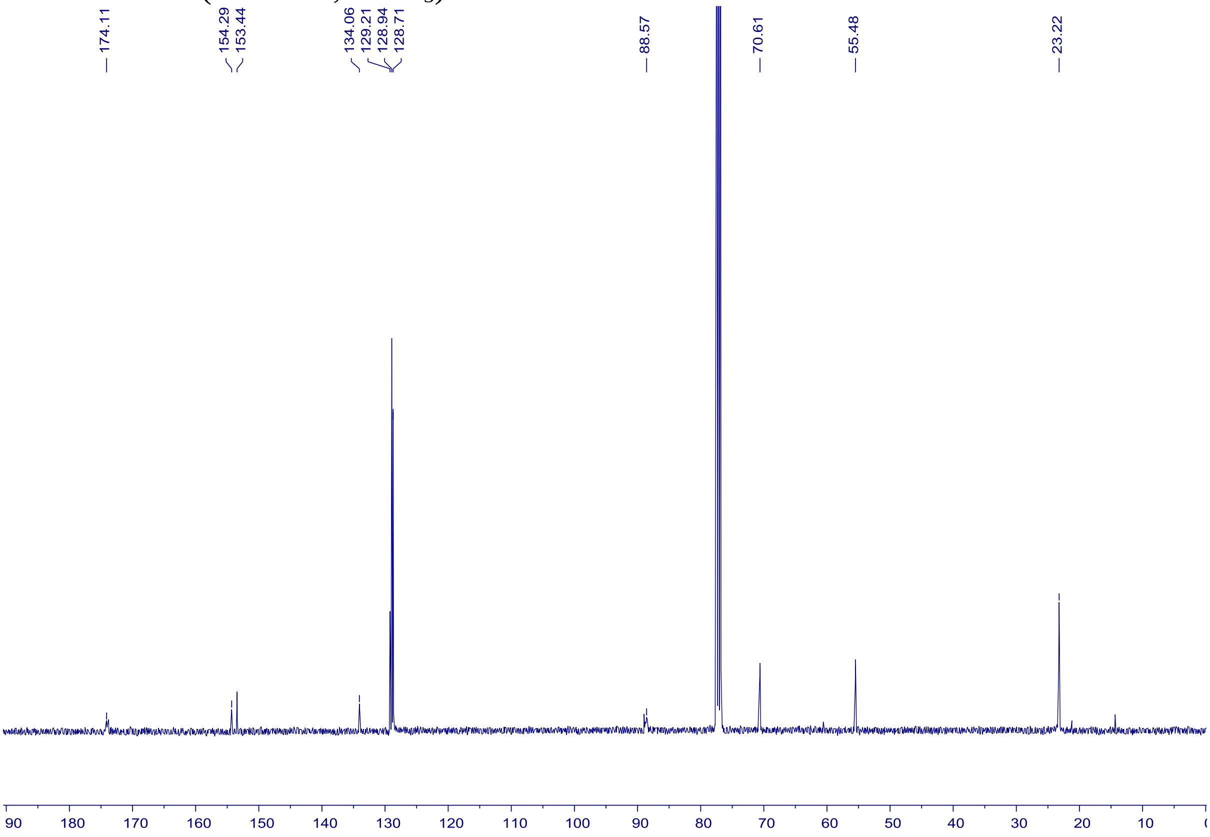

7i – ^{1}H NMR (400 MHz, $CDCl_3$)

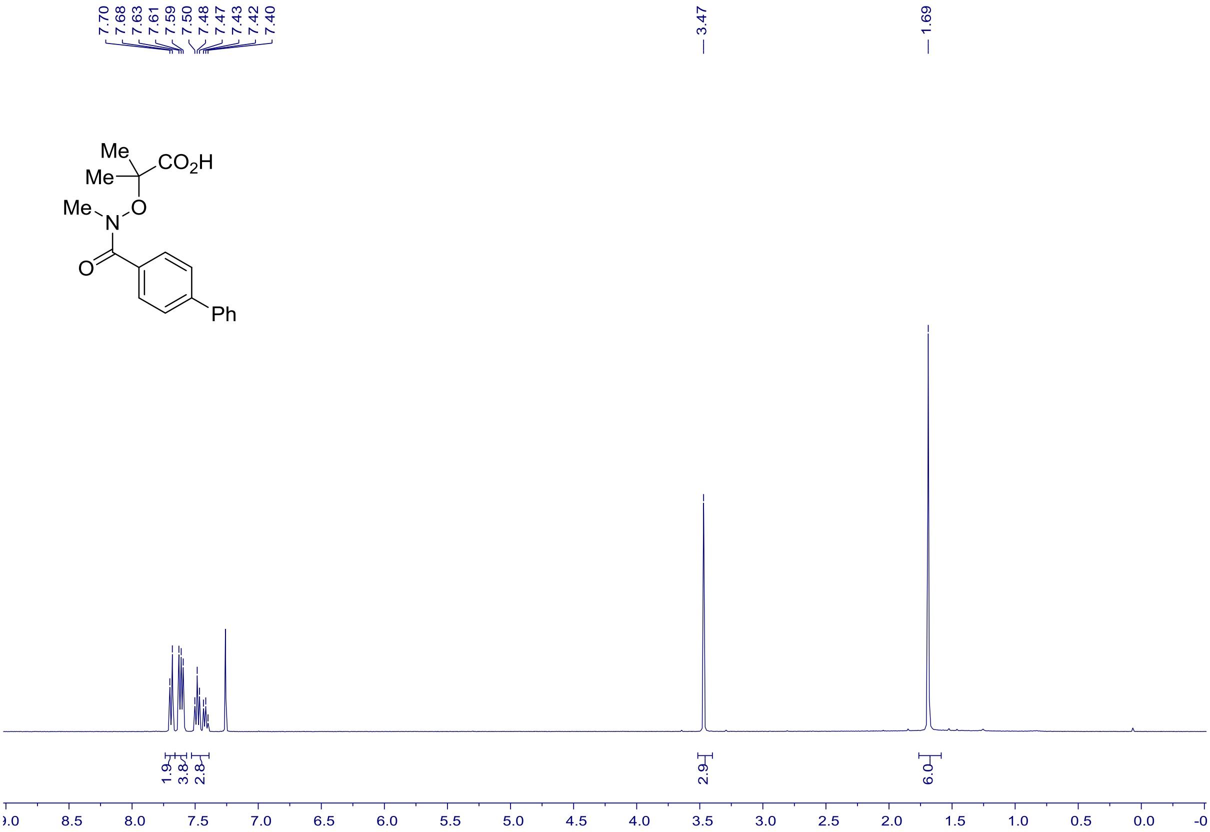

7i – ^{13}C NMR (101 MHz, $CDCl_3$)

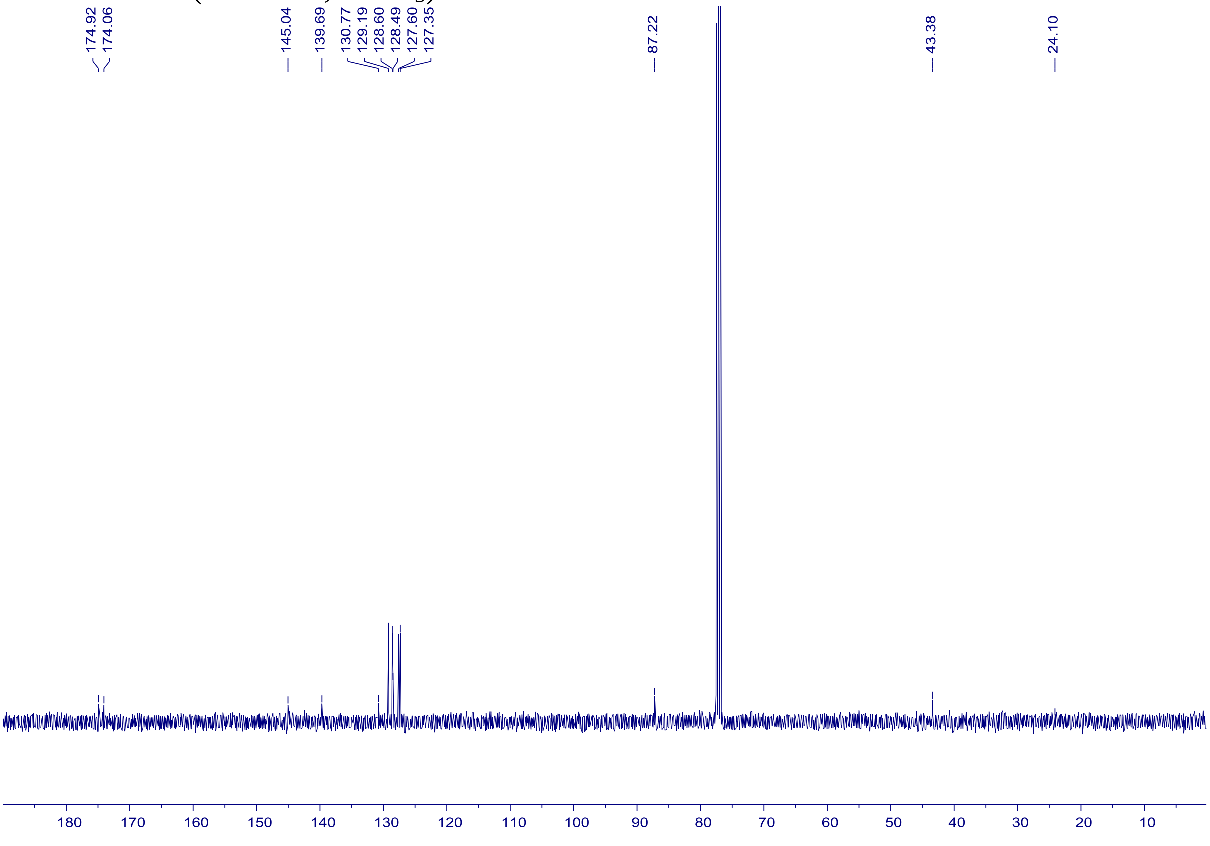

7j – ^{1}H NMR (500 MHz, $CDCl_3$)

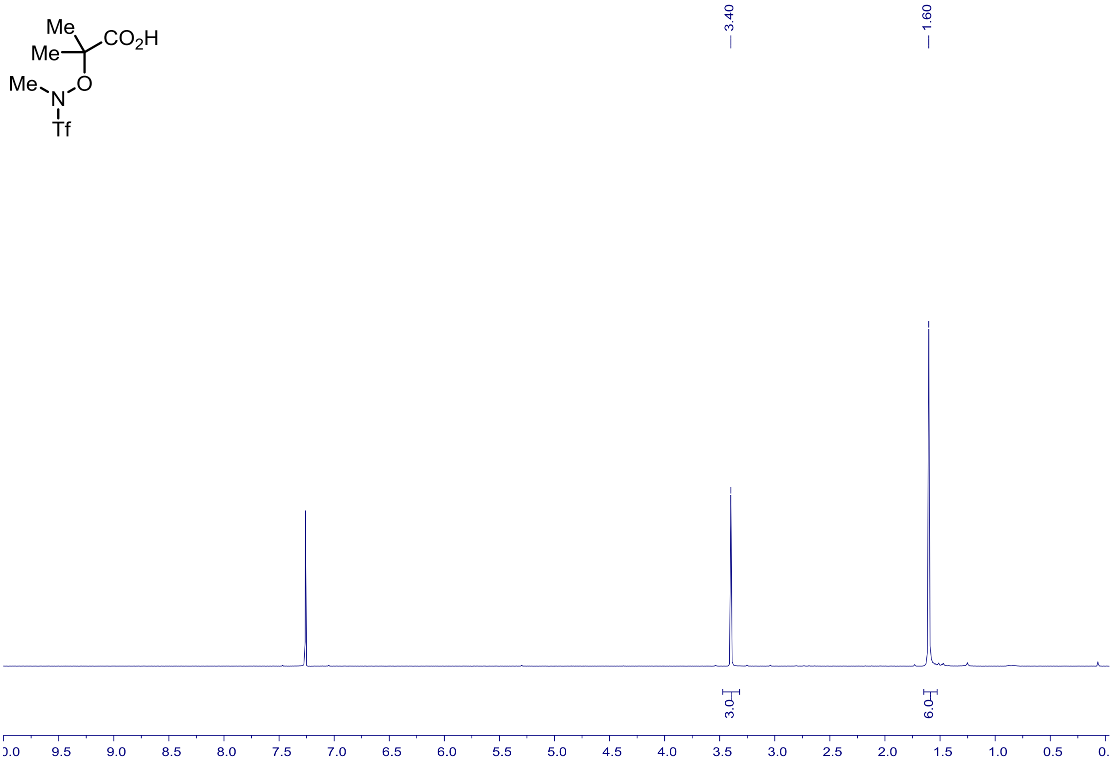

7j – ^{13}C NMR (126MHz, $CDCl_3$)

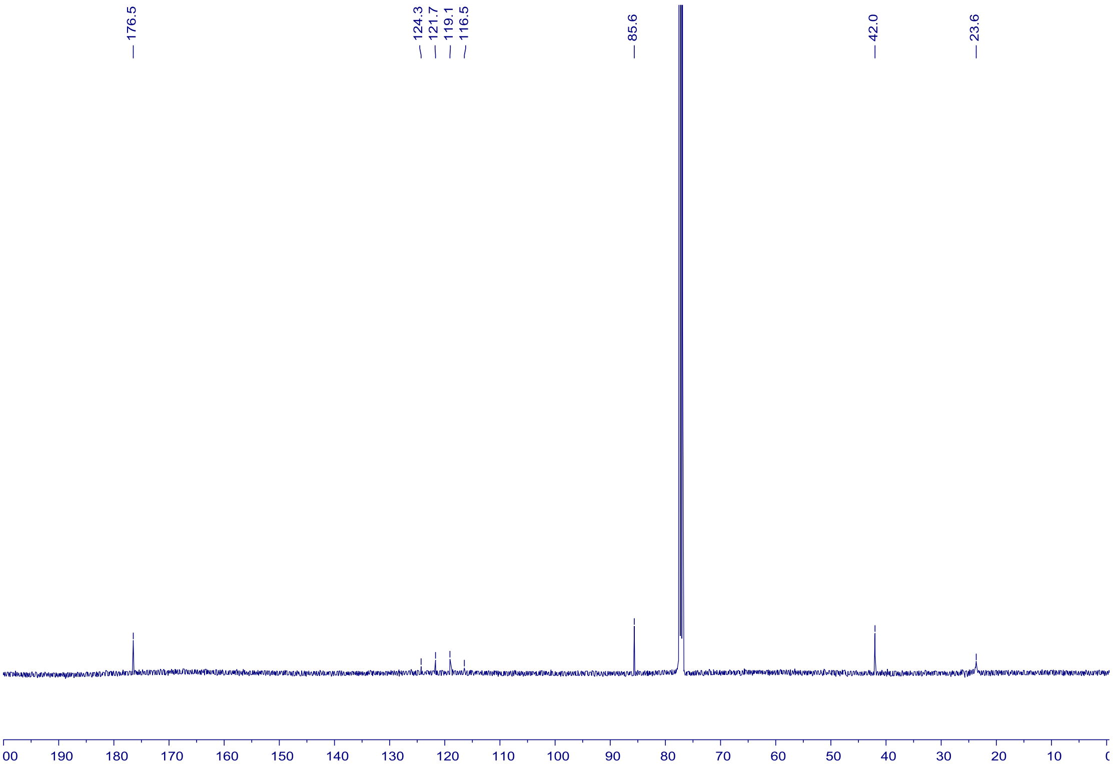

7j – ^{19}F NMR (376MHz, $CDCl_3$)

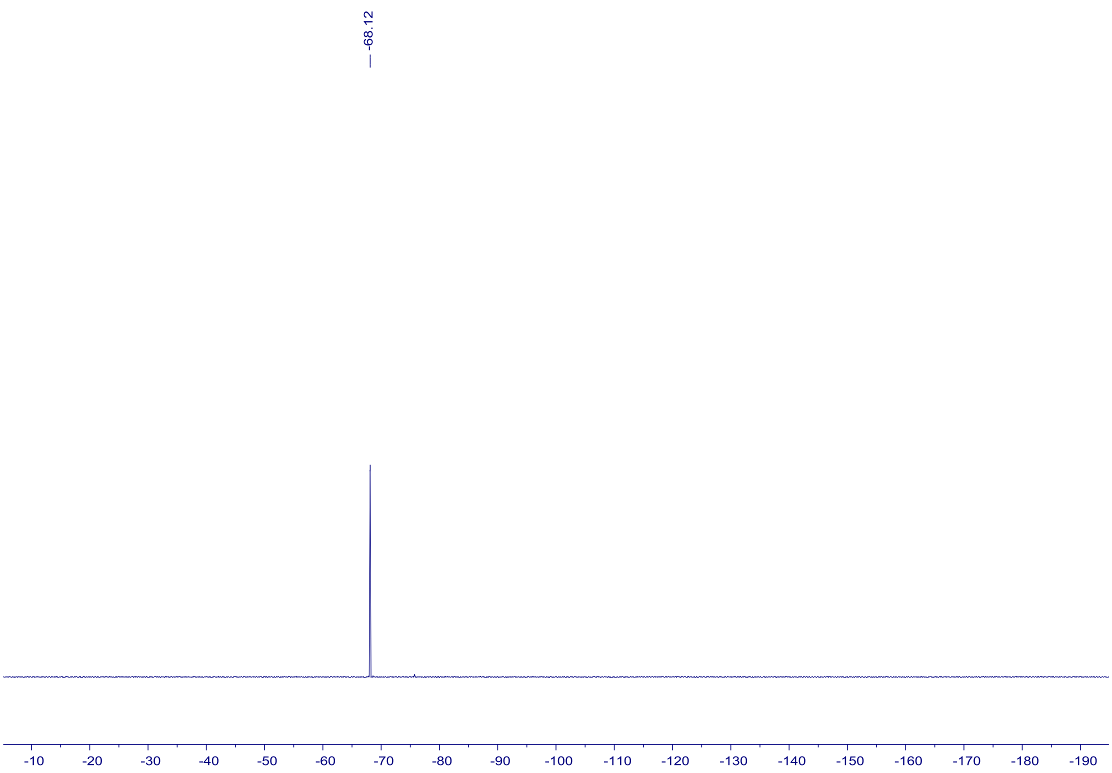

7j – HSQC (500 MHz, $CDCl_3$)

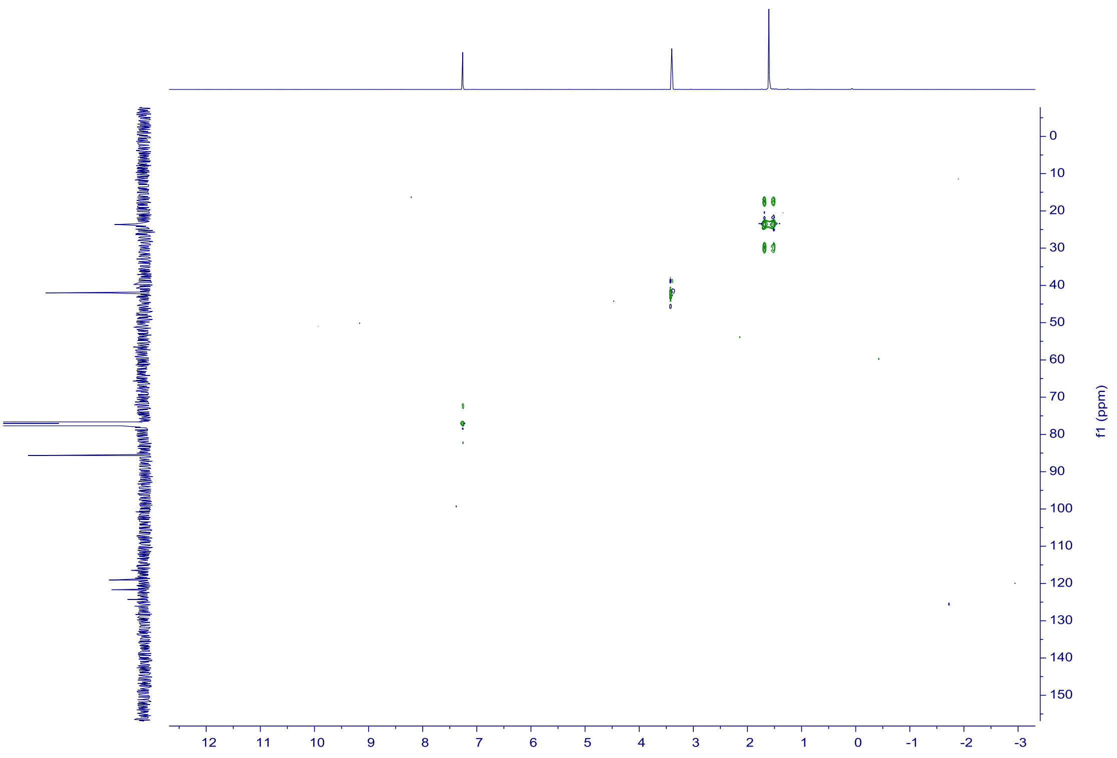

7j – HMBC (500 MHz, $CDCl_3$)

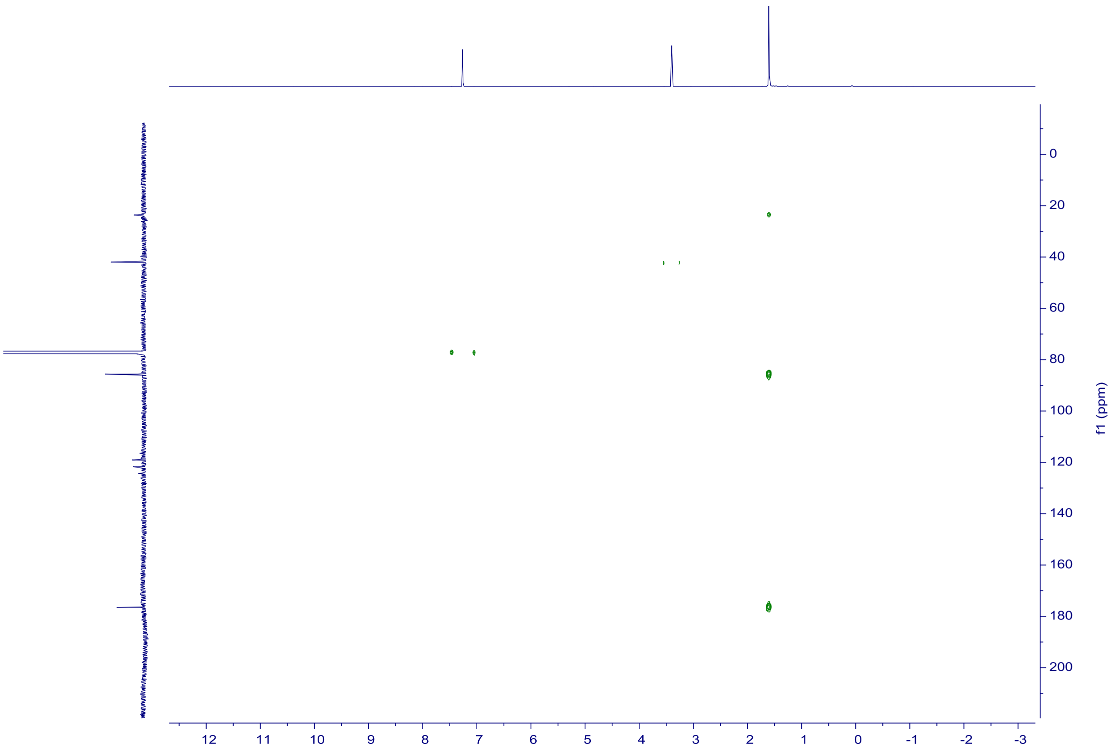

7k – ^{1}H NMR (400 MHz, $CDCl_3$)

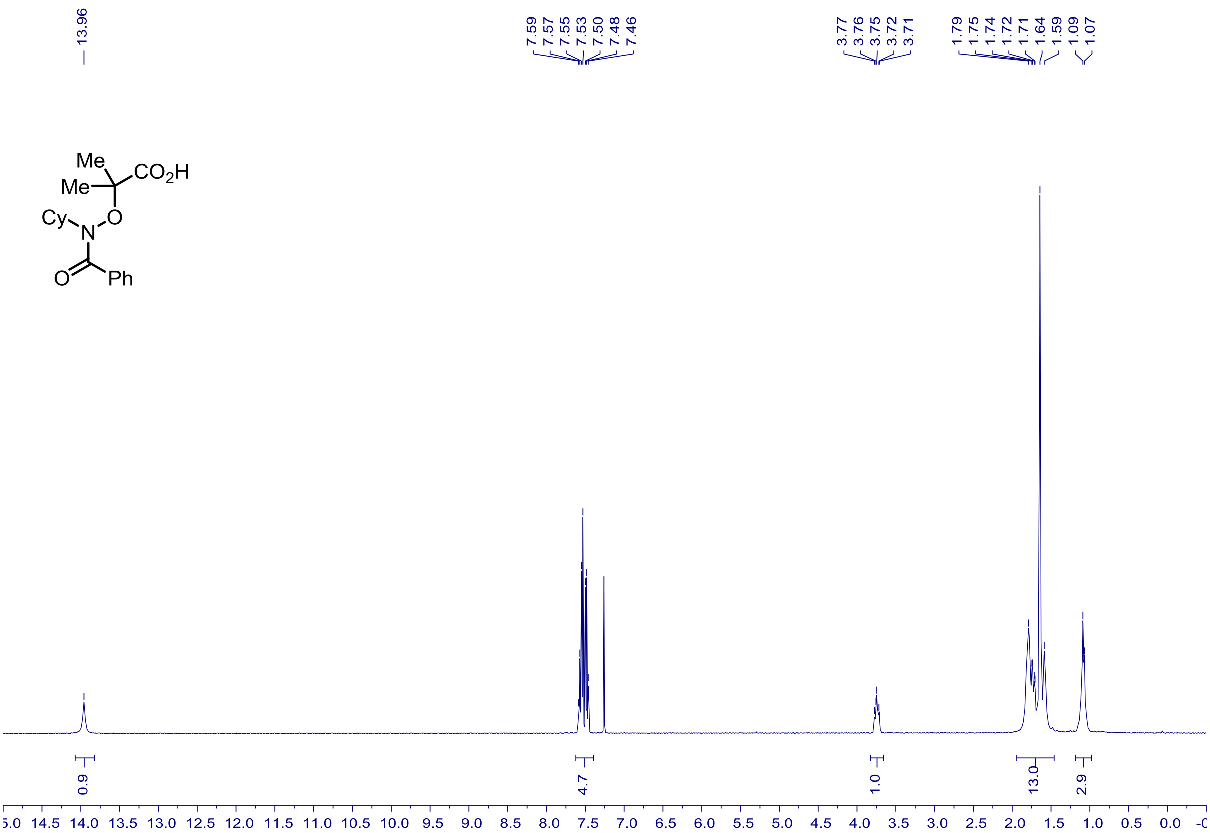

7k – ^{13}C NMR (101 MHz, $CDCl_3$)

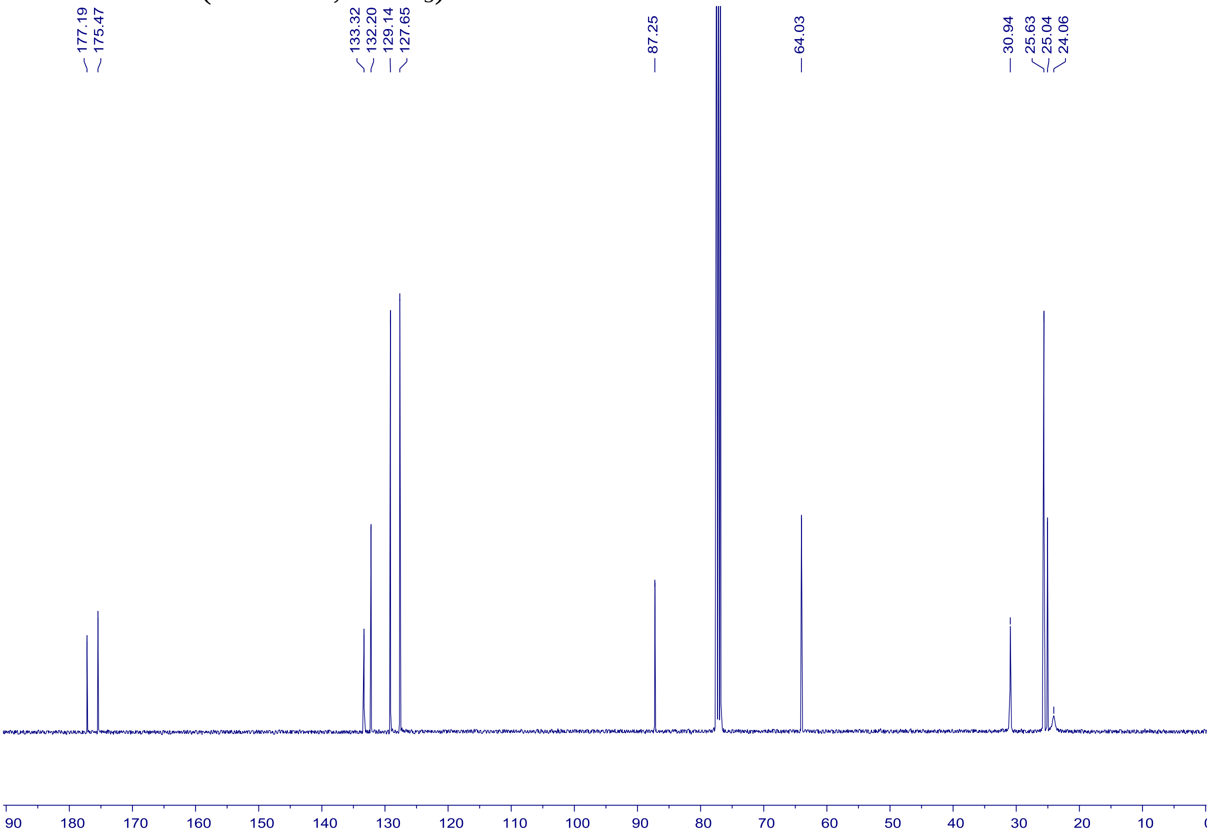

7l – ^{1}H NMR (400 MHz, $CDCl_3$)

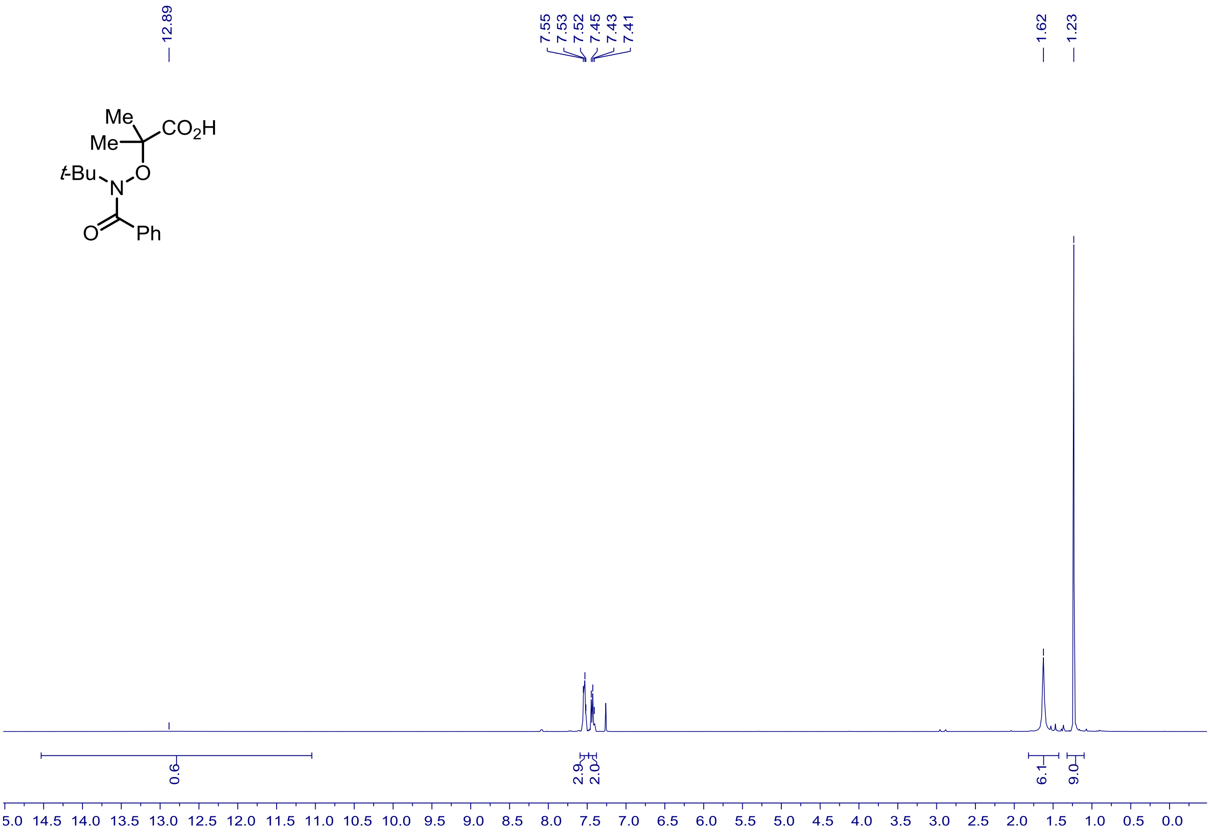

7l – ^{13}C NMR (126 MHz, $CDCl_3$)

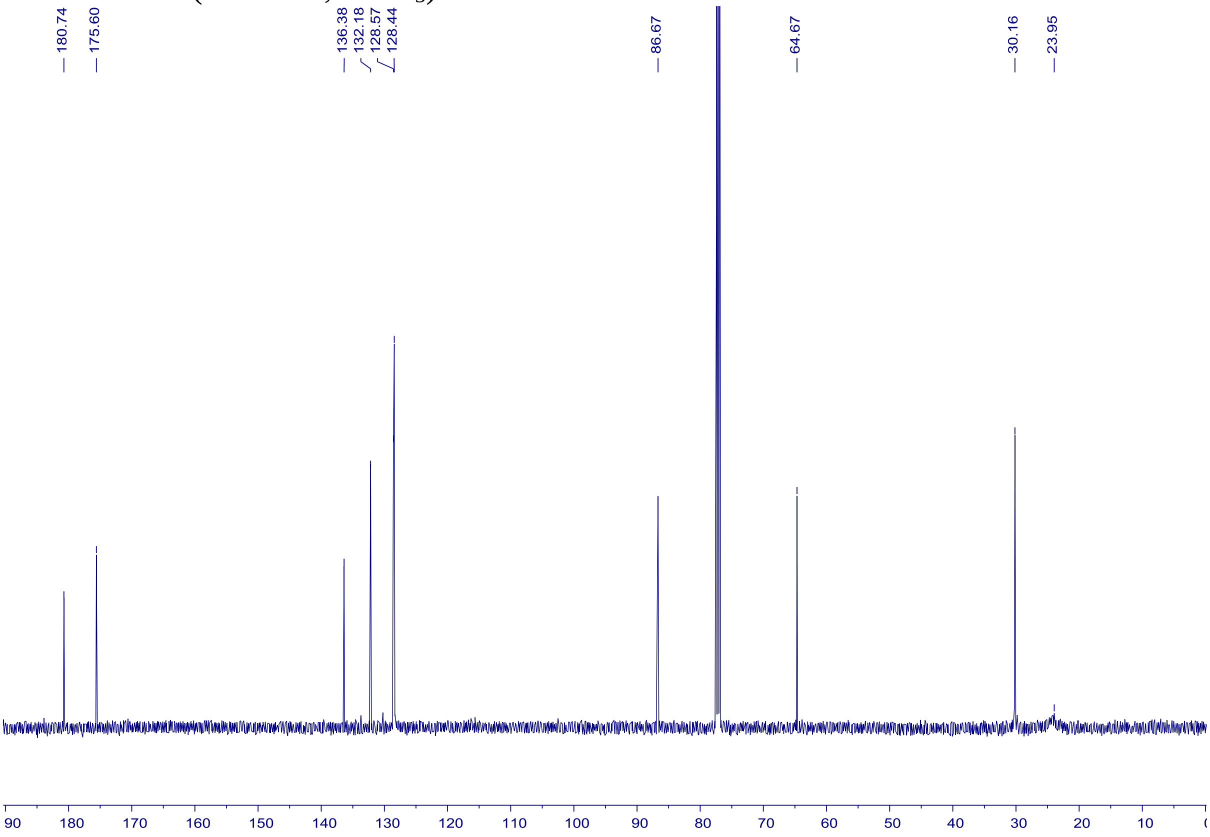

7m – ^{1}H NMR (500 MHz, $CDCl_3$)

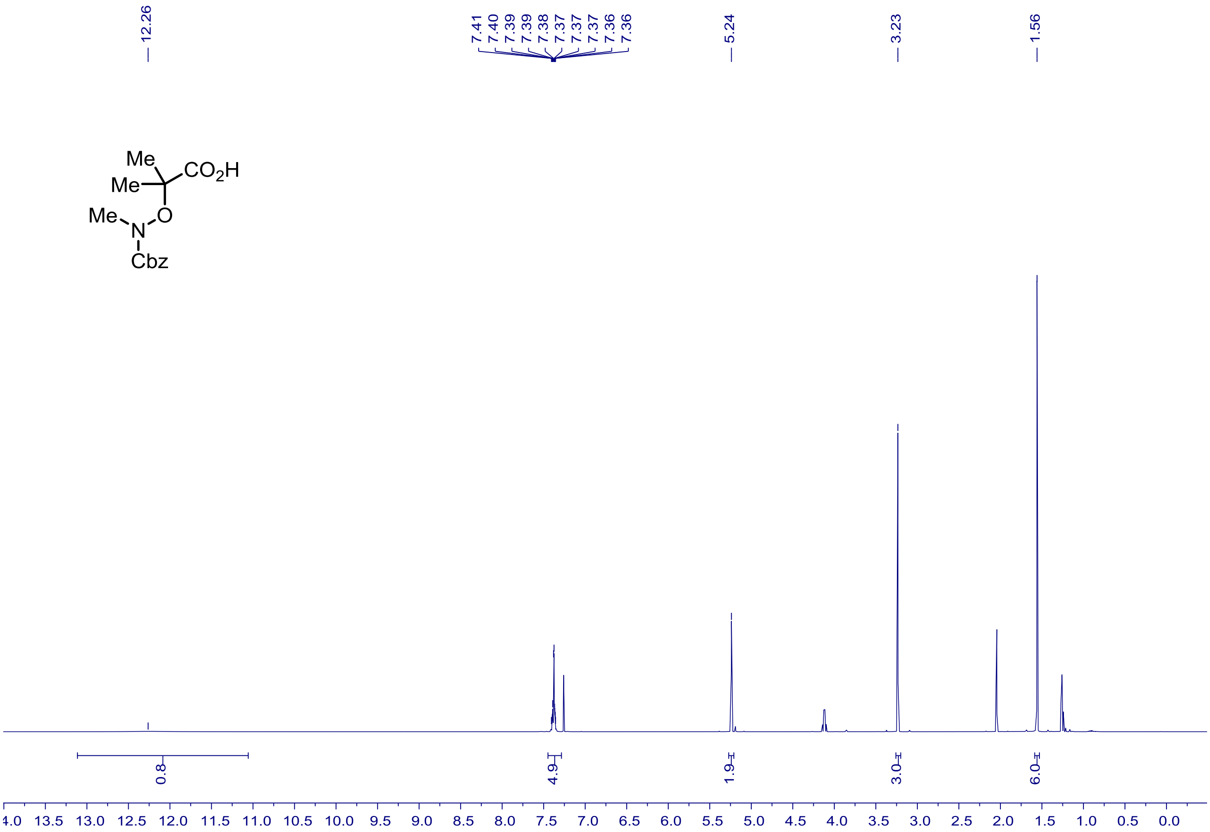

7m – ^{13}C NMR (126 MHz, $CDCl_3$)

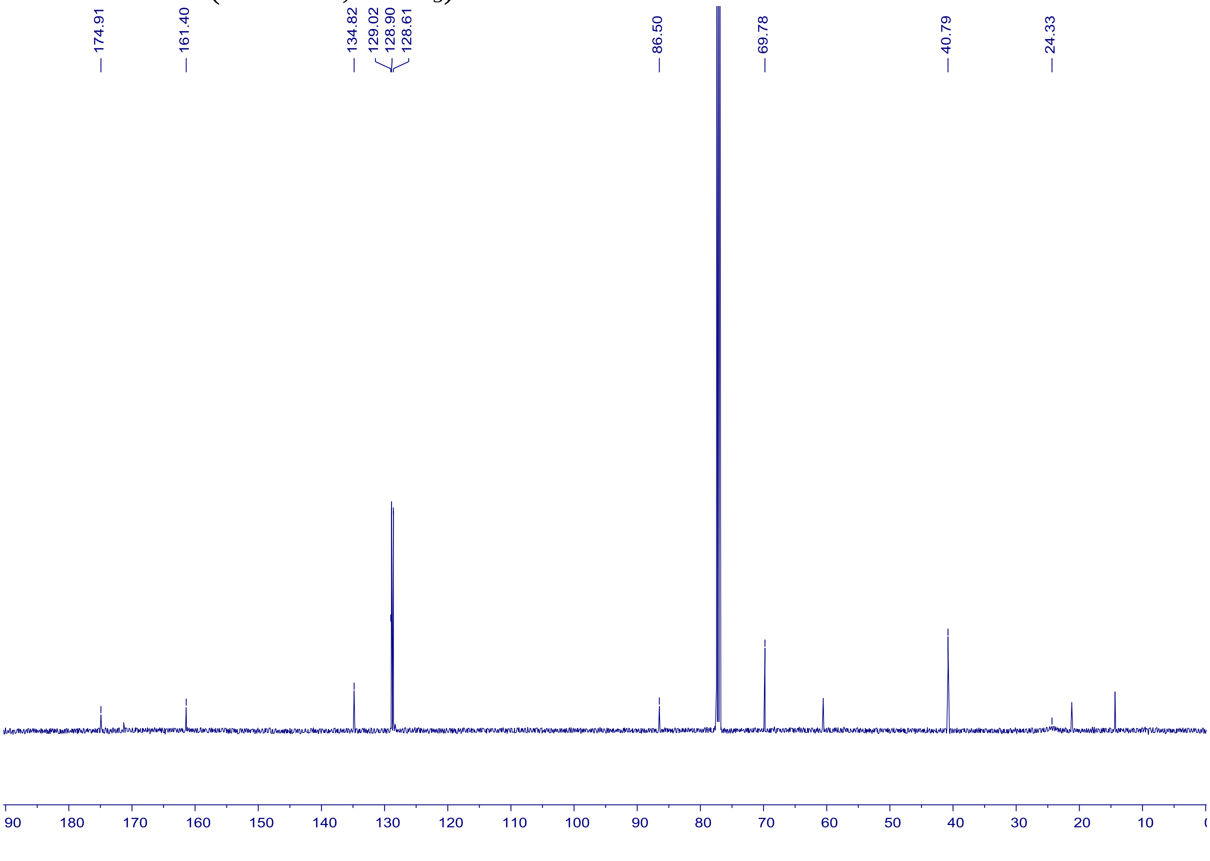

7n – ^{1}H NMR (400 MHz, $CDCl_3$)

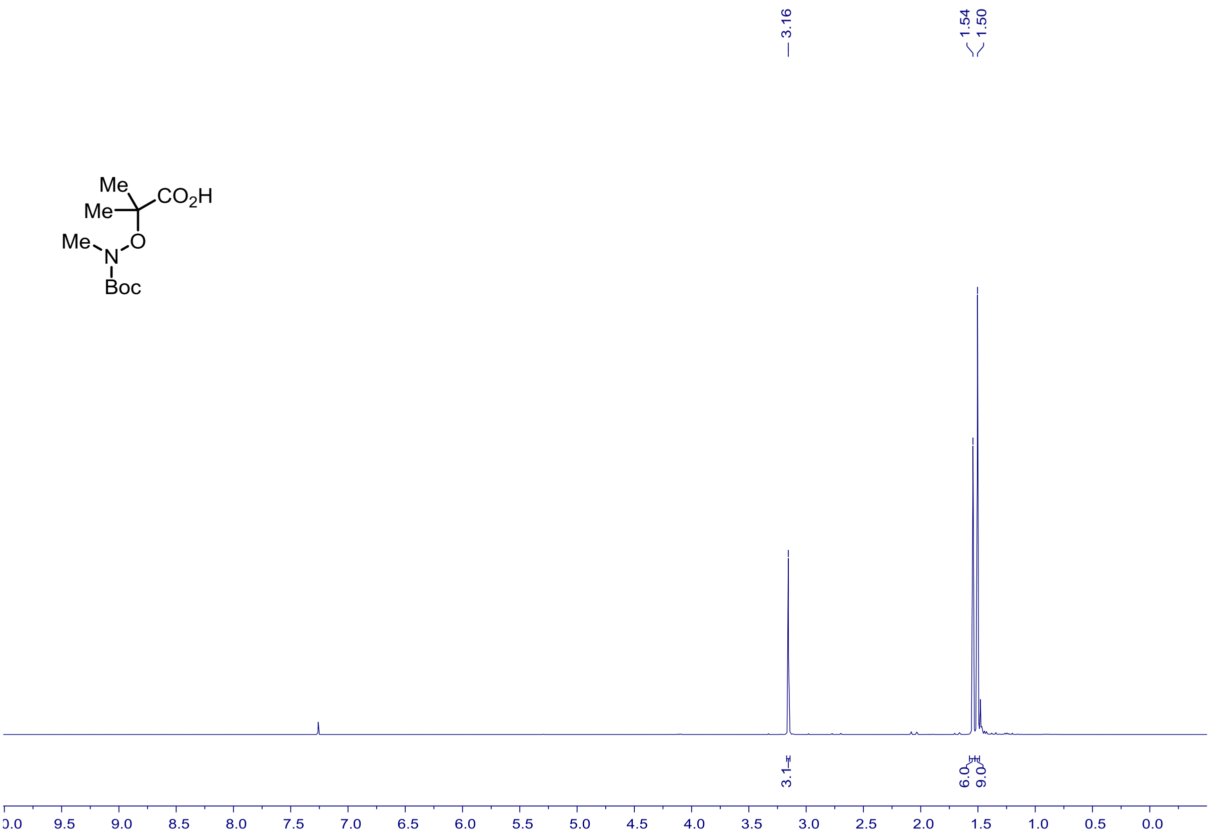

7n – ^{13}C NMR (101 MHz, $CDCl_3$)

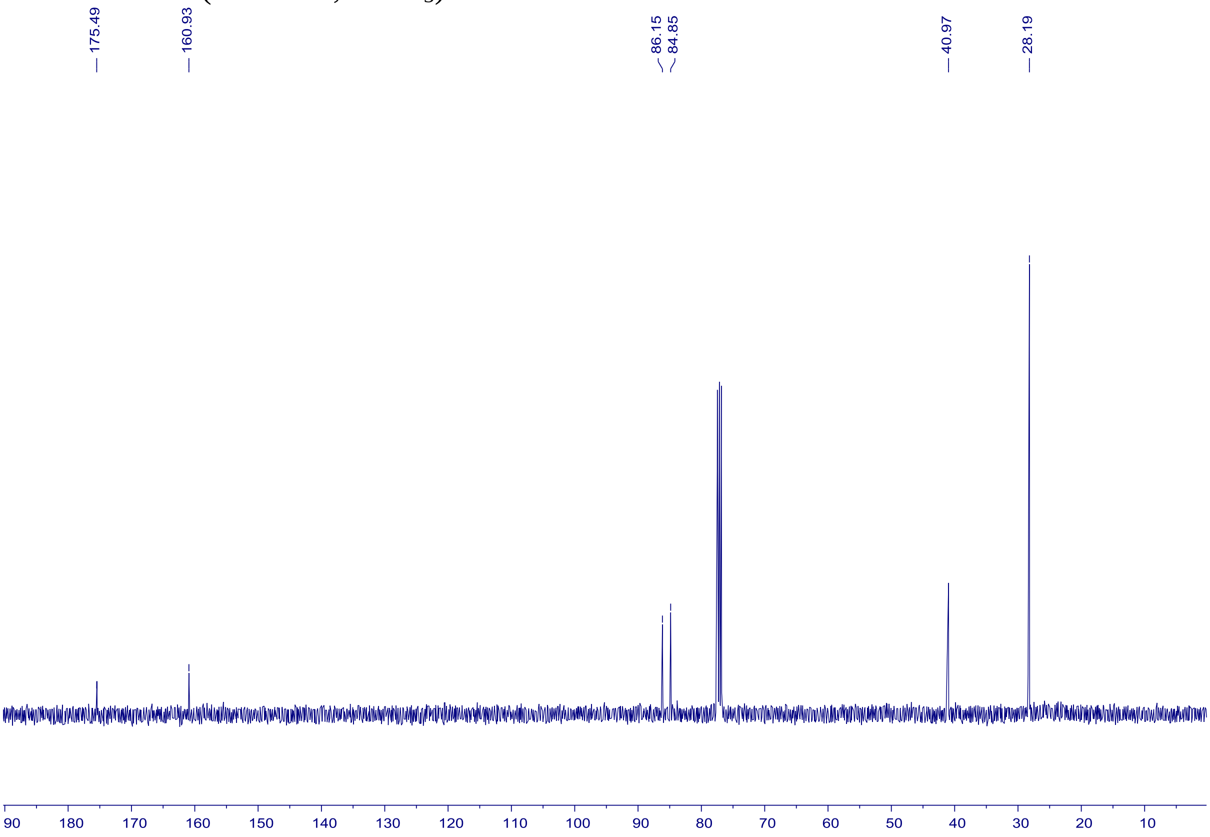

7o – ^{1}H NMR (400 MHz, $CDCl_3$)

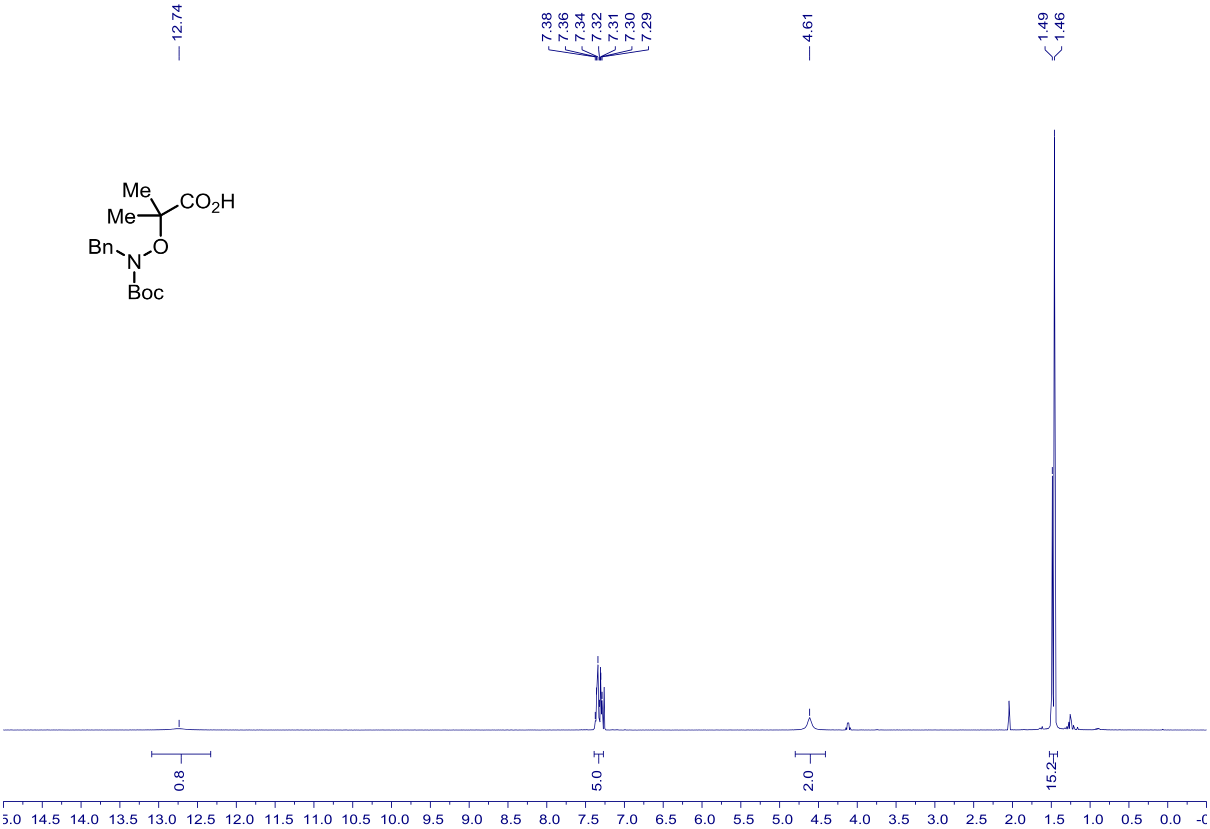

7o – ^{13}C NMR (101 MHz, $CDCl_3$)

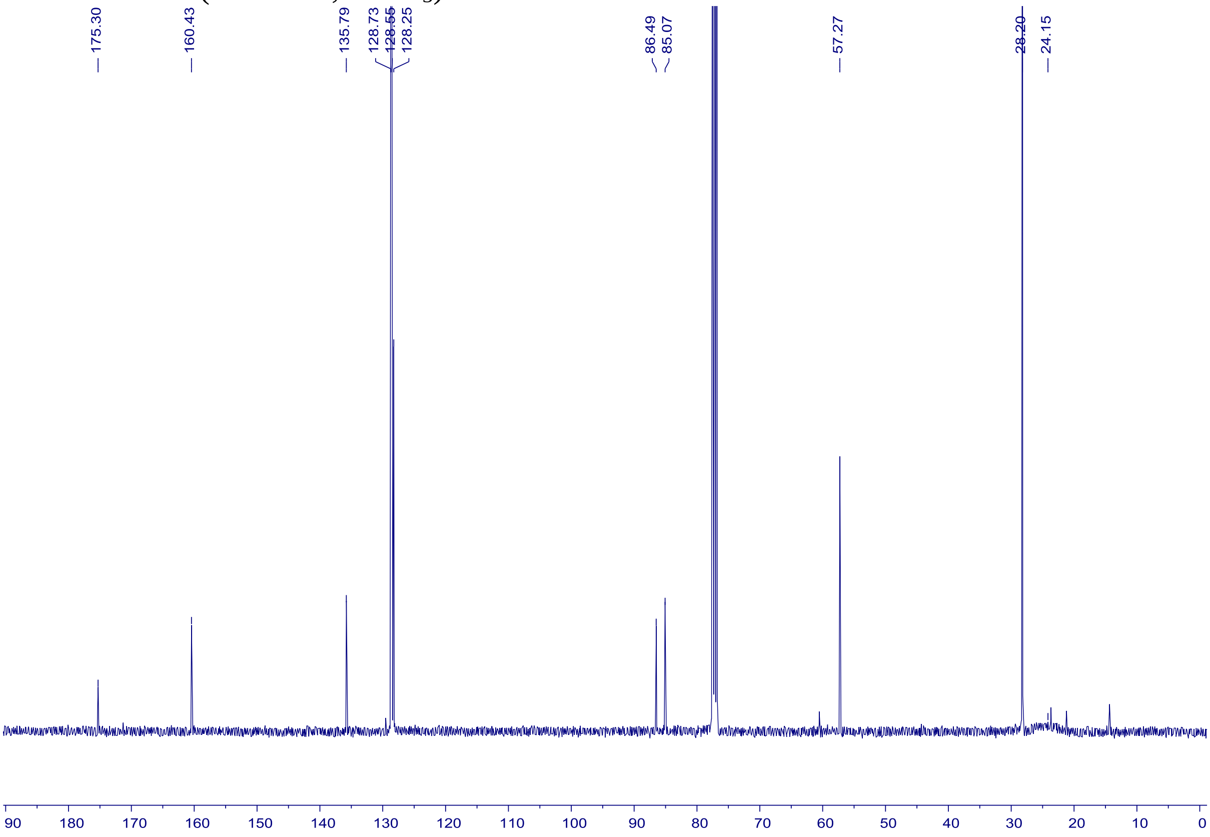

7p – ^{1}H NMR (400 MHz, $CDCl_3$)

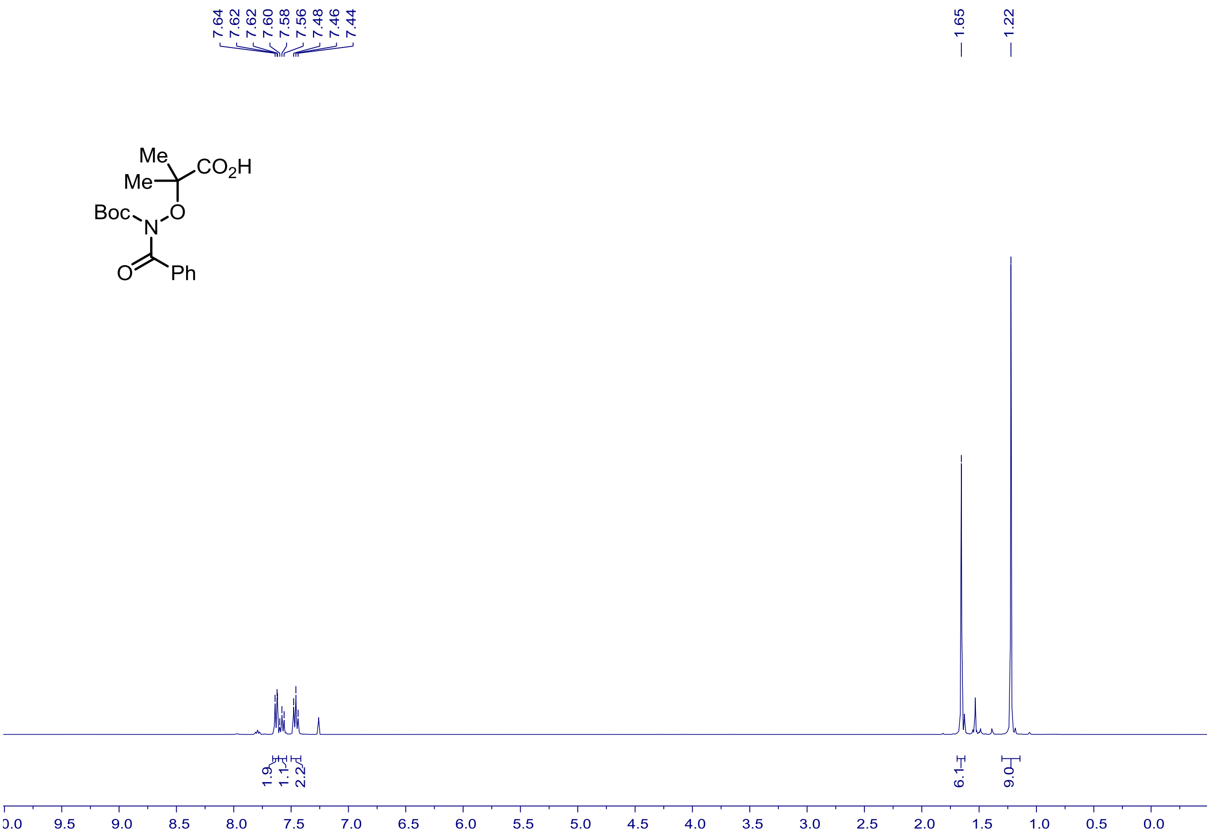

7p – ^{13}C NMR (101 MHz, $CDCl_3$)

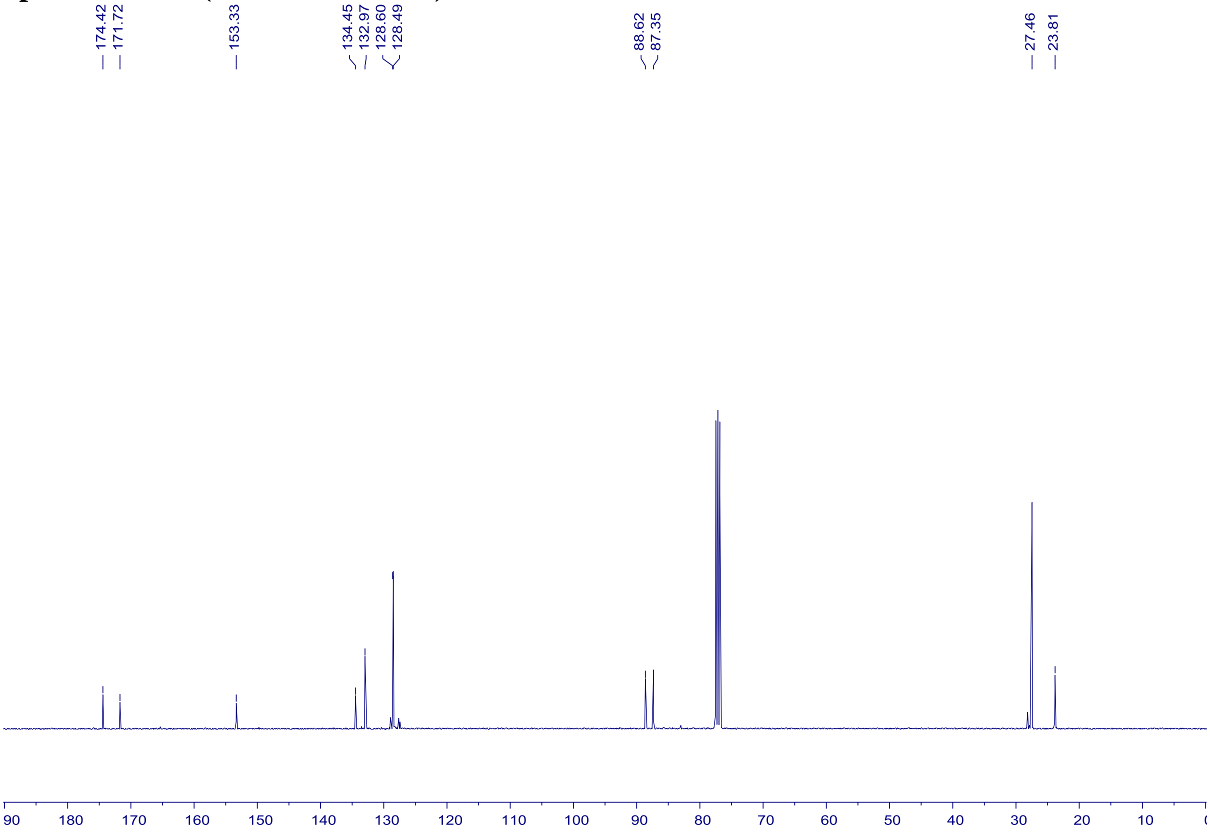

7q – ^{1}H NMR (500 MHz, $CDCl_3$)

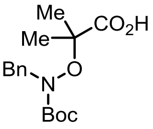

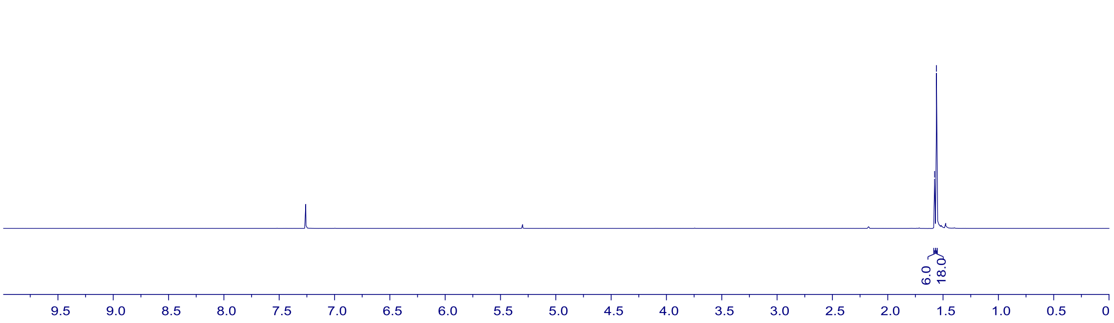

7q – ^{13}C NMR (126MHz, $CDCl_3$)

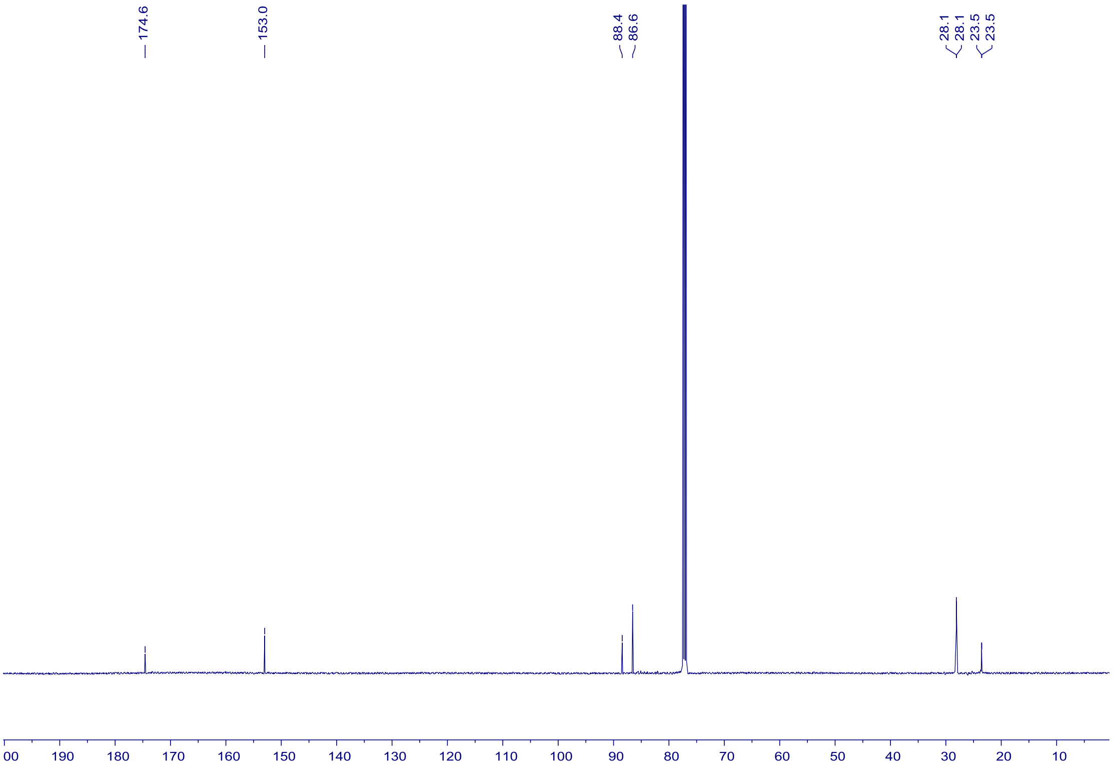

7q – HMBC (500 MHz, $CDCl_3$)

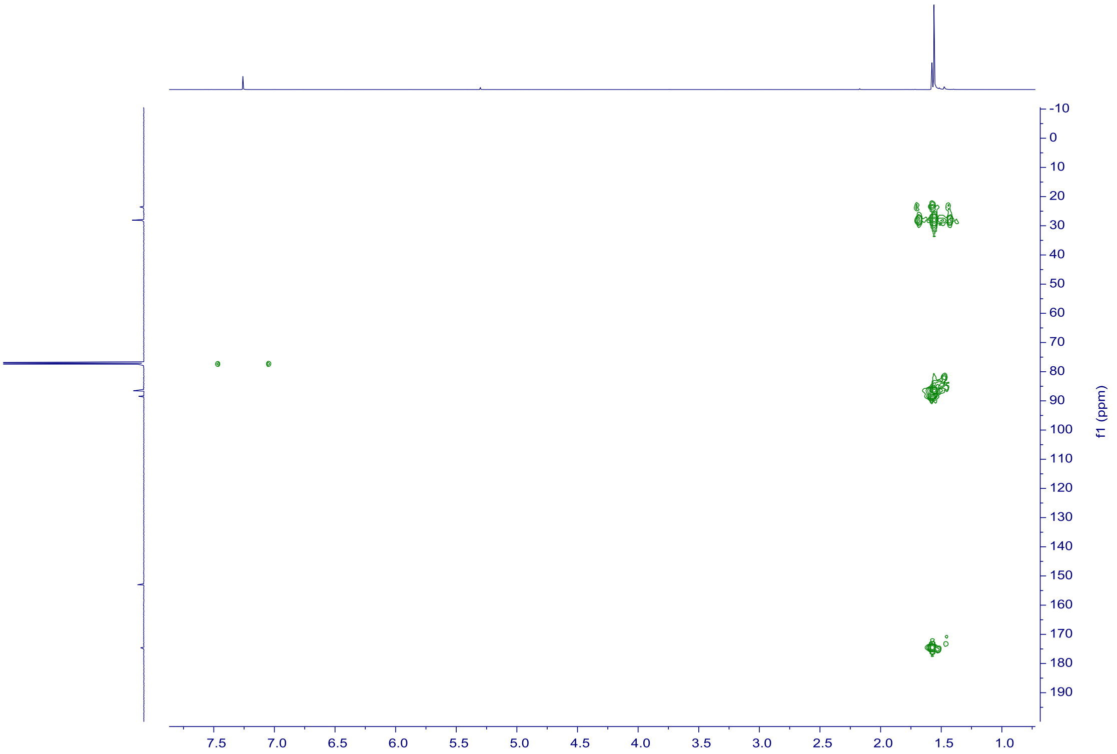

7-Cs – ^{1}H NMR (500 MHz, d_6-DMSO)

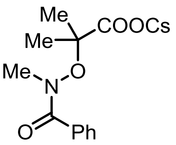

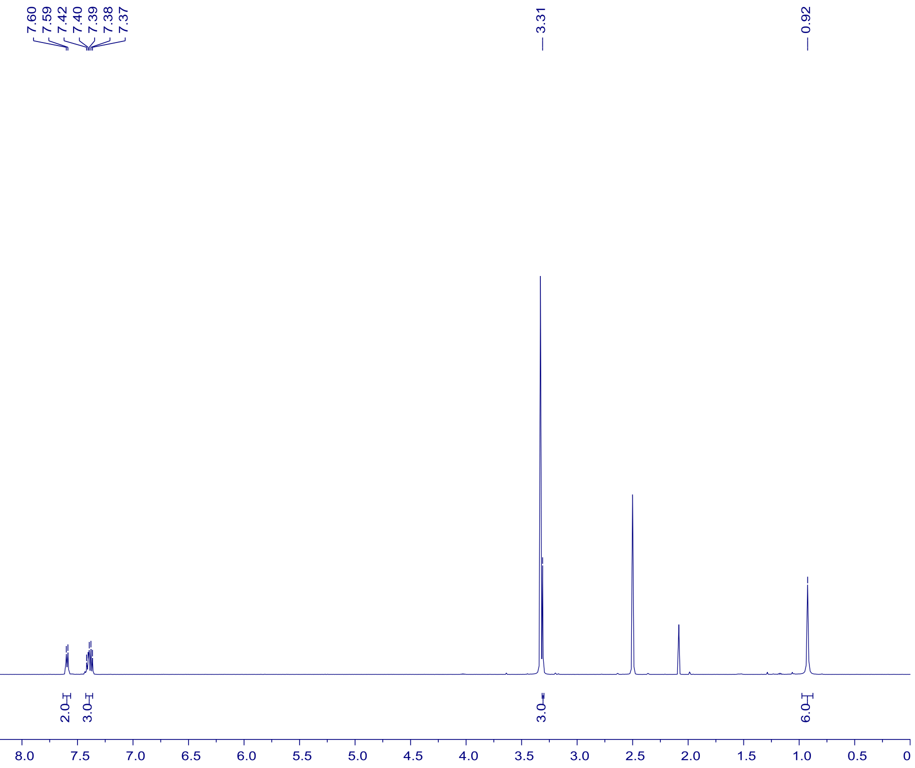

7-Cs – ^{13}C NMR (126MHz, d_6-DMSO)

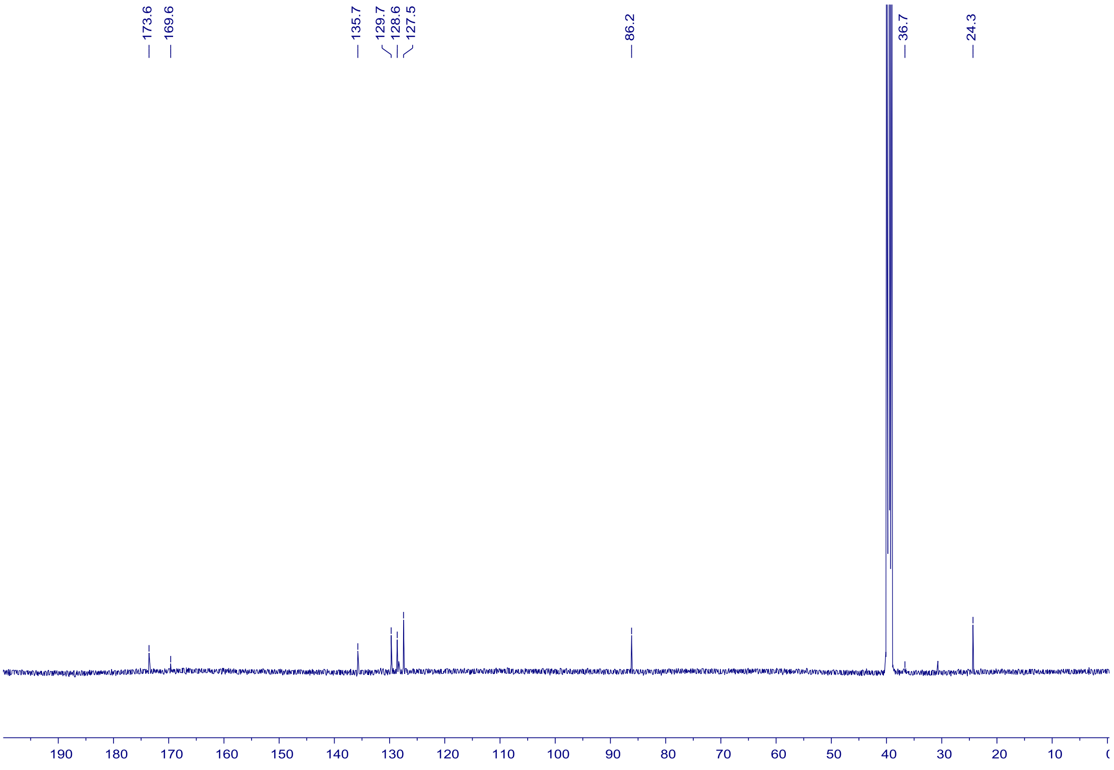

7-Cs – [13]HSQC (126MHz, d_6-DMSO)

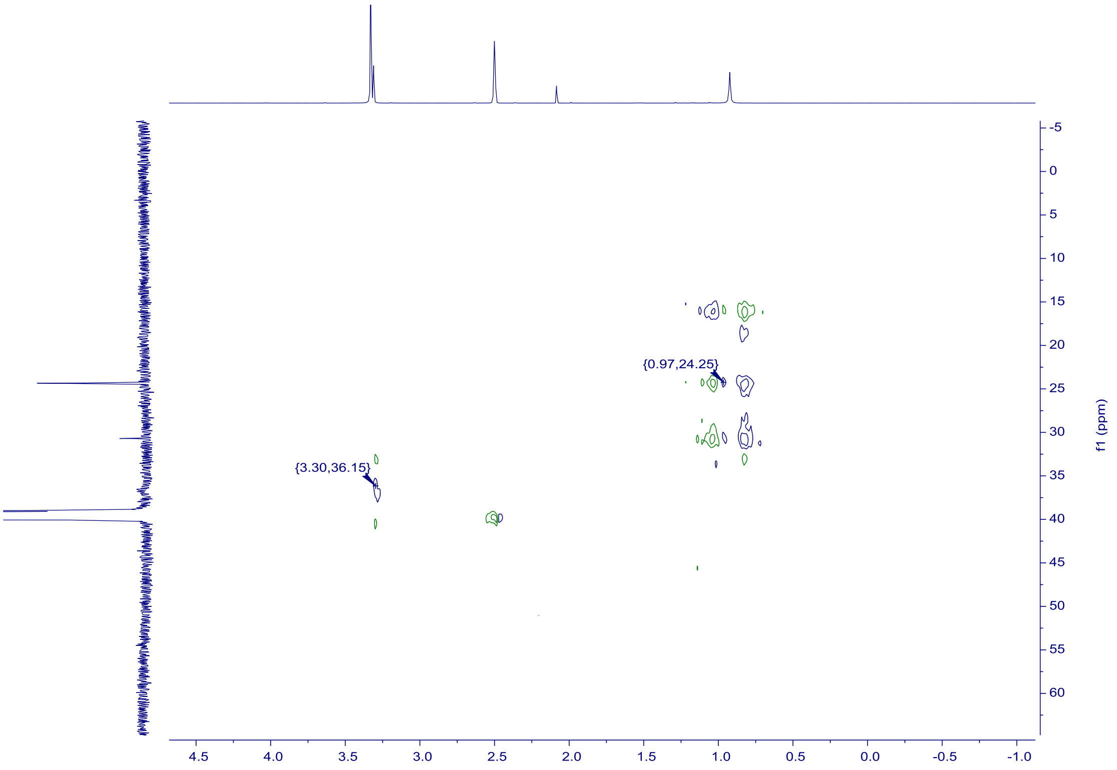

9 – ^{1}H NMR (500 MHz, $CDCl_3$)

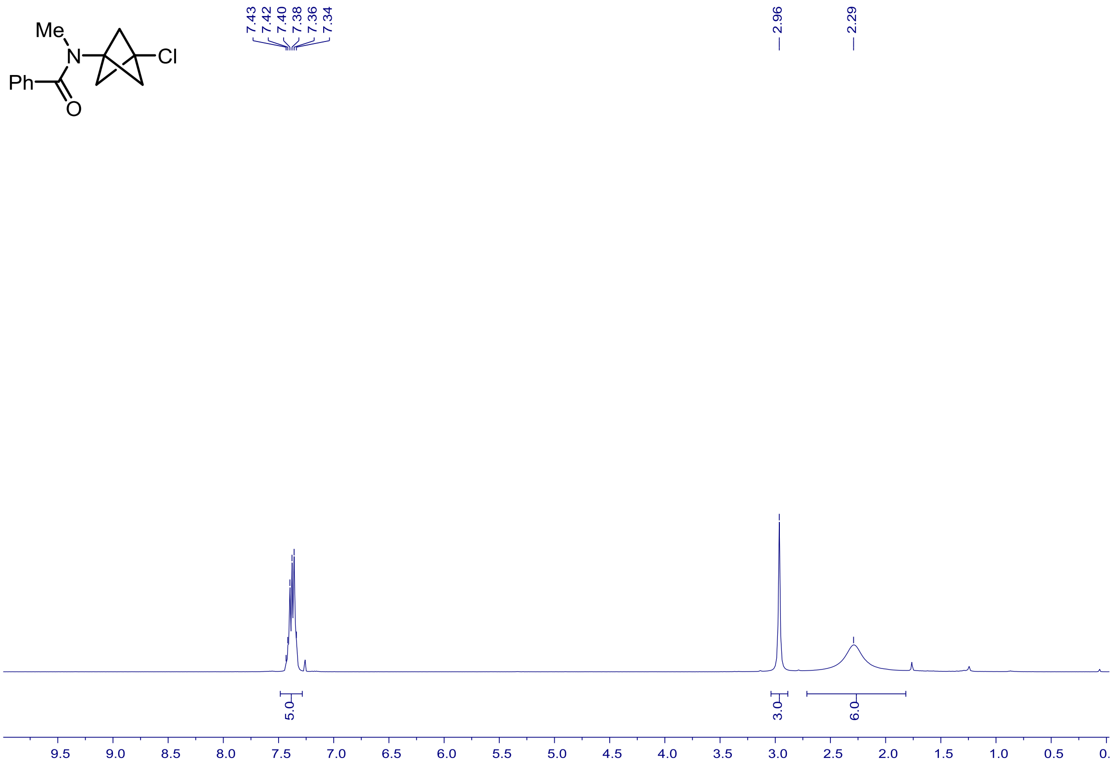

9– ^{13}C NMR (126 MHz, $CDCl_3$)

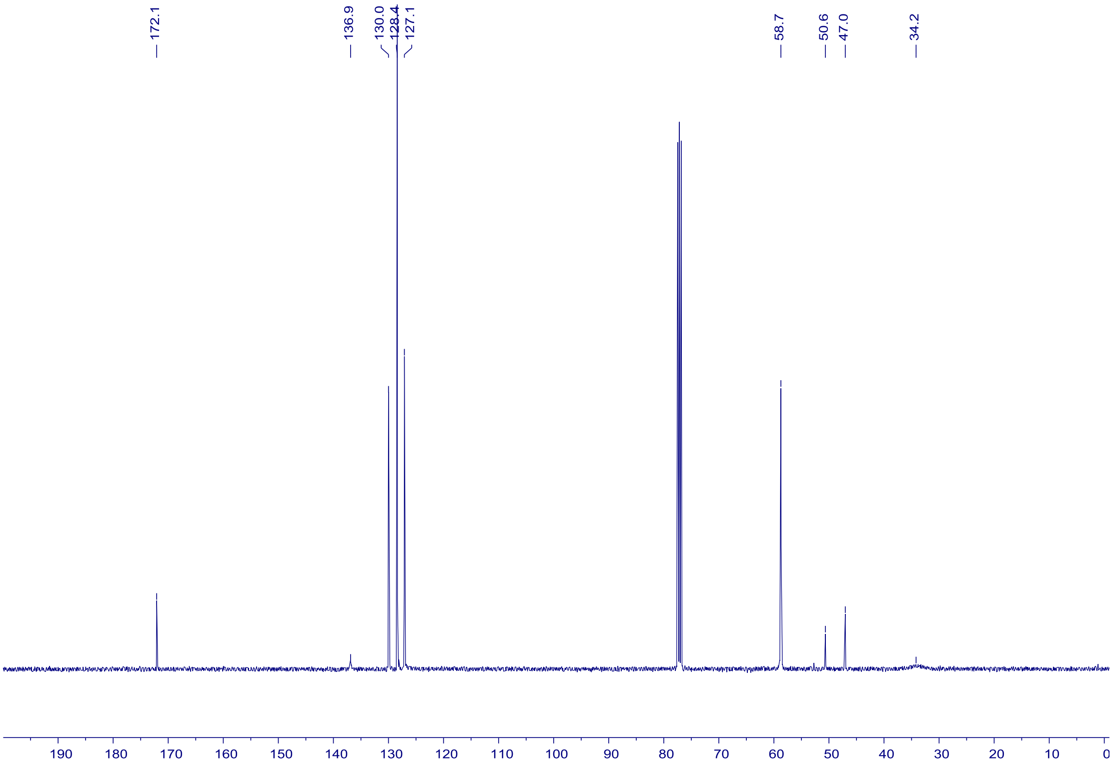

9 – HSQC (500 MHz, $CDCl_3$)

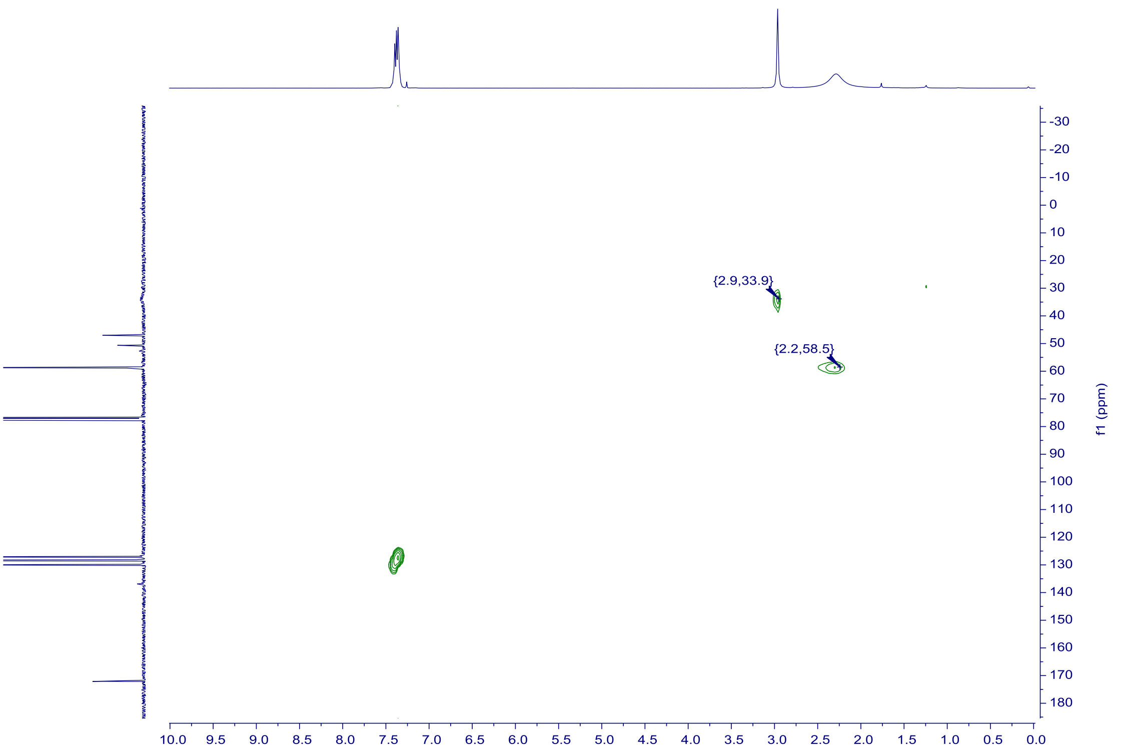

9 – HMBC (500 MHz, $CDCl_3$)

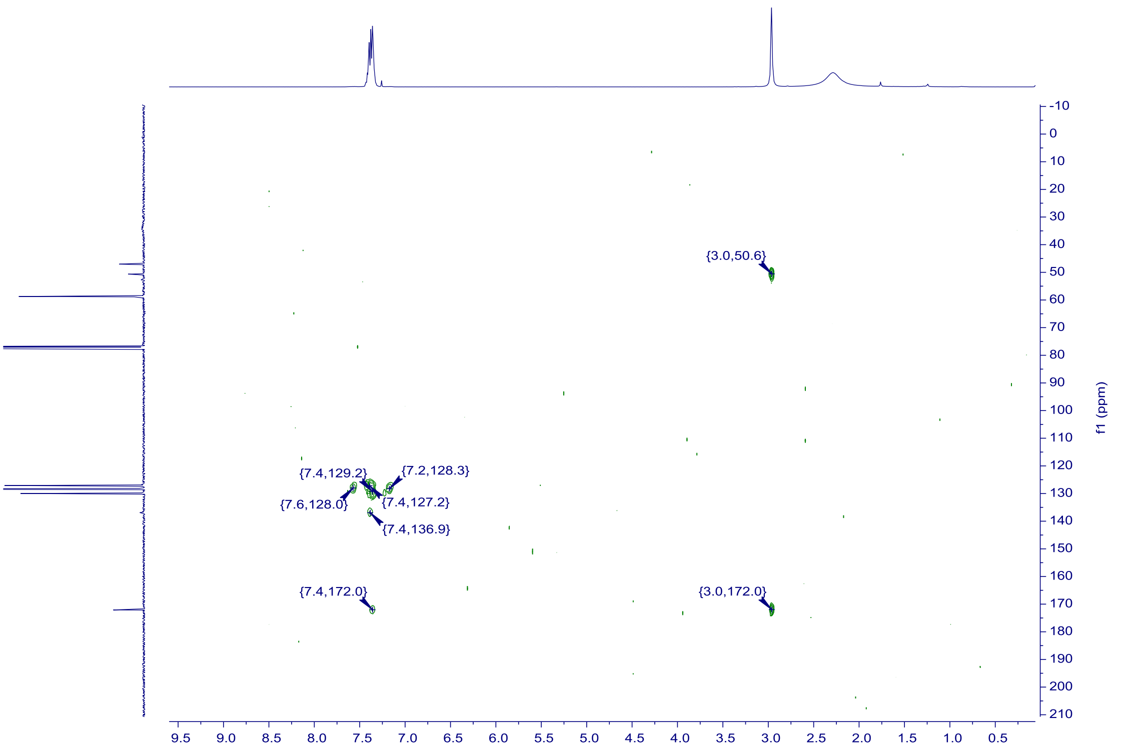

10 – ^{1}H NMR (400 MHz, $CDCl_3$)

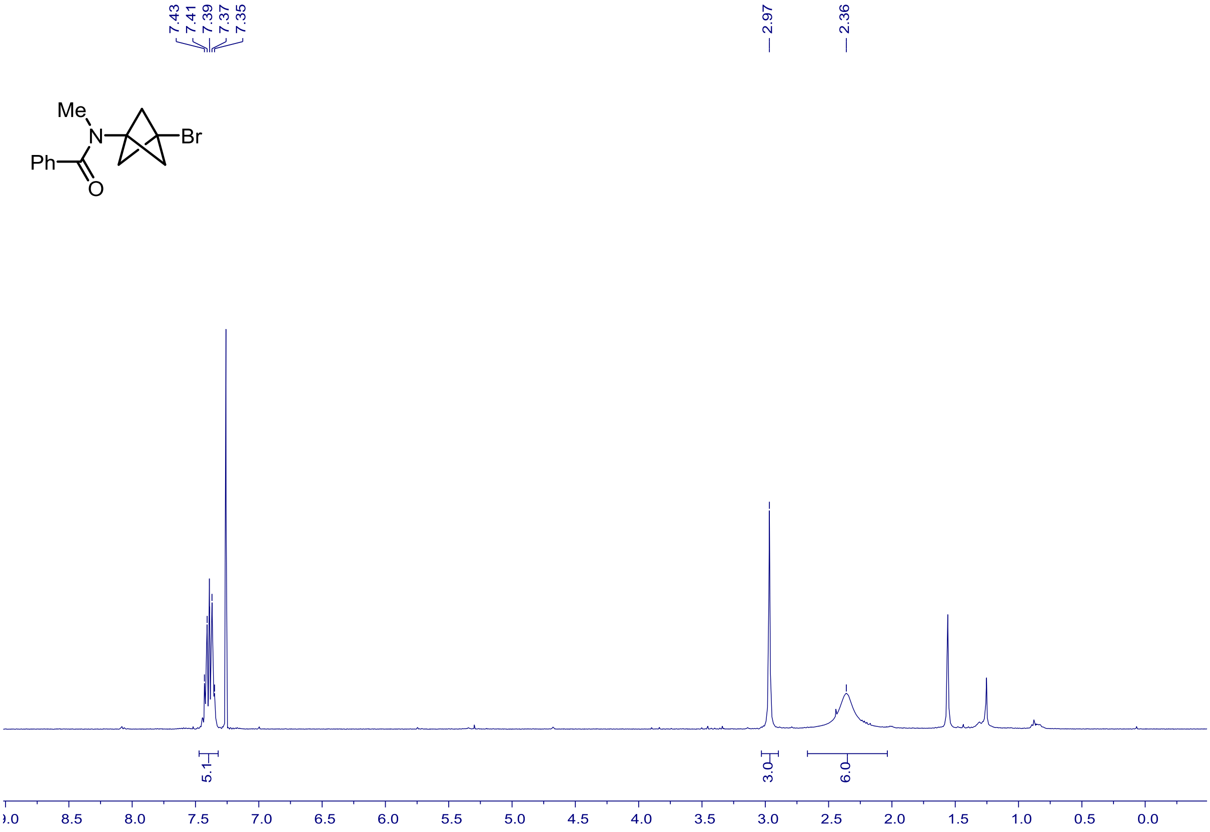

10 – ^{13}C NMR (101 MHz, $CDCl_3$)

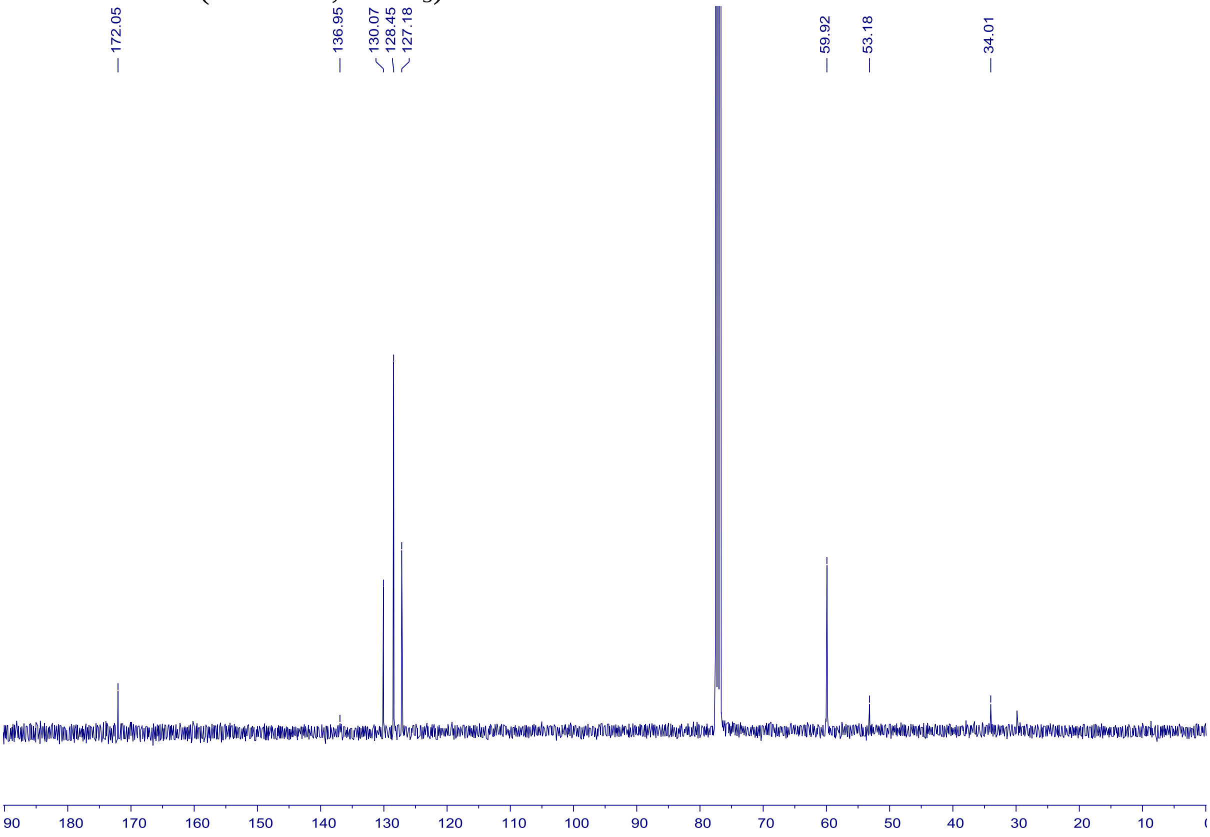

11 – ^{1}H NMR (500 MHz, $CDCl_3$)

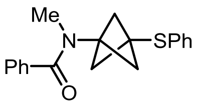

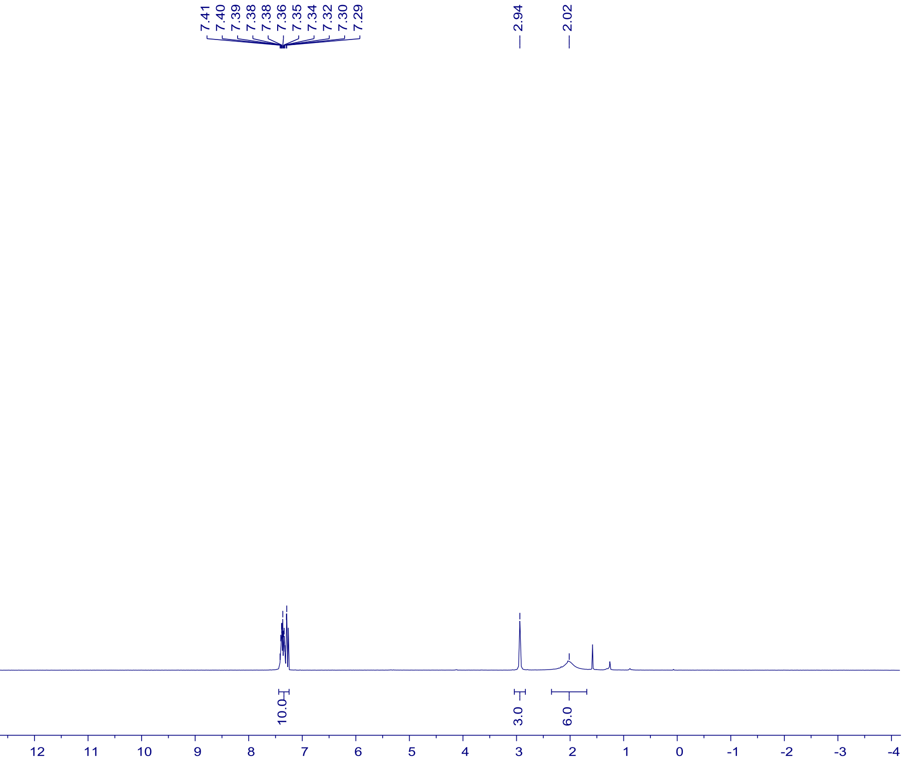

11 – ^{13}C NMR (126MHz, $CDCl_3$)

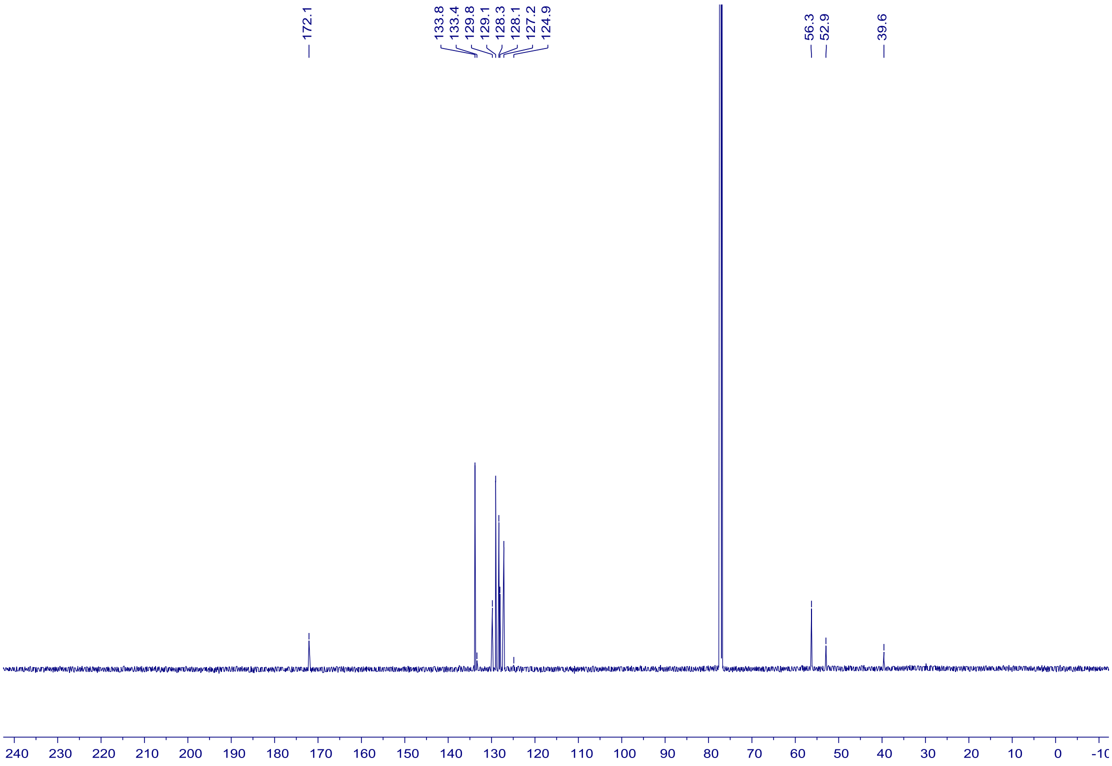

11 – HSQC (500 MHz, $CDCl_3$)

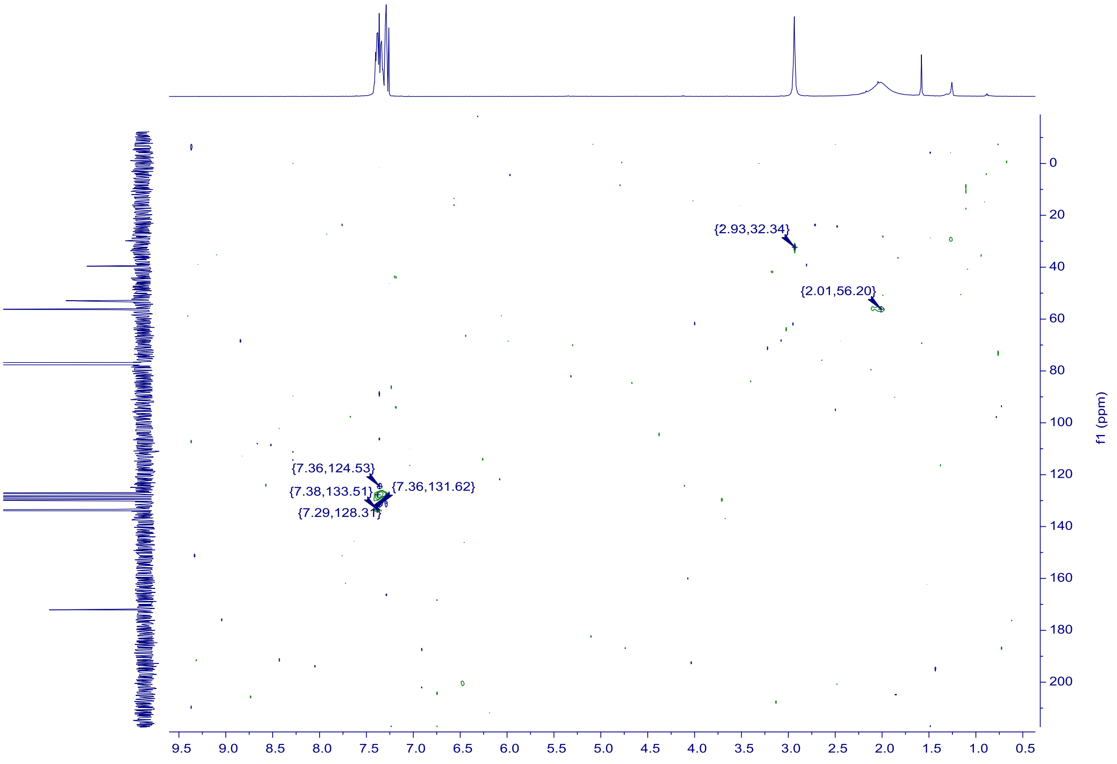

12 – ^{1}H NMR (500 MHz, $CDCl_3$)

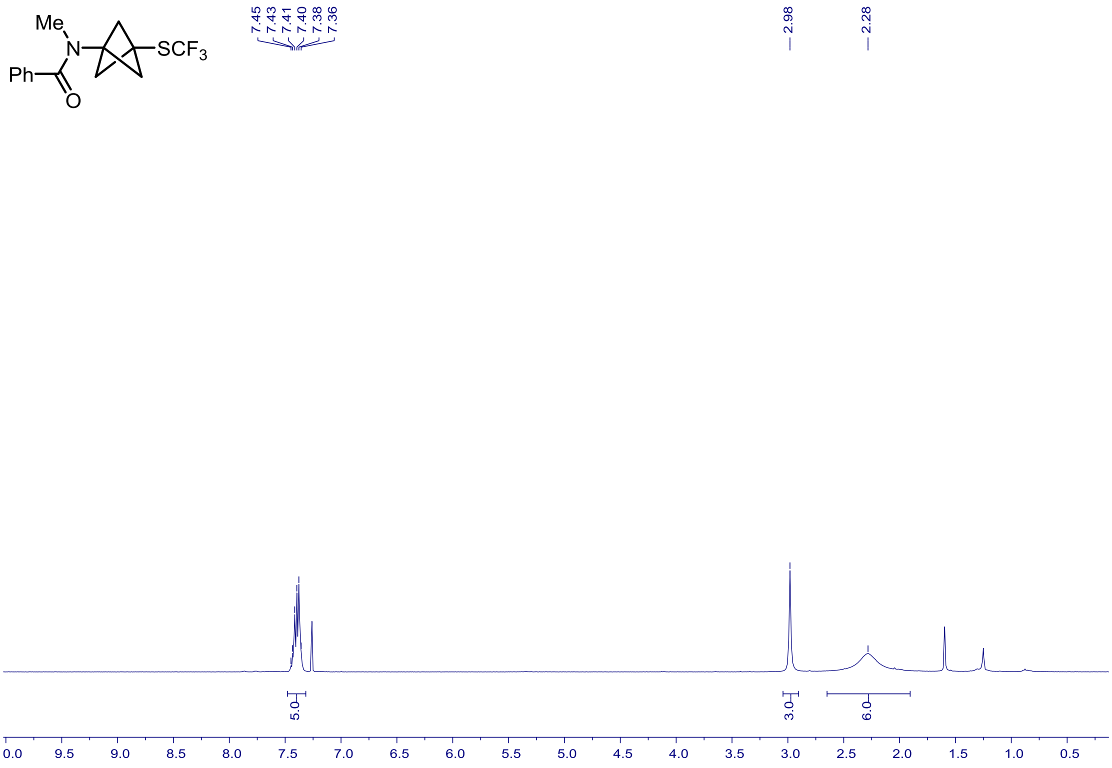

12 – ^{13}C NMR (126MHz, $CDCl_3$)

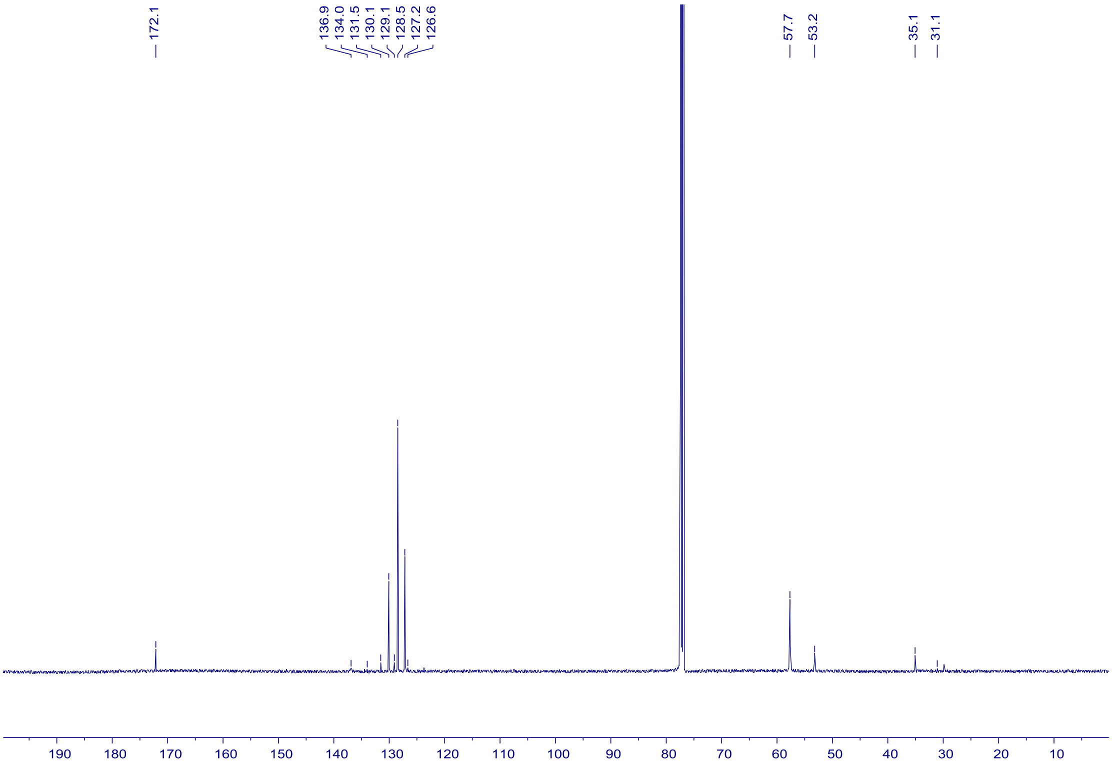

12 – HSQC (500 MHz, $CDCl_3$)

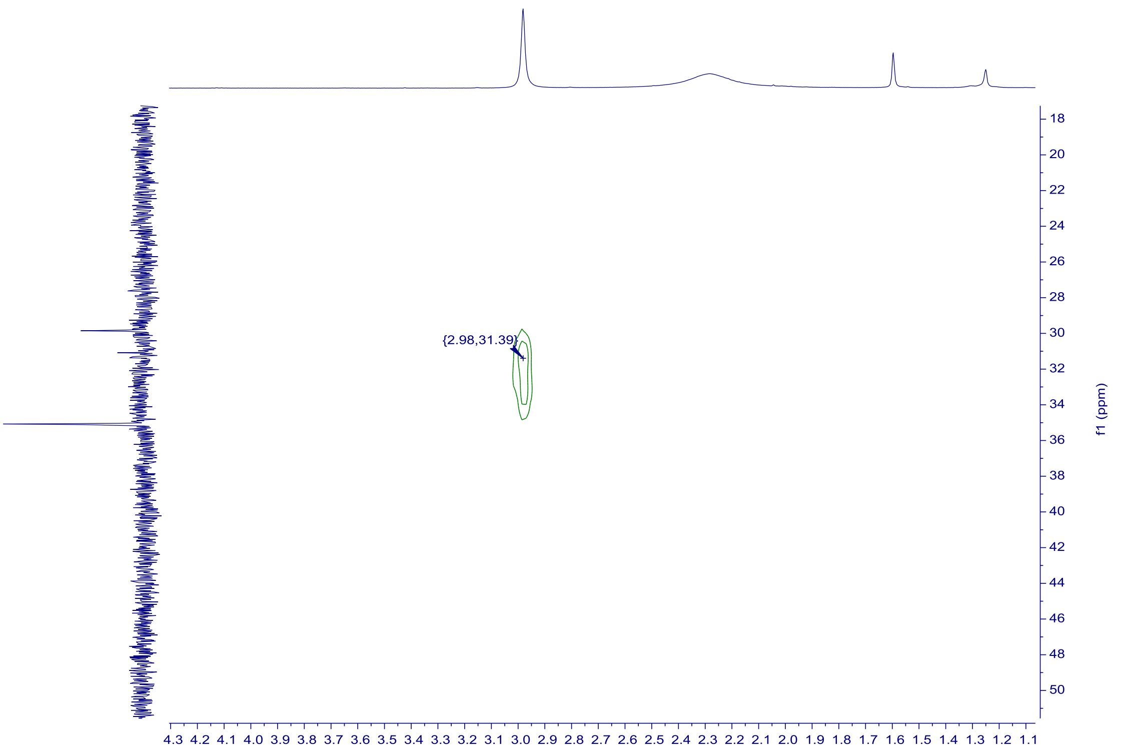

12 – HMBC (500 MHz, $CDCl_3$)

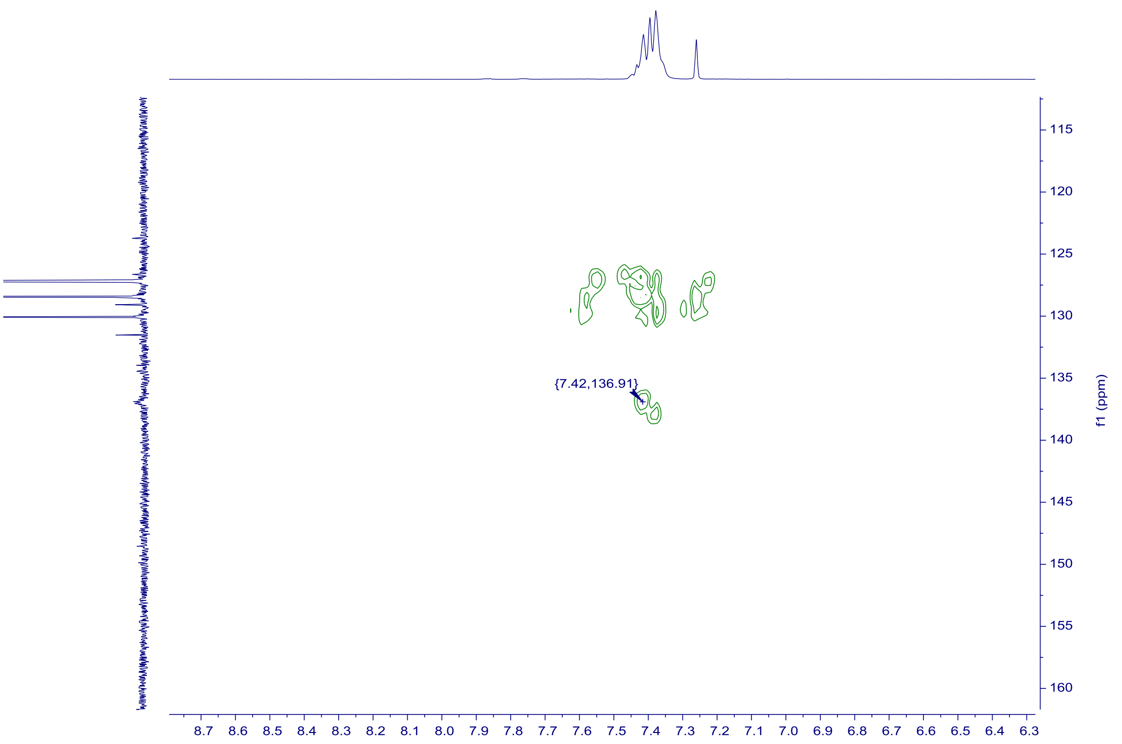

12 – ^{19}F NMR (376MHz, $CDCl_3$)

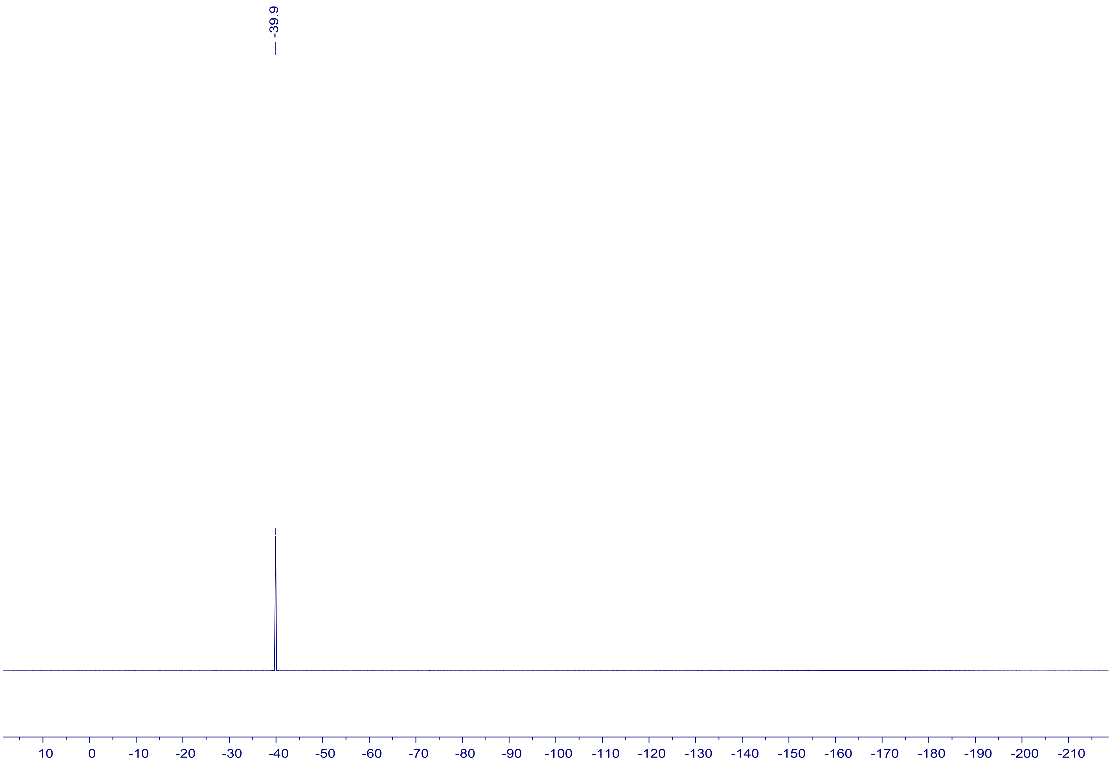

13 – ^{1}H NMR (400 MHz, $CDCl_3$)

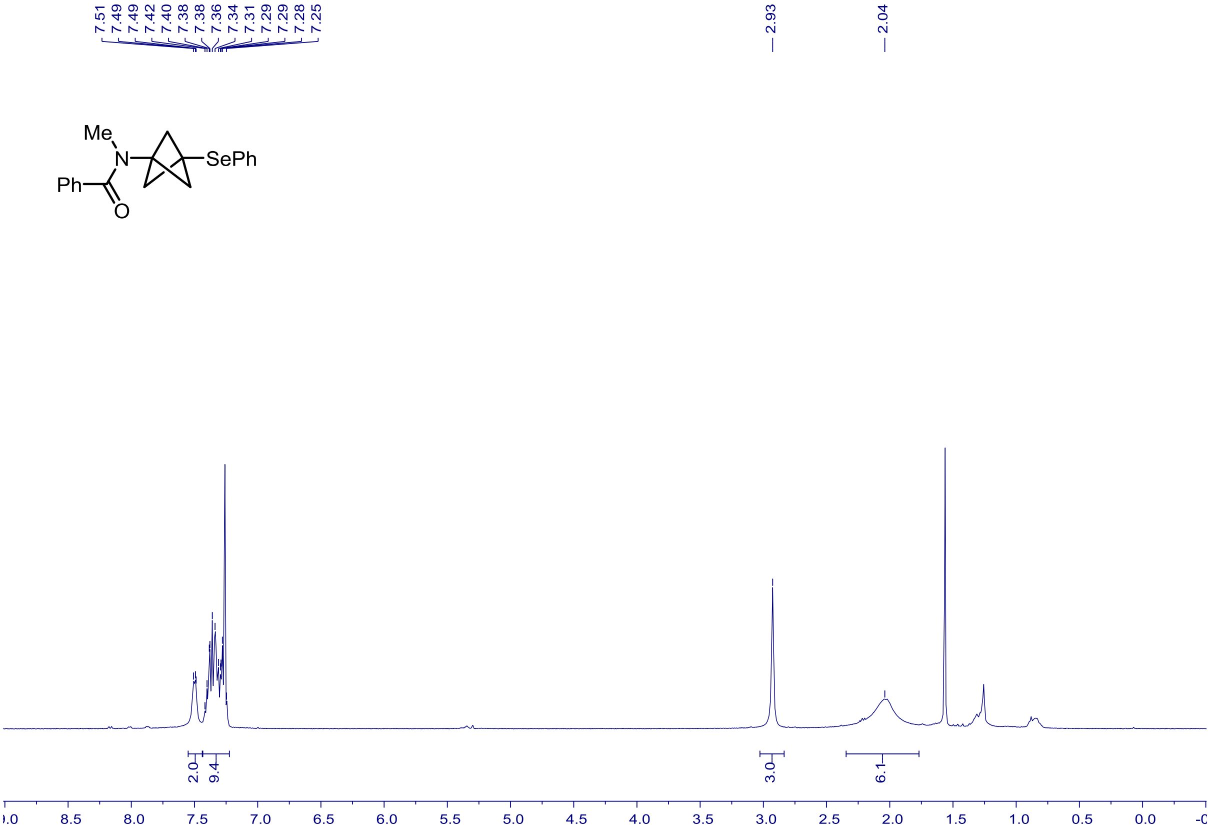

13 – ^{13}C NMR (101 MHz, $CDCl_3$)

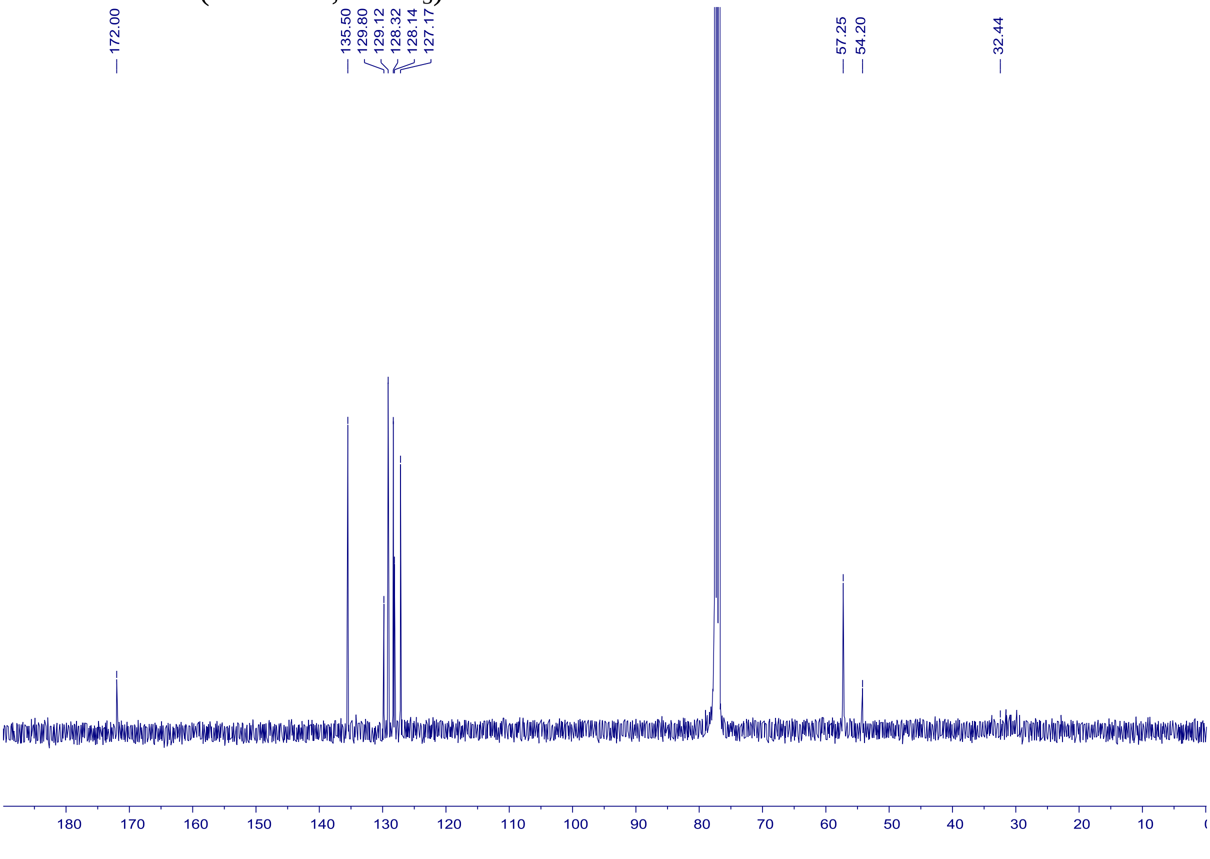

14 – ^{1}H NMR (500 MHz, $CDCl_3$

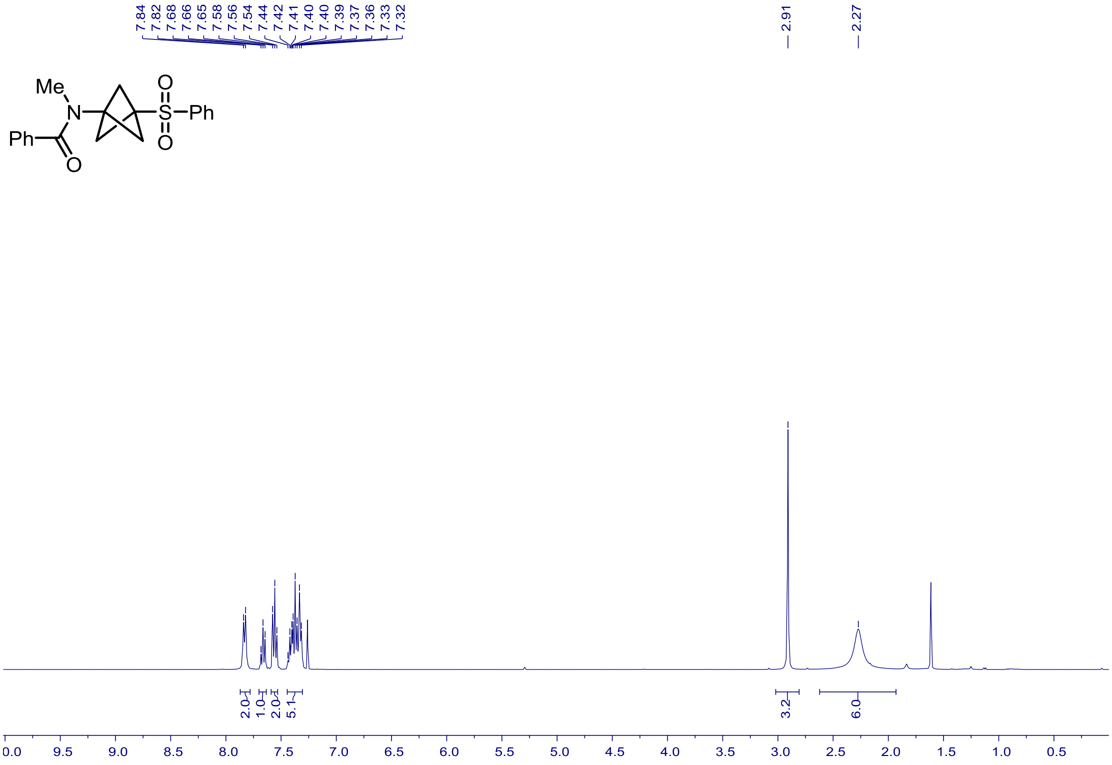

14 – ^{13}C NMR (126MHz, $CDCl_3$)

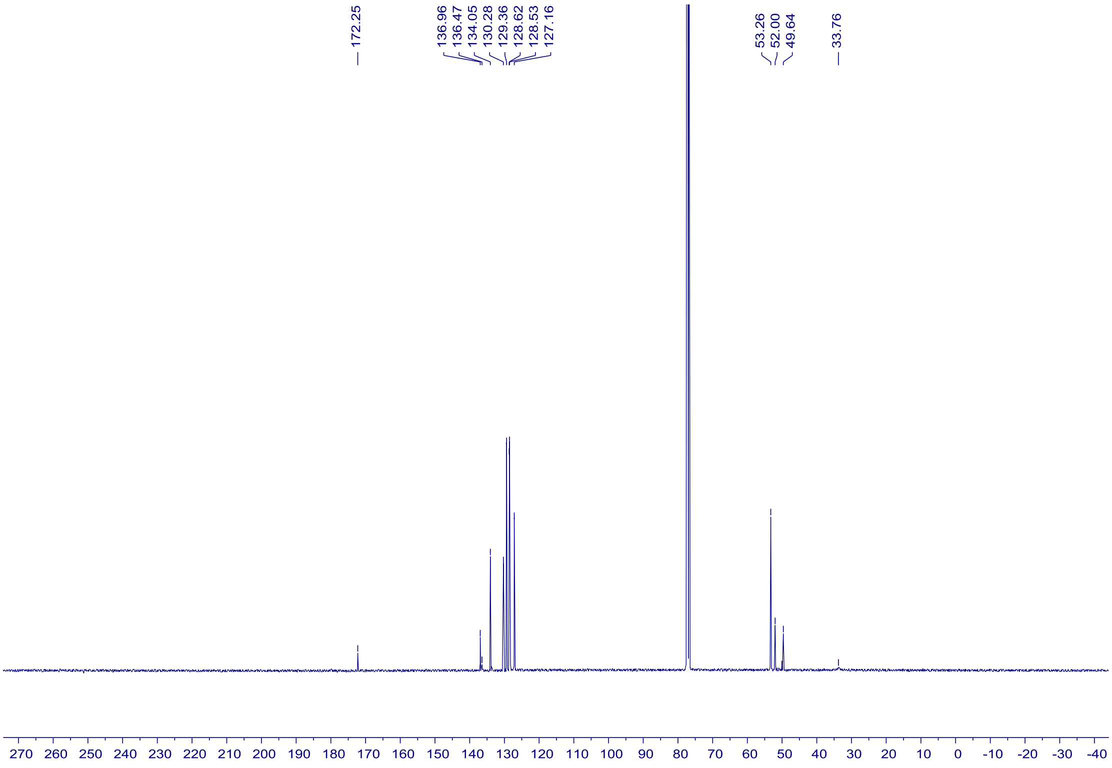

14 – HSQC (500 MHz, $CDCl_3$

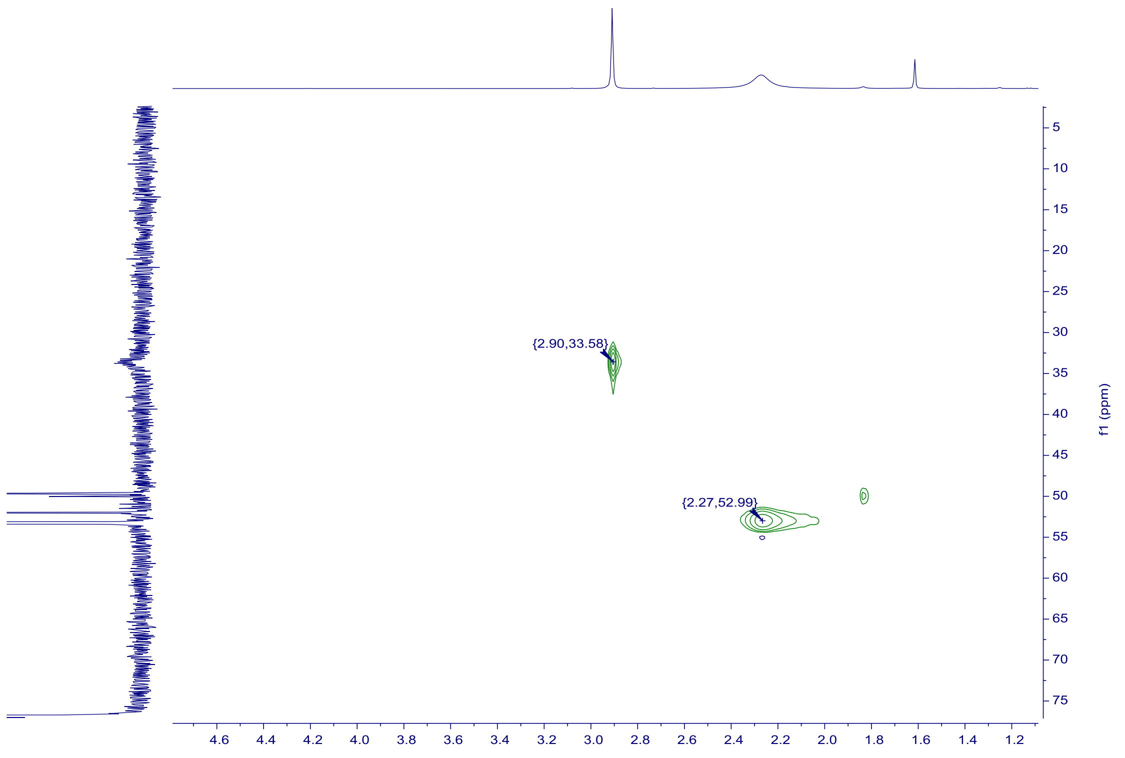

14 – HMBC (500 MHz, $CDCl_3$)

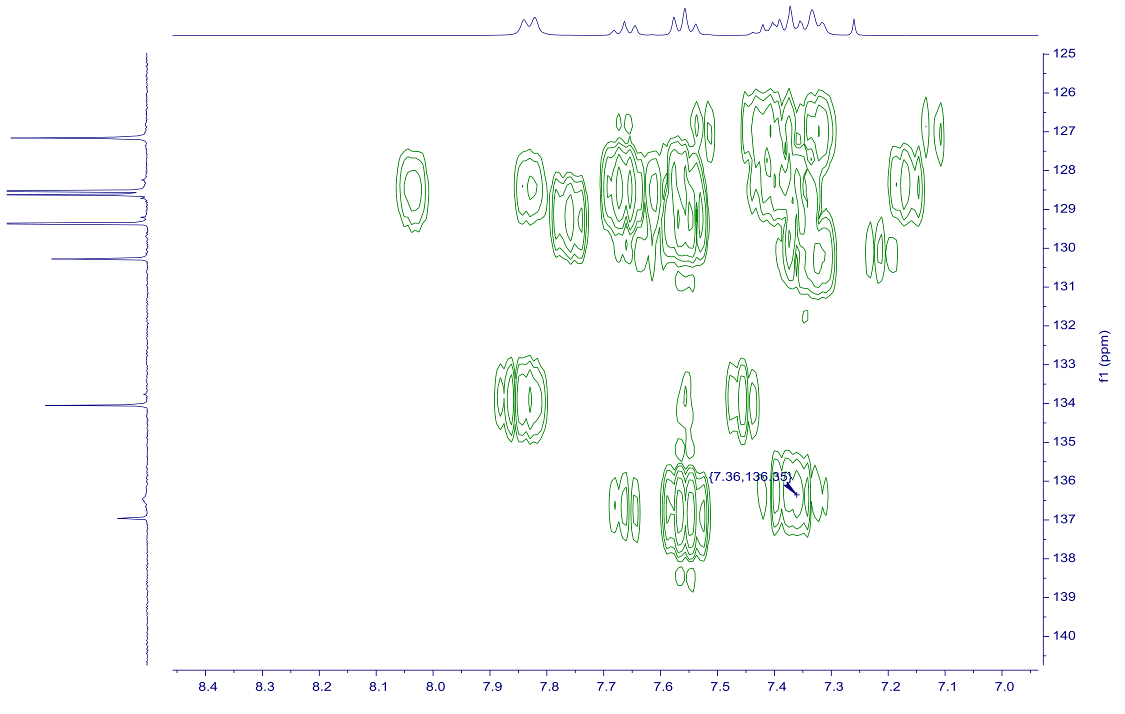

15 – ^{1}H NMR (500 MHz, $CDCl_3$)

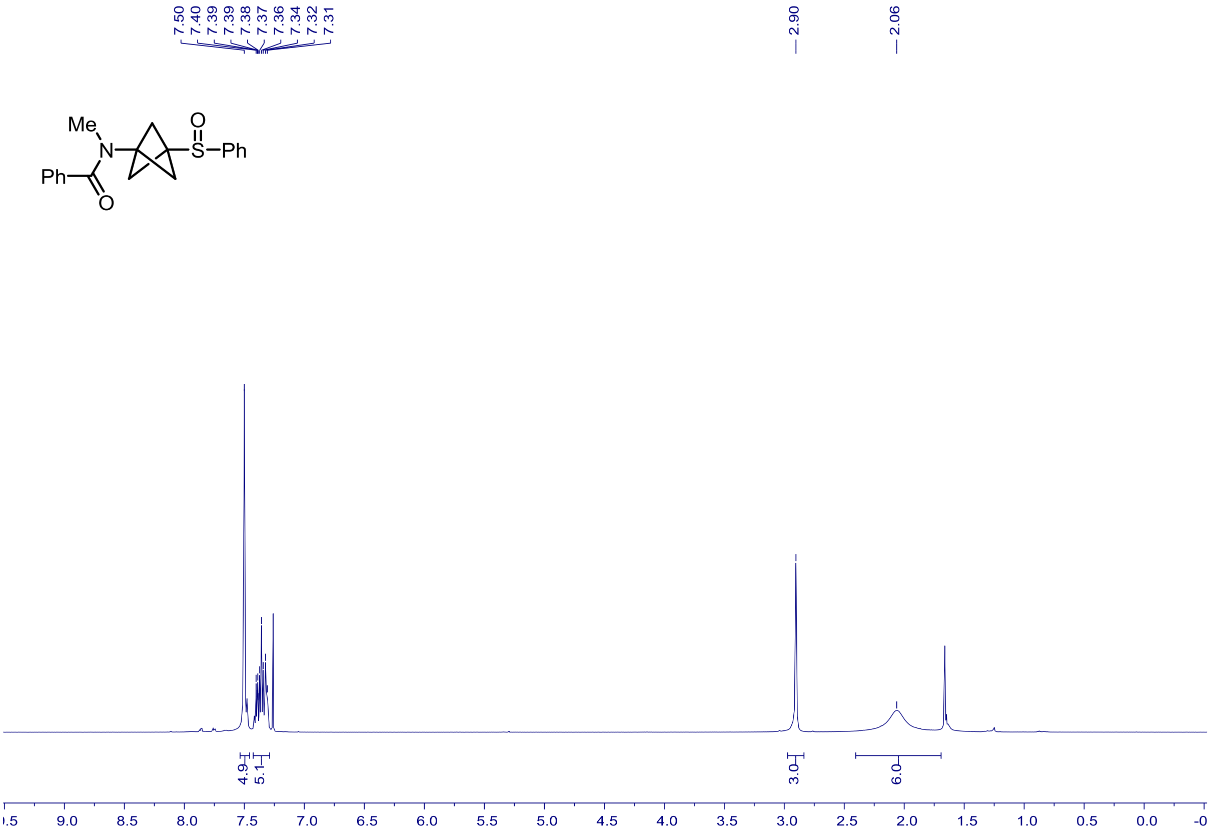

15 – ^{13}C NMR (126 MHz, $CDCl_3$)

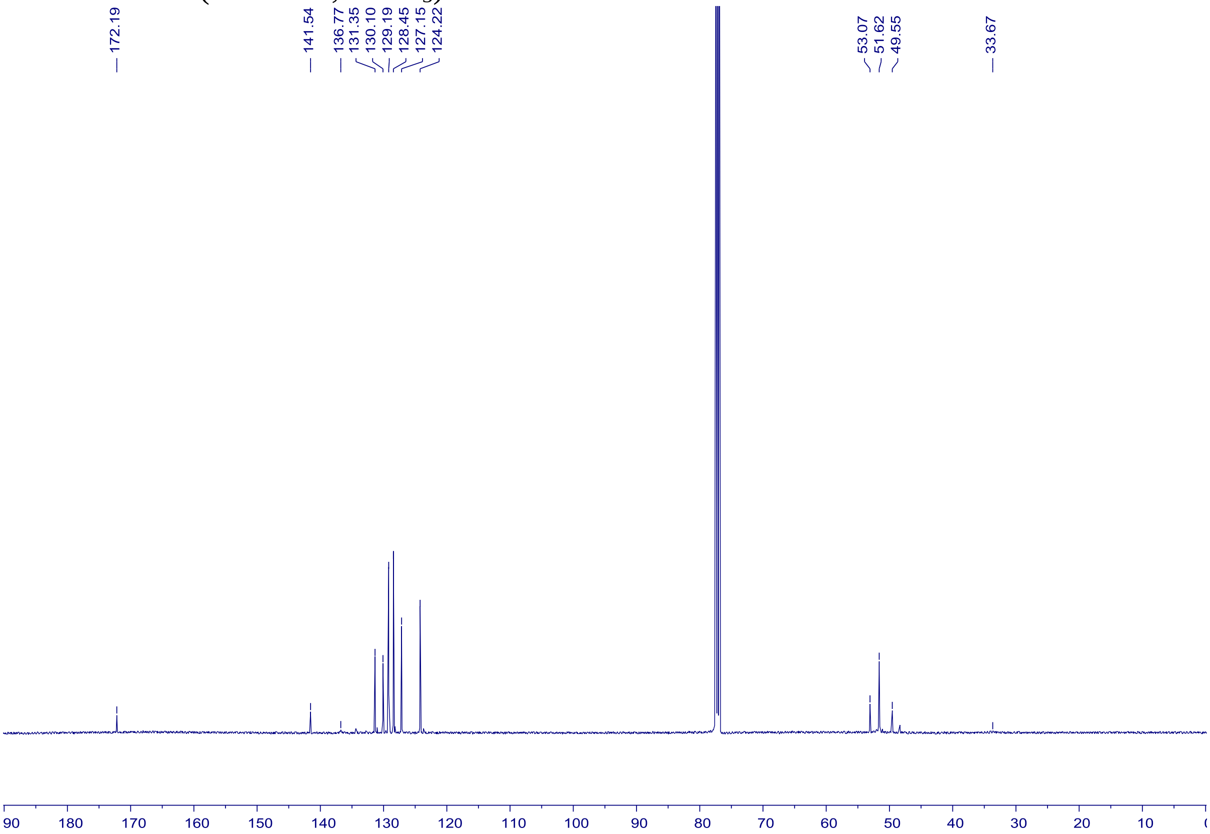

16 – ^{1}H NMR (500 MHz, $CDCl_3$)

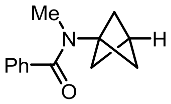

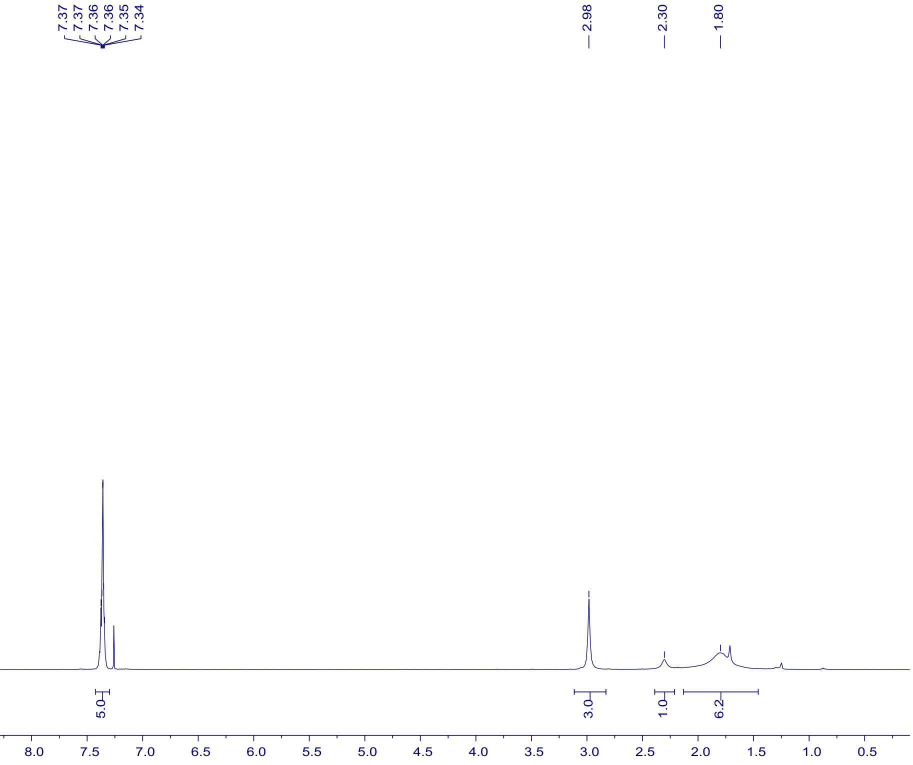

16 – ^{13}C NMR (126MHz, $CDCl_3$)

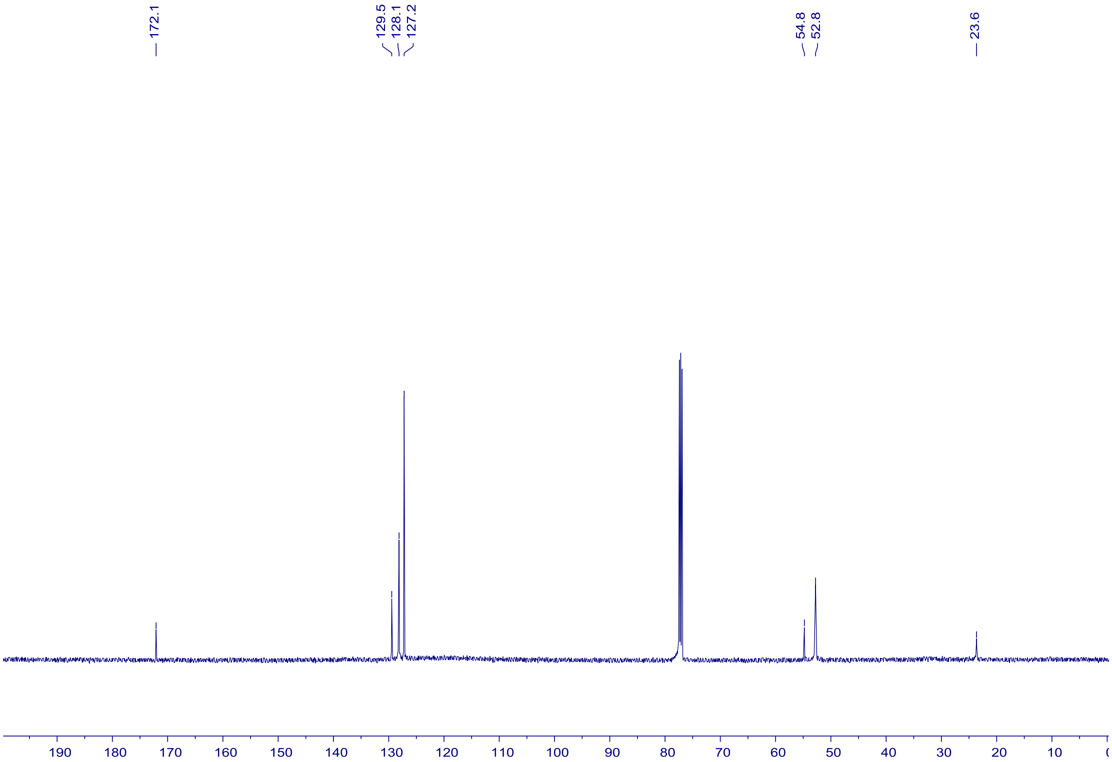

16 – HSQC (500 MHz, $CDCl_3$)

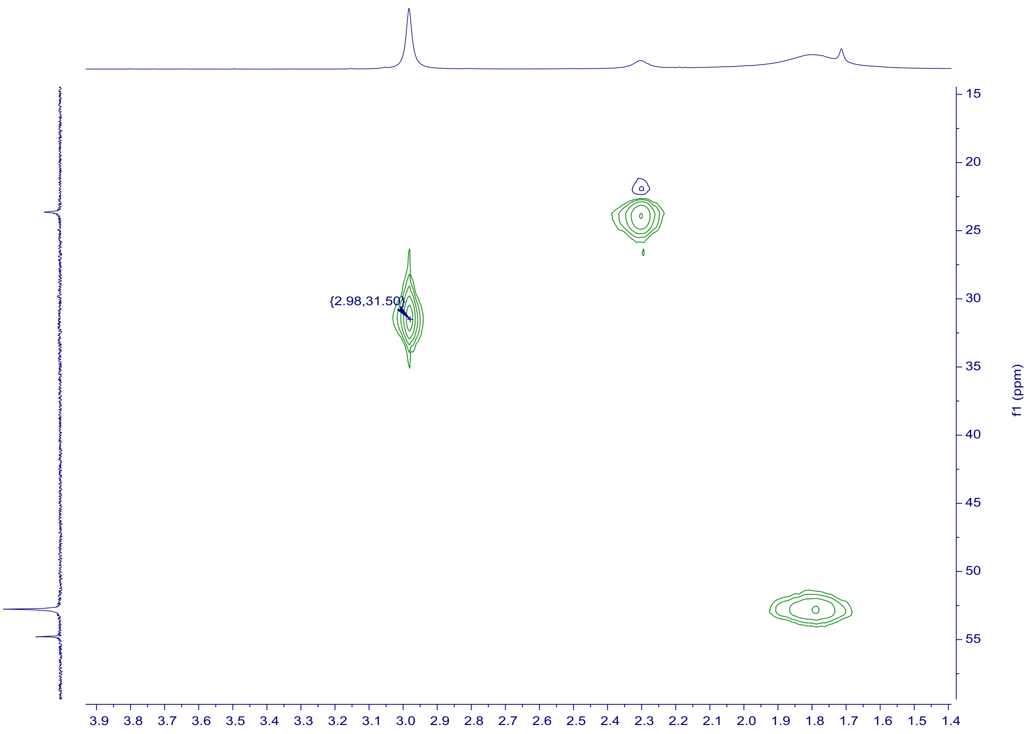

16 – HMBC (500 MHz, $CDCl_3$)

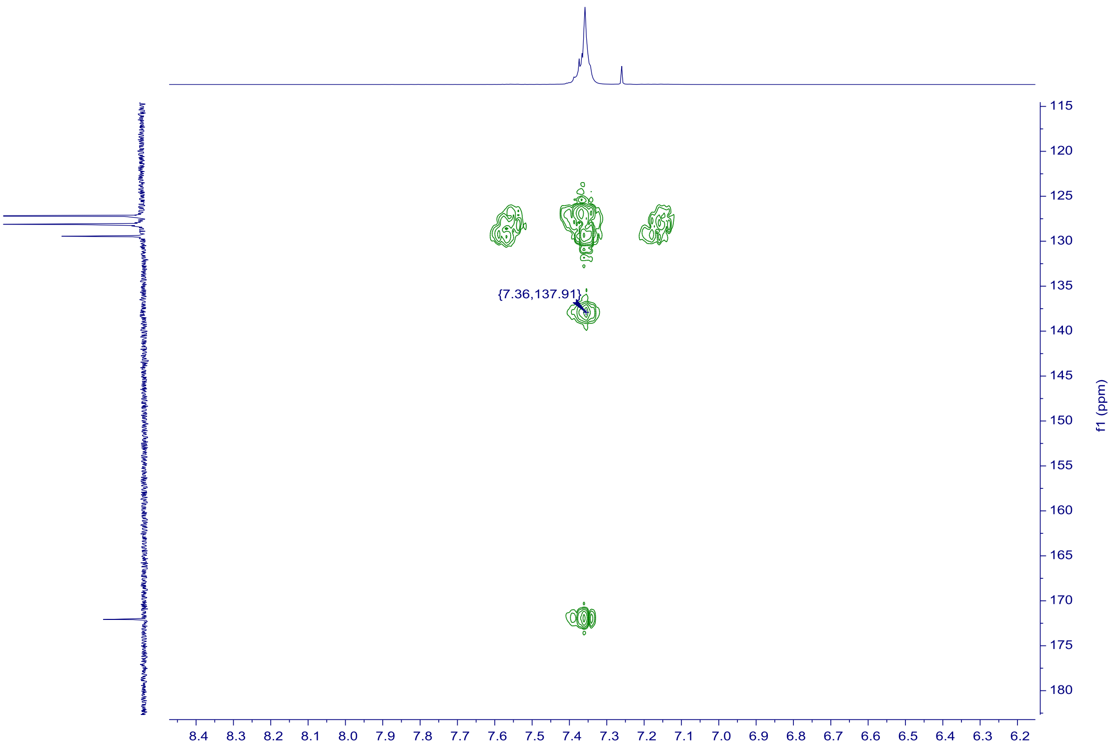

17 – ^{1}H NMR (400 MHz, $CDCl_3$)

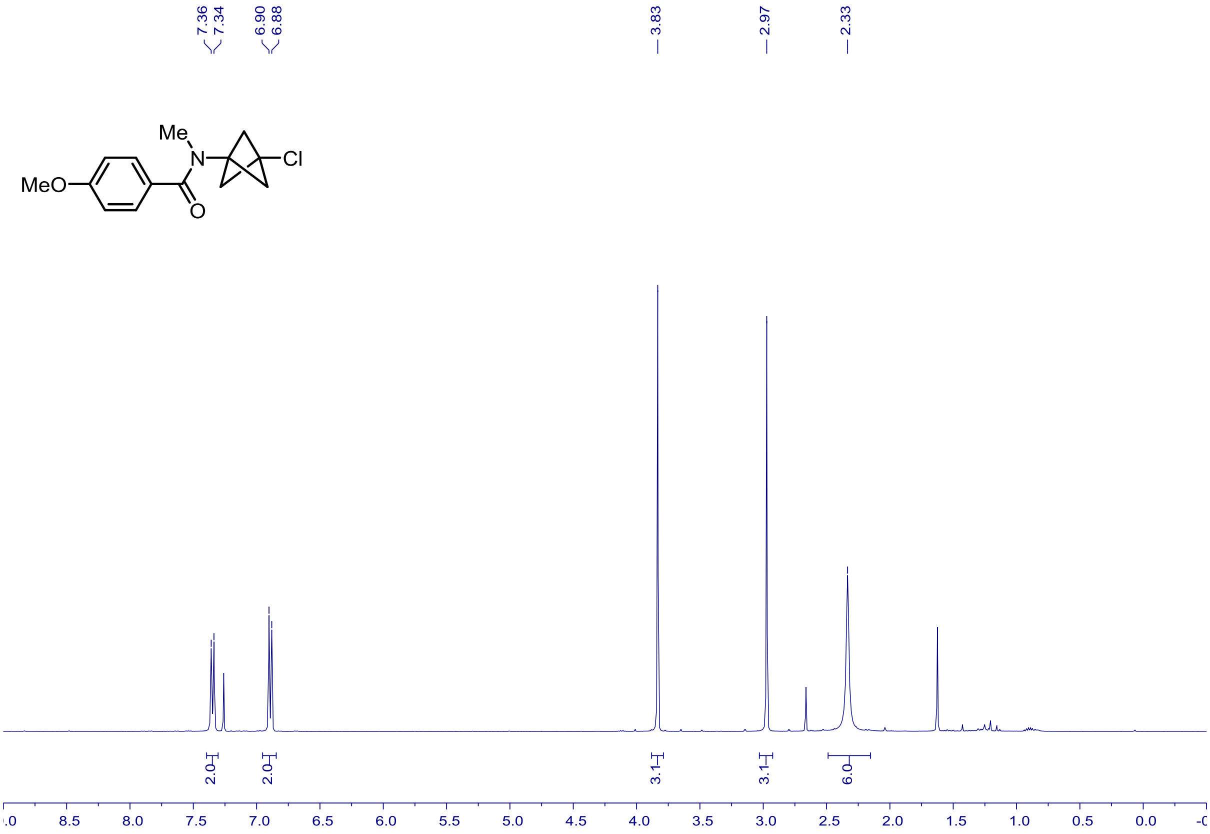

17 – ^{13}C NMR (101 MHz, $CDCl_3$)

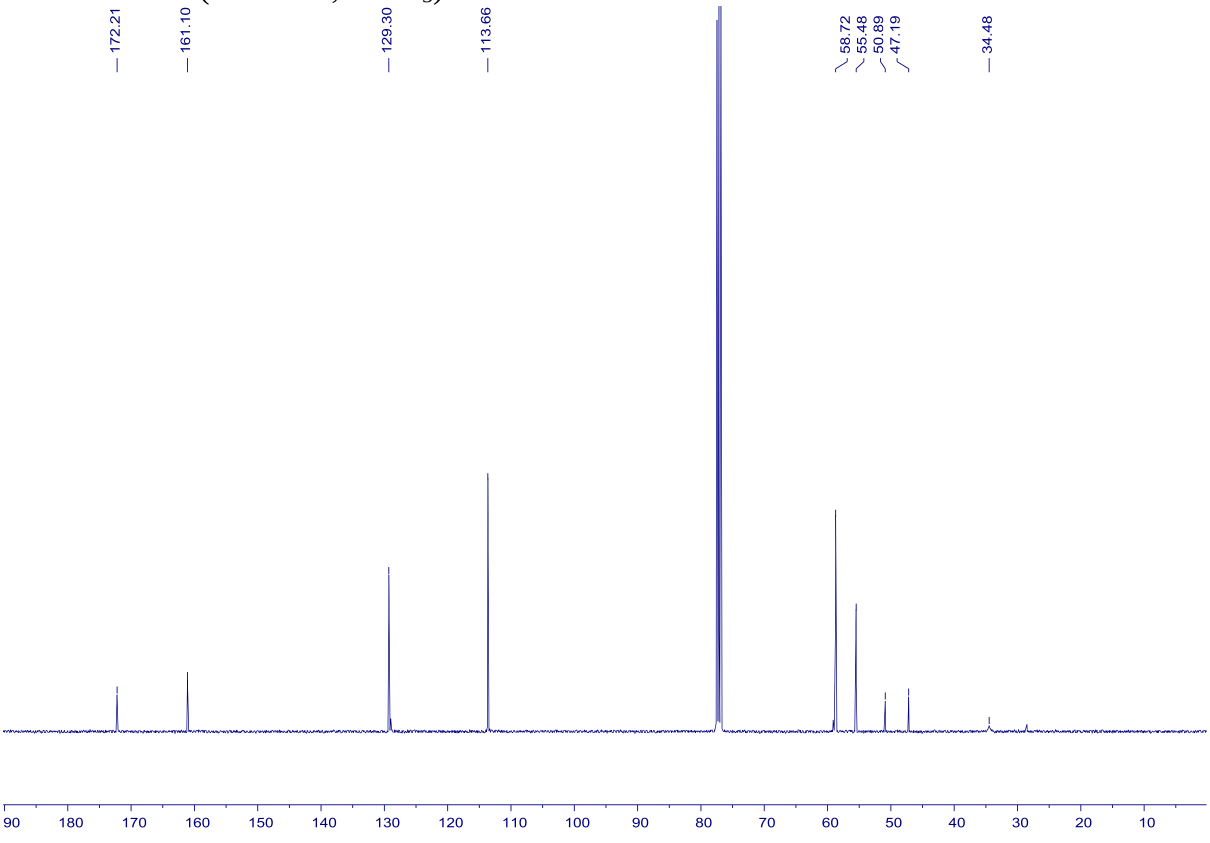

18 – ^{1}H NMR (500 MHz, $CDCl_3$)

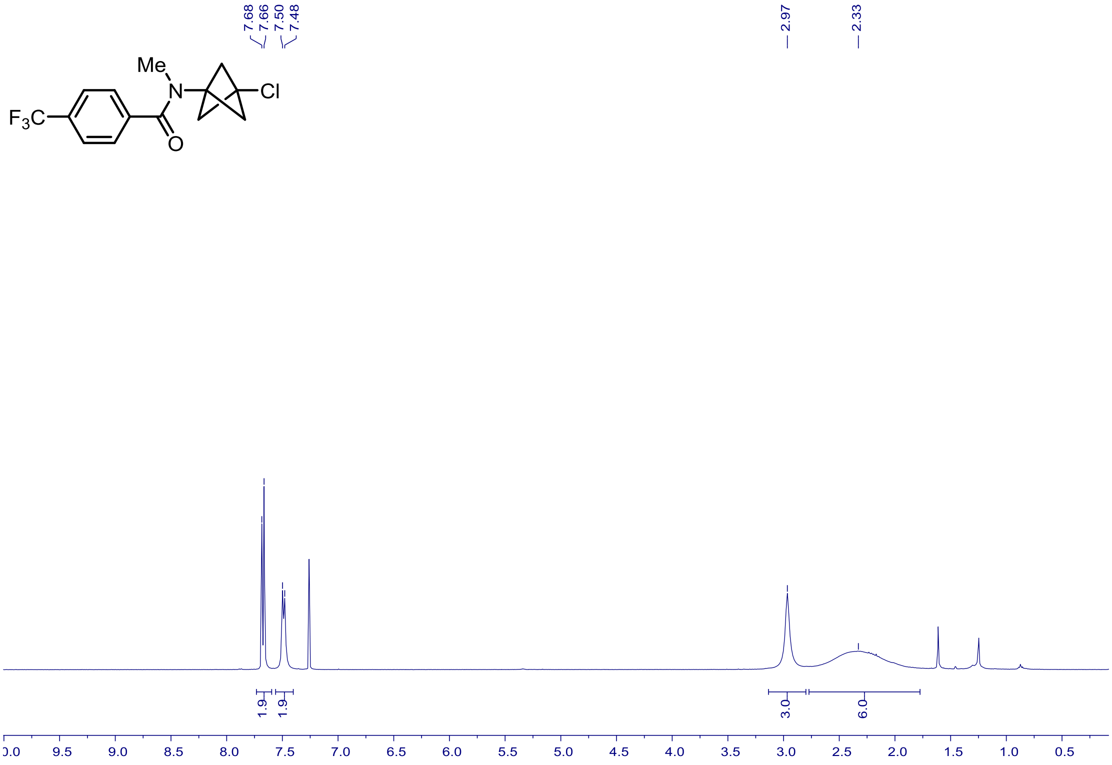

18 – ^{13}C NMR (126MHz, $CDCl_3$)

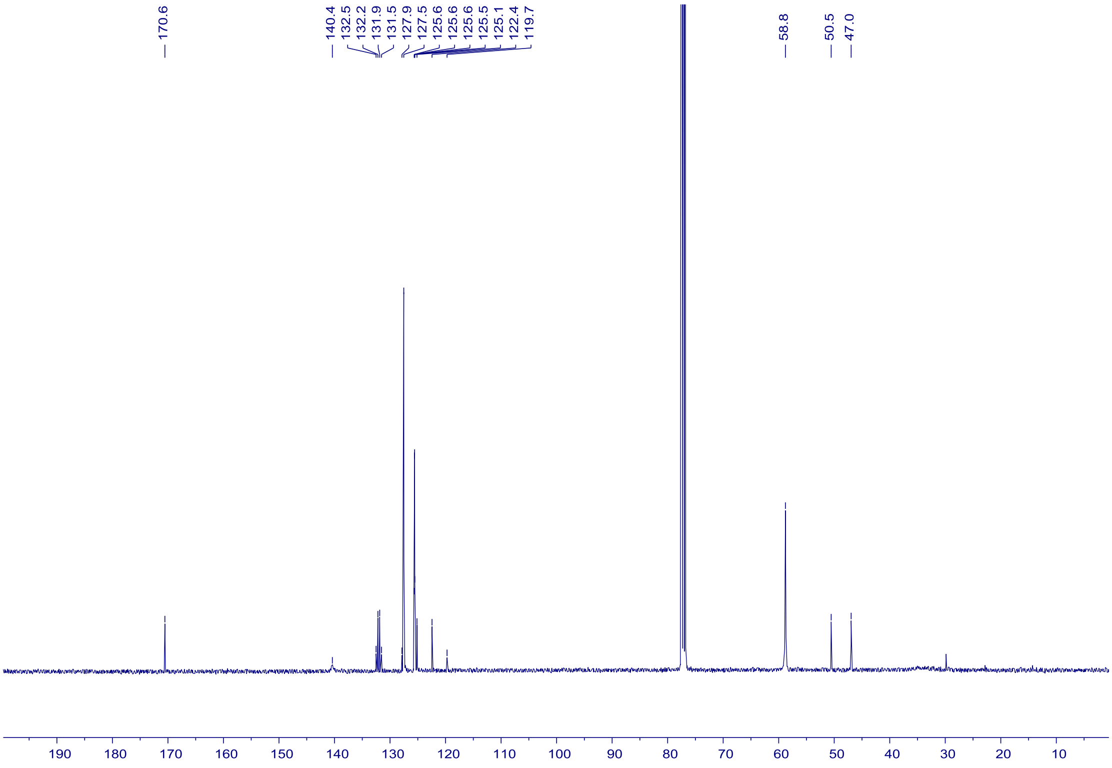

18 – HSQC (500 MHz, $CDCl_3$)

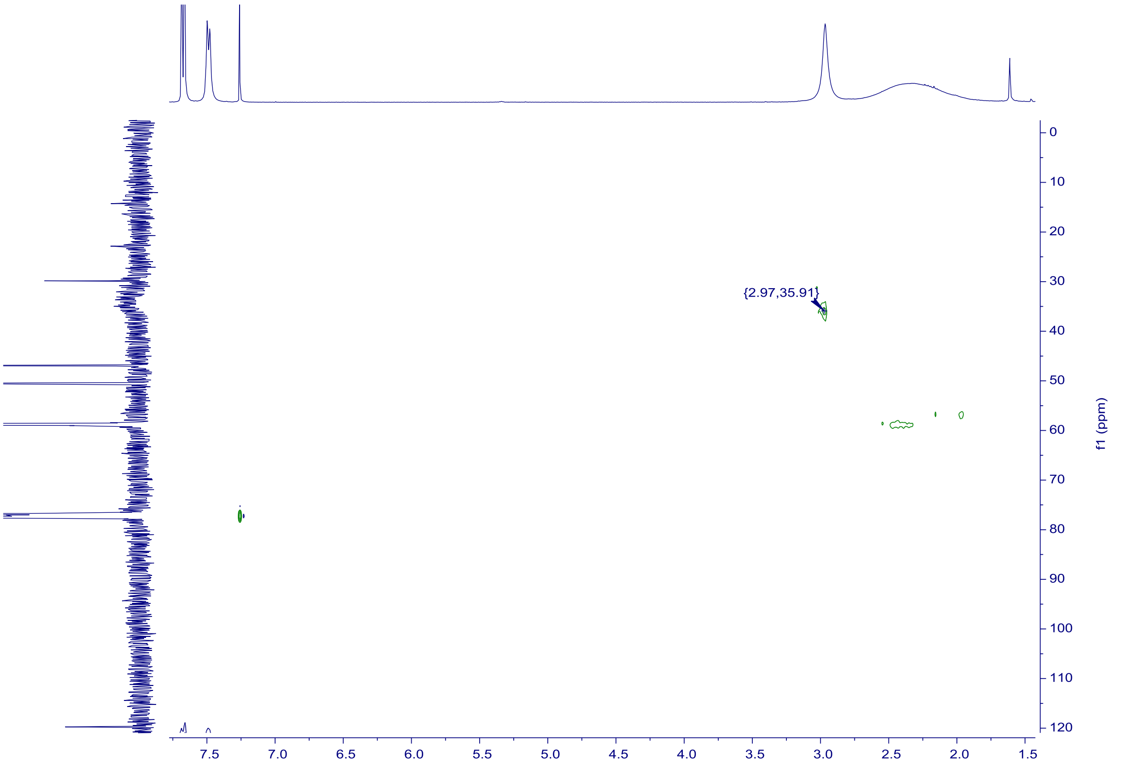

18 – HMBC (500 MHz, $CDCl_3$)

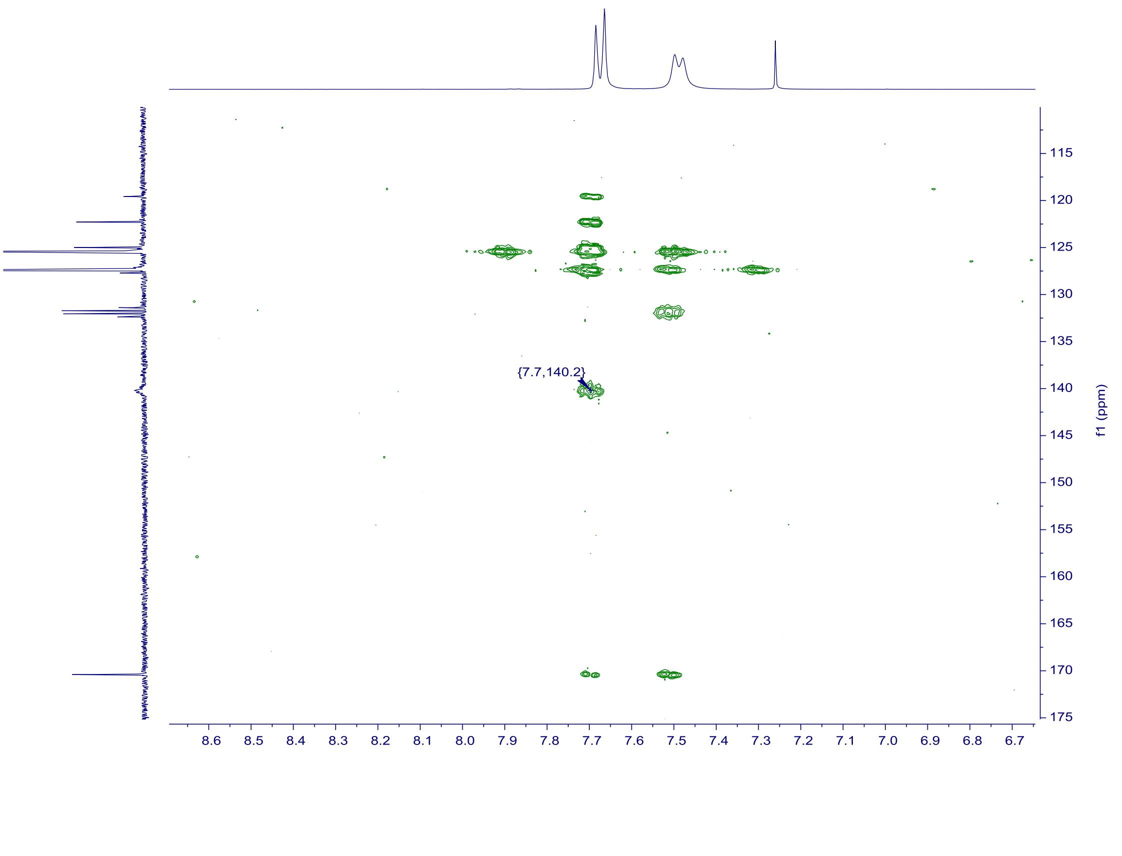

18 – ^{19}F NMR (376MHz, $CDCl_3$)

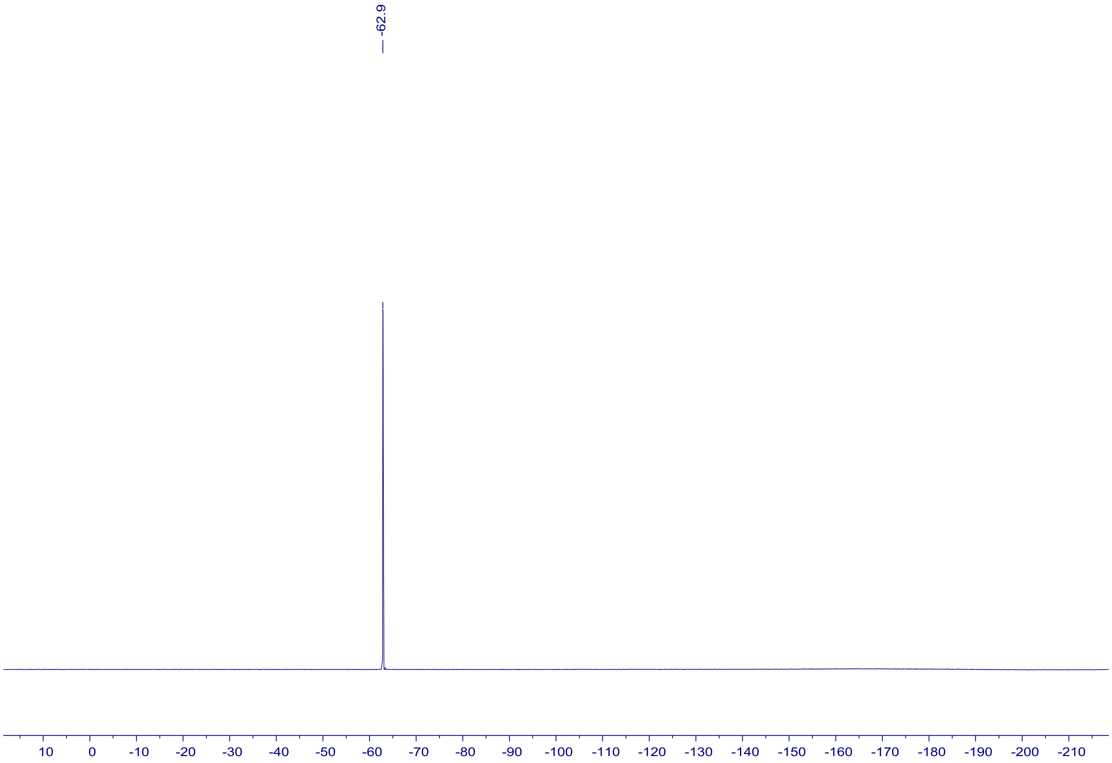

19 – ^{1}H NMR (500 MHz, $CDCl_3$)

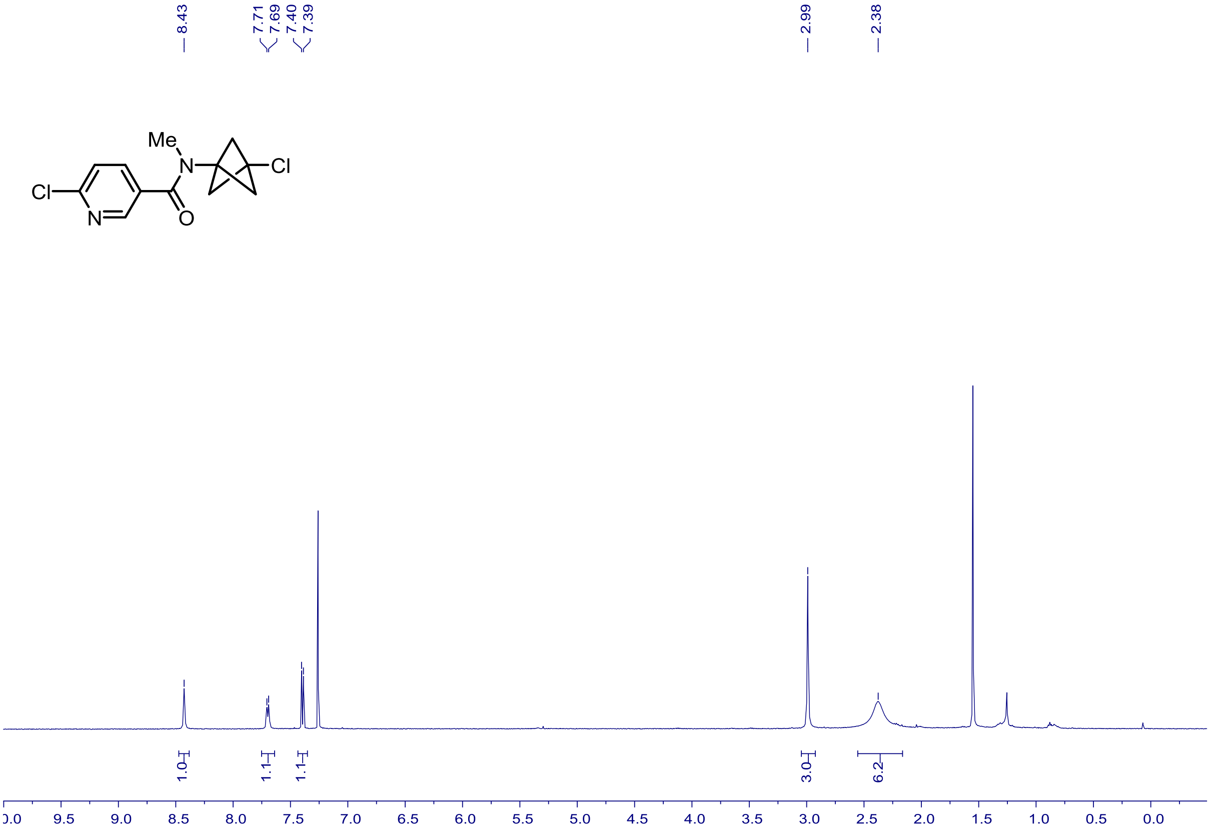

19 – ^{13}C NMR (101 MHz, $CDCl_3$)

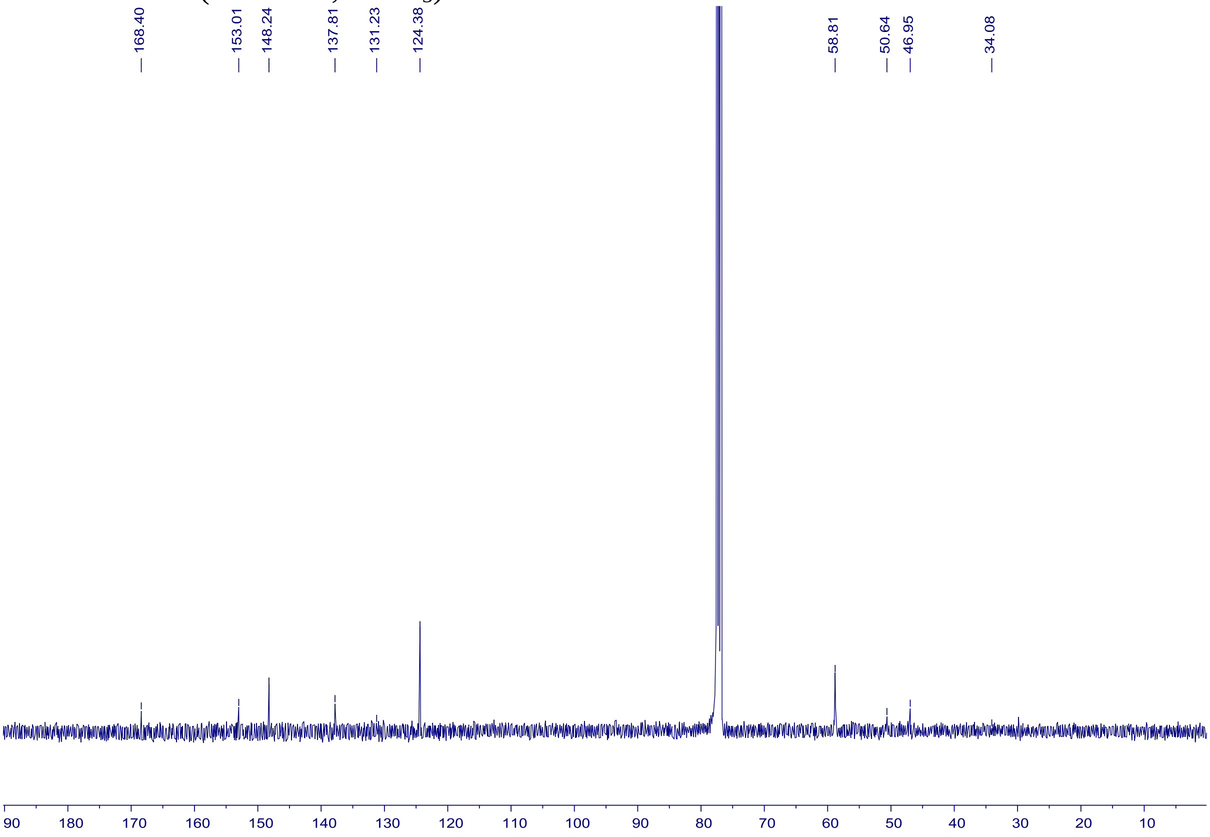

20 – ^{1}H NMR (400 MHz, $CDCl_3$)

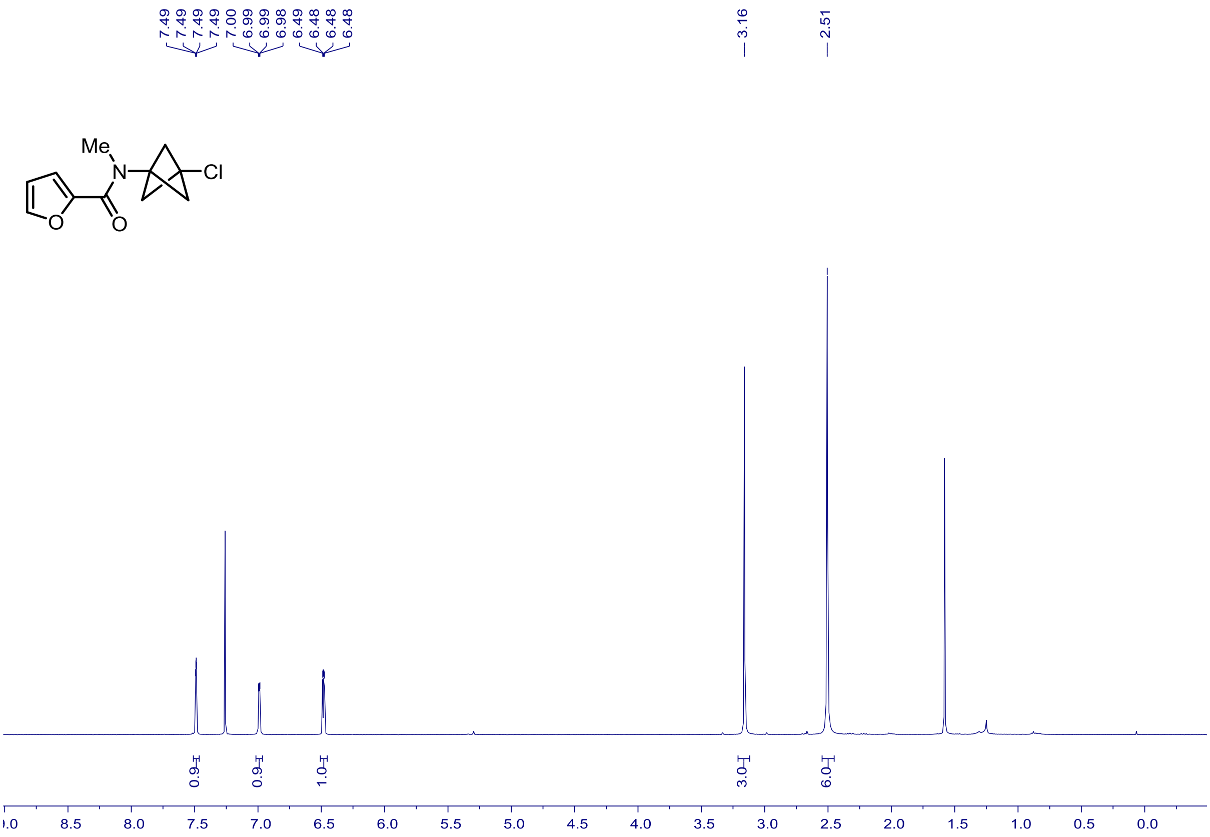

20 – ^{13}C NMR (101 MHz, $CDCl_3$)

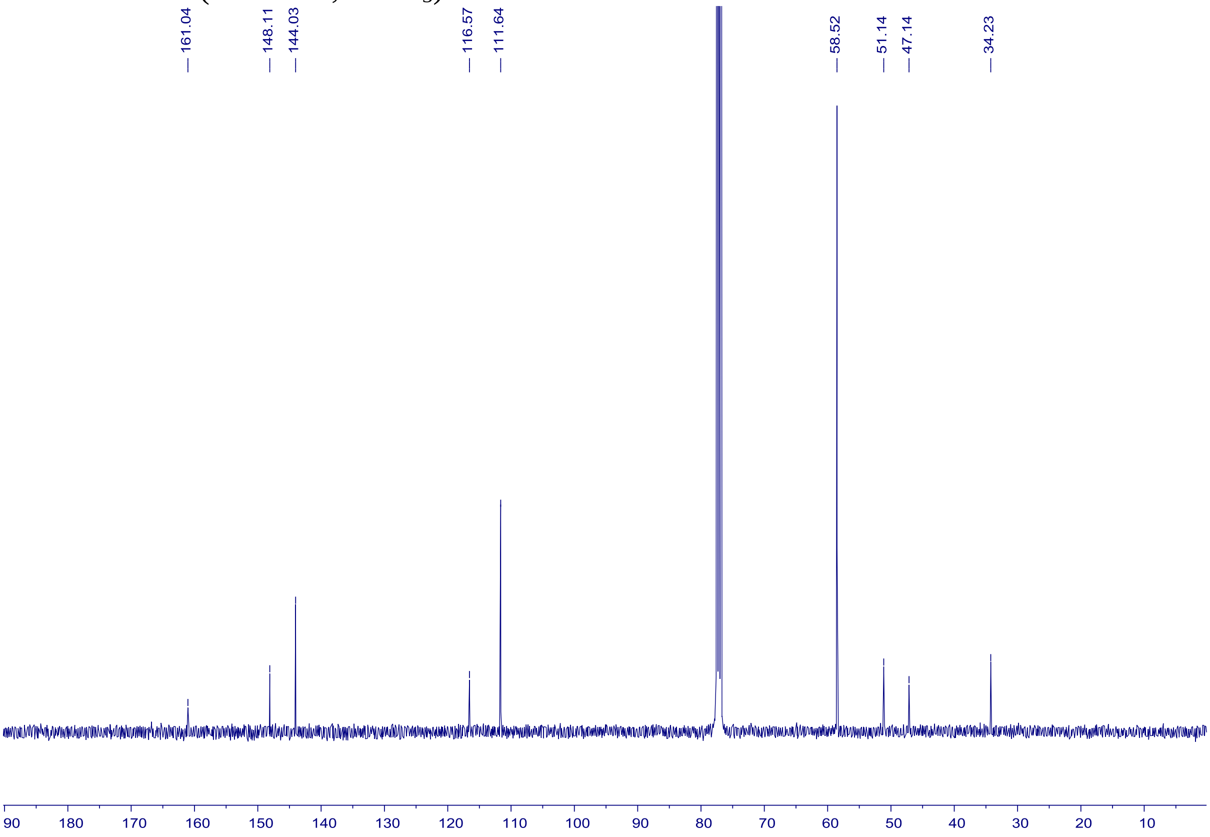

21 – ^{1}H NMR (500 MHz, $CDCl_3$)

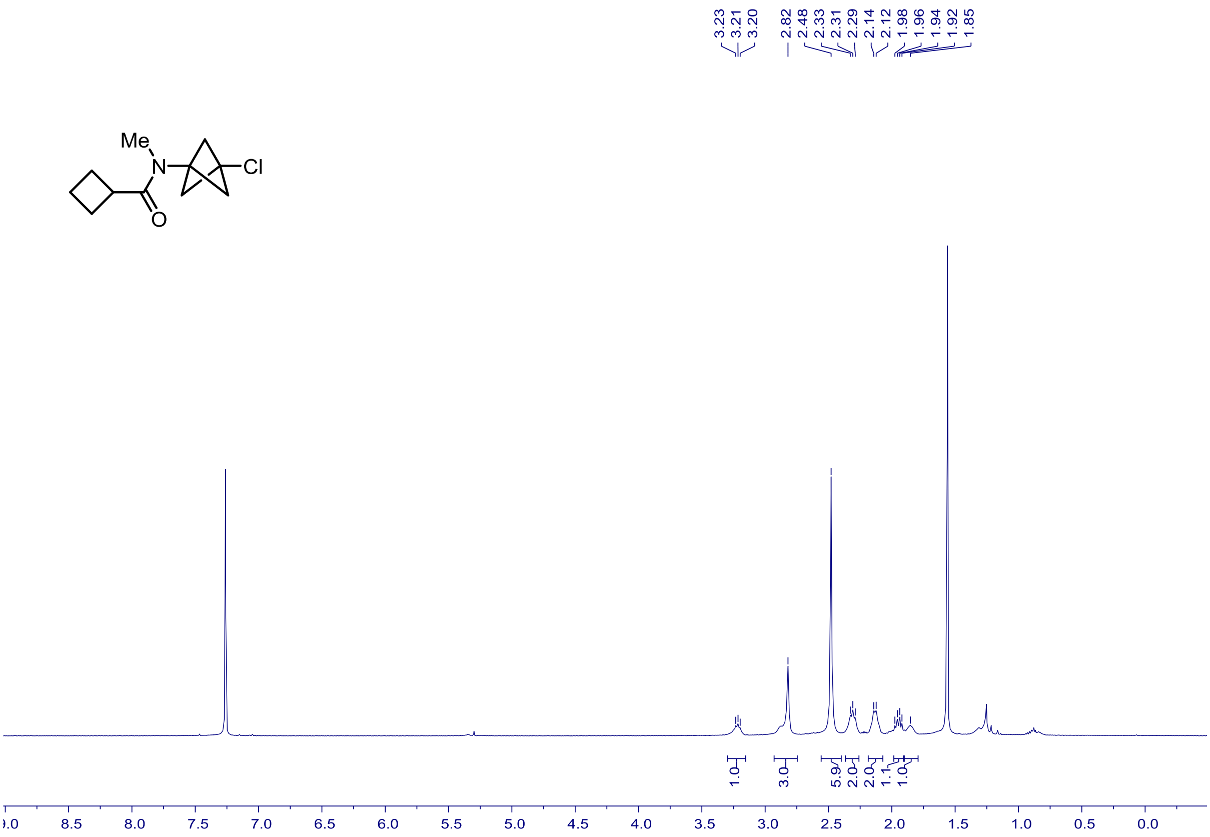

21 – ^{13}C NMR (126 MHz, $CDCl_3$)

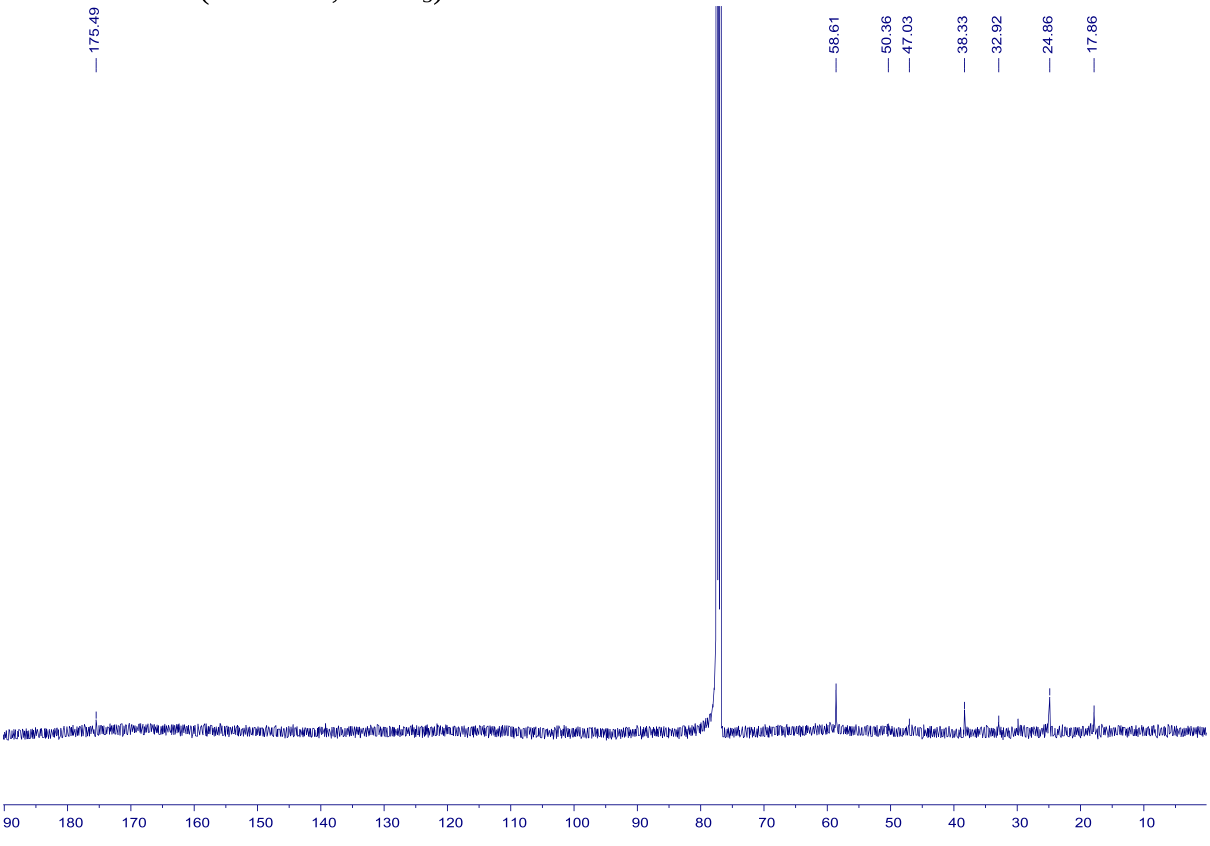

22 – ^{1}H NMR (500 MHz, $CDCl_3$)

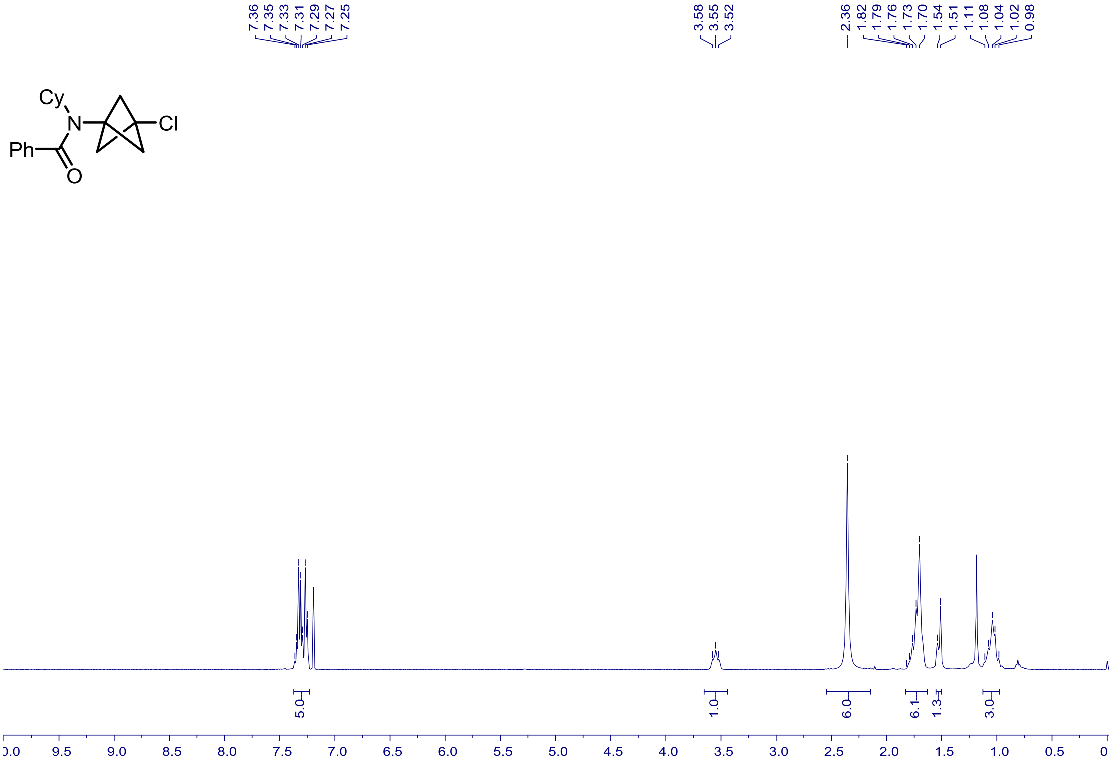

22 – ^{13}C NMR (126MHz, $CDCl_3$)

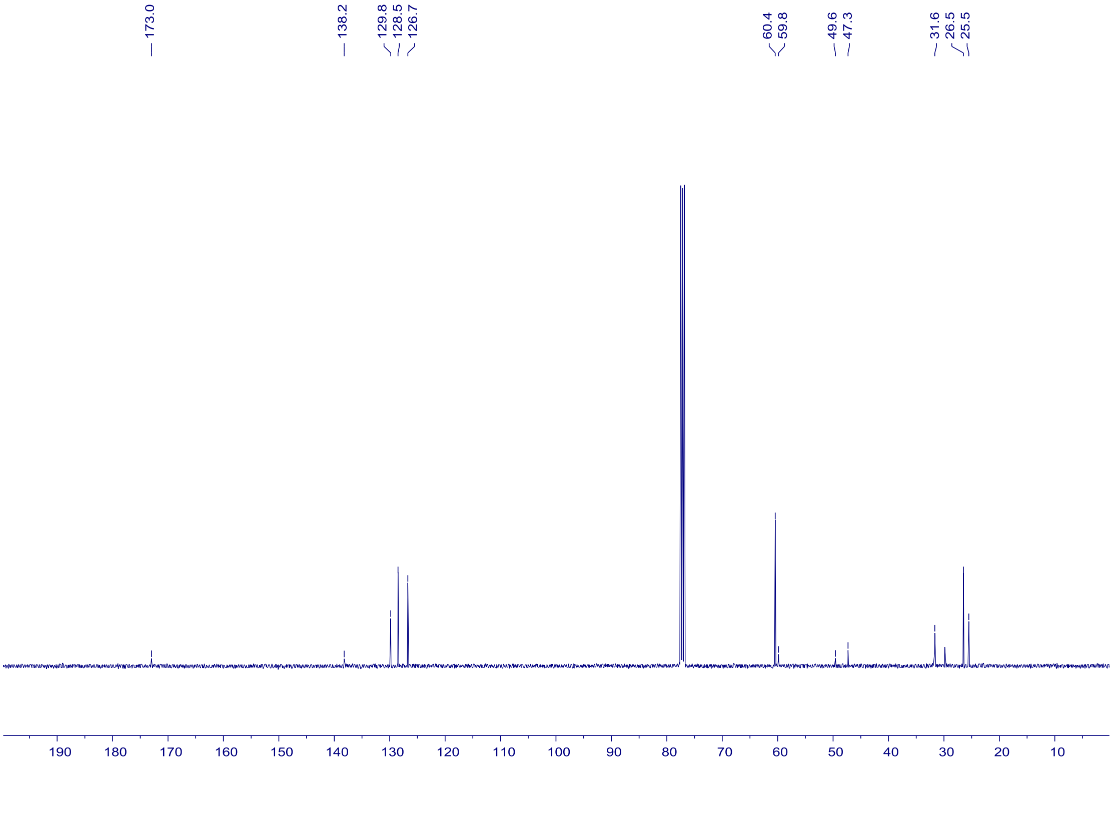

22 – HSQC (500 MHz, $CDCl_3$)

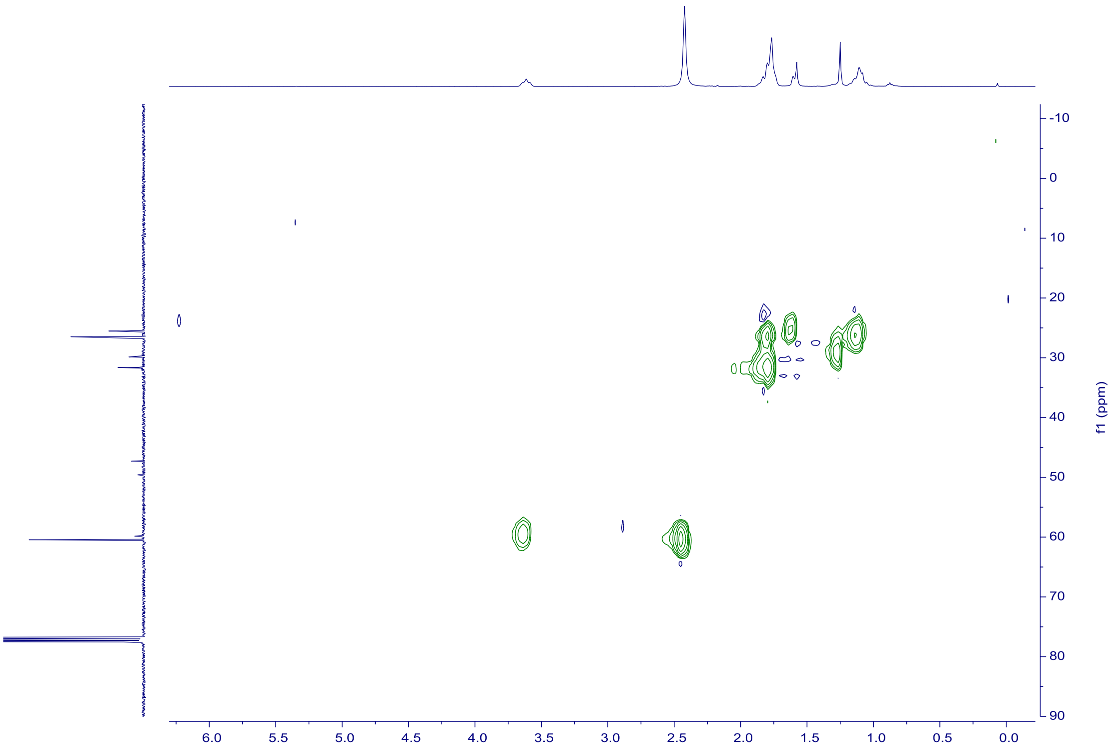

23 – ^{1}H NMR (500 MHz, $CDCl_3$)

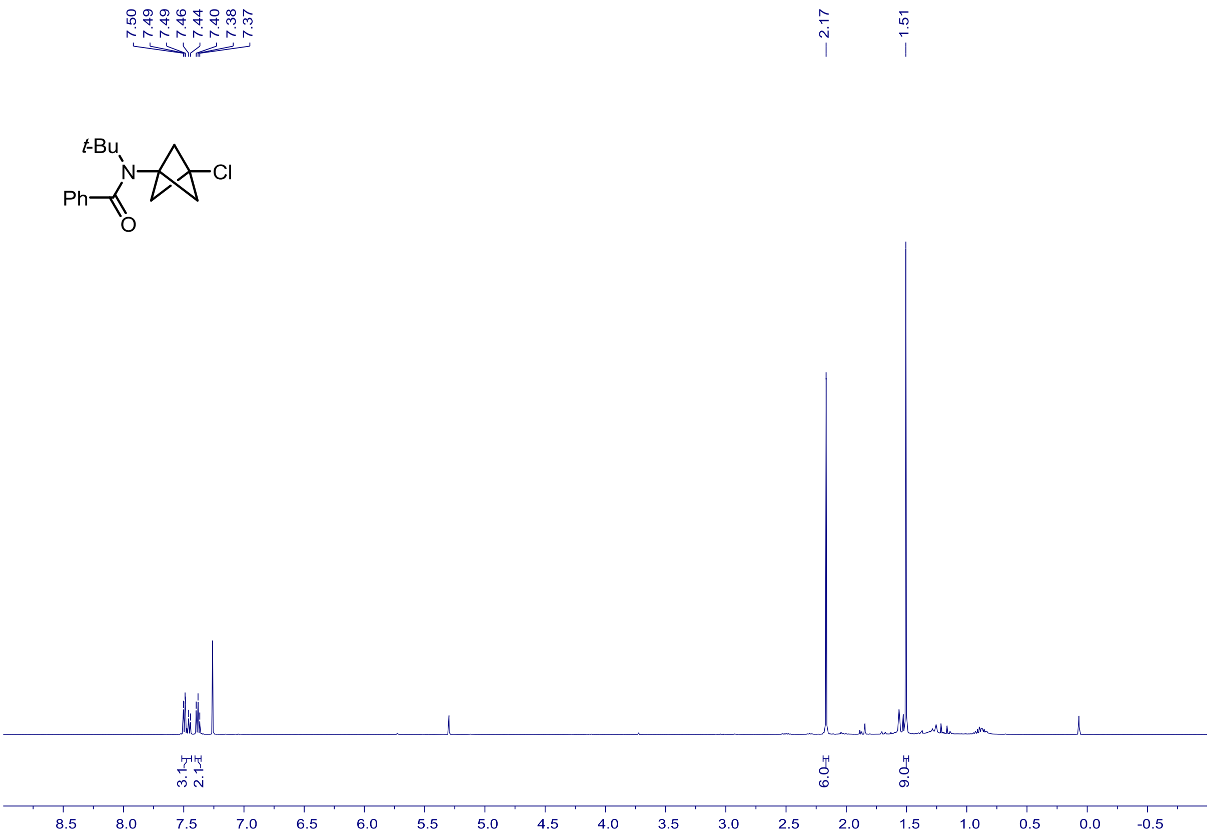

23 – ^{13}C NMR (126 MHz, $CDCl_3$)

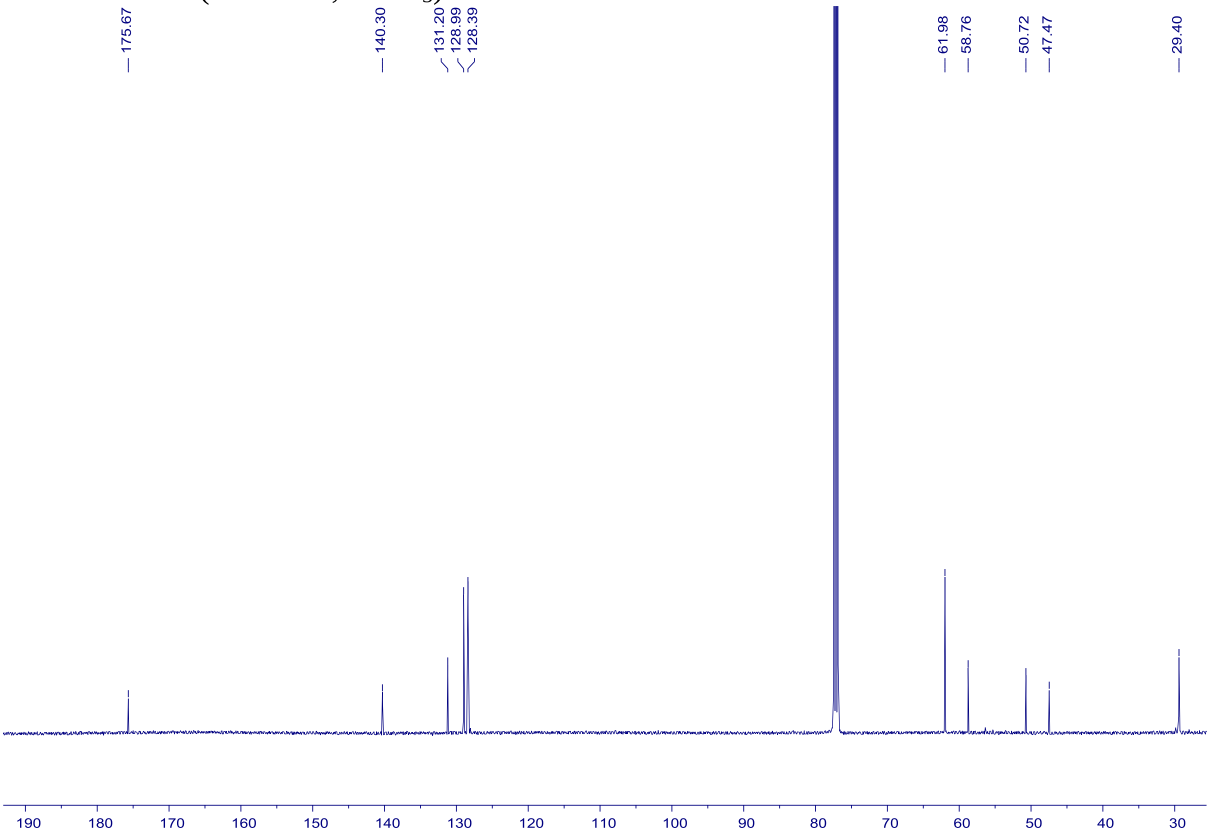

25 – ^{1}H NMR (500 MHz, $CDCl_3$)

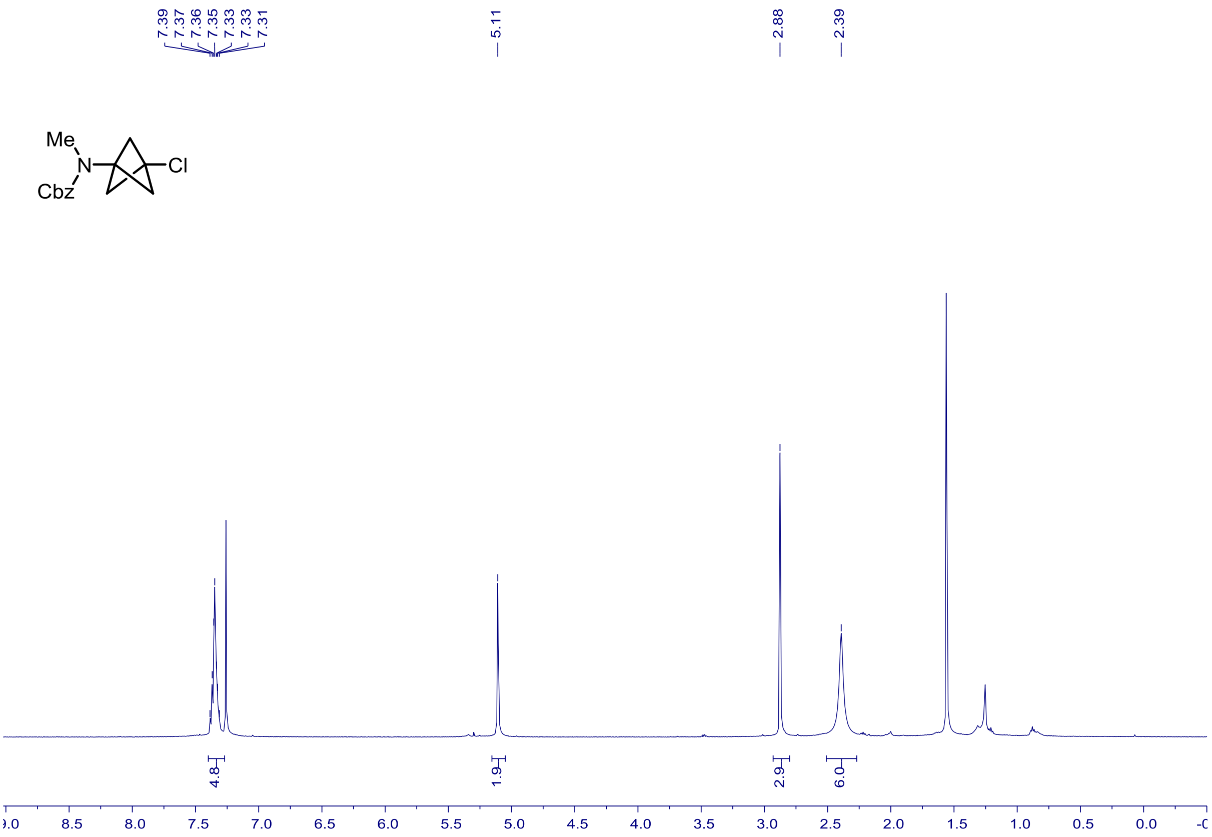

25 – ^{13}C NMR (126 MHz, $CDCl_3$)

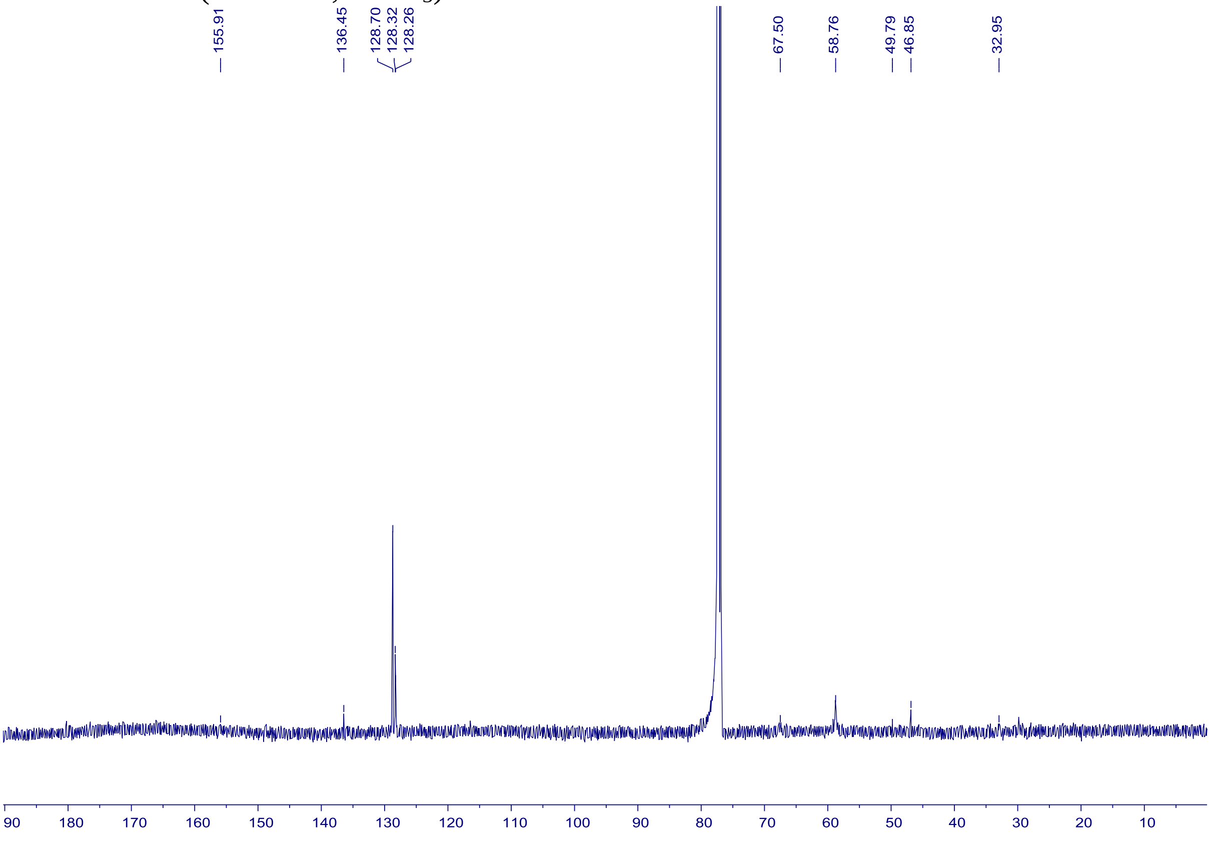

26 – ^{1}H NMR (500 MHz, $CDCl_3$)

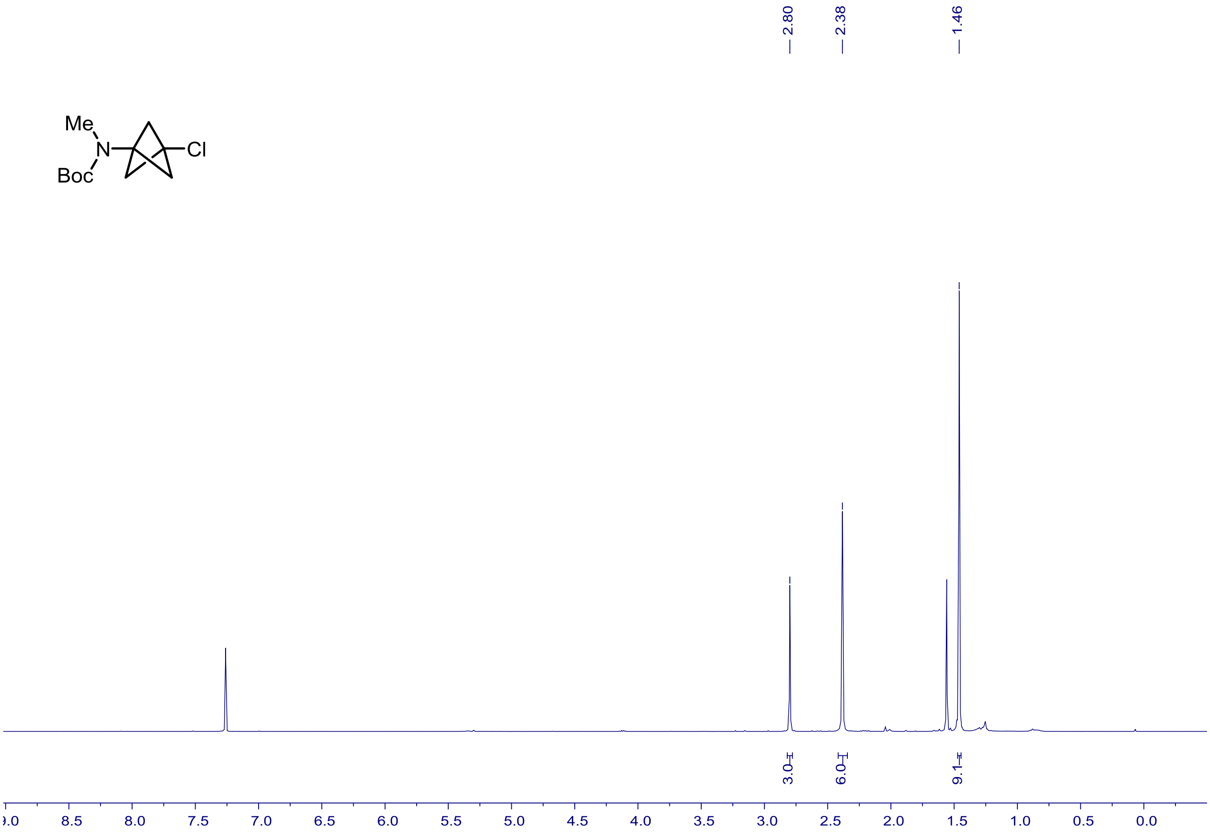

26 – ^{13}C NMR (101 MHz, $CDCl_3$)

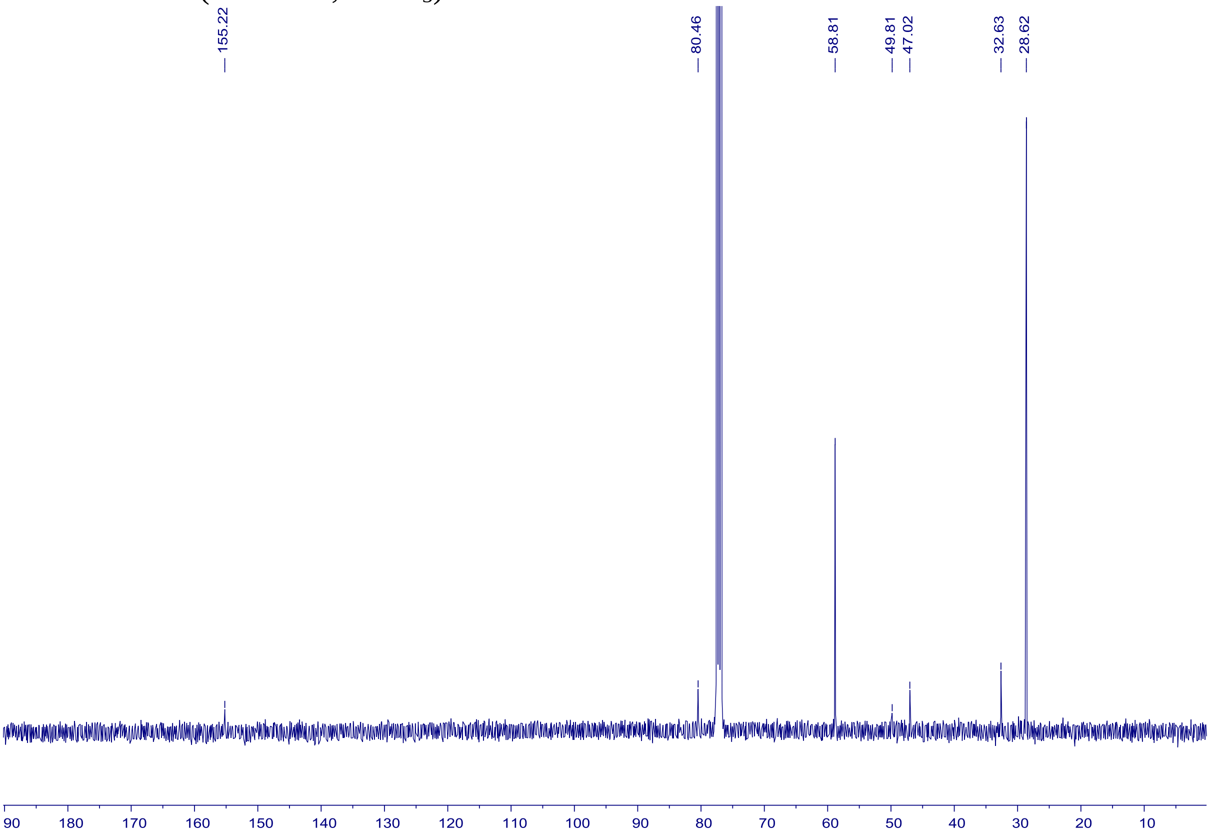

27 – ^{1}H NMR (500 MHz, $CDCl_3$)

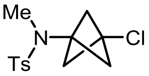

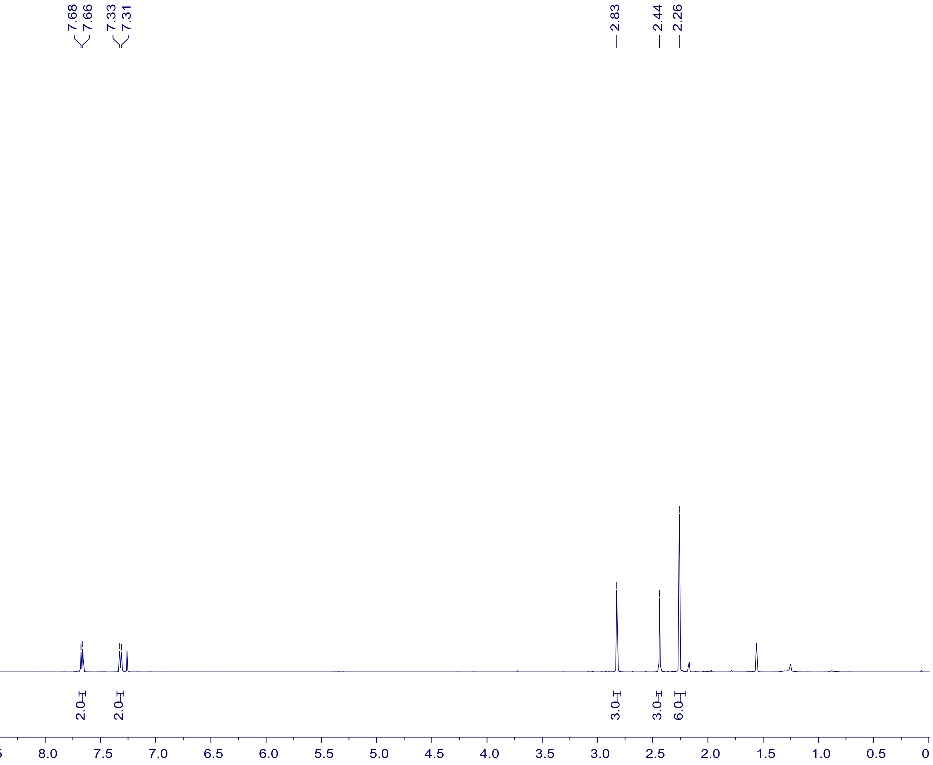

27 – ^{13}C NMR (126MHz, $CDCl_3$)

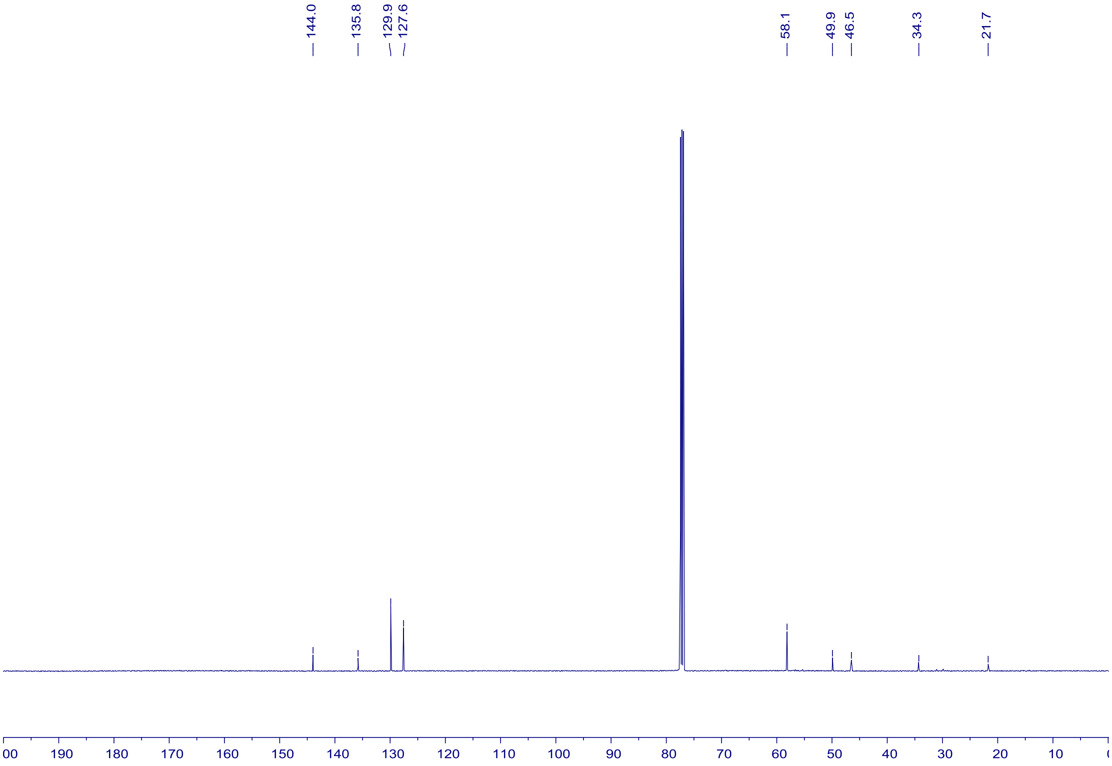

27 – HSQC (500 MHz, $CDCl_3$)

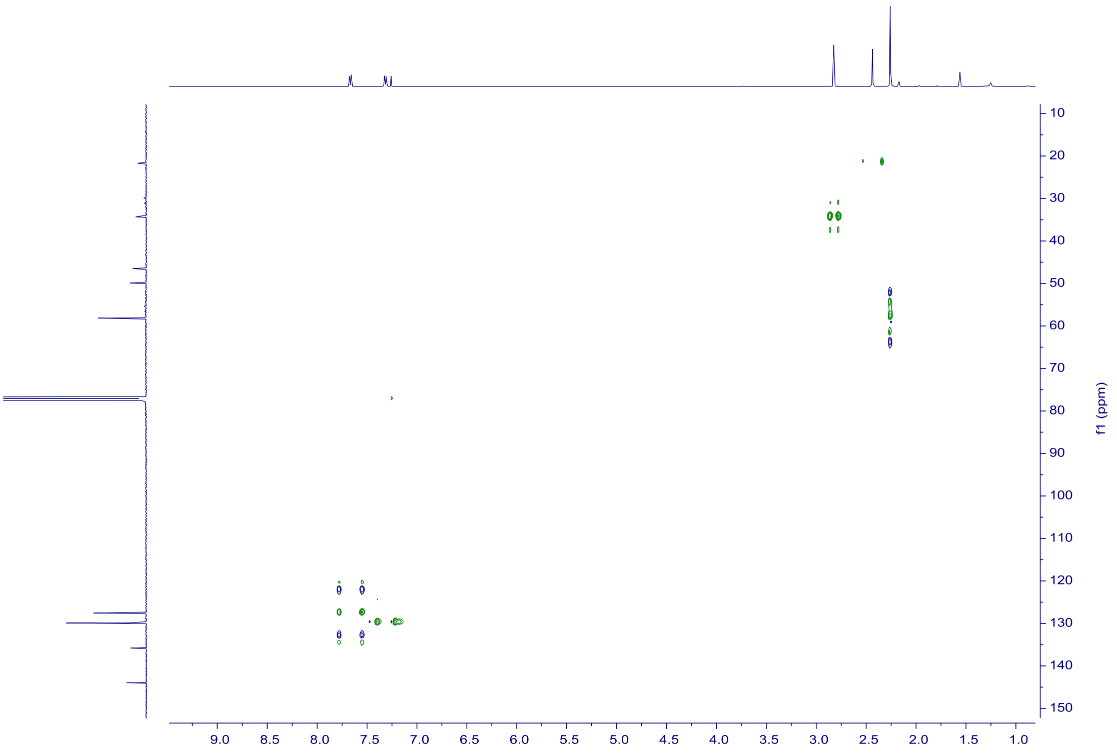

27 – HMBC (500 MHz, $CDCl_3$)

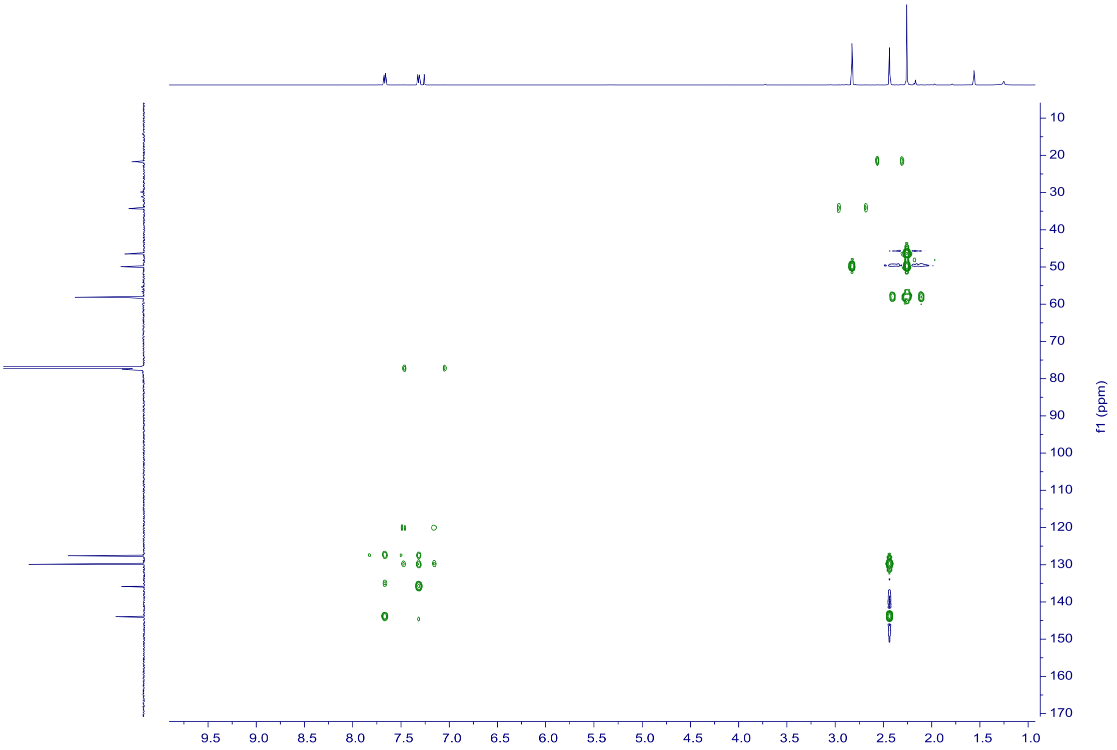

28 – ^{1}H NMR (400 MHz, $CDCl_3$)

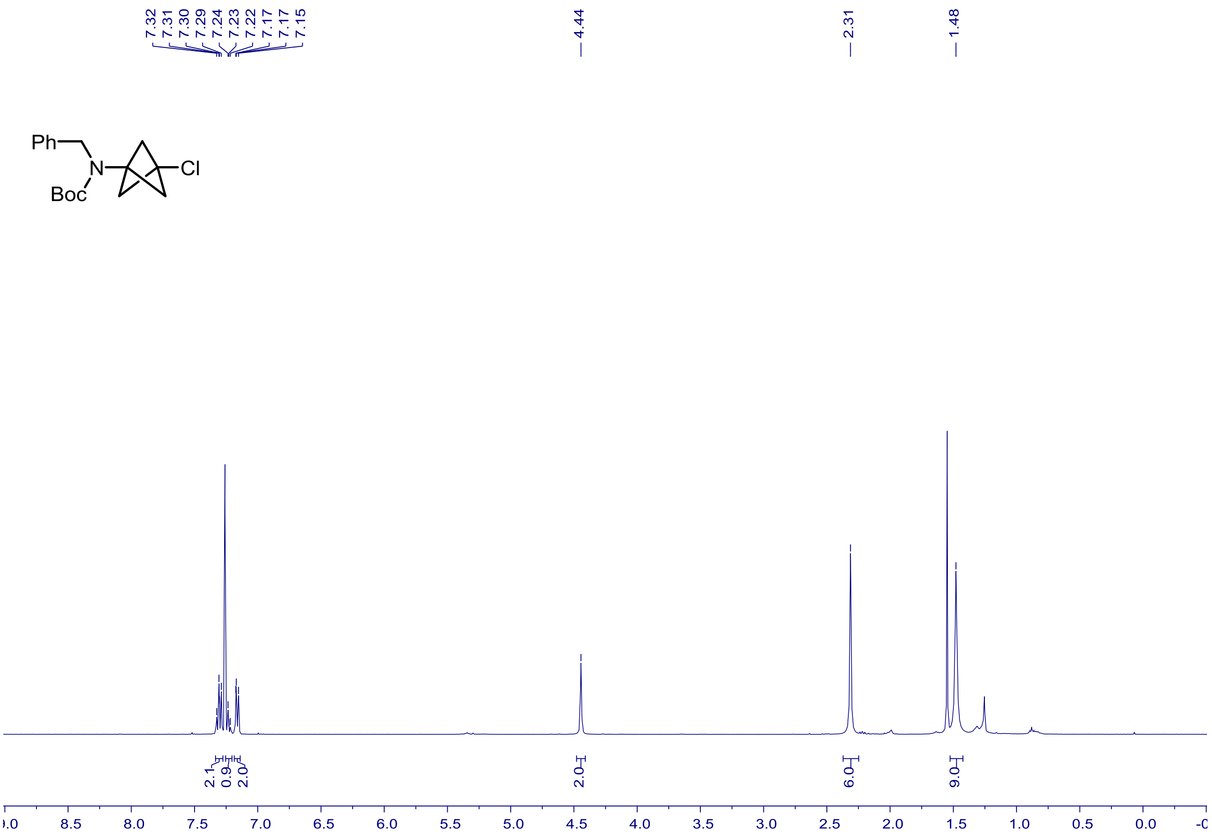

28 – ^{13}C NMR (101 MHz, $CDCl_3$)

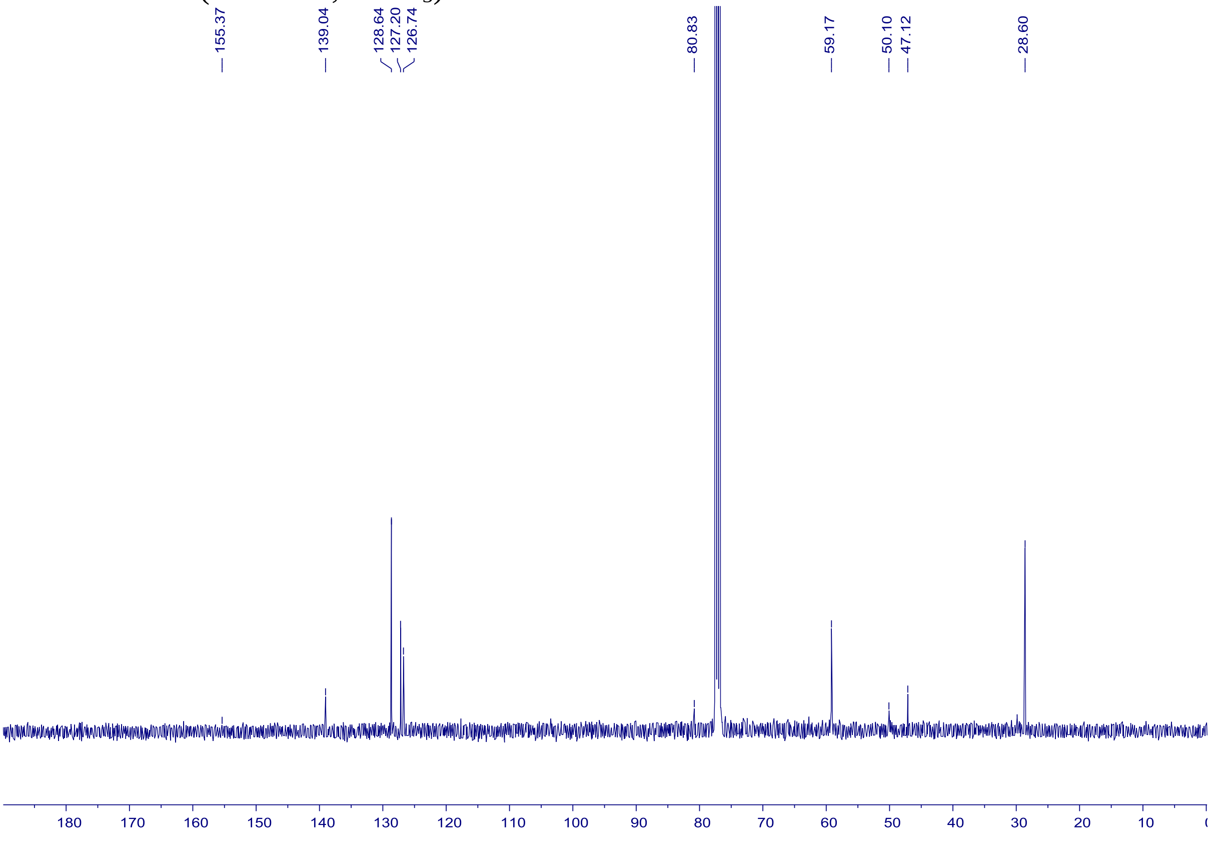

29 – ^{1}H NMR (400 MHz, $CDCl_3$)

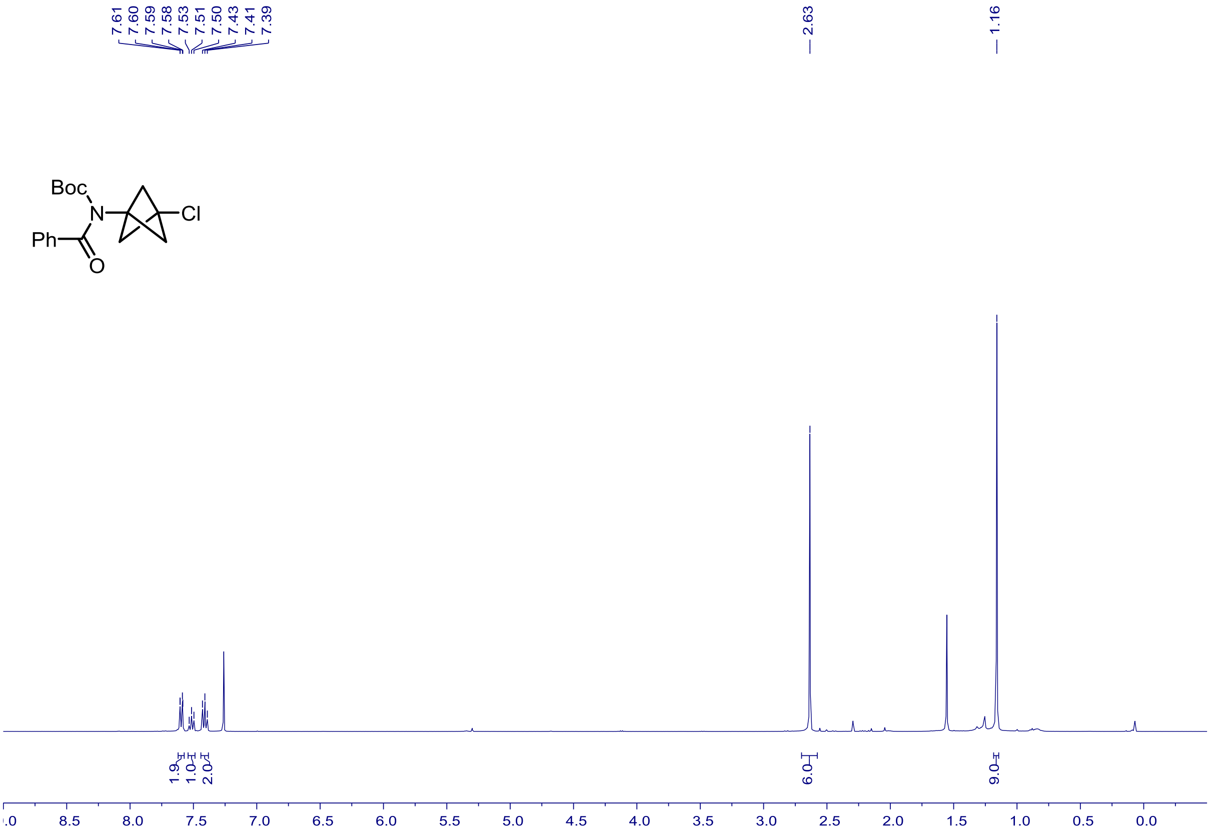

29 – ^{13}C NMR (101 MHz, $CDCl_3$)

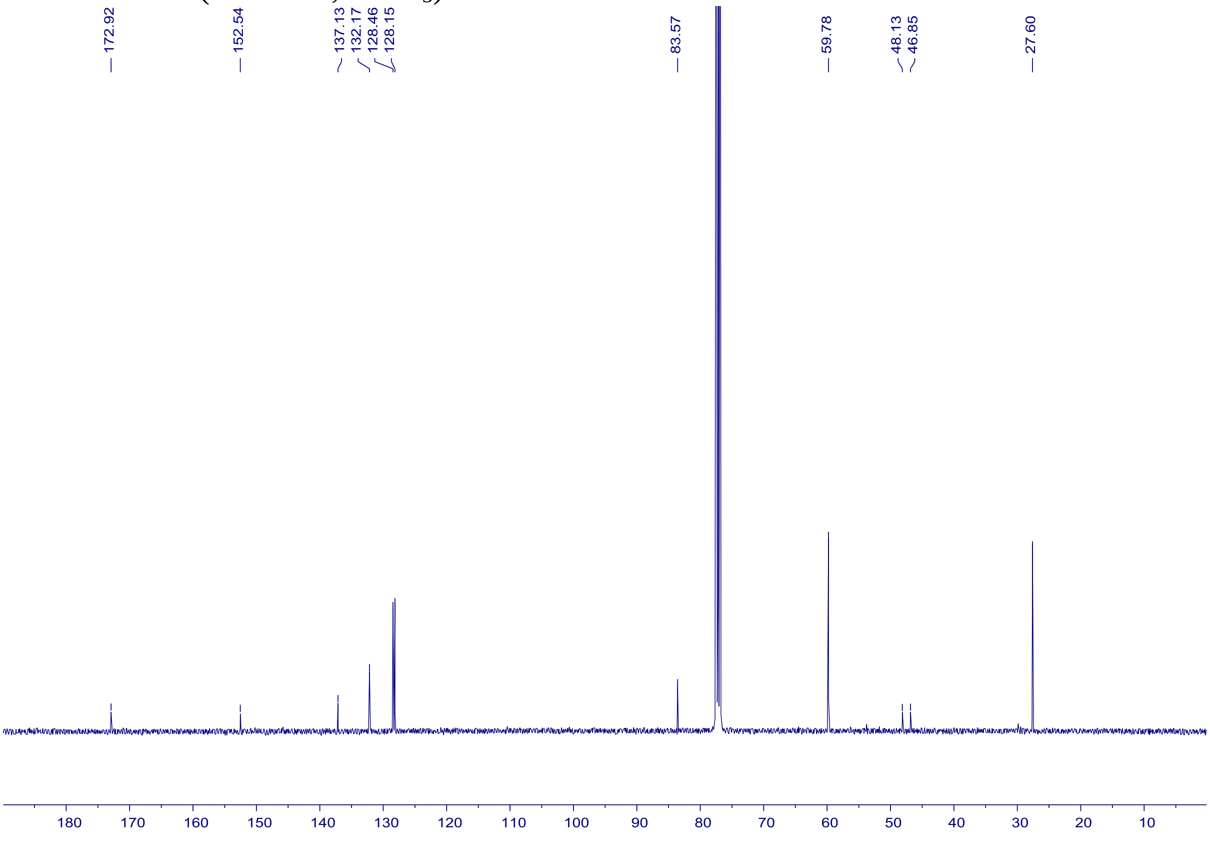

30 – ^{1}H NMR (400 MHz, $CDCl_3$)

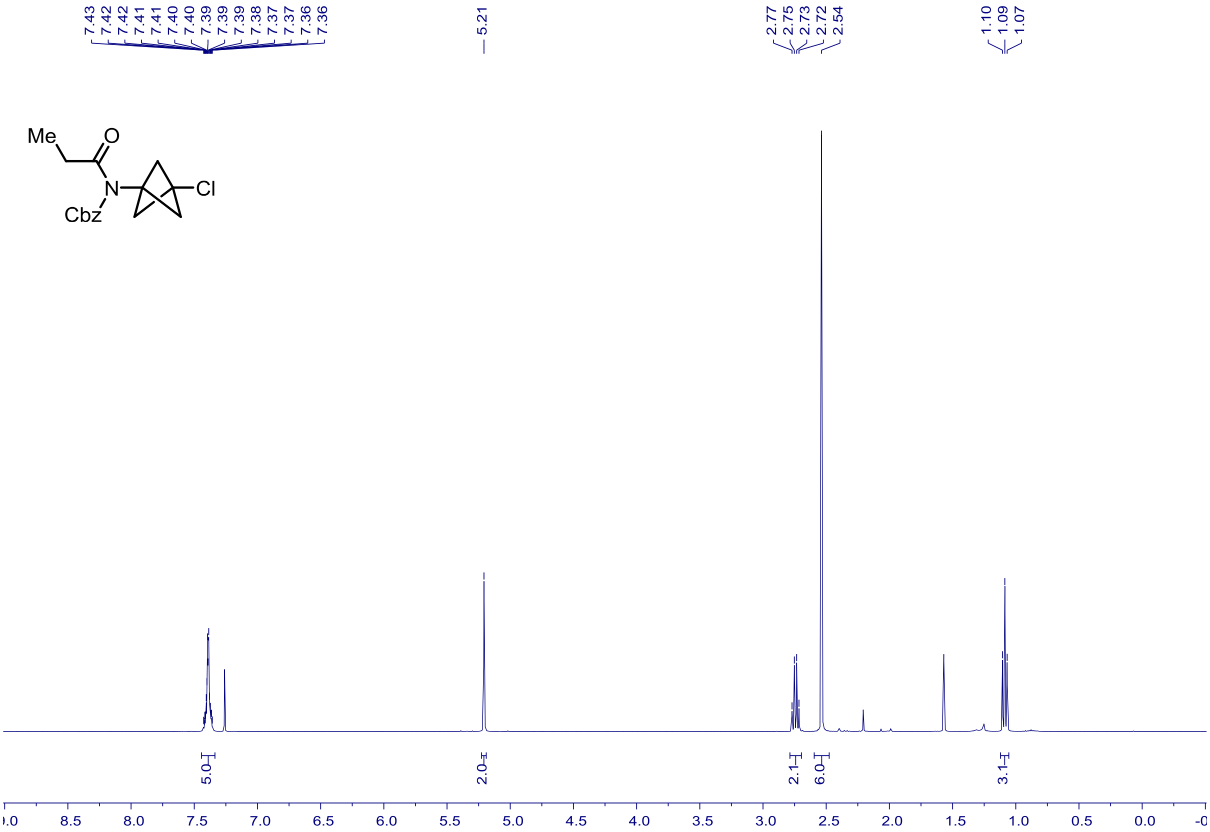

30 – ^{13}C NMR (101 MHz, $CDCl_3$)

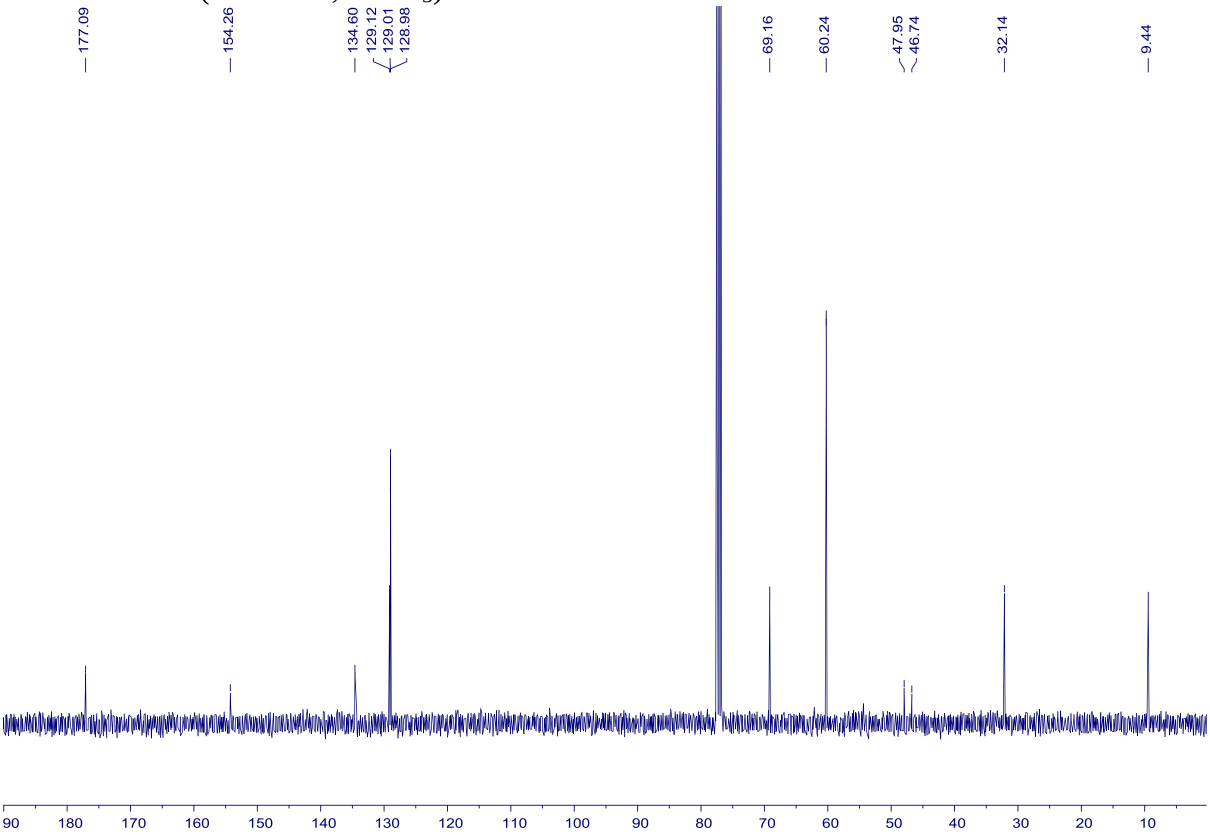

31 – ^{1}H NMR (400 MHz, $CDCl_3$)

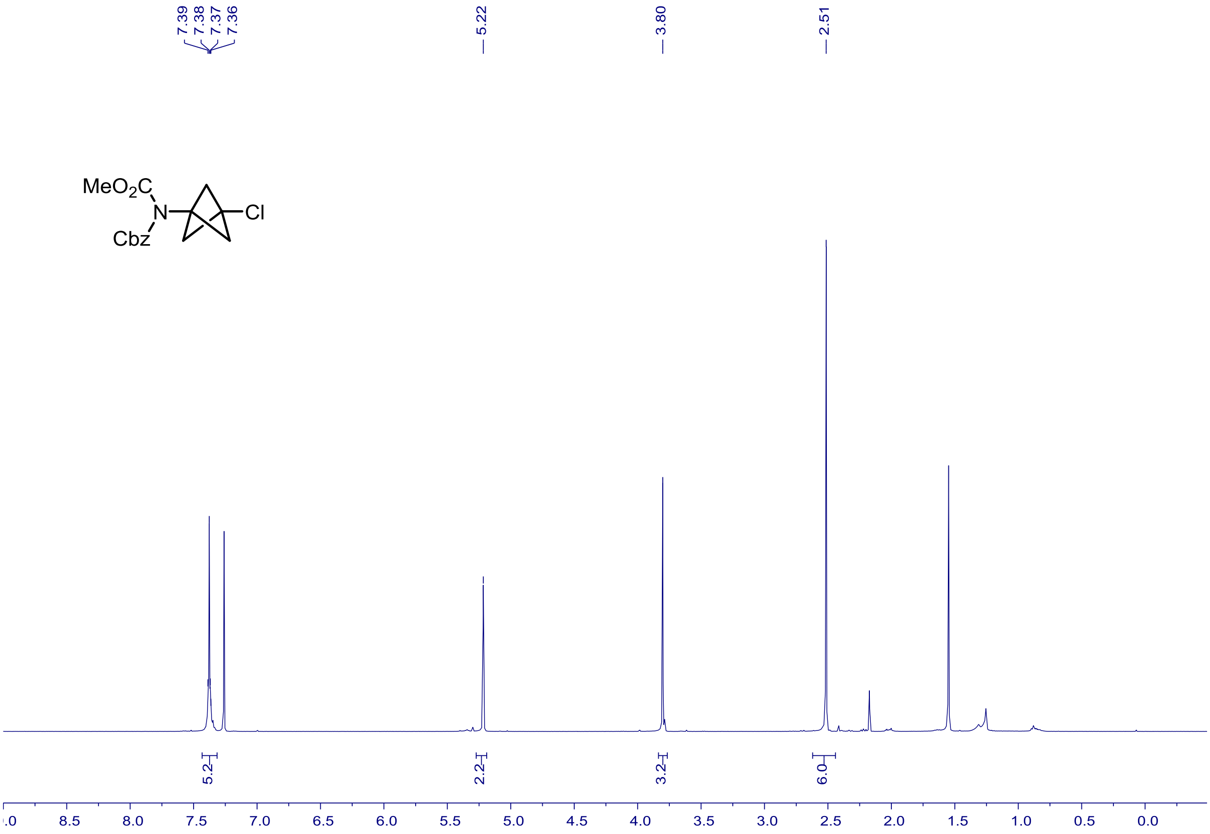

31 – ^{13}C NMR (101 MHz, $CDCl_3$)

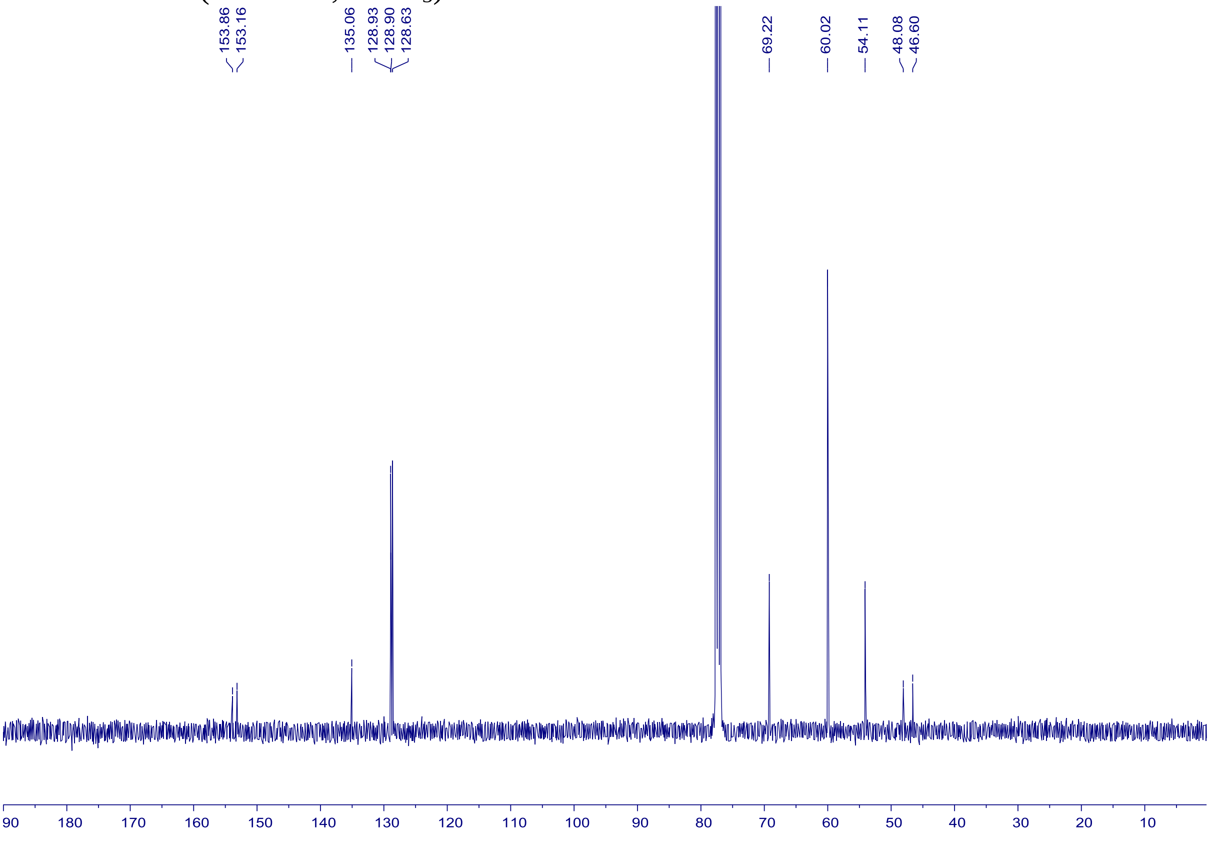

32 – ¹H NMR (400 MHz, $CDCl_3$)

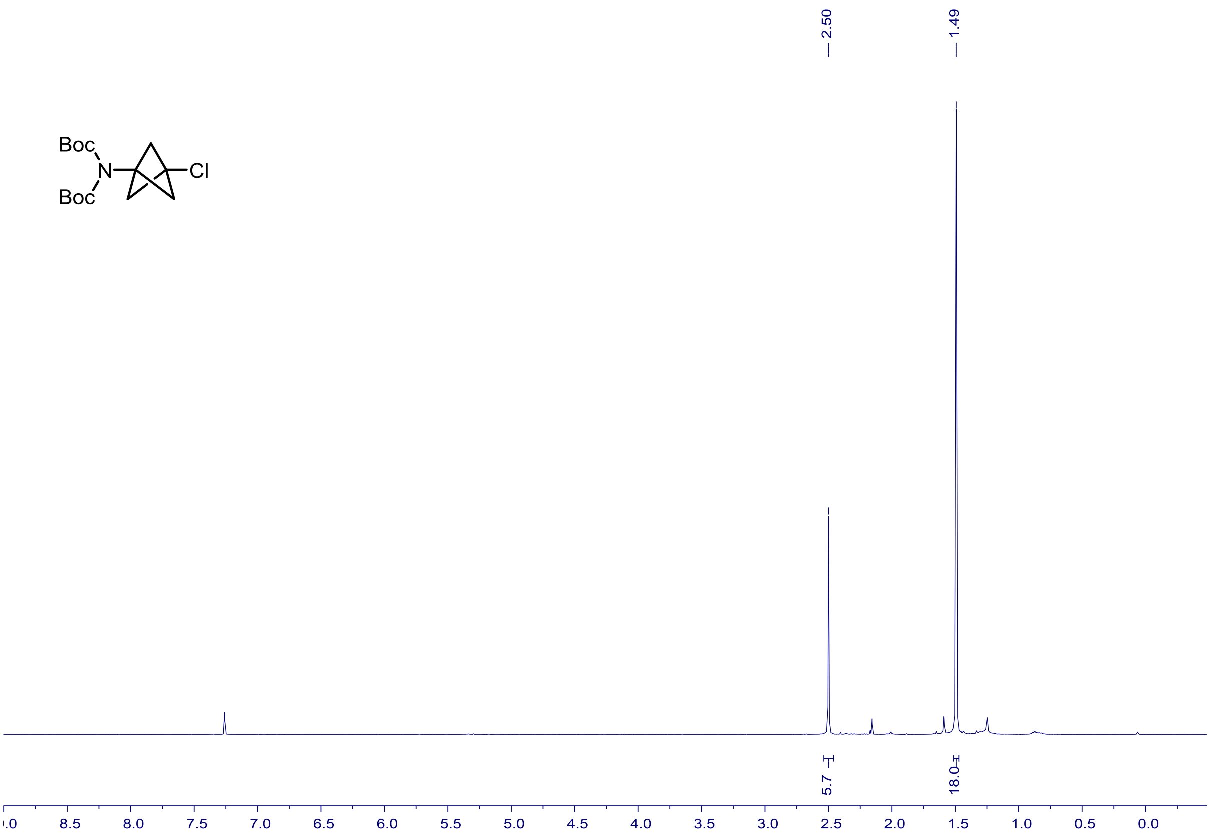

32 – ¹³C NMR (101 MHz, $CDCl_3$)

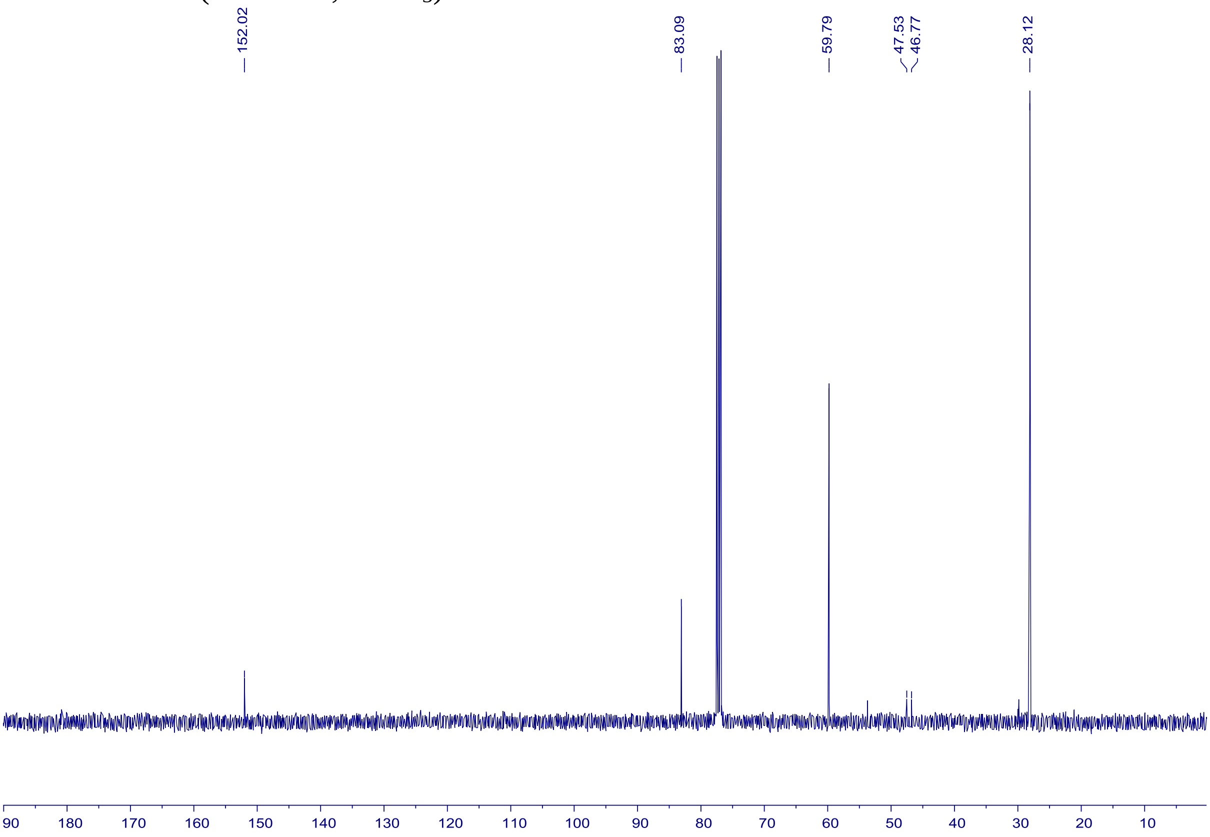

35 – ^{1}H NMR (500 MHz, $CDCl_3$)

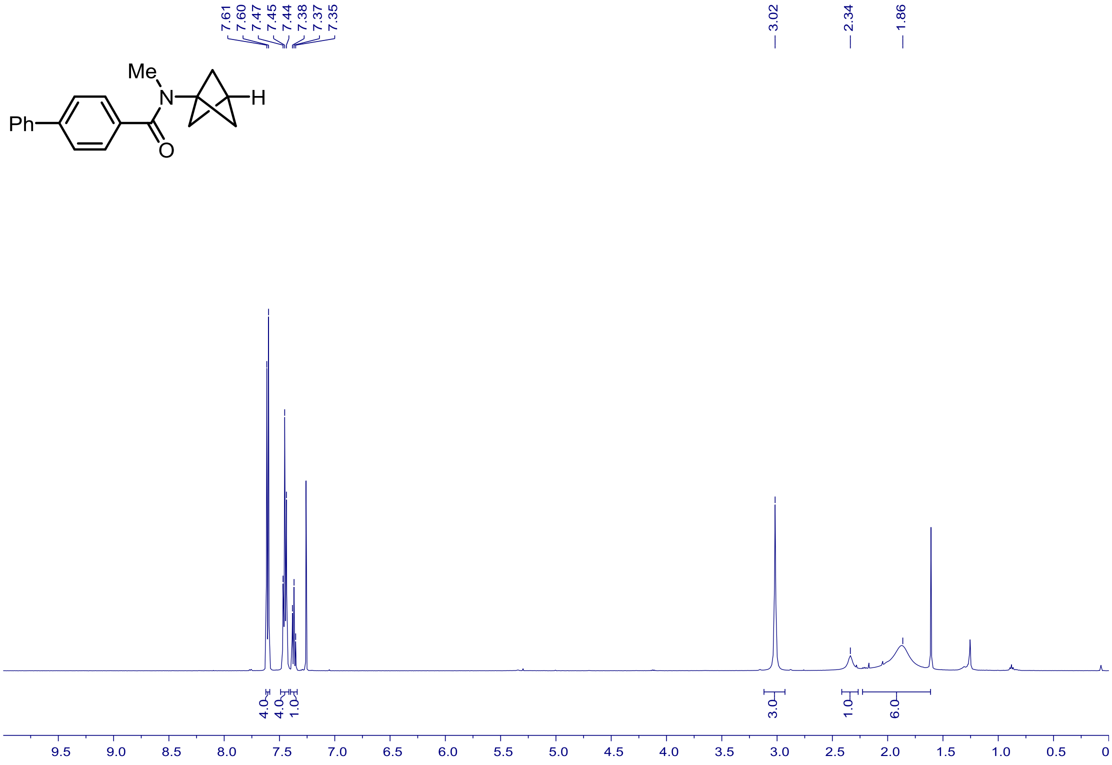

35 – ^{13}C NMR (126MHz, $CDCl_3$)

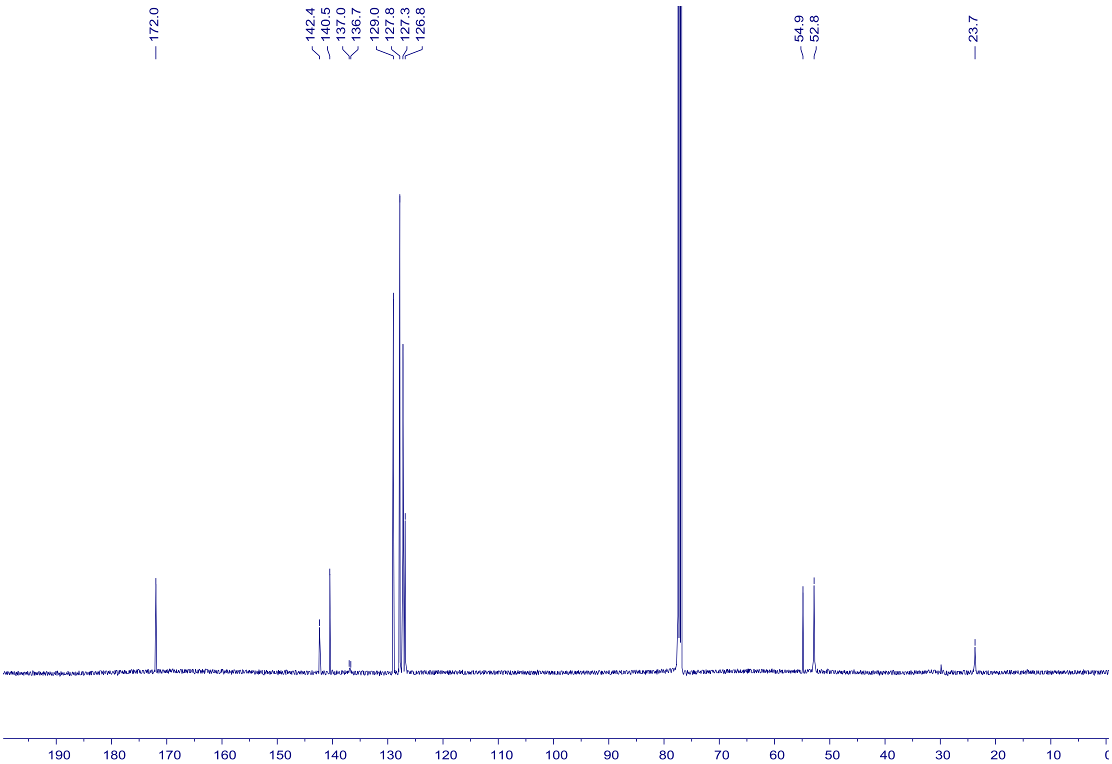

35 – HSQC (500 MHz, $CDCl_3$)

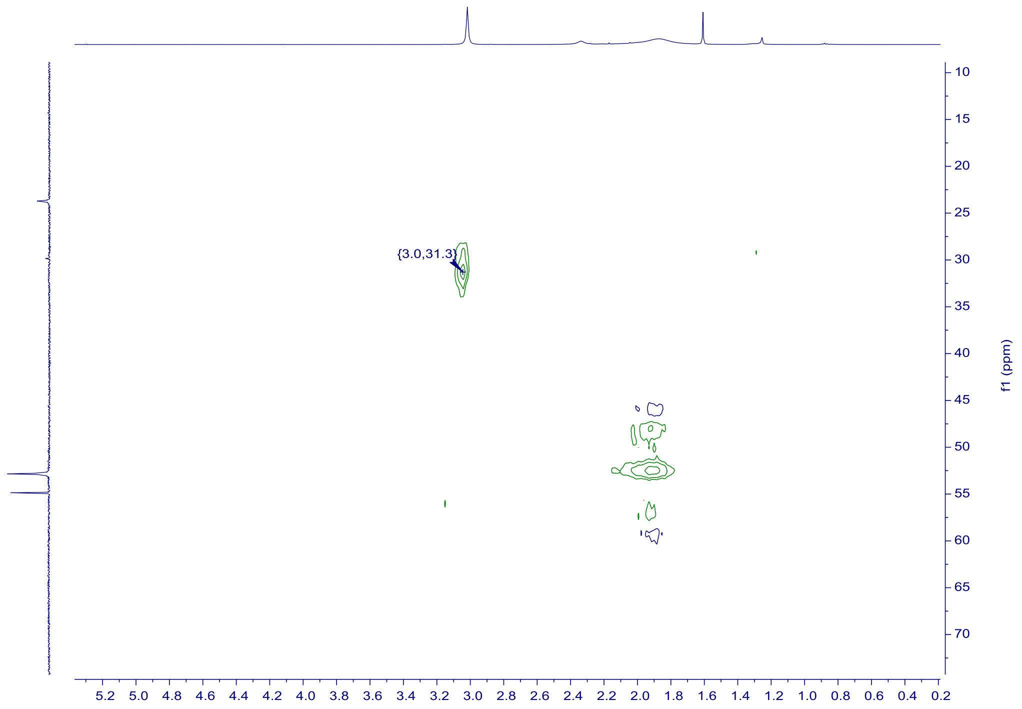

35 – HMBC (500 MHz, $CDCl_3$)

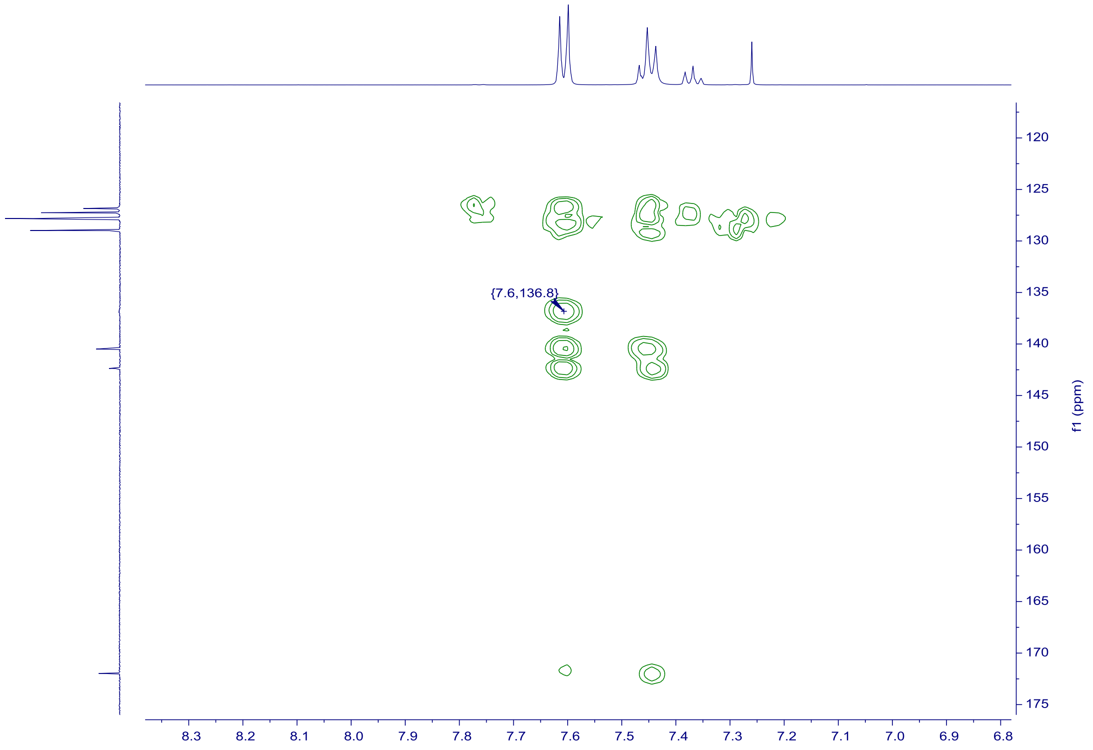

36 – ^{1}H NMR (500 MHz, $CDCl_3$)

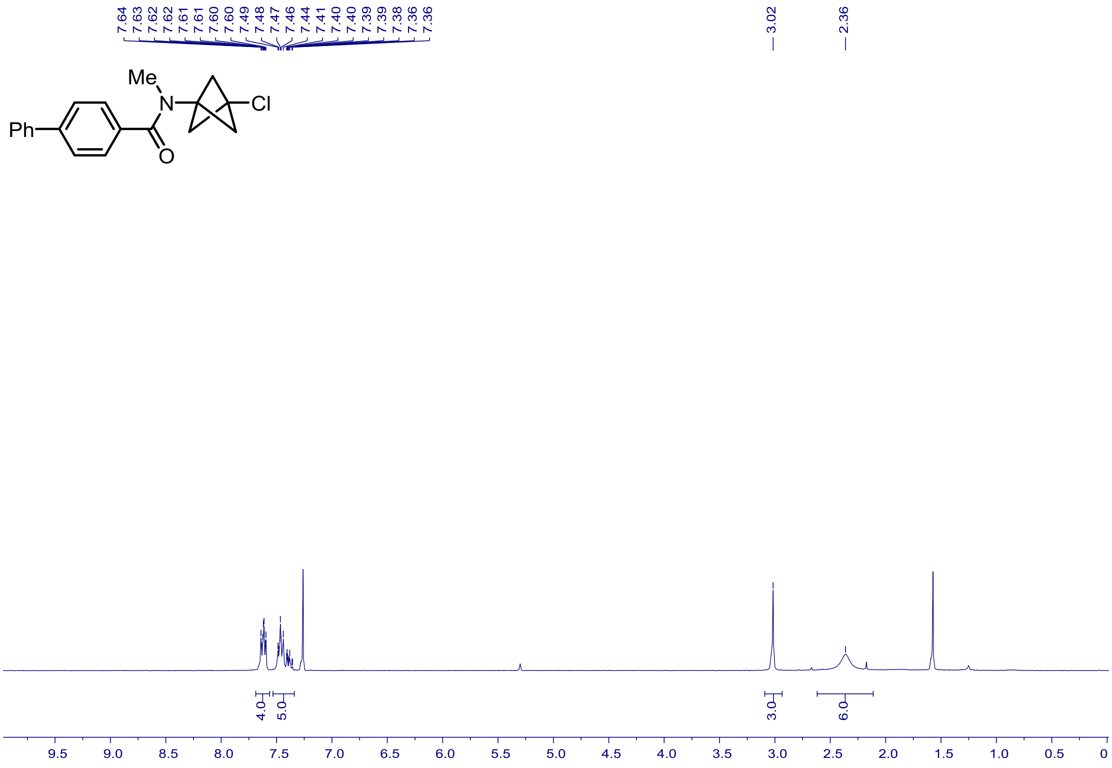

36 – ^{1}H NMR (126 MHz, $CDCl_3$)

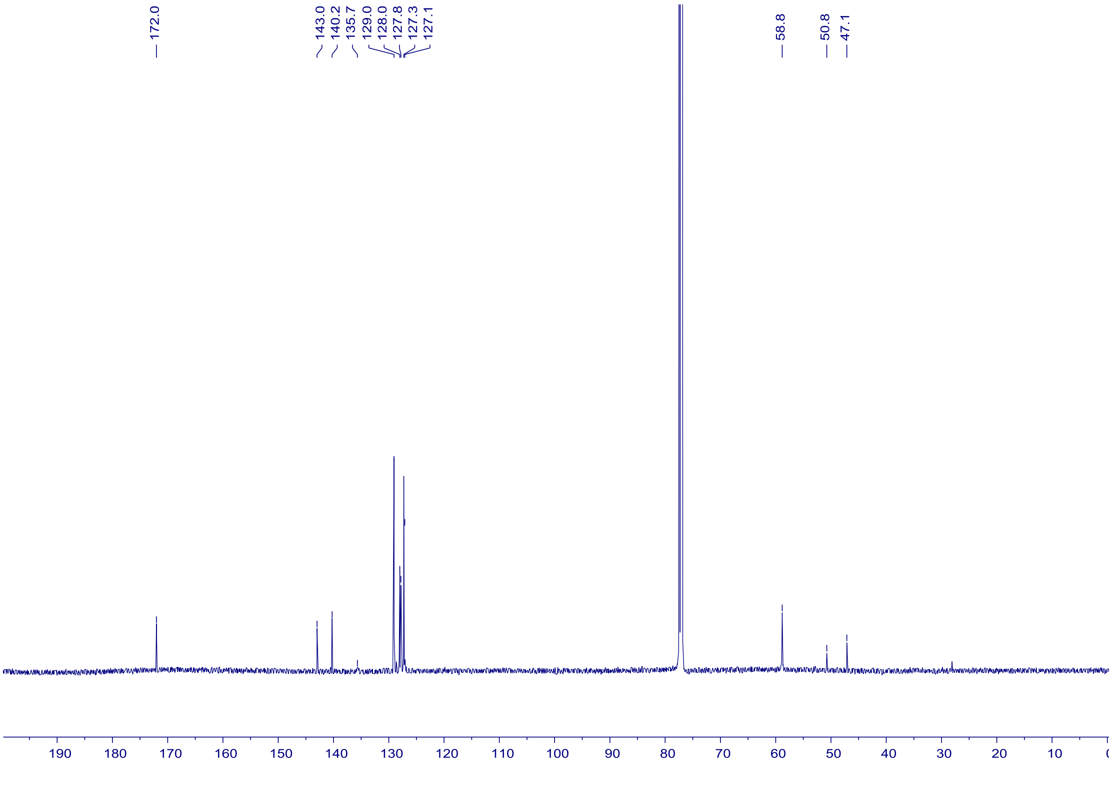

36 – HSQC (500 MHz, $CDCl_3$)

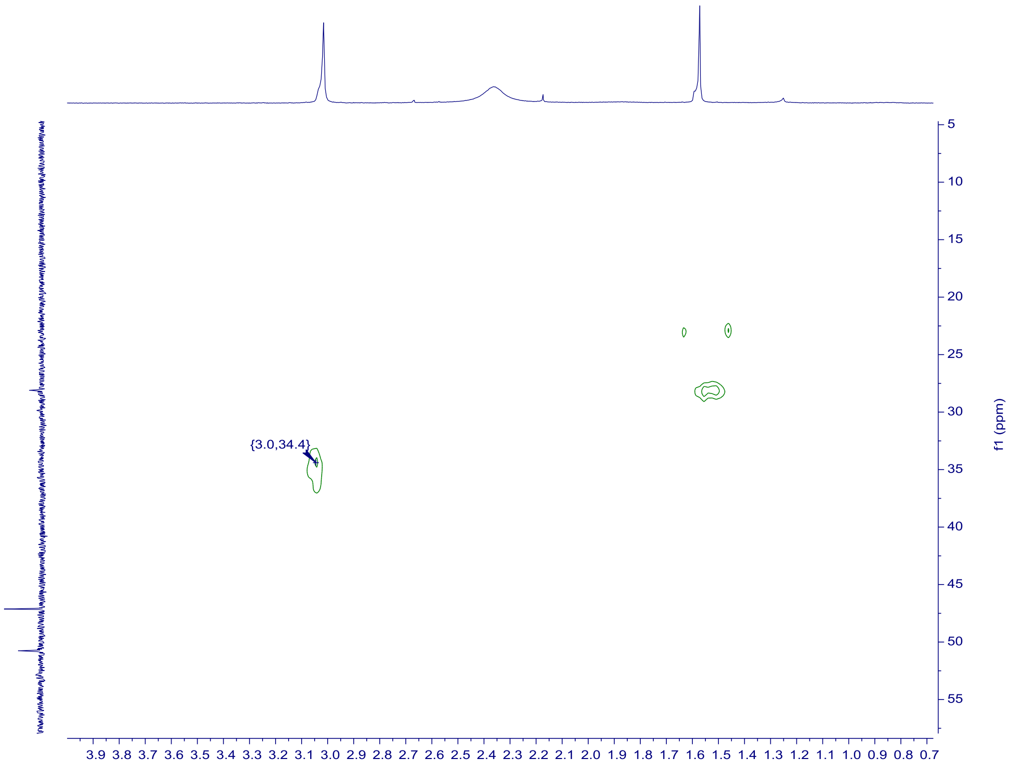

36 – HMBC (500 MHz, $CDCl_3$)

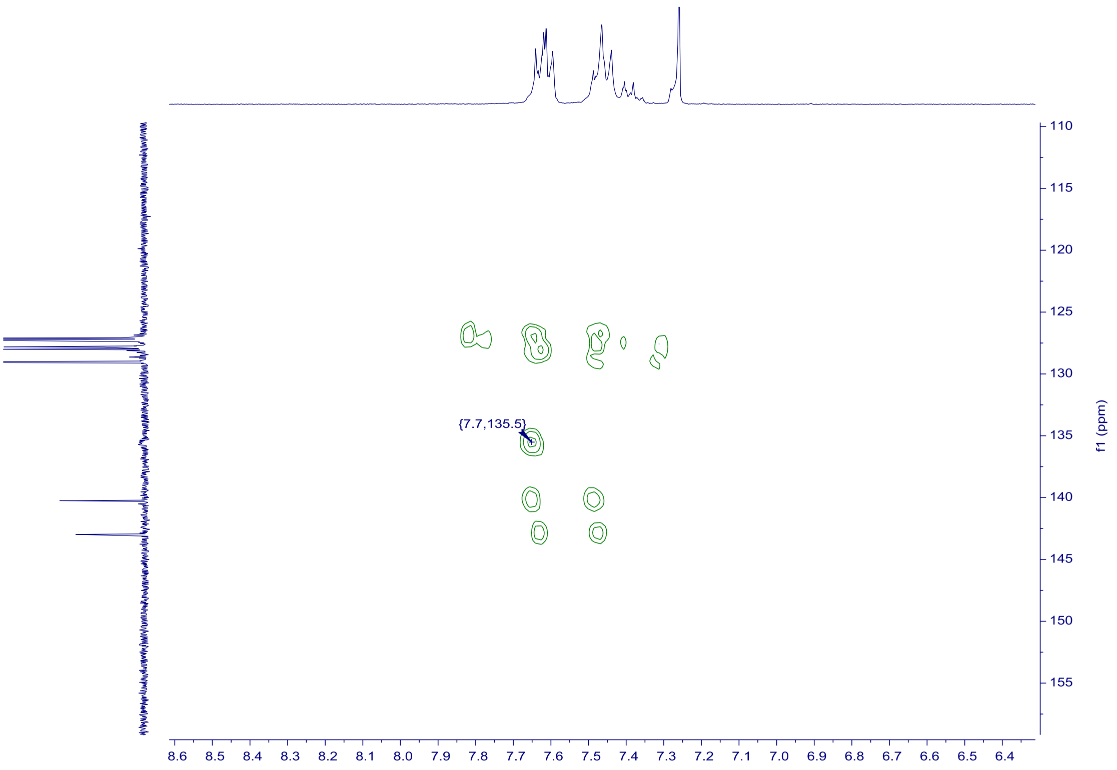

37 – ^{1}H NMR (500 MHz, CDCl$_3$)

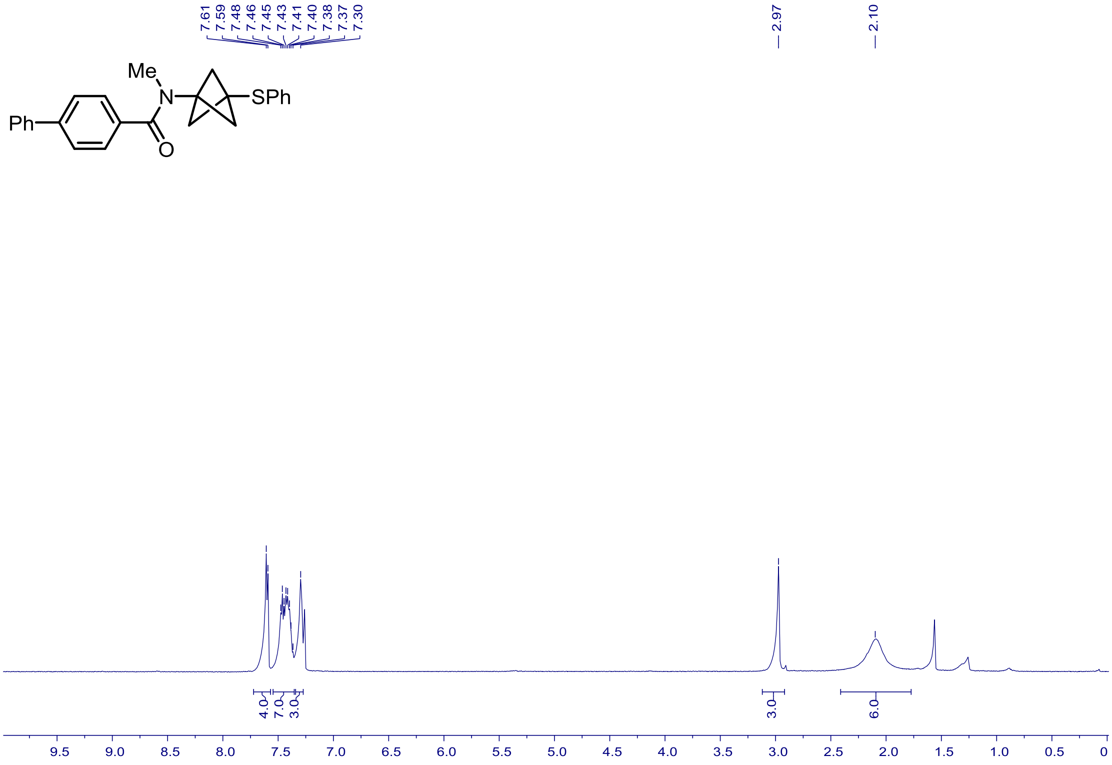

37 – ^{13}C NMR (126MHz, CDCl$_3$)

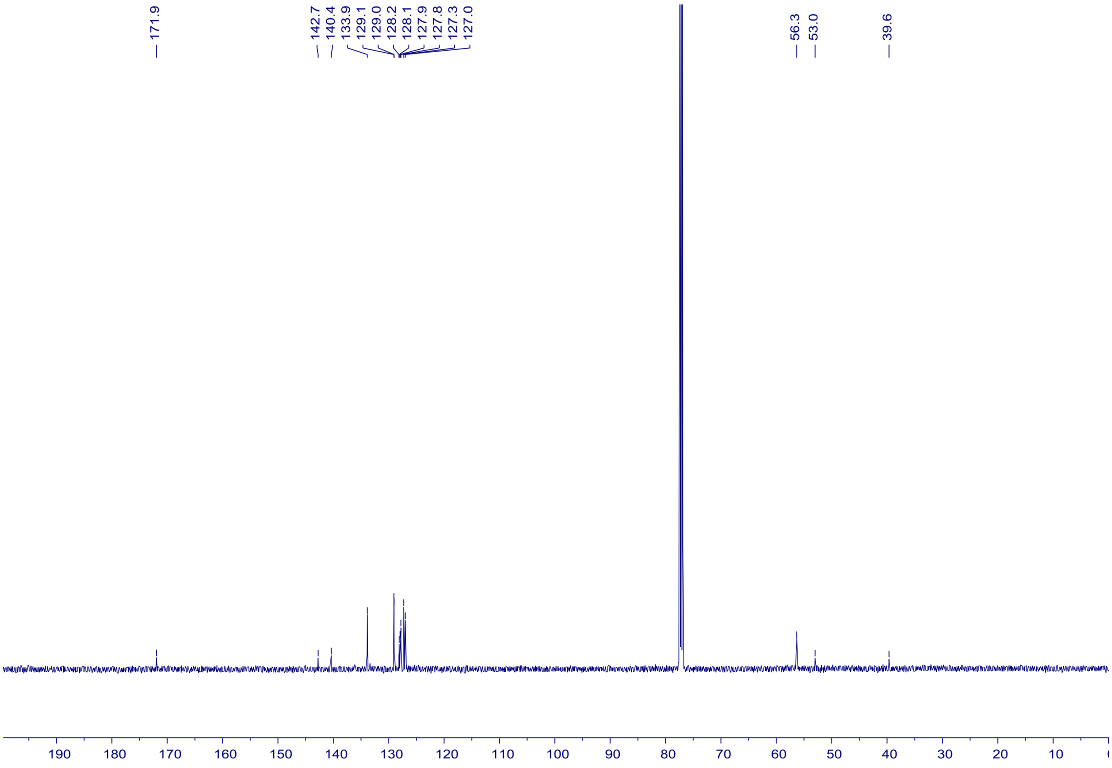

37– HSQC (500 MHz, $CDCl_3$)

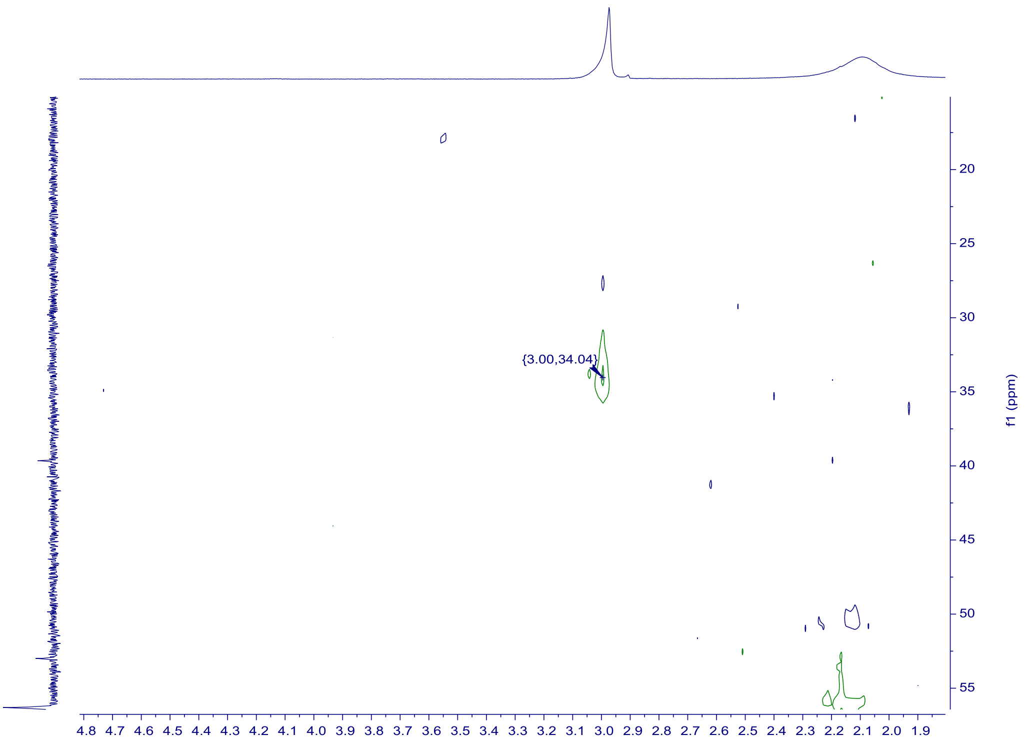

37 – HMBC (500 MHz, $CDCl_3$)

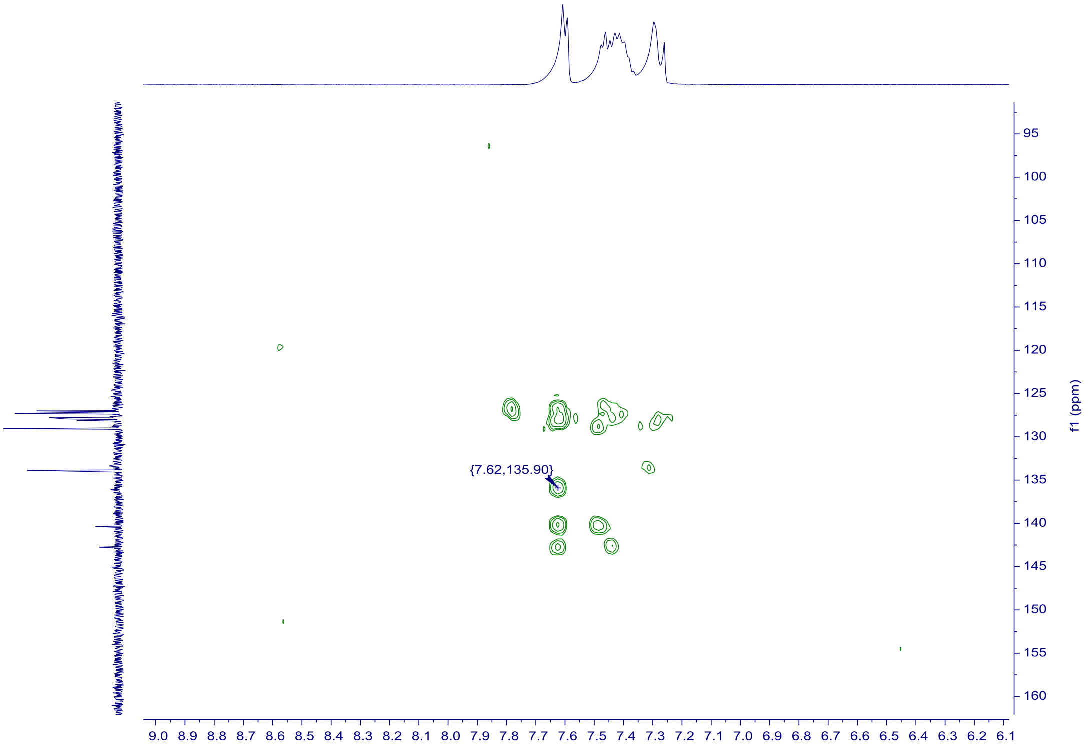

38 – ^{1}H NMR (500 MHz, $CDCl_3$)

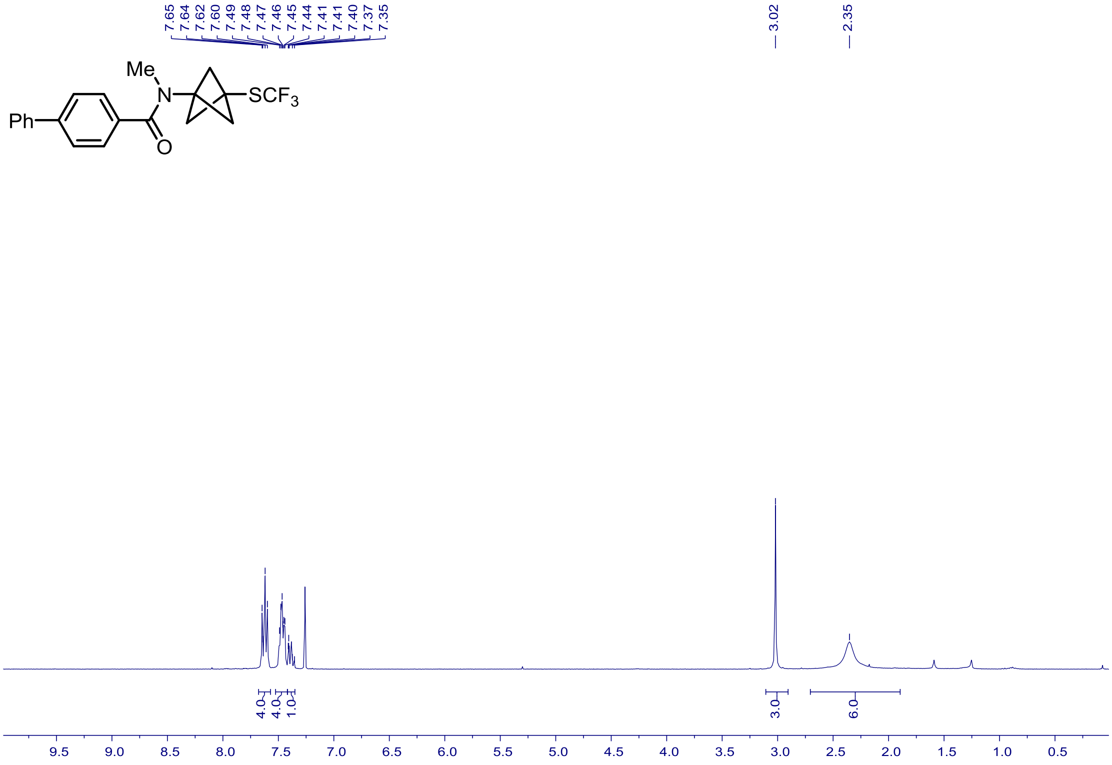

38– ^{13}C NMR (126MHz, $CDCl_3$)

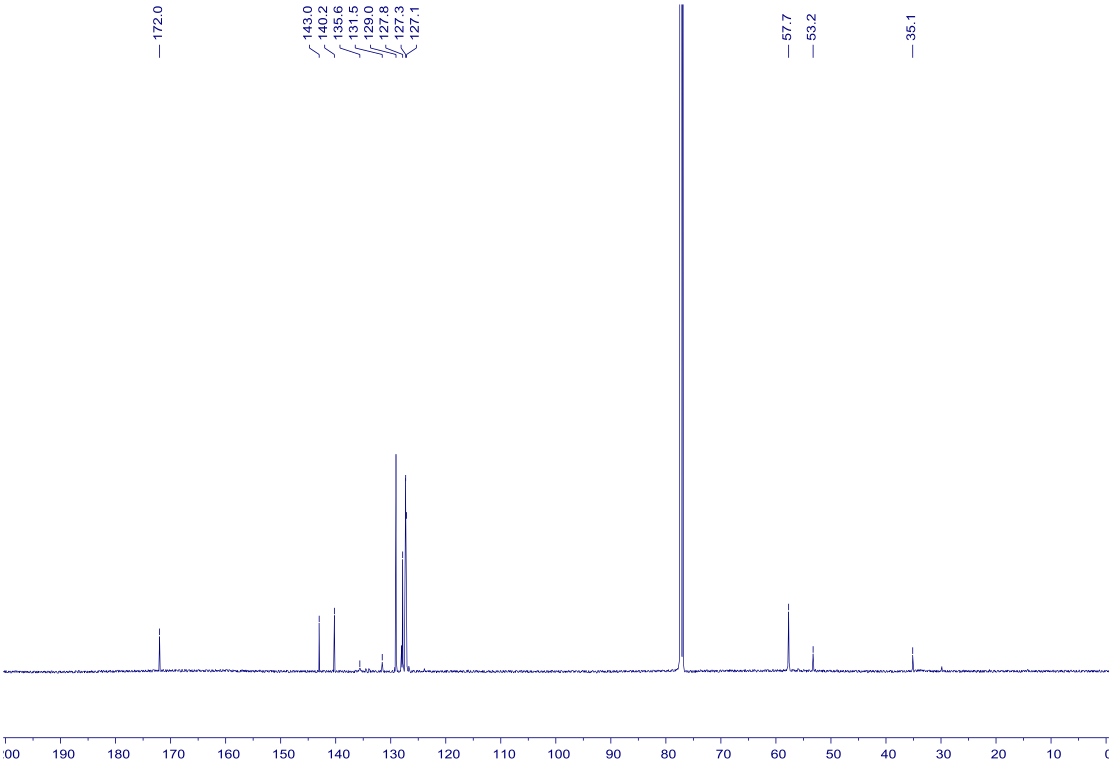

38– HSQC (500 MHz, $CDCl_3$)

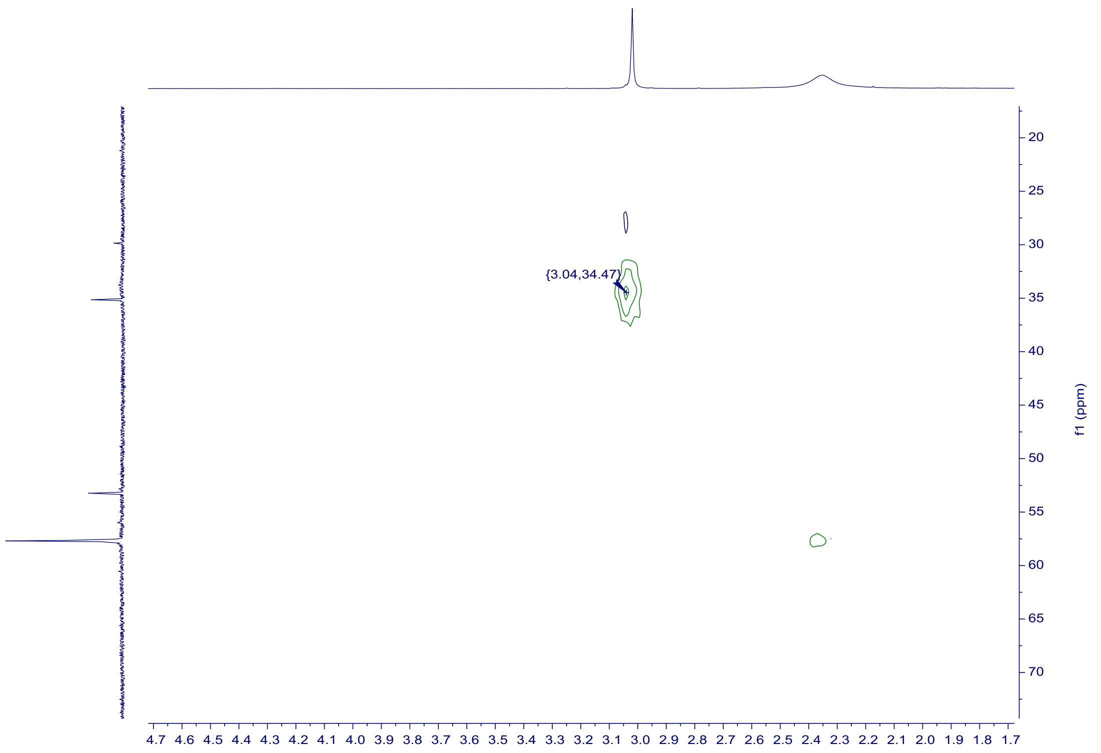

38 – HMBC (500 MHz, $CDCl_3$)

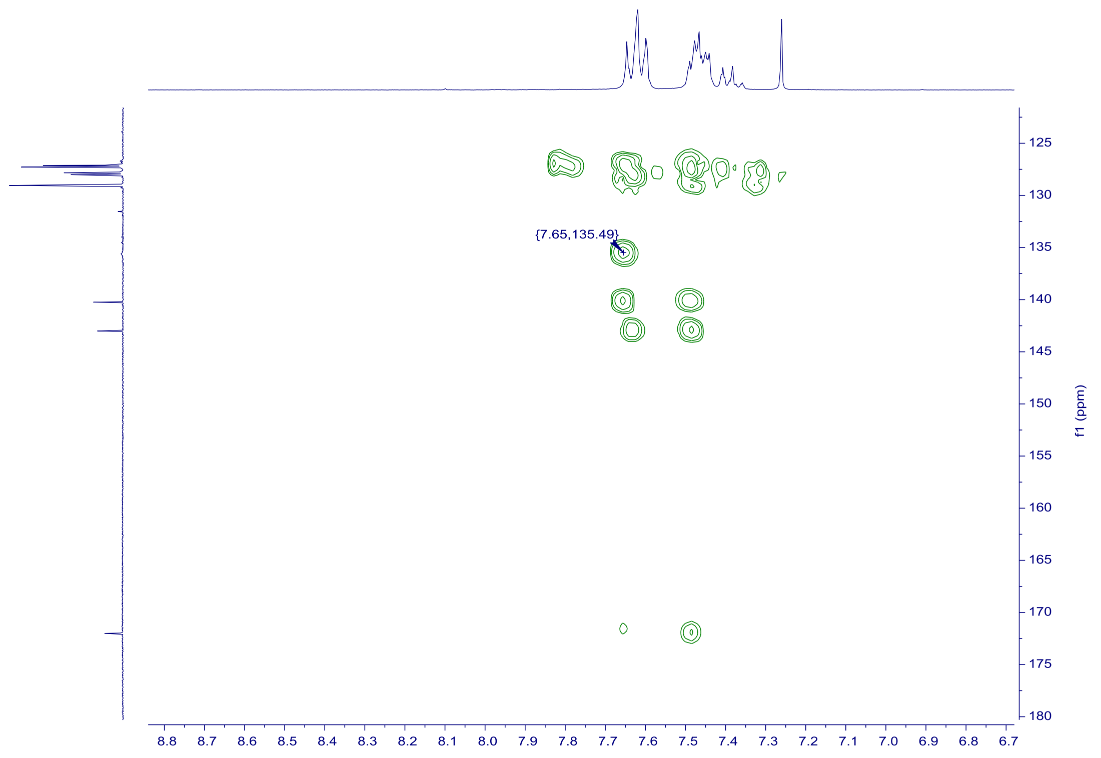

38 – ^{19}F NMR (376MHz, $CDCl_3$)

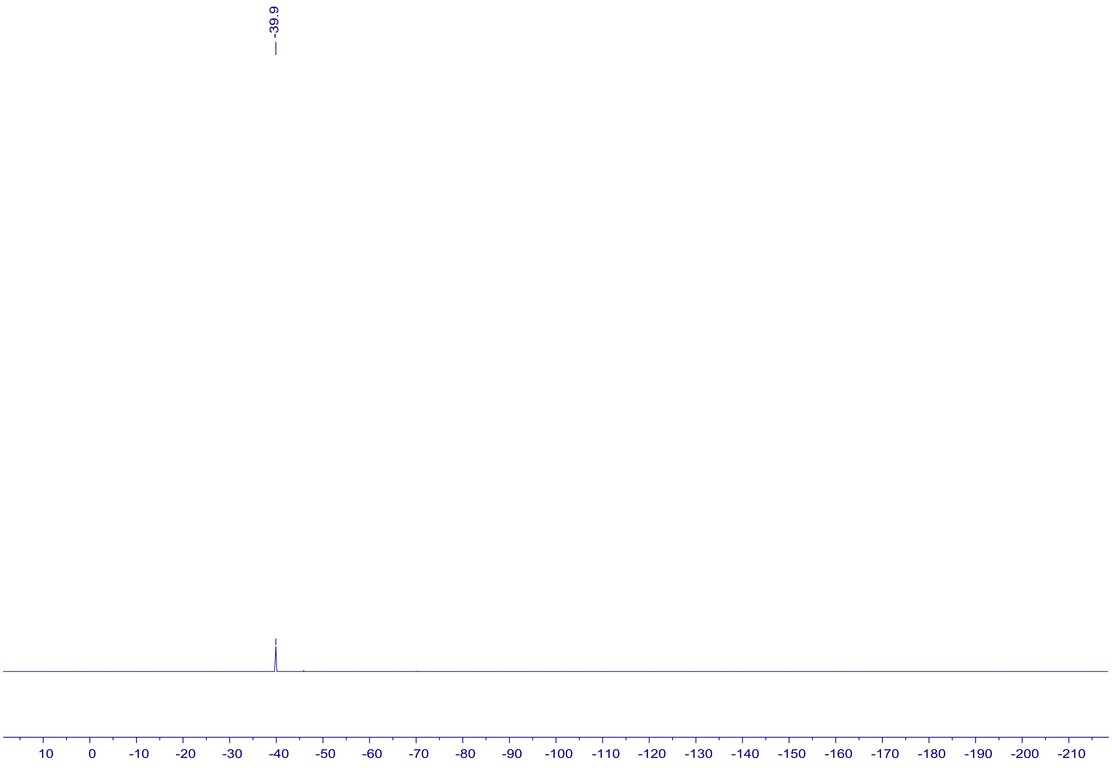

39 – ^{1}H NMR (500 MHz, $CDCl_3$)

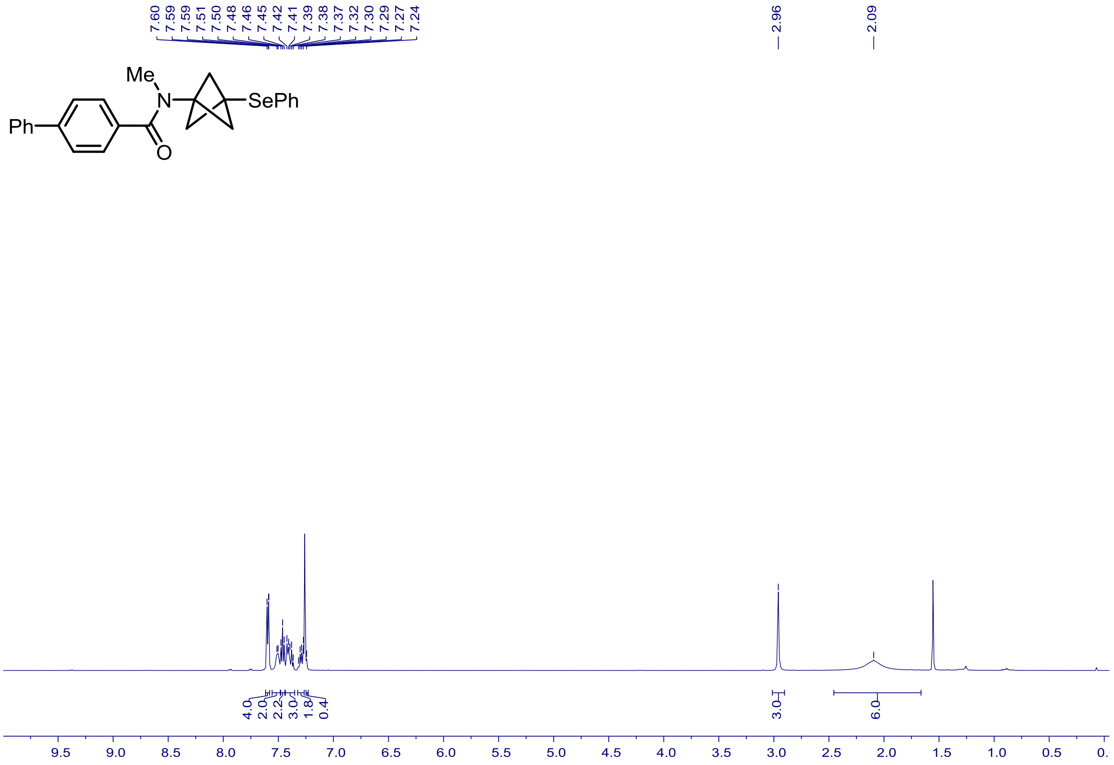

39– ^{13}C NMR (126MHz, $CDCl_3$)

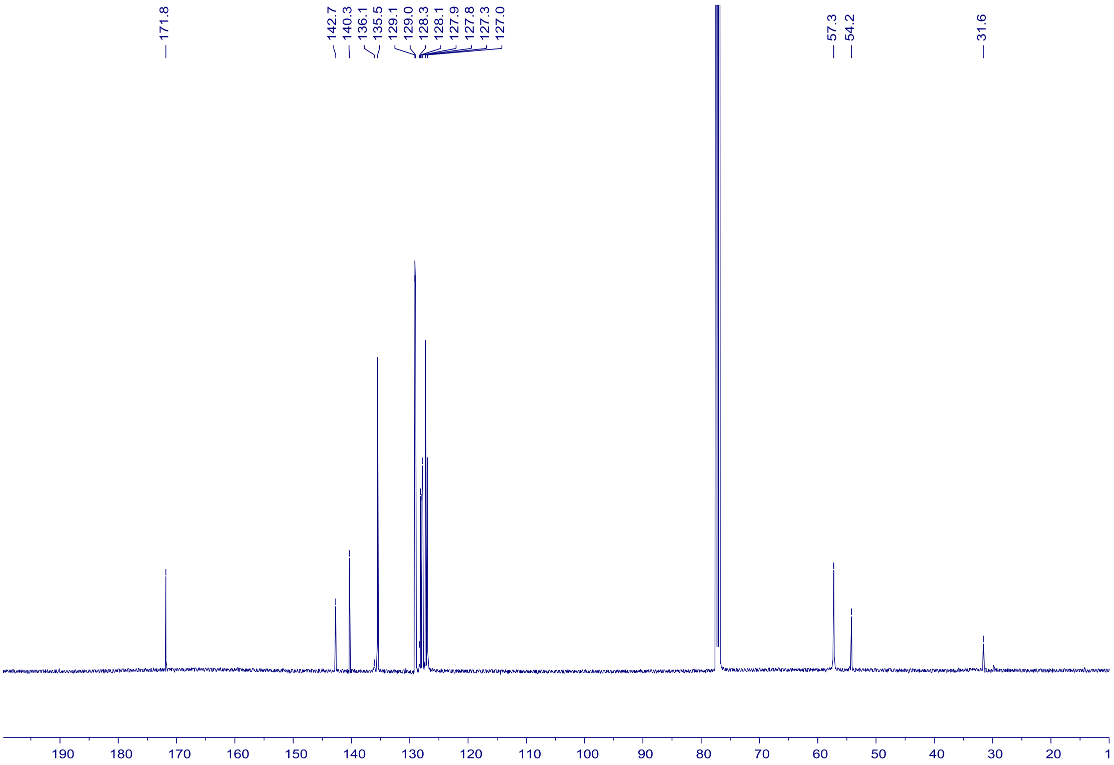

39 – HSQC (500 MHz, $CDCl_3$)

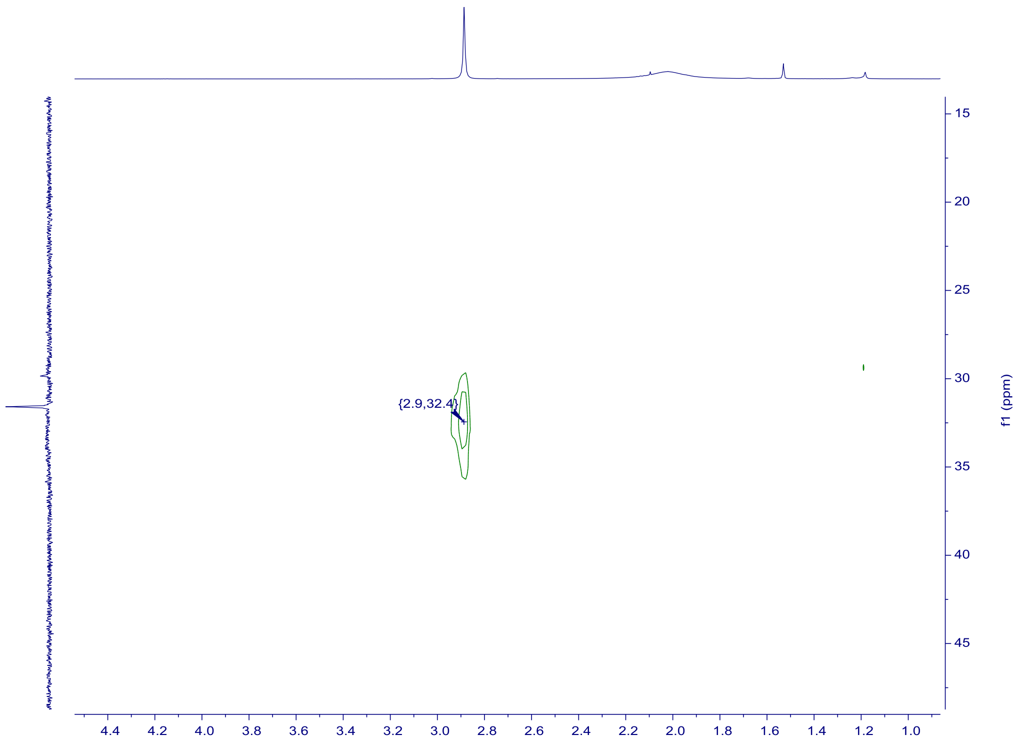

40 – ^{1}H NMR (500 MHz, $CDCl_3$)

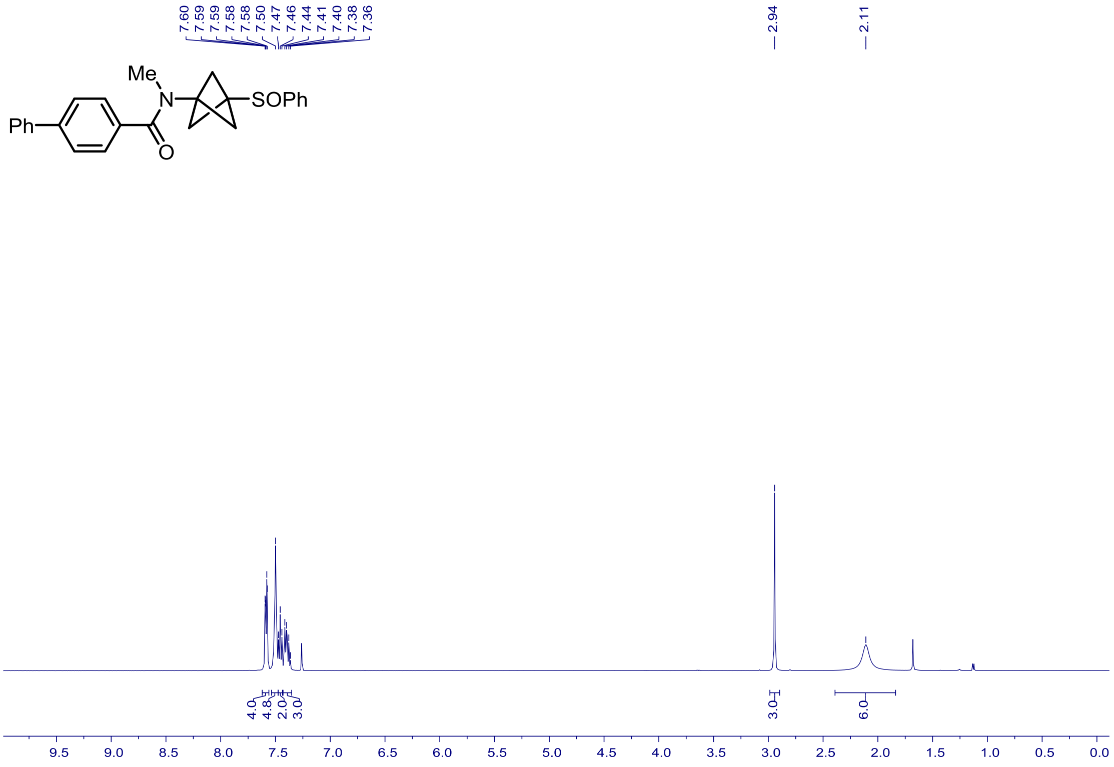

40– ^{13}C NMR (126MHz, $CDCl_3$)

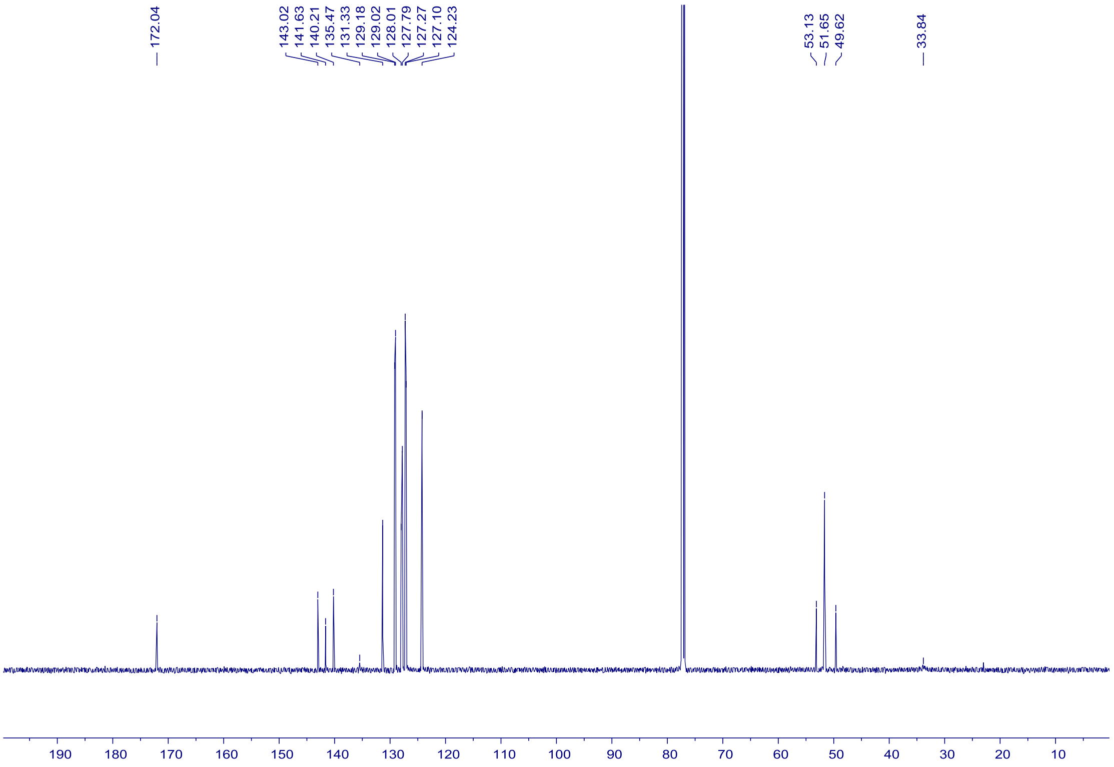

40 – HSQC (500 MHz, $CDCl_3$)

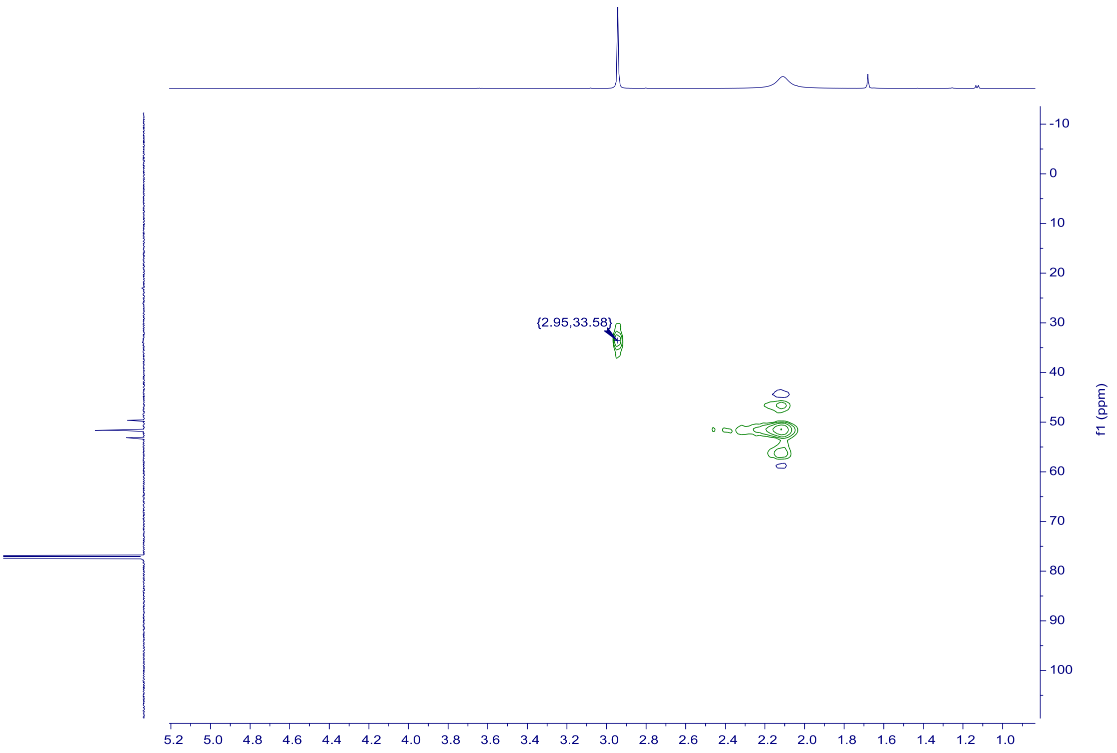

41 – ^{1}H NMR (500 MHz, $CDCl_3$)

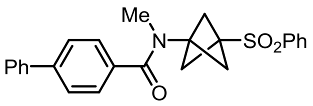

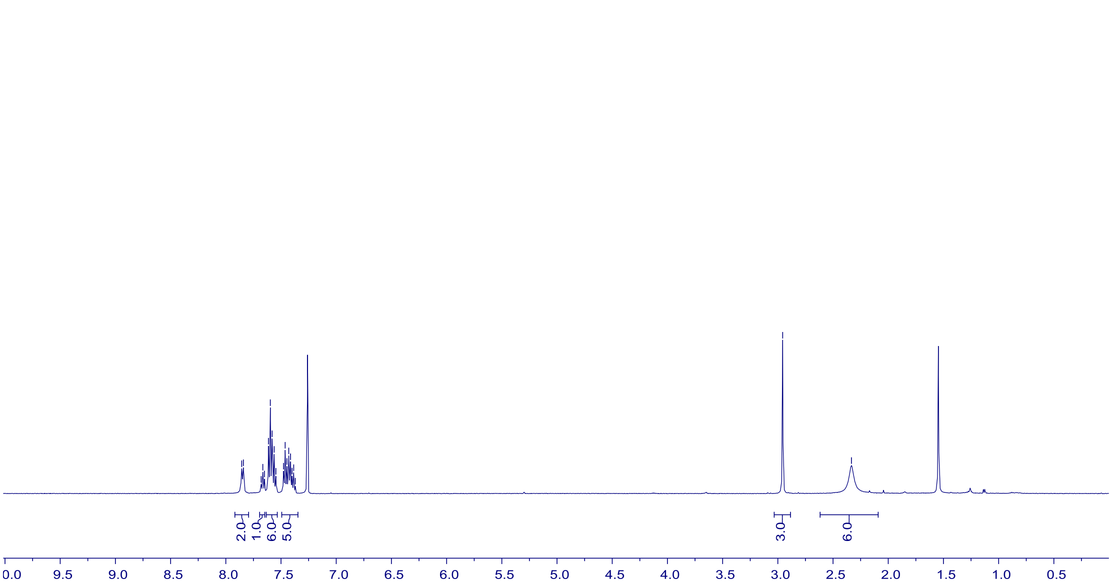

41 – ^{13}C NMR (126MHz, $CDCl_3$)

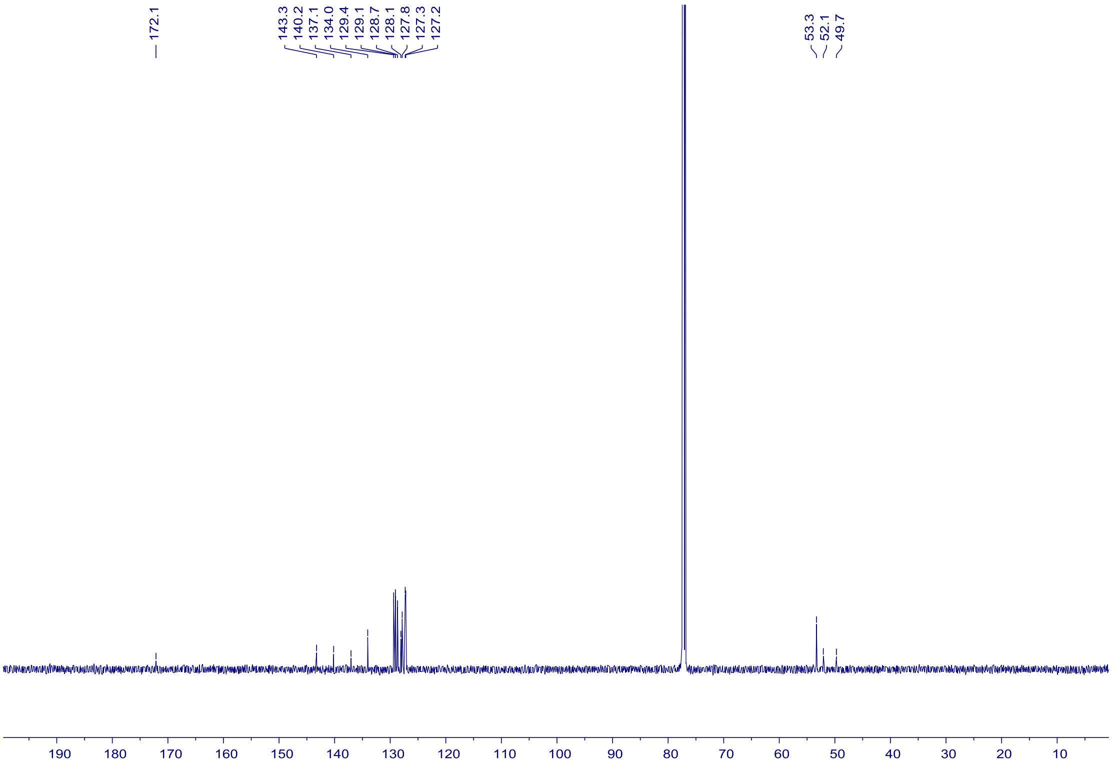

41 – HSQC (500 MHz, $CDCl_3$)

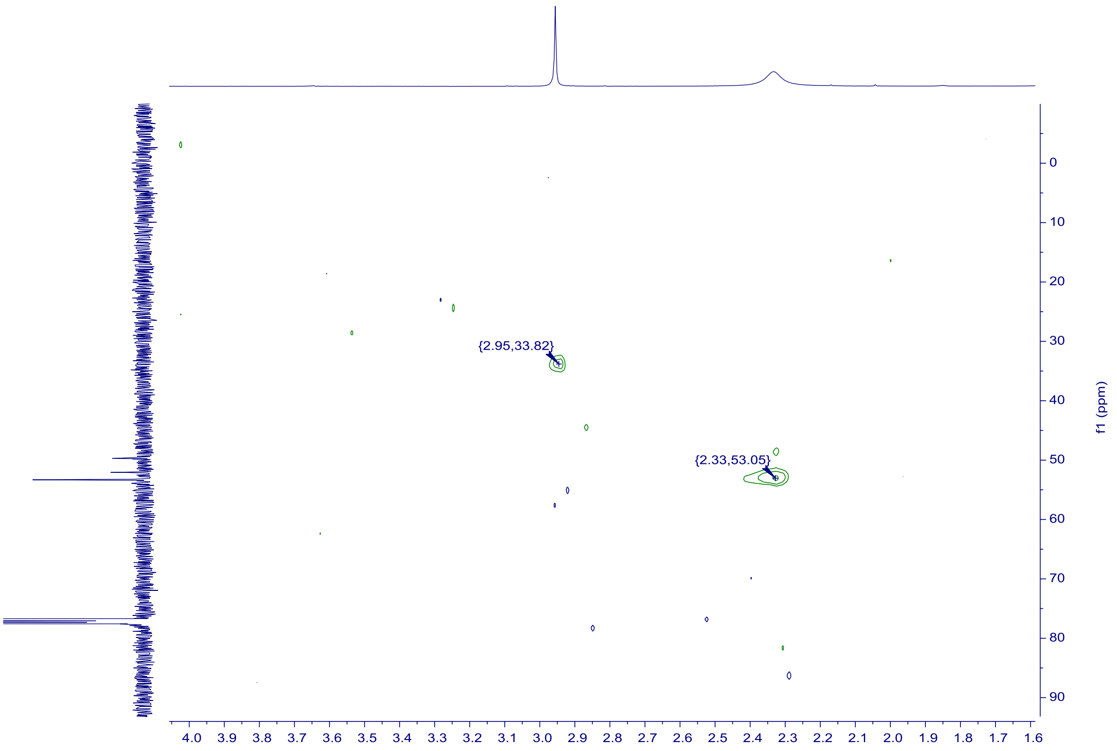

41 – HMBC (500 MHz, $CDCl_3$)

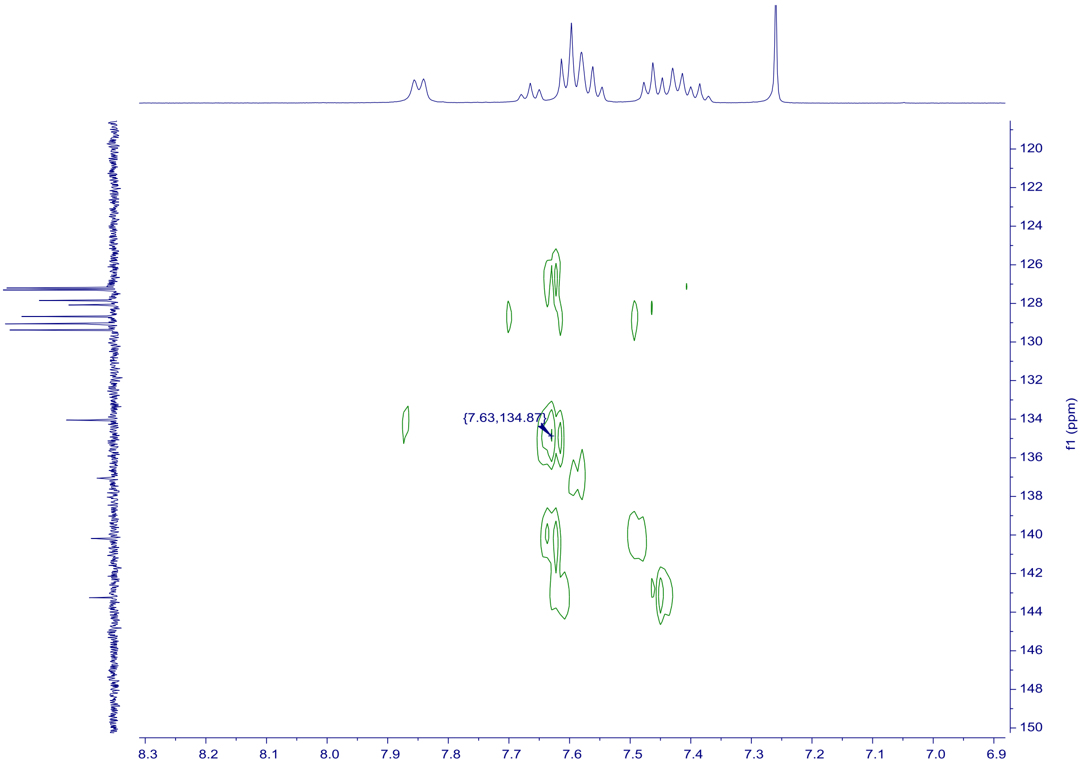

11 References:

1. Yuan, C.; Du, B.; Xun, M.-M.; Liu, B., *J. Am. Chem. Soc.* **2017,** *73*, 3622.

2. Morcillo, S. P.; Dauncey, E. M.; Kim, J. H.; Douglas, J. J.; Sheikh, N. S.; Leonori, D., *Angew. Chem. Int. Ed.* **2018,** *57*, 12945.

3. Goh, Y. L.; Tam, E. K. W.; Bernardo, P. H.; Cheong, C. B.; Johannes, C. W.; William, A. D.; Adsool, V. A., *Org. Lett.* **2014,** *16*, 1884.

4. Parr, R. G.; Yang, W., *Density-Functional Theory of Atoms and Molecules* **1989**, Oxford University Press, Oxford U.K.

5. Frisch, M. J.; Trucks, G. W.; Schlegel, H. B.; Scuseria, G. E.; Robb, M. A.; Cheeseman, J. R.; Scalmani, G.; BaronE, V.; Mennucci, B.; Petersson, G. A.; Nakatsuji, H.; Caricato, M.; Li, X.; Hratchian, H. P.; Izmaylov, A. F.; Bloino, J.; Zheng, G.; Sonnenberg, J. L.; Hada, M.; Ehara, M.; Toyota, K.; Fukuda, R.; Hasegawa, J.; Ishida, M.; Nakajima, T.; Honda, Y.; Kitao, O.; Nakai, H.; Vreven, T.; Jr., J. A. M.; Peralta, J. E.; Ogliaor, F.; Bearpark, M.; Heyd, J. J.; Brothers, E.; Kudin, K. N.; taroverov, V. N.; Keith, T.; Kobayashi, R.; Normand, J.; Raghavachari, K.; Rendell, A.; Burant, J. C.; Iyengar, S. S.; Tomasi, J.; CossI, M.; Rega, N.; Millam, J. M.; Klene, M.; Knox, J. E.; CrossI, J. B.; Bakken, V.; Adamo, C.; Jaramillo, J.; Gomperts, R.; Stratmann, R. E.; Yazyev, O.; Austin, A. J.; Cammi, R.; Pomelli, C.; Ochterski, J. W.; Martin, R. L.; Morokuma, K.; Zakrzewski, V. G.; Voth, G. A.; Salvador, P.; Dannenberg, J. J.; Dapprich, S.; Daniels, A. D.; Farkas, O.; Foresman, J. B.; Ortiz, J. V.; Cioslowski, J.; Fox, D. J., *Gaussian 09* **2013**, revision D.01; Gaussian, Inc.

6. Denningtom, R.; Keith, T.; Millam, J., *GaussView* **2009**, version 5; Semichem Inc.: Shawnee Mission, KS, 2009.

7. Pratt, L. M.; Truhlar, D. G.; Cramer, C. J.; Kass, S. R.; Thompson, J. D.; Xidos, J. D., *J. Am. Chem. Soc.* **2007,** *72* (8), 2962-2966.

8. Kanazawa, J.; Maeda, K.; Uchiyama, M., *J. Am. Chem. Soc.* **2017,** *139*, 17791.

9. Hay, P. J.; Wadt, W. R., *J. Chem. Phys.* **1985,** *82*, 270.

10. Hay, P. J.; Wadt, W. R., *J. Chem. Phys.* **1985,** *82*, 284.

11. Hay, P. J.; Wadt, W. R., *J. Chem. Phys.* **1985,** *82*, 299.

12. Stephens, P. J.; Devlin, F. J.; Chabalowski, C. F.; Frish, M. J., *J. Chem. Phys.* **1994,** *98*, 11623.

13. Becke, A. D., *J. Chem. Phys.* **1993,** *98*, 5648.

14. Becke, A. D., *J. Chem. Phys.* **1993,** *98*, 1372.

15. Lee, C.; Yang, W.; Parr, R. G., *Phys. Rev. B* **1988,** *37,* 785.

16. Vleeschouwer, F. D.; Speybroeck, V. V.; Waroquier, M.; Geerlings, P.; Proft, F. D., *Org. Lett.* **2007,** *9* (2721).

17. Hirshfeld, F. L., *Theoret. Chim. Acta* **1977,** *44*, 129.

18. DiLabio, G. A.; Pratt, D. A., *J. Phys. Chem. A* **2000,** *104*, 1938.

19. DiLabio, G. A.; LoFaro, A. D.; Wright, J. S., *J. Phys. Chem. A* **1999,** *103*, 1653.

20. Scot, A. P.; Random, L., *J. Phys. Chem.* **1996,** *100*, 16502.

21. Lalevee, J.; Allonas, X.; Fouassier, J.-P., *J. Org. Chem.* **2005,** *70,* 814.

22. Lalevee, J.; Allonas, X.; Genet, S.; Fouassier, J.-P., *J. Am. Chem. Soc.* **2003,** *125*, 9377.

23. Liu, J.; Niwayame, S.; You, Y.; Houk, K. N., *J. Org. Chem.* **1998,** *63*, 1064.
